# VaporSPOT: Parallel Synthesis of Oligosaccharides on Membranes

**DOI:** 10.1021/jacs.2c07285

**Published:** 2022-10-21

**Authors:** Alexandra Tsouka, Pietro Dallabernardina, Marco Mende, Eric T. Sletten, Sabrina Leichnitz, Klaus Bienert, Kim Le Mai Hoang, Peter H. Seeberger, Felix F. Loeffler

**Affiliations:** $ Department of Biomolecular Systems, Max Planck Institute of Colloids and Interfaces, Am Muehlenberg 1, 14476 Potsdam, Germany; & Institute of Chemistry and Biochemistry, Freie Universität Berlin, Arnimallee 22, 14195 Berlin, Germany; # GlycoUniverse GmbH & Co. KGaA, Am Muehlenberg 11, 14476 Potsdam, Germany

## Abstract

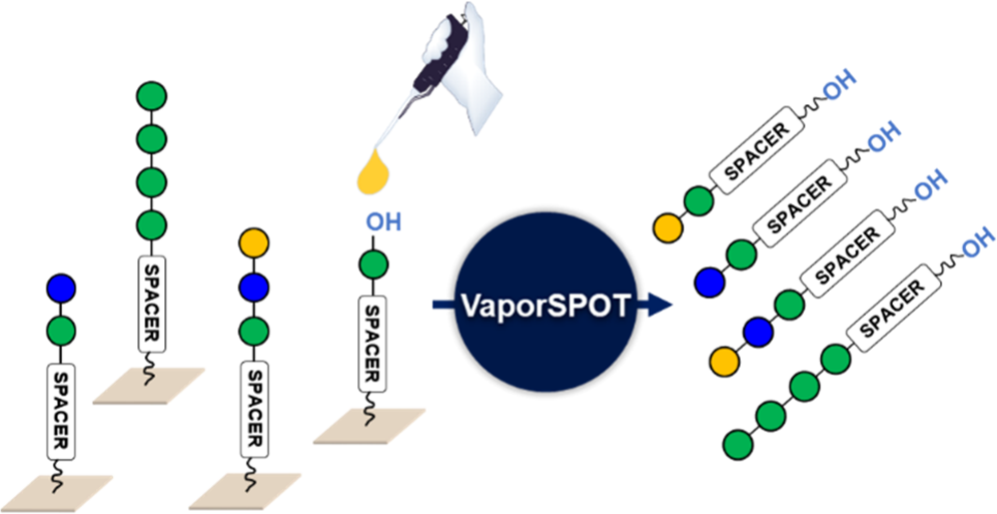

Automated chemical synthesis has revolutionized synthetic access to biopolymers in terms of simplicity and speed. While automated oligosaccharide synthesis has become faster and more versatile, the parallel synthesis of oligosaccharides is not yet possible. Here, a chemical vapor glycosylation strategy (VaporSPOT) is described that enables the simultaneous synthesis of oligosaccharides on a cellulose membrane solid support. Different linkers allow for flexible and straightforward cleavage, purification, and characterization of the target oligosaccharides. This method is the basis for the development of parallel automated glycan synthesis platforms.

## Introduction

Oligosaccharides are the most abundant biopolymers in nature and play a fundamental role in many biological functions. Despite their relevance in many processes of life, their role is still not sufficiently understood.^1,2^ Their structural heterogeneity, complexity, and diversity often make their isolation from natural sources laborious and their synthesis a cumbersome process. Therefore, different automated platforms for enzymatic,^3−5^ chemoenzymatic,^6,7^ and chemical oligosaccharide^8−12^ synthesis have been developed, giving access to complex and biologically valuable structures.^13−15^ In the last two decades, the development of automated glycan assembly (AGA) has enabled the successful synthesis of defined and complex synthetic oligosaccharide libraries of biological and medical interest,^8,15−21^ as well as very long polysaccharides including complex branching, up to 100-mers.^22,23^ Currently, AGA is used to prepare one single oligosaccharide at a time. Parallel oligosaccharide synthesis would be more cost- and time-efficient. Compared to the well-established parallel peptide and oligonucleotide synthesis methods,^24,25^ the parallel synthesis of oligosaccharides remains a major challenge. Early work by the Kahne group relied mainly on acylations to generate diversity and study the interaction of disaccharides with carbohydrate-binding proteins.^26^ Mrksich and colleagues demonstrated the on-chip synthesis of a collection of different disaccharides and subsequent enzymatic modification.^27^ Recently, Heo et al. reported their on-chip sequential enzymatic glycosylation strategy for the synthesis of several Globo H-related oligosaccharides on a DNA linker, enabling characterization and purification of the synthetic structures.^28^

None of these methods were advanced beyond the proof-of-principle stage, since the chemical synthesis requires inert and temperature-controlled glycosylation conditions, making automation a challenging process. Enzymatic synthesis overcomes these issues, but still, only a limited number of glycosyltransferases is available.^29^

Here, we present the VaporSPOT method for parallel oligosaccharide synthesis that overcomes these limitations. SPOT synthesis, initially developed by Frank et al.,^30^ can be performed manually or automated^31−34^ and is commonly used to simultaneously generate peptide, small-molecule, or glycopeptide libraries.^34−37^ The original SPOT method follows the 9-fluorenylmethoxycarbonyl (Fmoc)-based solid-phase synthesis protocol under ambient conditions, using cellulose membranes as a solid support. Cleavage of the products can be achieved after treatment with strong bases or acids, giving access to a library of soluble and/or cellulose-tethered peptides. However, SPOT synthesis is incompatible with chemical carbohydrate synthesis, since the conditions are neither inert nor temperature-controlled. To solve this problem, we devised a novel method and designed a setup to ensure controlled conditions suitable for glycosylation reactions. With this, we show the parallel synthesis of six different oligosaccharides and up to four residues in length in the micromolar scale (∼1 μmol). In contrast to other solid-phase approaches, it is a simple setup that saves time and reagents by parallelization.

## Results and Discussion

**Figure 1:**
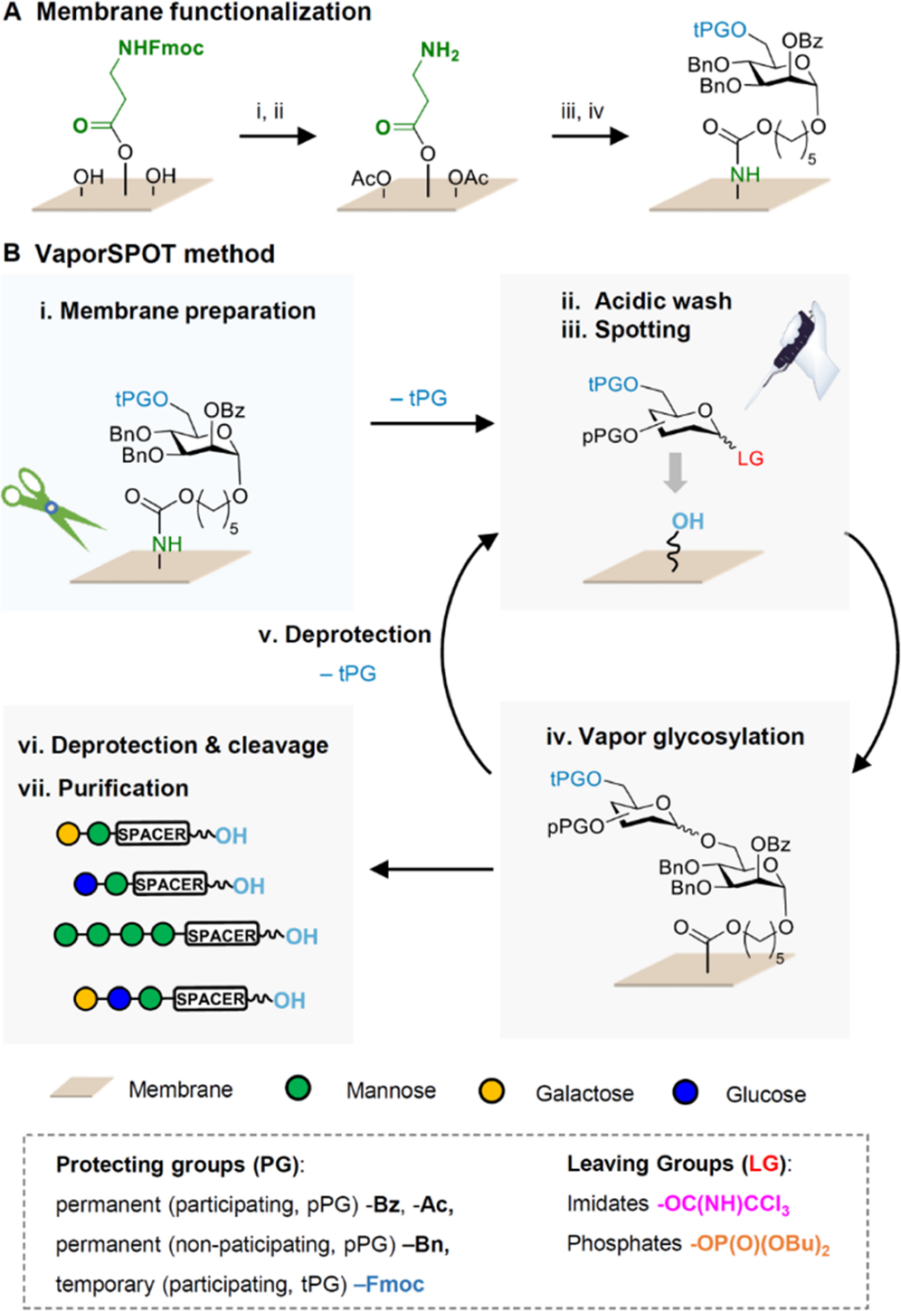
Schematic representation of VaporSPOT synthesis: (A) reagents and conditions for membrane functionalization: (i) capping 10% Ac_2_O, 2% MsOH in DCM, rt, 30 min; (ii) 20% piperidine in DMF, rt, 20 min; (iii) attachment of the linker, rt, overnight; and (iv) capping 10% Ac_2_O, 20% DIPEA in DMF, rt, 30 min. (B) Modules of the VaporSPOT process: (i) preparation of the membrane; (ii) acidic wash of the membrane; (iii) spotting of the building block; (iv) chemical vapor glycosylation; (v) removal of tPG; (vi) deprotection of pPGs and release of oligosaccharides from the solid support; and (vii) purification and characterization of synthesized structures.

**Figure 2:**
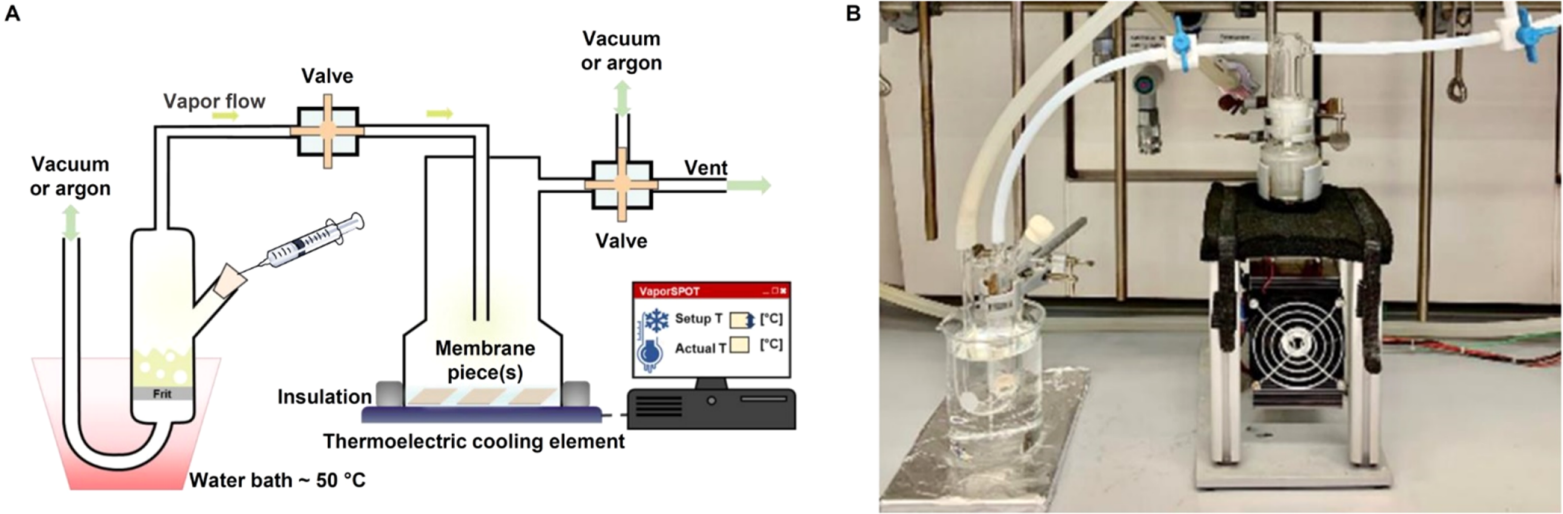
VaporSPOT setup: (A) schematic representation and (B) experimental setup of the custom-built apparatus for parallel and temperature-controlled oligosaccharide synthesis on membranes.

The VaporSPOT process under inert argon conditions was designed for building block delivery at room temperature and subsequent chemical vapor glycosylation at low temperature. The synthesis begins with the functionalization and preparation of the cellulose membrane (Figure 1), which was preloaded with a base-labile mannopyranoside linker (Sections C and E in the Supporting Information), bearing an Fmoc group on the C-6 position, after deprotection, serving as the nucleophile for the first glycosylation. Therefore, the commercially available Fmoc-β-alanine esterified cellulose membrane (Figure 1A) was acetylated to minimize unspecific glycosylation reactions. After Fmoc deprotection, an Fmoc-protected mannopyranoside was attached (Figure S2) and unreacted free amino groups of the β-alanine were acetylated to minimize side reactions (see Section E in the Supporting Information). The mannopyranoside linker was Fmoc-deprotected and an acidic wash of the membrane was performed to remove any residual base, followed by spotting of the first building block and then drying under high vacuum. One or several spotted membranes were transferred to the bottom of the custom-built instrument (Figure 2) and cooled to −15 °C. Activation of the glycosyl donor, similar to batch or solid-phase syntheses, was achieved by delivery and condensation of TMSOTf and dichloromethane vapor inside the glycosylation chamber (2 min). Then, the temperature was slowly increased to rt and maintained for 30 min. After completion, the remaining condensate was removed from the glycosylation chamber under high vacuum. The membrane(s) were transferred to a Petri dish and washed with dichloromethane and dimethylformamide. For higher coupling efficiencies with poorly reactive and/or sterically hindered building blocks, coupling can be repeated. Removal of the temporary protecting group with piperidine unmasks the nucleophile for the next synthesis cycle. These steps are repeated until the target structure(s) are formed (Figure 1B, modules ii–v). Simultaneous deprotection of the ester protecting groups and release of the product(s) from the surface was achieved by sodium methoxide, followed by purification and characterization.

**Figure 3:**
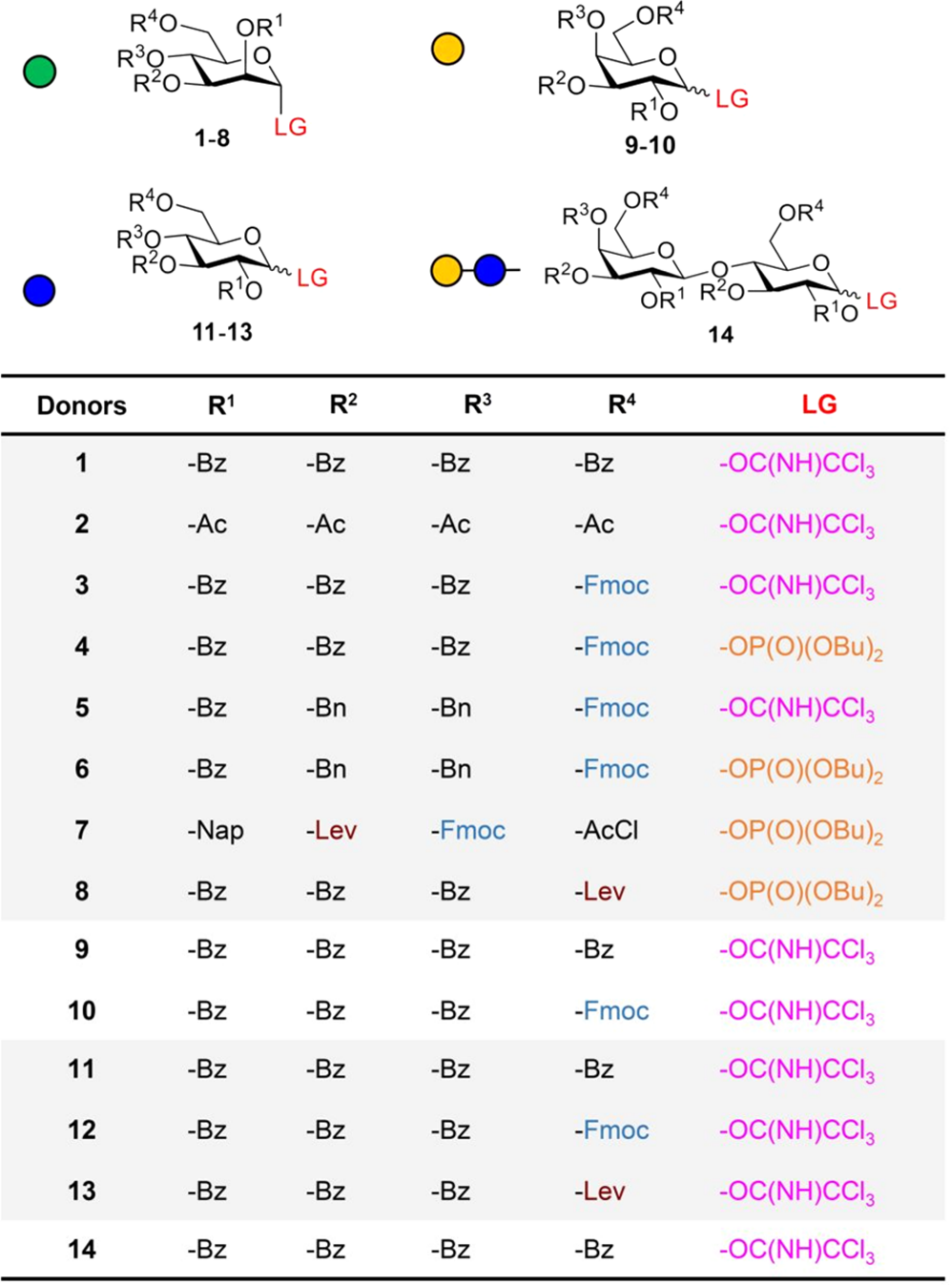
Synthesized building blocks for VaporSPOT synthesis.

**Figure 4:**
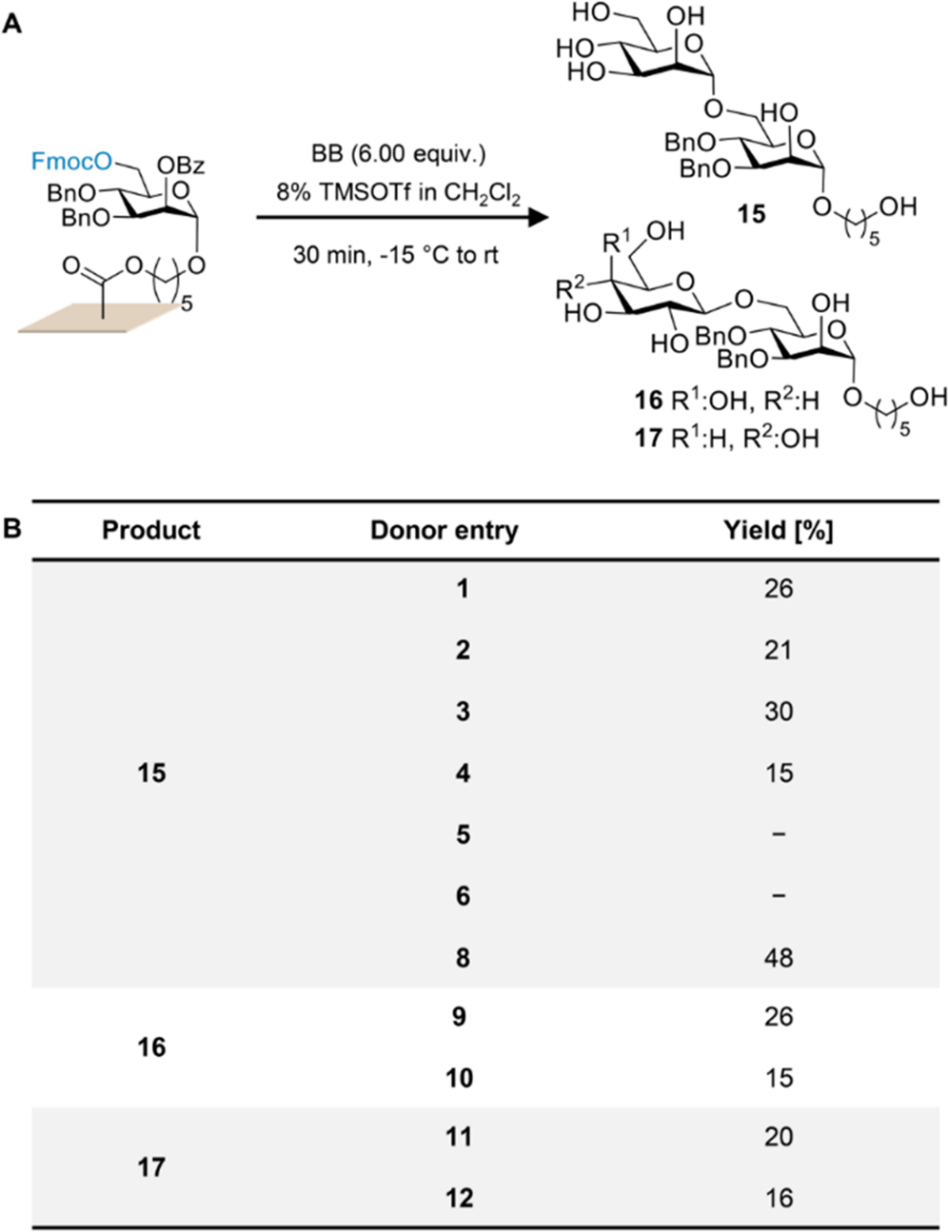
(A) Screening of BBs **1**–**6** and **8**–**12** under the same vapor glycosylation conditions (8% TMSOTf in dichloromethane) for the synthesis of dimers **15**–**17**. (B) Obtained yields after cleavage and characterization of the target dimers.

For successful glycosylations, two main parameters had to be optimized: the temperature inside the glycosylation chamber and the amount and concentration of the activator solution to be delivered. Both affect the reactivity of the building blocks and the yield.^38−41^ For the preparation of different oligosaccharides, 14 building blocks (BBs, Figure 3) were either synthesized following established protocols^13,42−49^ or prepared from commercially available precursors (trichloroacetimidates **3**, **5**, and **9** and phosphates **6** and **8**, see Section B in the Supporting Information). To optimize the coupling conditions, glycosyl donors **1**–**6** and **8**–**12** were screened, using 8% TMSOTf in dichloromethane (Figure 4A), to furnish the corresponding dimers **15**–**17** in different yields (Figure 4B). While perbenzoylated building blocks (**1**, **9**, **11**) show relatively similar results, mannoside **3**, with an Fmoc temporary protecting group on the C-6 position, gave the best glycosylation outcome in comparison to galactoside **10** and glucoside **12**, due to its higher reactivity (arming/disarming effect)^41,50^ at these temperatures. Moreover, no glycosylation was observed with more reactive BBs **5** and **6** with two electron-donating groups. This is likely due to the currently limited minimum temperature (−15 °C) achievable by the setup.

However, a significantly higher yield was obtained with building block **8**, where the Fmoc group on the C-6 position is replaced with the more electron-withdrawing Lev group, yielding 48% of the desired di-mannopyranoside **15** under the exact same conditions.

**Scheme 1:**
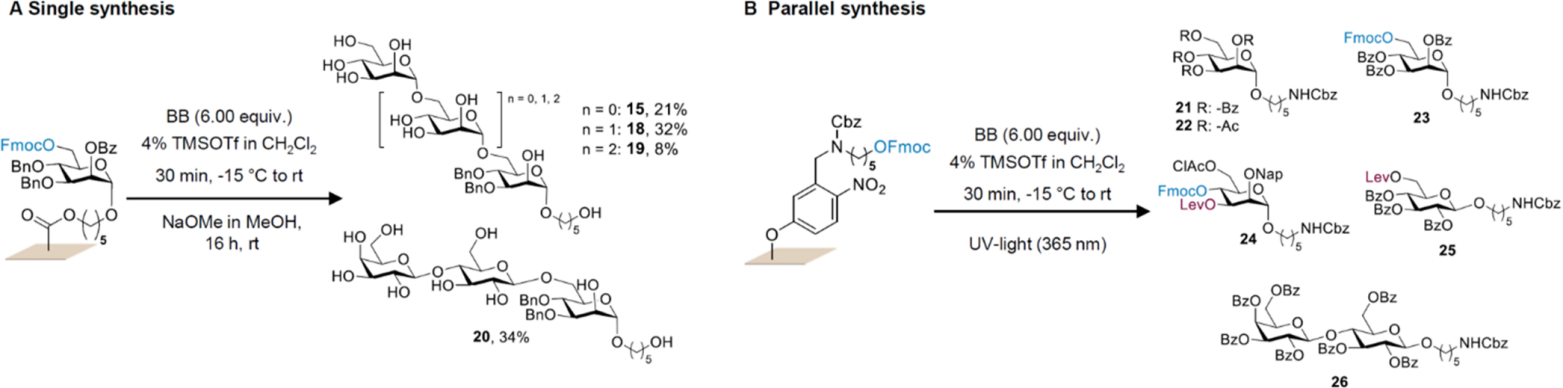
Synthesized Oligosaccharide Collection Using the VaporSPOT Oligosaccharide Synthesis Approach on Functionalized Membranes (A) Structures **15–20** were synthesized with a base-labile linker, fully characterized and purified. (B) Structures **21–26** were synthesized in parallel, performing two glycosylation cycles, using a photolabile linker, characterized by MALDI.

Next, trimannoside **18** and tetramannoside **19** were synthesized, using mannopyranosides **1**, **3**, and **4** for chain elongation. While BB **8** resulted in a higher yield, the Lev deprotection would require extensive optimization on the cellulose membrane. Thus, we selected the already established Fmoc deprotection strategy to synthesize longer structures. In the first experiments, partial decomposition of the membrane and reproducibility issues were observed during the synthesis of **18** under the reported glycosylation conditions. Reduction of the activator amount to 4% provided trisaccharide **18** in 32% yield, retaining the integrity of the membrane. Using the optimized activator solution, tetramannoside **19** was successfully synthesized (8 steps) in an overall yield of 8%. Furthermore, with a 4% activator solution, trimer **20** was obtained in 34% yield with β-(1-6) and β-(1-4) linkage, starting from disaccharide **14** (Scheme 1A). All final structures **15**–**20** were characterized using mass spectrometry (MS) and nuclear magnetic resonance spectroscopy (NMR). Both purity and anomeric purity of the final structures were analyzed by high-performance liquid chromatography (HPLC) and decoupled ^1^H–^13^C heteronuclear single quantum coherence spectroscopy (HSQC NMR).

Encouraged by these results, the parallel synthesis of oligosaccharides was investigated (Scheme 1B). To assess, whether diffusion or contamination can occur between different membrane pieces placed in close proximity inside the setup, a photolabile linker was designed and synthesized (Sections D and E in the Supporting Information). With the current set of BBs, the previously used base-labile linker would deliver partially deprotected compounds of mostly indistinguishable molecular weight, since cleavage from the solid support and deprotection of the ester and carbonyl protecting groups occur simultaneously. In contrast, the photolabile linker will deliver protected compounds with distinguishable molecular weight. Using the same experimental setup, reaction time, and the 4% activator solution, six different glycosyl donors **1**–**3**, **7**, **13**, and **14**, bearing different protecting groups, were coupled onto six individual cellulose membrane pieces in parallel. The glycosylation reaction on each membrane piece was repeated once, while the positions of the membranes were shuffled between the glycosylations to detect any possible diffusion or contamination (Figure S9 and Section H in the Supporting Information). The desired products **21**–**26** were obtained after parallel cleavage under UV light (365 nm) and detected based on their molecular weight using MALDI-ToF mass spectrometry. During the parallel synthesis, no diffusion/contamination between the different membranes was observed. Nevertheless, further characterization was not possible due to inefficient photocleavage from the solid support.

Finally, to show the versatility of the VaporSPOT approach, a parallel reaction on a functionalized glass slide was performed (Figure S10 and Section I in the Supporting Information). Six glycosyl donors were spotted, glycosylated under chemical vapor, and the protecting groups were removed without cleavage of the synthesized structures from the solid support. Validation of the formed disaccharides was achieved by staining with a fluorescently labeled lectin.

## Conclusions

SPOT synthesis is widely used for the parallel synthesis of peptides. Here, we developed the VaporSPOT synthesis method for the parallel synthesis of oligosaccharides on cellulose membranes under inert and temperature-controlled chemical vapor conditions. This method offers a flexible and cost-efficient way to rapidly screen the glycosylation outcome of different glycosyl donors in parallel and synthesize oligosaccharides in good purity on the micromolar scale. In a parallel reaction approach, diffusion or contamination between the different spotted glycosyl donors was ruled out.

Future optimization of the methodology, including different protecting groups and linkers, will lead to more complex structures with higher yields. For example, the replacement of the Fmoc protecting group with the more electron-withdrawing Lev group on the C-6 position should lead to higher glycosylation yields. Besides, technical improvements (e.g., minimum achievable temperature) may enable more flexible chemical strategies.

Furthermore, other solid supports, such as cross-linked cellulose,^51^ polypropylene, or Teflon-patterned membranes,^34^ may further improve the spot density, substrate stability, and synthesis yield. The same VaporSPOT approach may be further expanded to high-throughput glycan array synthesis on functionalized glass slides or even beyond glycochemistry, for precisely controlled polymerization or cross-coupling reactions. Together with automated spotting of building blocks, this should enable even higher parallelization. Such oligosaccharide collections are ideal for microarray production, to drastically accelerate the screening of glycan–glycan binding protein interactions.

## Supporting Information Available

All experimental details regarding the VaporSPOT method; building block synthesis; and characterization (PDF)
