# Supplementary material for: VaporSPOT: Parallel Synthesis of Oligosaccharides on Membranes
Source: J Am Chem Soc. 2022 Oct 21;144(43):19832–7. doi: 10.1021/jacs.2c07285 (PMC9634802; doi:10.1021/jacs.2c07285)
Supplement: Supplementary file 1 — ja2c07285_si_001.pdf [file ja2c07285_si_001.pdf]

VaporSPOT: Parallel Synthesis of Oligosaccharides on Membranes

Alexandra Tsouka+,[1,2] Pietro Dallabernardina+,[1] Marco Mende,[1] Eric T. Sletten,[1] Sabrina Leichnitz,[1,2] Klaus Bienert,[1] Kim Le Mai Hoang,[3] Peter H. Seeberger,[1,2] and Felix F. Loeffler[1]*

[1] Department of Biomolecular Systems, Max-Planck-Institute of Colloids and Interfaces, Am Mühlenberg 1, 14476 Potsdam, Germany

[2] Institute of Chemistry and Biochemistry, Freie Universität Berlin, Arnimallee 22, 14195 Berlin, Germany

[3] GlycoUniverse GmbH & Co. KGaA, Am Muehlenberg 11, 14476 Potsdam, Germany

***Corresponding author:**

Felix F. Loeffler
E-mail: felix.loeffler@mpikg.mpg.de

Table of Content

A. General remarks 3

B. Building block synthesis 4

C. Synthesis of base-labile linker (28) 35

D. Synthesis of photo-labile linker (29) 41

E. Functionalization of cellulose membrane 48

Quantification/loading determination of functionalized membrane 50

F. VaporSPOT glycosylation 50

Preparation of stock solutions 50

Homebuilt setup 51

Temperature profile and regulation system 51

Concentration of activator solution 52

Concentration/equivalents of glycosyl donor 53

Module A: Membrane preparation for synthesis (39 min) 53

Module B: Acidic wash prior to glycosylation (58 min) 54

Module C: Spotting of BB (31 min) 54

Module D: Vapor glycosylation (106 min) 54

Module E: Fmoc-deprotection (35 min) 54

Post-Synthesis manipulation 54

Purification & Characterization 55

G. Oligosaccharide synthesis 56

H. Parallel synthesis 76

I. VaporSPOT synthesis on glass slide 81

References 82

A. General remarks

All applied solvents, deuterated solvents (99.5 atom% D), and chemicals were purchased from common suppliers such as Sigma-Aldrich, Alfa Aesar, Tokio Chemical Industry (TCI), Thermo Fischer Scientific, Acros Organics, Iris Biotech, Merck, and used without further purification. For High-Performance Liquid Chromatography (HPLC) solvents with corresponding quality were used. All starting materials such as **3.1**, **5.1**, **10.1**, **29.3** and all starting glycopyranoside building blocks used for the synthesis of the targeted compounds were purchased from GlycoUniverse GmbH & Co KGaA, apart from the lactose starting material purchased from Tokio Chemical Industry (TCI). The cellulose membrane used was acquired from AIMS Scientific Products GmbH. If not mentioned otherwise, saturated aqueous solutions of inorganic salts were used. Thin layer chromatography (TLC) using silica gel coated aluminium plates (MACHEREY-NAGEL, pre-coated TLC sheets ALUGRAM® Xtra SIL G/UV254 or Merck, pre-coated TLC sheets 60 F254) was applied to monitor reactions until completion. Compounds were visualized by UV light (λ = 254 nm) or stained either with Seebach (phosphomolybdic acid hydrate, cerium(IV) sulfate tetrahydrate, sulfuric acid and water), *p*-anisaldehyde (*p*-anisaldehyde, acetic acid, sulfuric acid and ethanol) or with potassium permanganate solution (potassium permanganate, potassium carbonate, sodium hydroxide and water). Flash column chromatography was carried out by using MACHEREY-NAGEL silica gel 60 (0.040 × 0.063 mm) and quartz sand. Final deprotected oligosaccharides were lyophilized using a Christ Alpha 2–4 LD plus freeze dryer. The spectra were recorded on Varian 400-MR (400 MHz), Bruker Ascend 400 (400 MHz), Varian 600-MR (600 MHz), or Bruker Biospin AVANCE700 (700 MHz) spectrometer. Chemical shifts δ are reported in ppm and are adjusted to internal standards of the residual proton signal of the deuterated solvent ($CDCl_3$: 7.26 ppm for ^{1}H and 77.0 ppm for ^{13}C, D_2O: 4.79 ppm for ^{1}H). The spectra were measured at room temperature. Having symmetrical signals, the center of the signal is given and for multiplets the area. The following characterization was used: s = singlet, sbr = singlet broad, d = doublet, t = triplet, q = quartet, m = multiplet or combinations like dd = doublet of doublet or dt = doublet of triplet and m = multiplet. Coupling constants (*J*) are given in Hz. The spectra were evaluated according to 1st order. For ^{1}H NMR spectra, the correlation of the signals was done according to the multiplicities. IR spectra were recorded on a FT-IR spectrometer from Perkin-Elmer. High-resolution mass spectrometry (HRMS) was conducted on a Waters Xevo G2-XS QTof device using ESI (electrospray ionization). Low-resolution mass spectrometry (LRMS) were obtained using an HPLC-System Serie 1100 coupled with ESI-single quadrupole from Agilent. The abbreviation $[M+Na]^+$ refers to the product–sodium adduct. ESI mass spectra were run on IonSpec Ultima instruments and MALDI-ToF autoflex™ (Bruker) instrument. Analytical reverse phase HPLC was performed on an HPLC-System Serie 1200 from Agilent using Synergi 4µm Hydro-RP 80 Å column (250 × 4.6 mm) and preparative reverse phase HPLC was performed on an Agilent 1200 using a preparative Synergi 4µm Hydro-RP 80 Å column (250 × 10 mm). UV-cleavage of the photo-labile linker was performed in a Vilber Lourmat black light ((VL.208.BL) lamp emitting 365 nm UV light with fractions of visible light (wavelength [nm]: 365, filter size [mm]: 230 × 60, power [W]: 2 x 8).

B. Building block synthesis

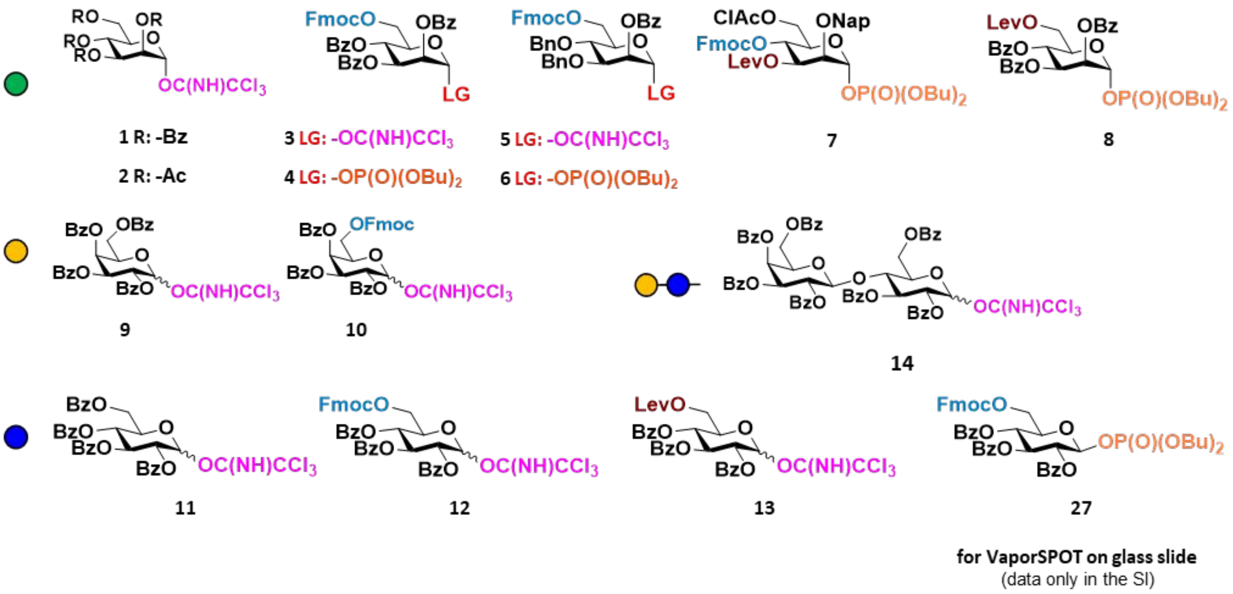

Building blocks **1**, **2**, **4**, **7**, **9, 11-14** were synthesized as reported and their analytical data agree with the literature.[1–10]

Synthesis of 2,3,4-*O*-tri-benzoyl-6-*O*-(9-fluorenylmethoxycarbonyl)-α-D-mannopyranosyl trichloroacetimidate (3)

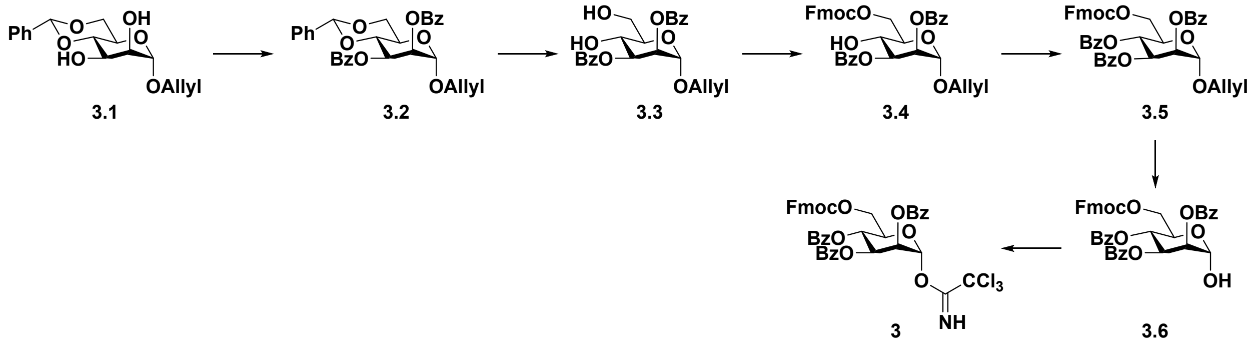

*Allyl 2,3-di-O-benzoyl-4,6-O-benzylidene-α-D-mannopyranoside (**3.2**)*

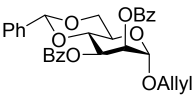

3.2

Allyl 4,6-*O*-benzylidene-α-D-mannopyranoside (**3.1**) (5.00 g, 16.2 mmol, 1.00 equiv.) was dissolved in pyridine (50 mL) and the solution was cooled to 0°C. Benzoyl chloride (BzCl) (4.90 mL, 42.2 mmol, 2.60 equiv.) was added dropwise to the solution and left to react overnight at room temperature (rt). After completion, the mixture was poured into iced water; the precipitate was filtered off and washed with water. The solid was dissolved in DCM (100 mL) and washed with hydrochloric acid (1 M, 100 mL), $NaHCO_3$-solution (100 mL), and water (100 mL). The organic layer was dried over Na_2SO_4 and the solvent was removed under reduced pressure. The crude product was used without any further purification.

*Allyl 2,3-di-O-benzoyl-α-D-mannopyranoside (**3.3**)*

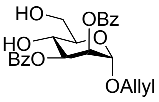

3.3

Trifluoroacetic acid (TFA) (9.17 mL, 119 mmol, 7.50 equiv.) and water (1.23 mL, 68.3 mmol, 4.30 equiv.) were added to a solution of **3.2** (8.20 g, 15.9 mmol, 1.00 equiv.) in DCM (215 mL). The mixture was stirred for 3 h, diluted with DCM (200 mL), washed with 10% (w/v) $NaHCO_3$-solution until neutral pH and 10% (w/v) NaCl-

solution. The organic layer was dried over Na_2SO_4, filtered, and concentrated. The crude product was purified by flash column chromatography using a mixture of hexane/ethyl acetate (1:2) as eluent. The desired product was obtained as a white foam in 84% yield over two steps (5.70 g, 13.28 mmol). ^{1}H NMR (400 MHz, $CDCl_3$): δ = 8.10 – 8.05 (m, 2H*, -Ar)*, 7.97 – 7.88 (m, 2H*, -Ar)*, 7.64 – 7.57 (m, 1H*, -Ar)*, 7.55 – 7.46 (m, 3H*, -Ar)*, 7.38 – 7.33 (m, 2H*, -Ar)*, 6.01 – 5.85 (m, 1H, CH_2=C*H*-) 5.61 (m, 2H, *H*-2, *H*-3), 5.36 (dd, *J* = 17.3, 1.6 Hz, 1H, CH*H*=C*H*-), 5.26 (dd, *J* = 10.4, 1.3 Hz, 1H, CH*H*=C*H*-), 5.05 (d, *J* = 1.6 Hz, 1H, *H*-1), 4.40 – 4.20 (m, 2H, *H*-4, *H*-6a), 4.14 – 4.06 (m, 1H, *H*-6b), 3.98 (d, *J* = 3.8 Hz, 2H, -CH_2-CH=), 3.90 (dt, *J* = 9.6, 3.7 Hz, 1H, *H*-5), 2.77 (s, 1H, -*OH*) ppm; ^{13}C NMR (101 MHz, $CDCl_3$): δ = 166.9, 165.5, 133.5, 133.4, 133.1, 129.8, 129.8, 129.4, 129.2, 128.6, 128.4, 118.2, 96.7, 73.2, 72.4, 70.6, 66.9, 62.3 ppm; IR (neat) ν_{max}: 3468, 2964, 2345, 1726, 1601, 1452, 1277,1114, 1071 cm^{-1}; ESI-HRMS: m/z [M+Na]$^+$ calcd. for $C_{23}H_{24}O_8Na$: 451.1363 found 451.1369.

^{1}H NMR ($CDCl_3$)

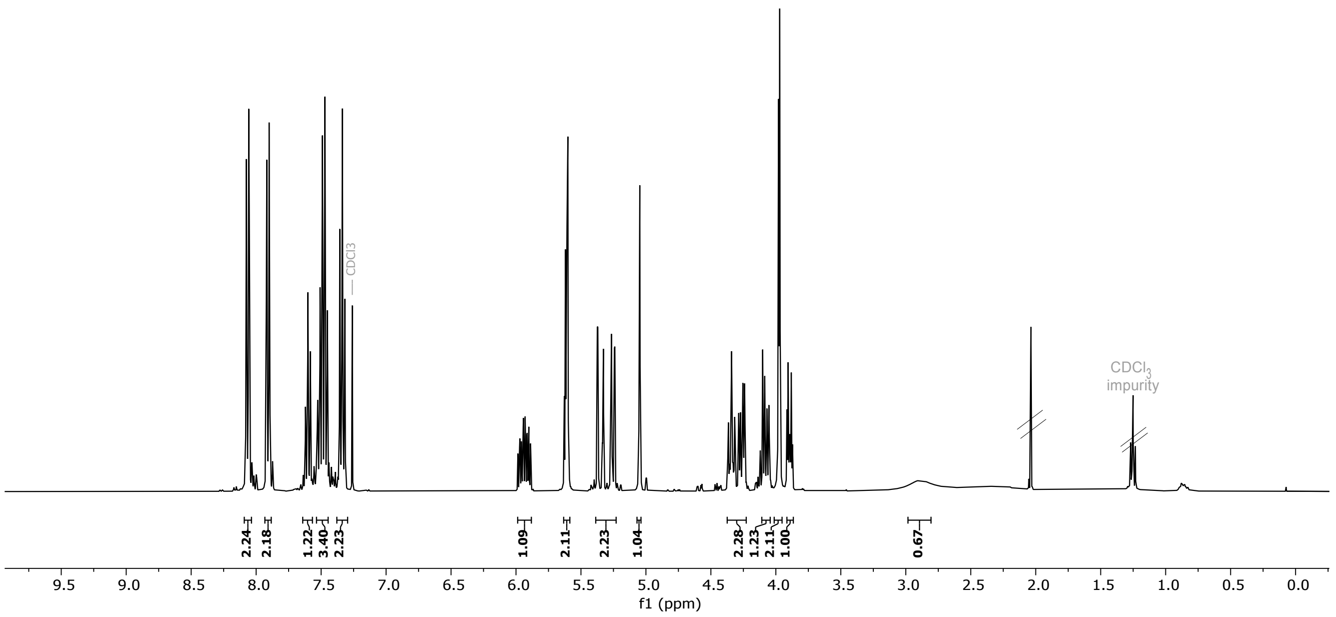

^{13}C NMR ($CDCl_3$)

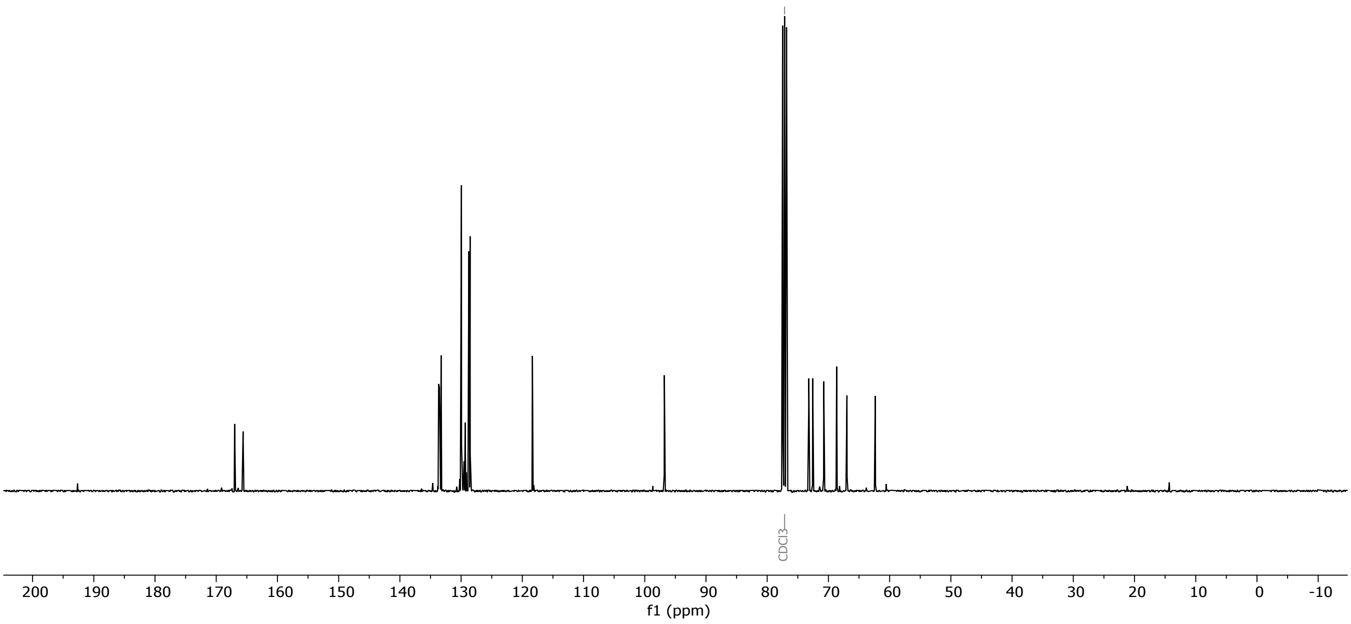

HSQC ($CDCl_3$)

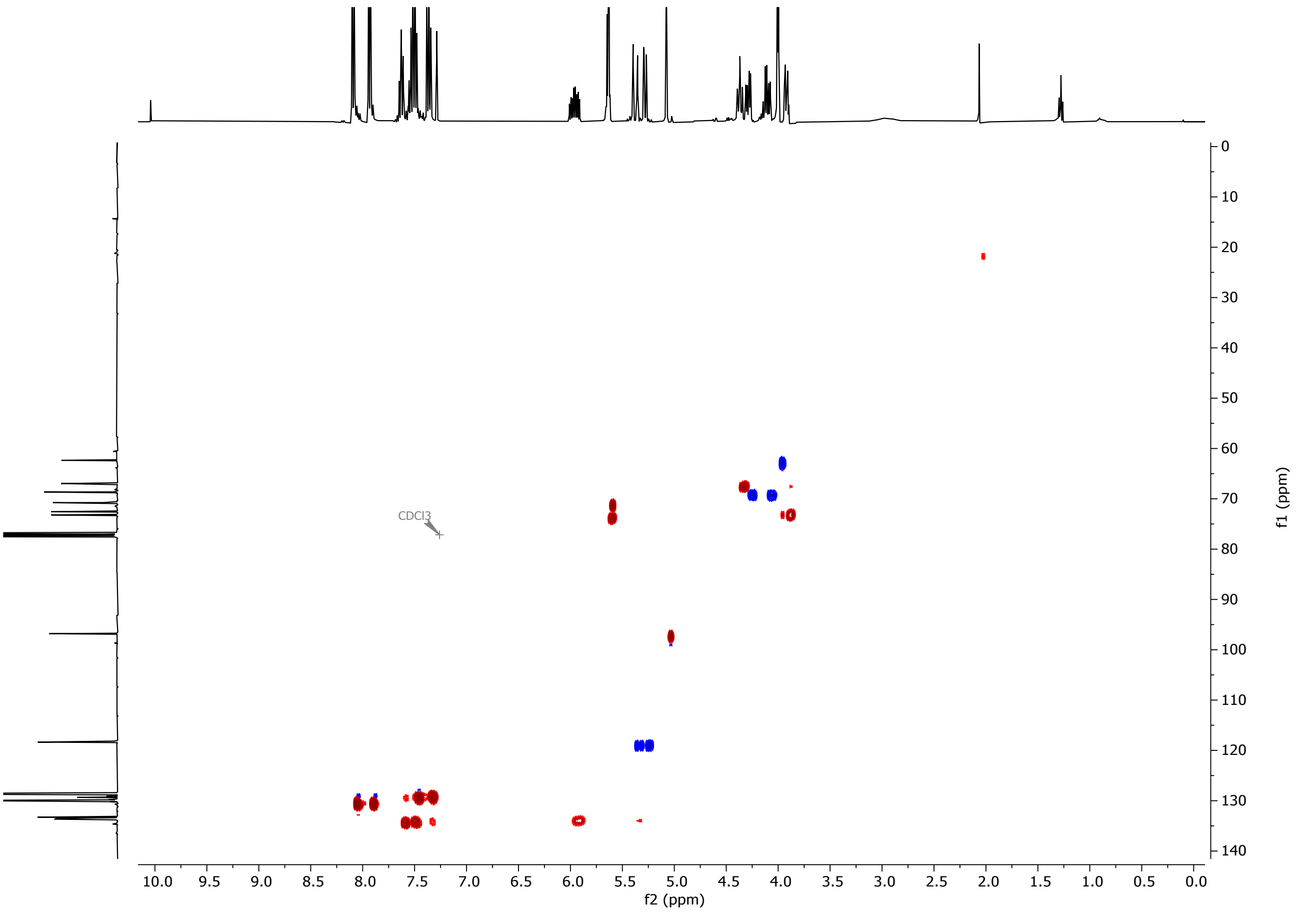

*Allyl 2,3-di-O-benzoyl-6-O-(9-fluorenylmethoxycarbonyl)-α-D-mannopyranoside (**3.4**)*

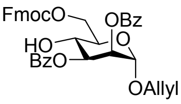

3.4

To a stirred suspension of **3.3** (4.00 g, 9.34 mmol, 1.00 equiv.) in anhydr. DCM (20 mL) and pyridine (3.77 mL, 46.7 mmol, 5.00 equiv.), in -20°C fluorenylmethyloxycarbonyl chloride (FmocCl) (2.90 g, 11.2 mmol, 1.20 equiv.) was added and the reaction mixture was stirred for 30 min. Then all the volatiles were removed under reduced pressure. The residue was purified by flash column chromatography using a mixture of hexane/ethyl acetate (4:1) as eluent. The product was obtained as a white solid in 78% yield (4.74 g, 7.28 mmol). ^{1}H NMR (400 MHz, $CDCl_3$): δ = 8.13 – 8.06 (m, 2H, *-Ar)*, 7.96 – 7.89 (m, 2H, *-Ar)*, 7.78 (m, 2H, *-Ar)*, 7.64 (m, 2H, *-Ar)*, 7.59 – 7.50 (m, 2H, *-Ar)*, 7.44 – 7.28 (m, 8H, *-Ar)*, 6.04 – 5.87 (m, 1H, CH_2=C*H*-, Allyl), 5.64 – 5.59 (m, 2H, *H*-2, *H*-3), 5.36 (dd, *J* = 17.2, 1.6 Hz, 1H, CH*H*=C*H-,* Allyl), 5.27 (dd, *J* = 10.4, 1.3 Hz, 1H, CH*H*=C*H-,* Allyl), 5.09 (d, *J* = 1.4 Hz, 1H, *H*-1), 4.69 – 4.54 (m, 2H, *H*-6a, *H*-6b), 4.48 – 4.43 (m, 2H, -CH_2-CH-, Fmoc), 4.33 – 4.23 (m, 3H, *H*-4, -OCH*H*-CH=CH_2, Allyl , -CH_2-C*H*-, Fmoc), 4.17 – 4.04 (m, 2H, -OCH*H*-CH=CH_2, Allyl, *H*-5), 2.85 (d, *J* = 4.9 Hz, 1H, -OH) ppm; ^{13}C NMR (101 MHz, $CDCl_3$): δ = 167.1, 165.5, 155.6, 143.4, 143.4, 141.4, 133.6, 133.6, 133.2, 130.0, 129.9, 129.5, 129.2, 128.7, 128.5, 128.0, 127.3, 125.3, 120.2, 118.5, 96.8, 73.1, 71.1, 70.6, 70.3, 68.8, 66.8, 66.7, 46.8 ppm; IR (neat) ν_{max}: 3528, 2983, 2928, 1725, 1601, 1386, 1281, 1256, 1154 cm^{-1}; ESI-HRMS: m/z $[M+Na]^+$ calcd. for $C_{38}H_{34}O_{10}Na$: 673.2044 found 673.2070.

^{1}H NMR ($CDCl_3$)

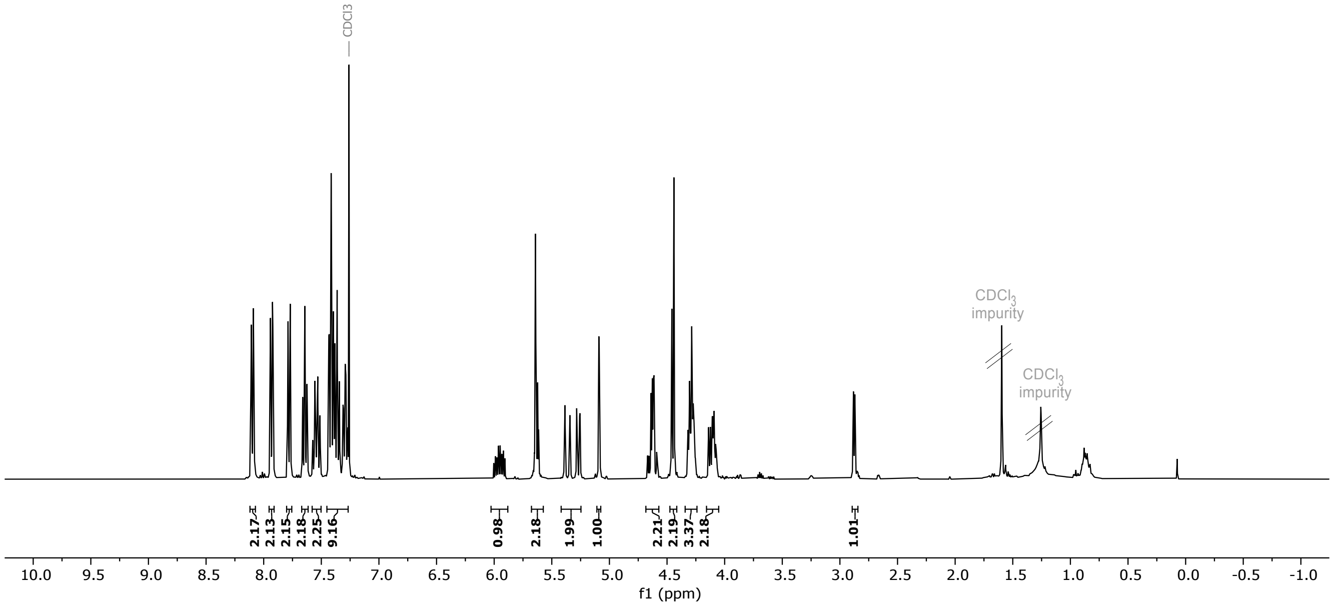

^{13}C NMR ($CDCl_3$)

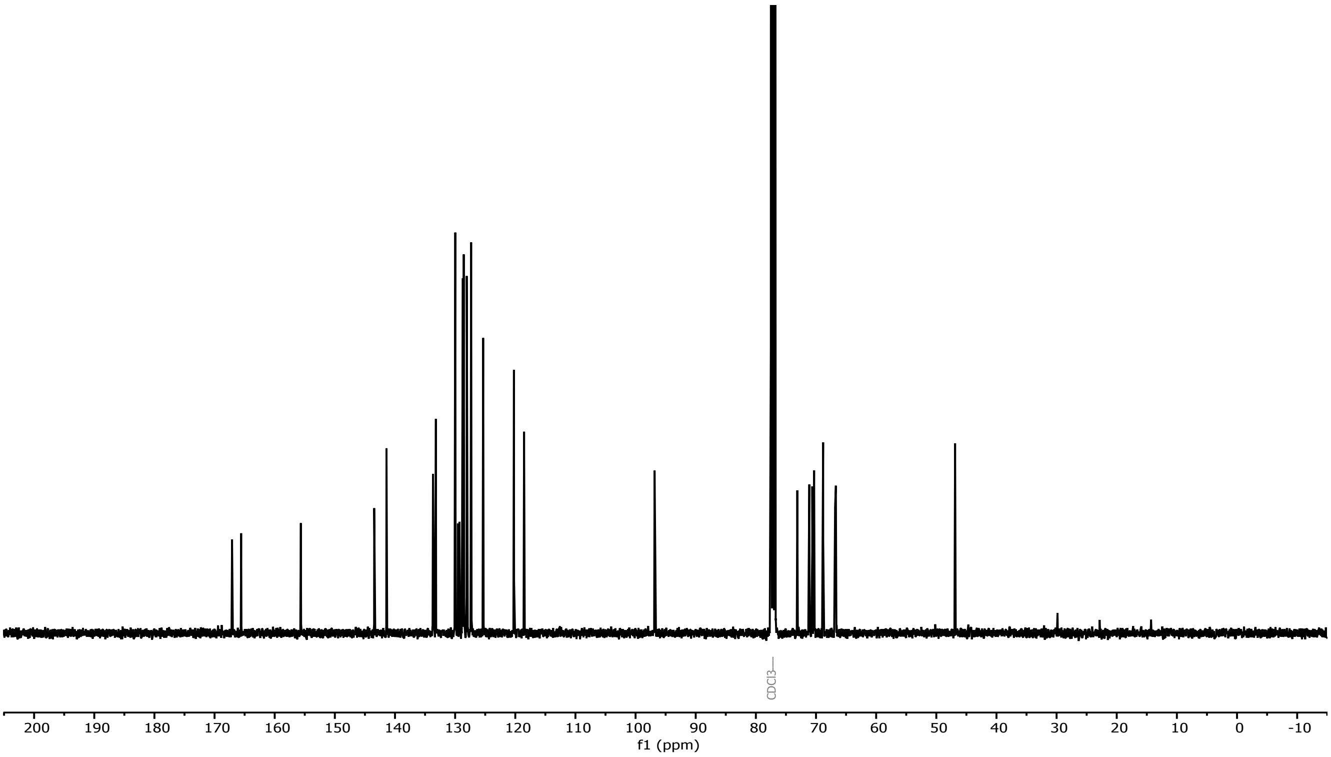

HSQC NMR ($CDCl_3$)

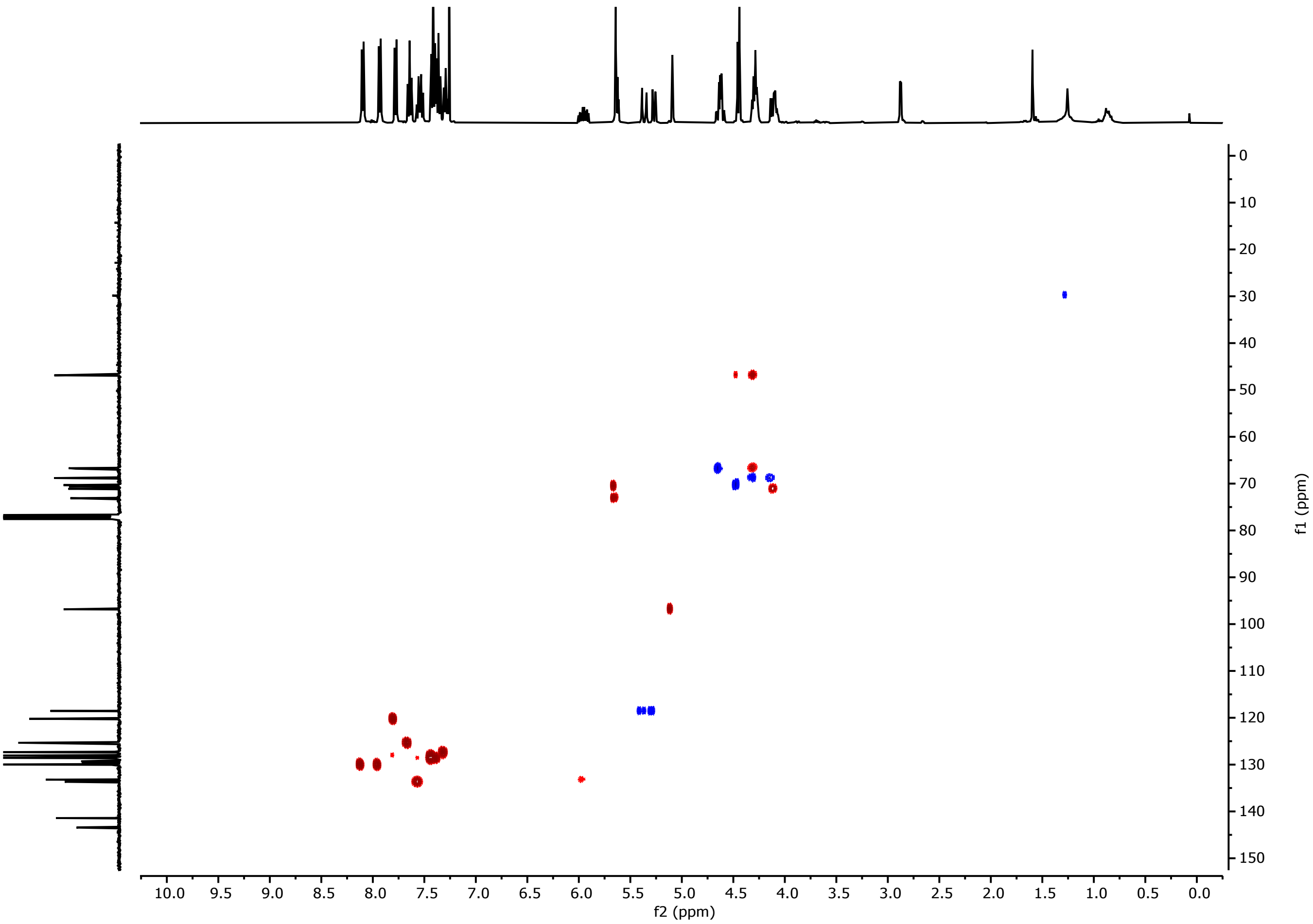

*Allyl 2,3,4-tri-O-benzoyl-6-O-(9-fluorenylmethoxycarbonyl)-α-D-mannopyranoside (**3.5**)*

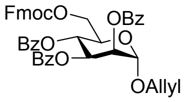

3.5

3.4 (2.30 g, 3.53 mmol, 1.00 equiv.) was dissolved in pyridine (23 mL) and the solution was cooled to 0°C. BzCl (1.40 mL, 12.2 mmol, 4.00 equiv.) was added dropwise to the solution and the mixture was left for 3 h at rt. Then, the mixture was concentrated under vacuum to remove pyridine. The residue was diluted and extracted with ethyl acetate (50 mL), washed with water (50 mL), $NaHCO_3$-solution (50 mL), and NaCl-solution. The combined organic layers were dried over Na_2SO_4 and concentrated under reduced pressure. The crude product was purified by flash column chromatography using a mixture of hexane/ethyl acetate (4:1) as eluent. The product was obtained as a white solid in 89% yield (2.38 g, 3.15 mmol). ^{1}H NMR (400 MHz, $CDCl_3$): δ = 8.15 – 8.09 (m, 2H*, -Ar),* 8.01 – 7.95 (m, 2H*, -Ar)*, 7.86 – 7.82 (m, 2H*, -Ar)*, 7.82 – 7.74 (m, 2H*, -Ar)*, 7.63 – 7.35 (m, 12H*, -Ar)*, 7.33 – 7.28 (m, 3H*, -Ar)*, 6.05 – 5.96 (m, 1H, -CH_2=C*H*-), 5.96 – 5.91 (m, 1H, *H*-3,), 5.71 (t, *J* = 2.3 Hz, 1H, *H*-2), 5.44 – 5.26 (m, 2H, C*H*$_2$=C*H*-), 5.17 (d, *J* = 1.8 Hz, 1H, *H*-1), 4.53 – 4.09 (m, 7H, *H*-4, *H*-5, *H*-6a, *H*-6b, -C*H*$_2$-CH=, -C*H*- Fmoc) ppm; ^{13}C NMR (101 MHz, $CDCl_3$): δ = 165.7, 165.6, 165.5, 155.1, 143.5, 143.3, 141.3, 141.3, 133.7, 133.6, 133.3, 133.1, 130.0, 129.9, 129.8, 129.3, 129.1, 129.0, 128.7, 128.6, 128.4, 128.0, 128.0, 127.3, 127.3, 125.4, 125.3, 120.1, 120.1, 118.7, 96.7, 70.5, 70.3, 70.0, 69.1, 68.9, 67.2, 66.5, 46.7 ppm; IR (neat) ν_{max}: 3068, 2919, 1729, 1452, 1259, 1108, 1070, 1027 cm^{-1}; ESI-HRMS: m/z [M+Na]$^+$ calcd. for $C_{45}H_{38}O_{11}$: 777.2306 found 777.2349.

^{1}H NMR ($CDCl_3$)

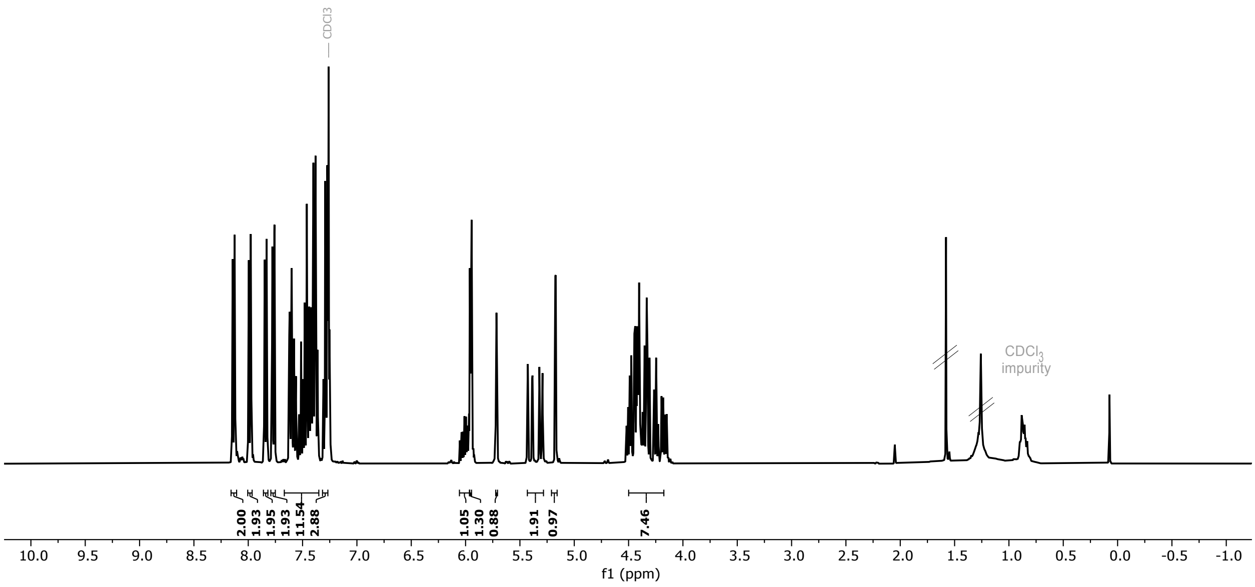

^{13}C NMR ($CDCl_3$)

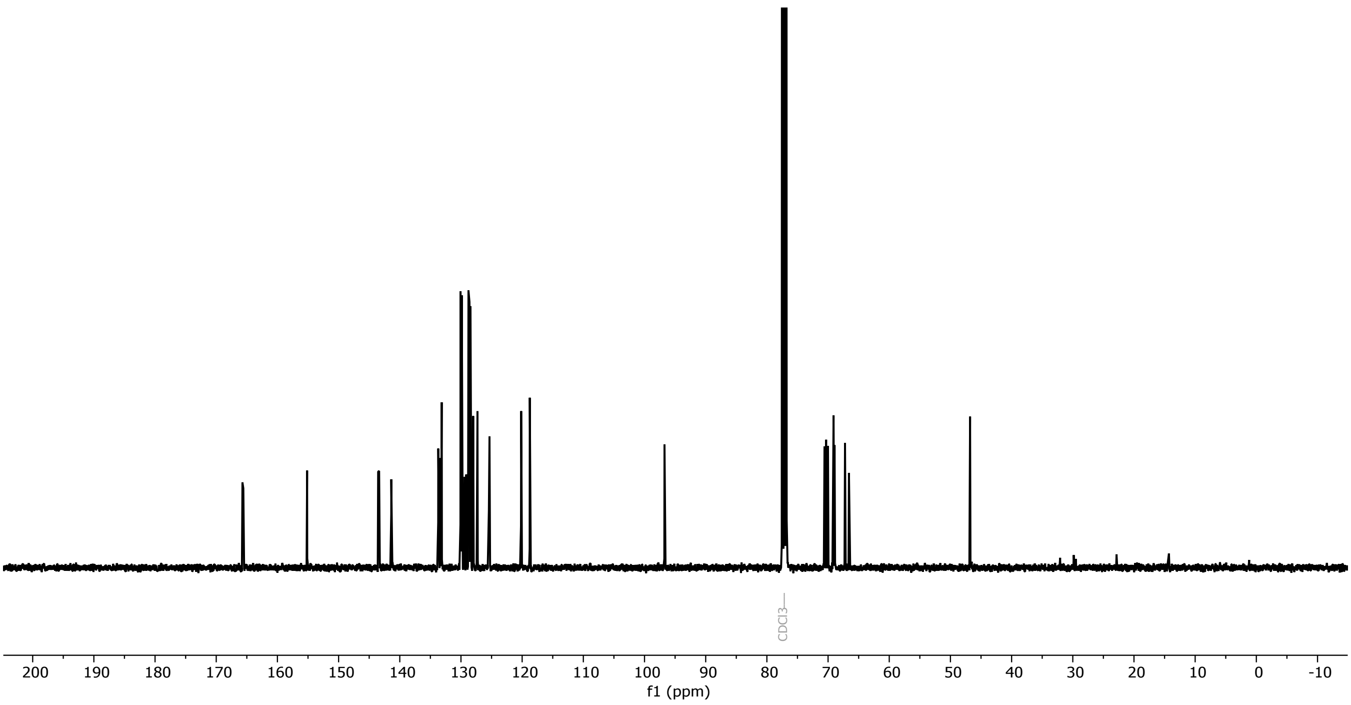

HSQC ($CDCl_3$)

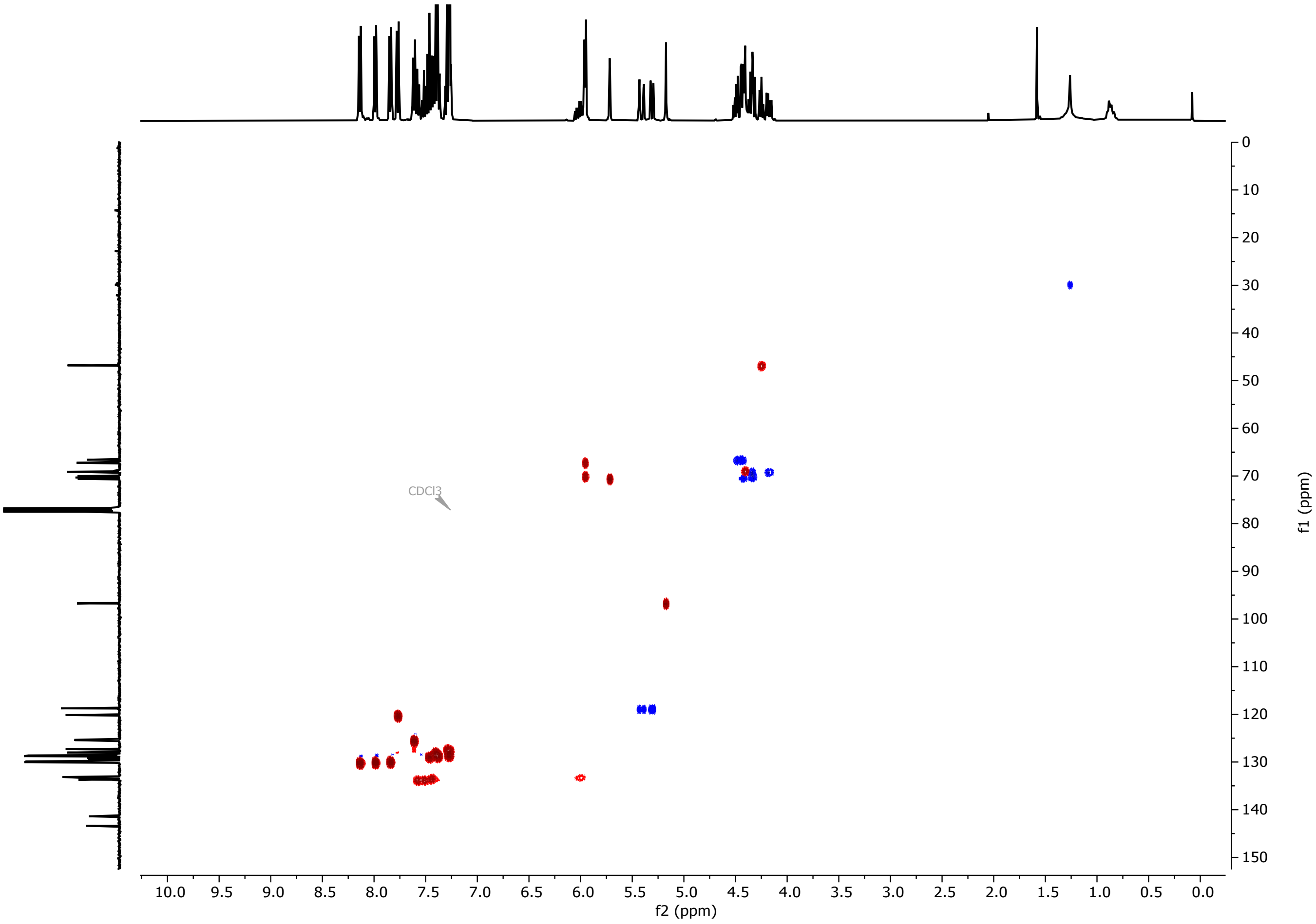

*2,3,4-tri-O-benzoyl-6-O-(9-fluorenylmethoxycarbonyl)-α-D-mannopyranoside (**3.6**)*

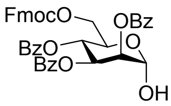

3.6

The mixture of **3.5** (2.22 g, 2.95 mmol, 1.00 equiv.) and $PdCl_2$ (260 mg, 1.47 mmol, 0.50 equiv.) in MeOH/DCM (1:1, 14 mL) was stirred for 4 h at rt. The reaction mixture was filtered through Celite® and concentrated under vacuum. The residue was purified by flash column chromatography using a mixture of hexane/ethyl acetate (2:1) as eluent. The product was obtained as a white solid in 98% yield (2.08 g, 2.91 mmol). ^{1}H NMR (400 MHz, $CDCl_3$): δ = 8.16 – 8.09 (m, 2H*, -Ar)*, 8.01 – 7.94 (m, 2H*, -Ar)*, 7.86 – 7.82 (m, 2H*, -Ar)*, 7.77 (d, *J* = 7.6 Hz, 2H*, -Ar)*, 7.64 – 7.37 (m, 12H*, -Ar)*, 7.32 – 7.27 (m, 3H*, -Ar)*, 6.00 (dd, *J* = 10.0, 3.1 Hz, 1H, *H*-3), 5.98 – 5.91 (m, 1H, *H*-4), 5.73 (dd, *J* = 3.1, 1.9 Hz, 1H, *H*-2), 5.55 (dd, *J* = 4.1, 1.9 Hz, 1H, *H*-1,), 4.62 (dt, *J* = 9.1, 4.3 Hz, 1H, *H*-5), 4.51 – 4.39 (m, 3H, *H*-6a, -CH_2-CH- Fmoc), 4.35 (dd, *J* = 10.4, 7.4 Hz, 1H, *H*-6b), 4.24 (t, *J* = 7.4 Hz, 1H, -C*H*- Fmoc), 2.11 (d, *J* = 4.1 Hz, 1H, -O*H*) ppm; ^{13}C NMR (101 MHz, $CDCl_3$): δ = 165.7, 165.6, 165.6, 155.1, 143.5, 143.3, 141.3, 133.7,133.6, 133.3, 130.0, 130.0, 129.9, 129.9, 129.8, 129.3, 129.1, 129.0, 128.9, 128.9, 128.7, 128.6, 128.4, 128.0, 128.0, 127.3, 127.3, 125.4, 125.3, 120.1, 120.1, 70.7, 70.2, 69.5, 69.0, 68.9, 67.1, 66.6, 46.8 ppm; IR (neat) ν_{max}: 3438, 2982, 1730, 1603, 1452, 1263, 1109, 1070, 1027 cm^{-1}; ESI-HRMS: m/z [M+Na]$^+$ calcd. for $C_{42}H_{34}O_{11}$ Na: 737.1993 found 737.2000.

^{1}H NMR ($CDCl_3$)

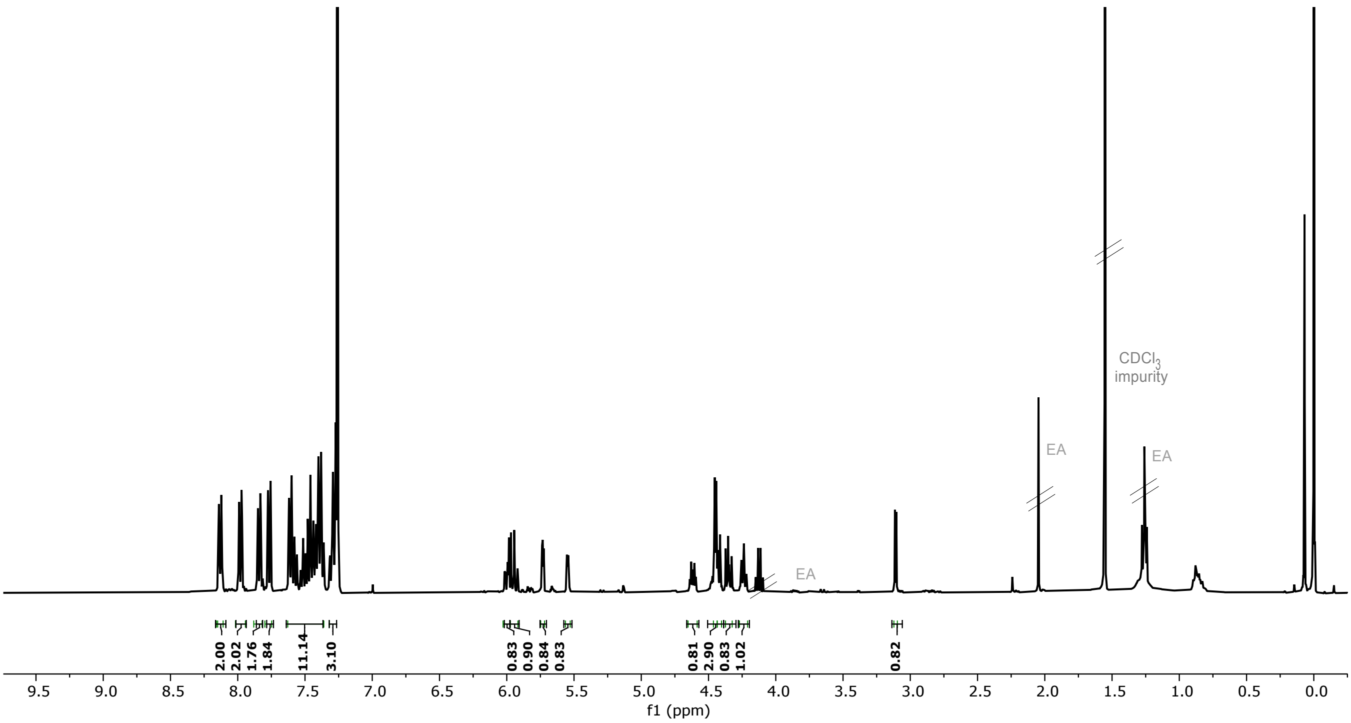

^{13}C NMR ($CDCl_3$)

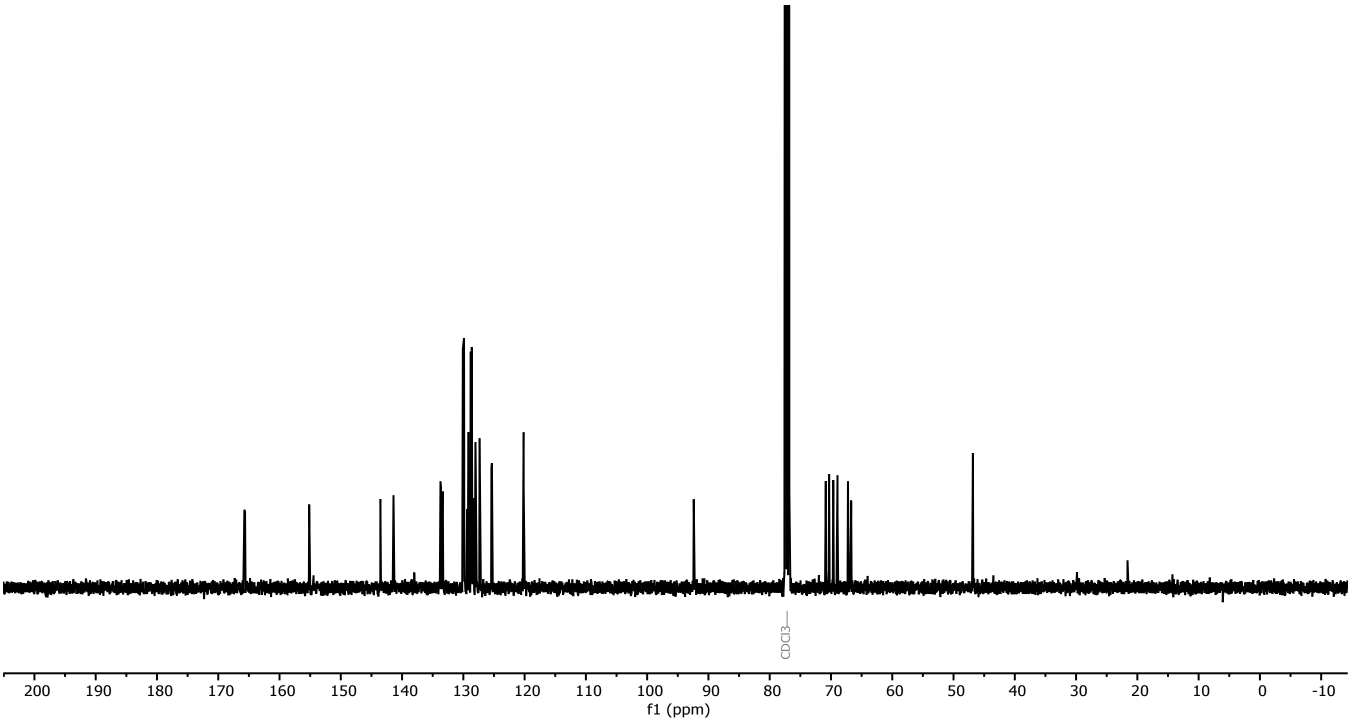

HSQC ($CDCl_3$)

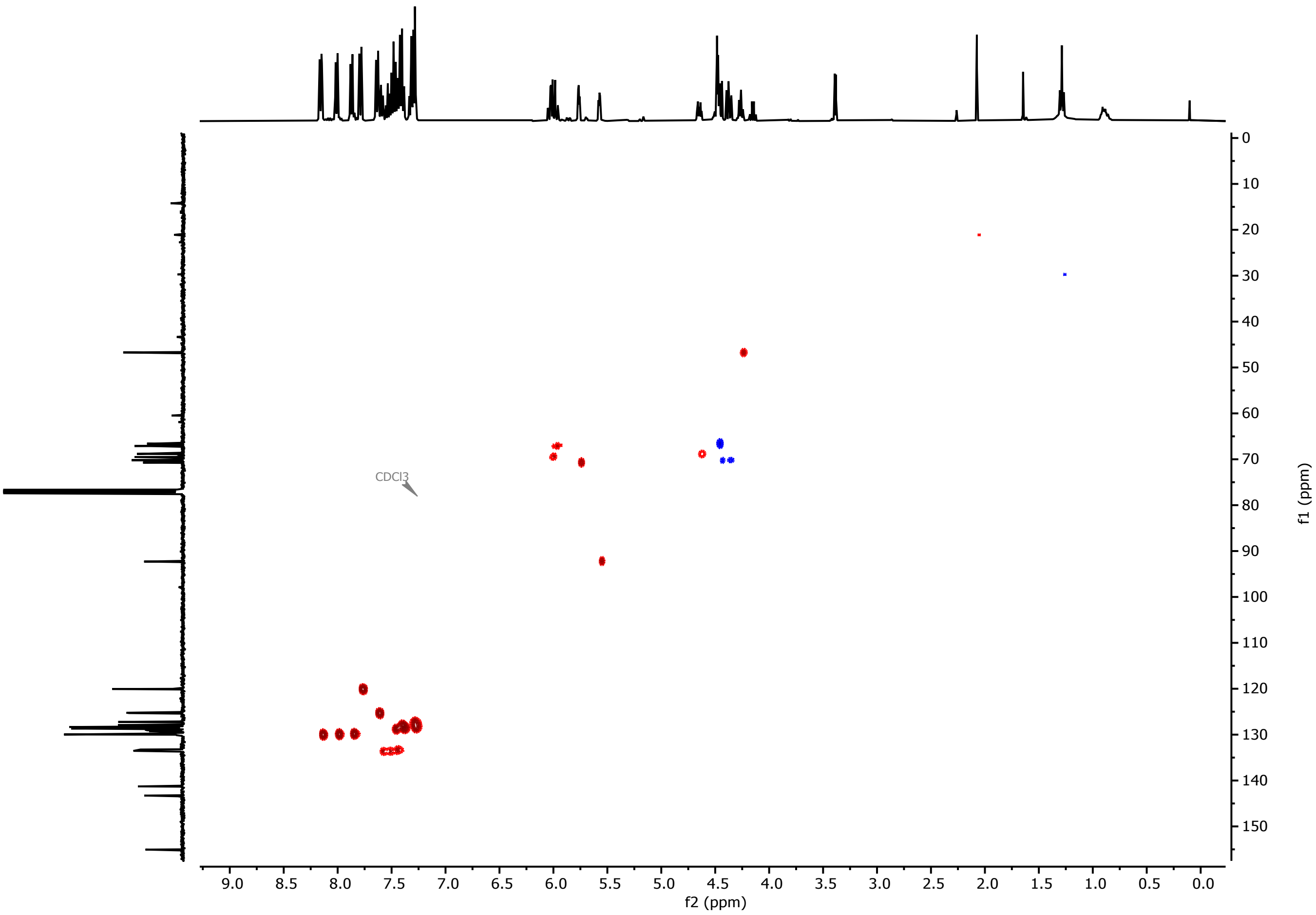

*2,3,4-O-tri-benzoyl-6-O-(9-fluorenylmethoxycarbonyl)-α-D-mannopyranosyl trichloroacetimidate (**3**)*

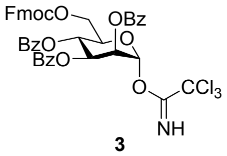

3

To a stirred solution of **3.6** (1.00 g, 1.40 mmol, 1.00 equiv.) and trichloroacetonitrile (2.81 mL, 28.0 mmol, 20.00 equiv.) in anhydr. DCM (13 mL), NaH (60% dispersion in oil, 16.8 mg, 0.70 mmol, 0.50 equiv.) was added at rt under argon atmosphere and the reaction left to react overnight. Upon completion, solvent (in presence of silica to quench the remaining NaH) was evaporated and the residue was purified by flash column chromatography using a mixture of hexane/ethyl acetate (3:1) to afford the desired compound as a white solid in 97% yield (1.17 g, 1.37 mmol). ^{1}H NMR (400 MHz, $CDCl_3$): δ = 8.87 (s, 1H, =N*H*), 8.17 – 8.12 (m, 2H, -*Ar*), 8.01 – 7.96 (m, 2H, -*Ar*), 7.86 – 7.80 (m, 2H, -*Ar*), 7.79 – 7.74 (m, 2H, -*Ar*), 7.63 – 7.36 (m, 11H, -*Ar*), 7.30 – 7.27 (m, 4H, -*Ar*), 6.59 (d, *J* = 2.0 Hz, 1H, *H*-1), 6.09 (t, *J* = 10.0 Hz, 1H, *H*-4), 5.97 (dd, *J* = 10.0, 3.3 Hz, 1H, *H*-3), 5.93 (dd, *J* = 3.3, 2.0 Hz, 1H, *H*-2), 4.58 (dt, *J* = 10.0, 3.9 Hz, 1H, *H*-5), 4.51 – 4.39 (m, 3H, *H*-6a, -CH_2-CH-, Fmoc), 4.35 (dd, *J* = 10.4, 7.4 Hz, 1H, *H*-6b), 4.24 (t, *J* = 7.4 Hz, 1H, -CH_2-C*H*-, Fmoc) ppm; ^{13}C NMR (101 MHz, $CDCl_3$): δ = 165.5, 165.3, 159.9, 155.0, 143.5, 143.3, 141.3, 141.3, 133.9, 133.8, 133.5, 130.1, 130.0, 129.8, 128.9, 128.8, 128.8, 128.8, 128.7, 128.6, 128.6, 128.5, 128.0, 128.0, 127.3, 127.3, 125.5, 125.3, 120.1, 120.1, 94.5, 90.6, 71.4, 70.4, 69.7, 68.8, 66.3, 66.0, 46.7 ppm; IR (neat) ν_{max}: 3372, 2256, 1733, 1694, 1603, 1452, 1260, 1093, 1070 cm^{-1}; ESI-HRMS: m/z $[M+Na]^+$ calcd. for $C_{44}H_{34}Cl_3NO_{11}Na$: 880.1090 found 880.1144.

^{1}H NMR ($CDCl_3$)

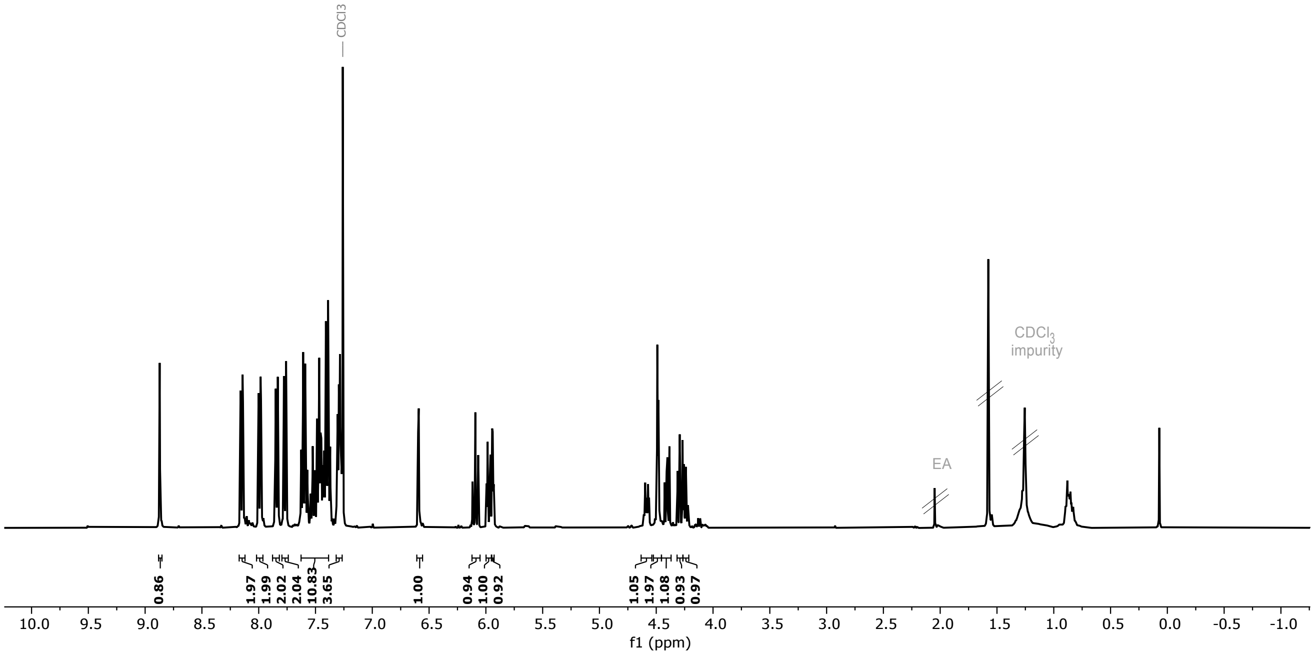

^{13}C NMR ($CDCl_3$)

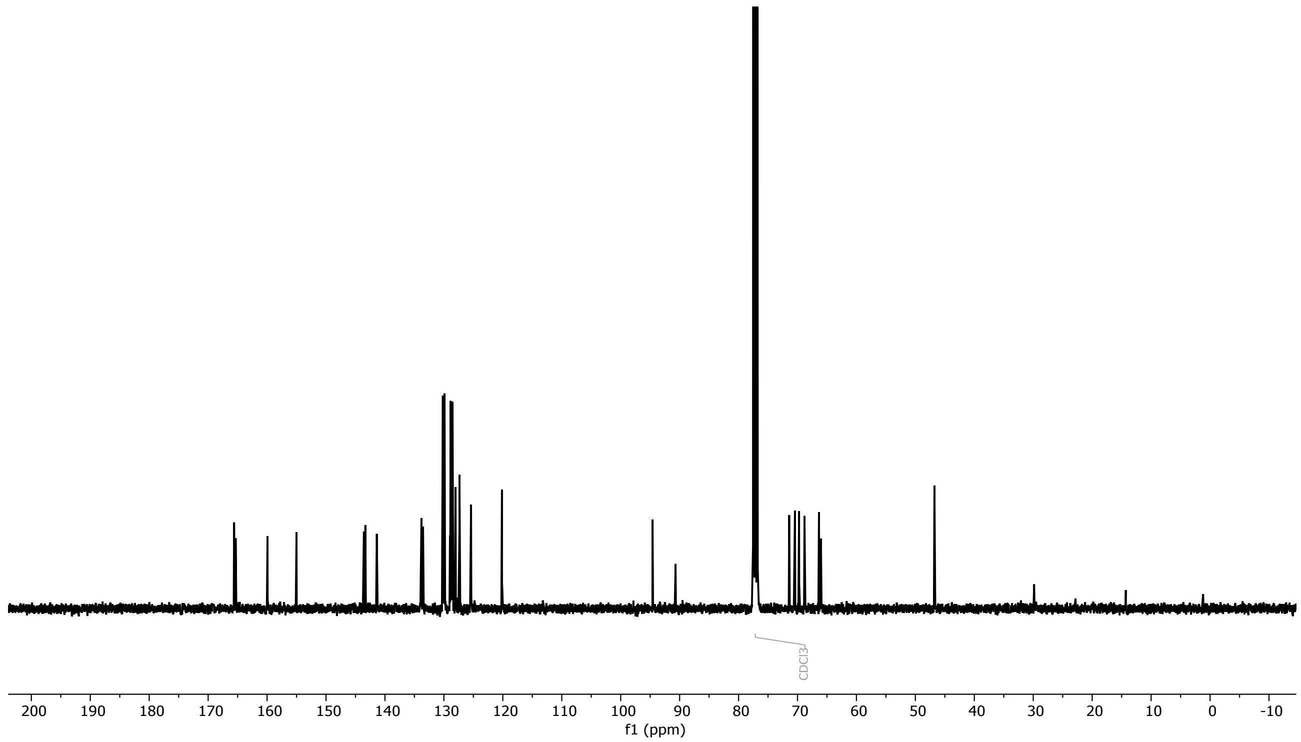

HSQC ($CDCl_3$)

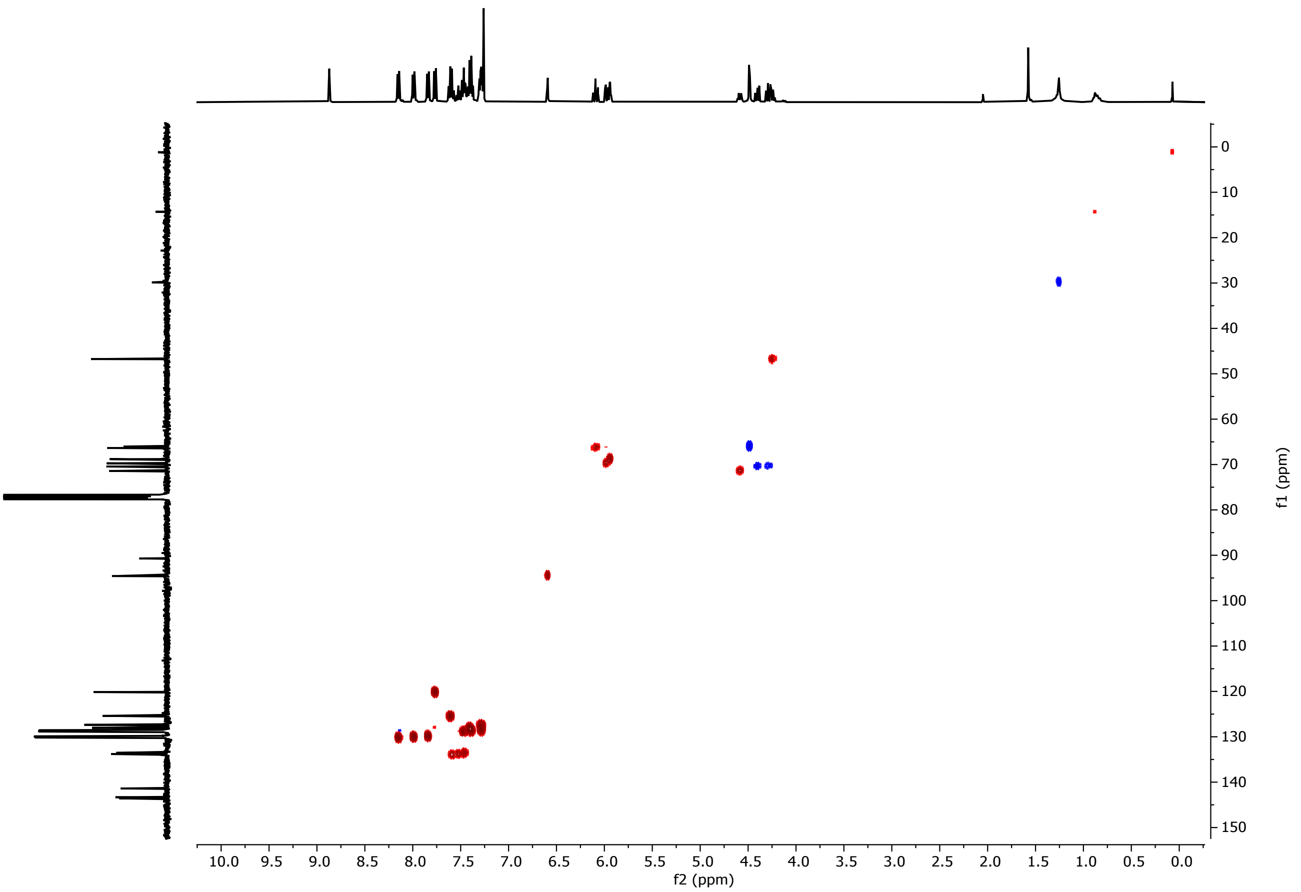

Synthesis of 2-*O*-benzoyl-3,4-di-*O*-benzyl-6-*O*-(9-fluorenylmethoxycarbonyl)-α-D-mannopyranosyl trichloroacetimi-date (5)

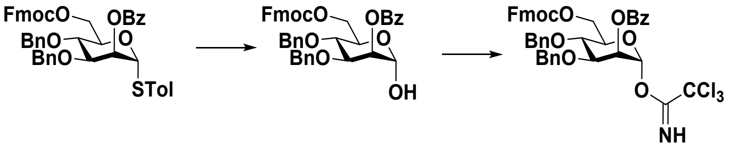

5.1 **5.2** **5**

*2-O-benzoyl-3,4-di-O-benzyl-6-O-(9-fluorenylmethoxycarbonyl)-α-D-mannopyranoside (**5.2**)*

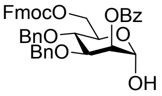

5.2

N-Bromosuccinimide (NBS) (1.60 g, 8.99 mmol, 2.00 equiv.) was added to a stirred solution of **5.1** (4.41 g, 16.1 mmol, 1.00 equiv.) in acetone/water (9:1, 40 mL). The reaction was stirred overnight at rt. Additional amount of NBS (2.00 g, 11.2 mmol, 2.50 equiv.) was added. After 1 h, the reaction mixture was diluted with water (50 mL) and extracted with DCM (50 mL) three times. The combined organic phase was washed with $NaHCO_3$-solution (100 mL), dried over Na_2SO_4 and concentrated under reduced pressure. The crude compound was purified by flash column chromatography using a mixture of hexane/ethyl acetate (3:1) to afford the desired compound as a white foam in 88% yield (2.68 g, 3.90 mmol). ^{1}H NMR (400 MHz, $CDCl_3$): δ = 8.13 – 7.27 (m, 23H, -*Ar*), 5.67 (dd, *J* = 3.1, 2.0 Hz, 1H, *H*-2), 5.38 (dd, *J* = 3.9, 1.9 Hz, 1H, *H*-1), 4.94 (d, *J* = 10.9 Hz, 1H, -C*H*H-, Bn), 4.82 (d, *J* = 11.3 Hz, 1H, -C*H*H-, Bn), 4.65 – 4.56 (m, 2H, -C*H*H-, Bn, -C*H*H, –Bn,), 4.52 (dd, *J* = 11.5, 2.2 Hz, 1H, *H*-6a), 4.45 – 4.36 (m, 3H, -CH_2-CH- Fmoc, *H*-6b), 4.26 (t, *J* = 7.4 Hz, 1H, -C*H*-, Fmoc), 4.20 (ddt, *J* = 10.3, 7.1, 2.6 Hz, 2H, *H*-3, *H*-4), 3.95 (t, *J* = 9.6 Hz, 1H, *H*-5), 3.14 (brs, 1H, -O*H*) ppm; ^{13}C NMR (101 MHz, $CDCl_3$): δ = 165.6, 155.2, 143.4, 143.3, 141.2, 137.9, 137.7, 133.3, 129.9, 129.7, 128.5, 128.4, 128.4, 128.2, 128.1, 127.9, 127.8, 127.7, 127.2, 125.2, 125.2, 120.1, 92.6, 75.2, 73.8, 71.5, 70.0, 69.9, 68.9, 67.0, 46.7 ppm; IR (neat) ν_{max}: 2932, 1750, 1726, 1452, 1257, 1099 cm^{-1}; ESI-HRMS: m/z [M+Na]$^+$ calcd. for $C_{42}H_{38}O_9Na$: 709.2407 found 709.2425 .

^1H NMR ($CDCl_3$)

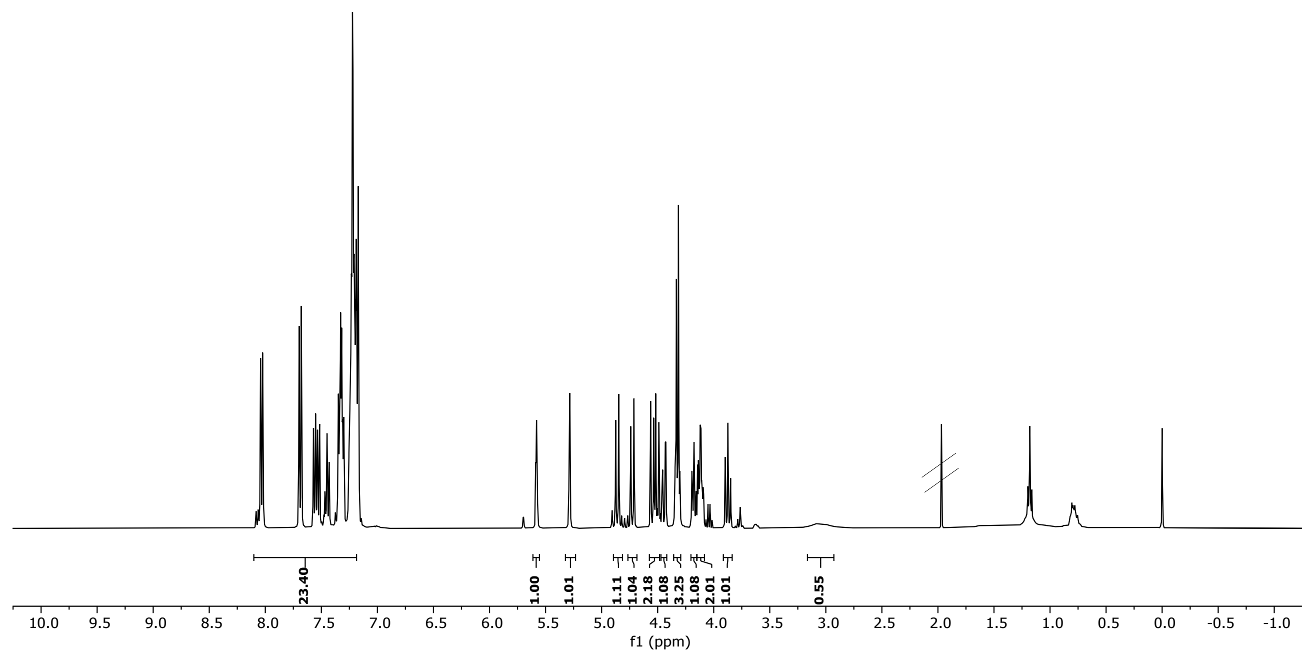

^{13}C NMR ($CDCl_3$)

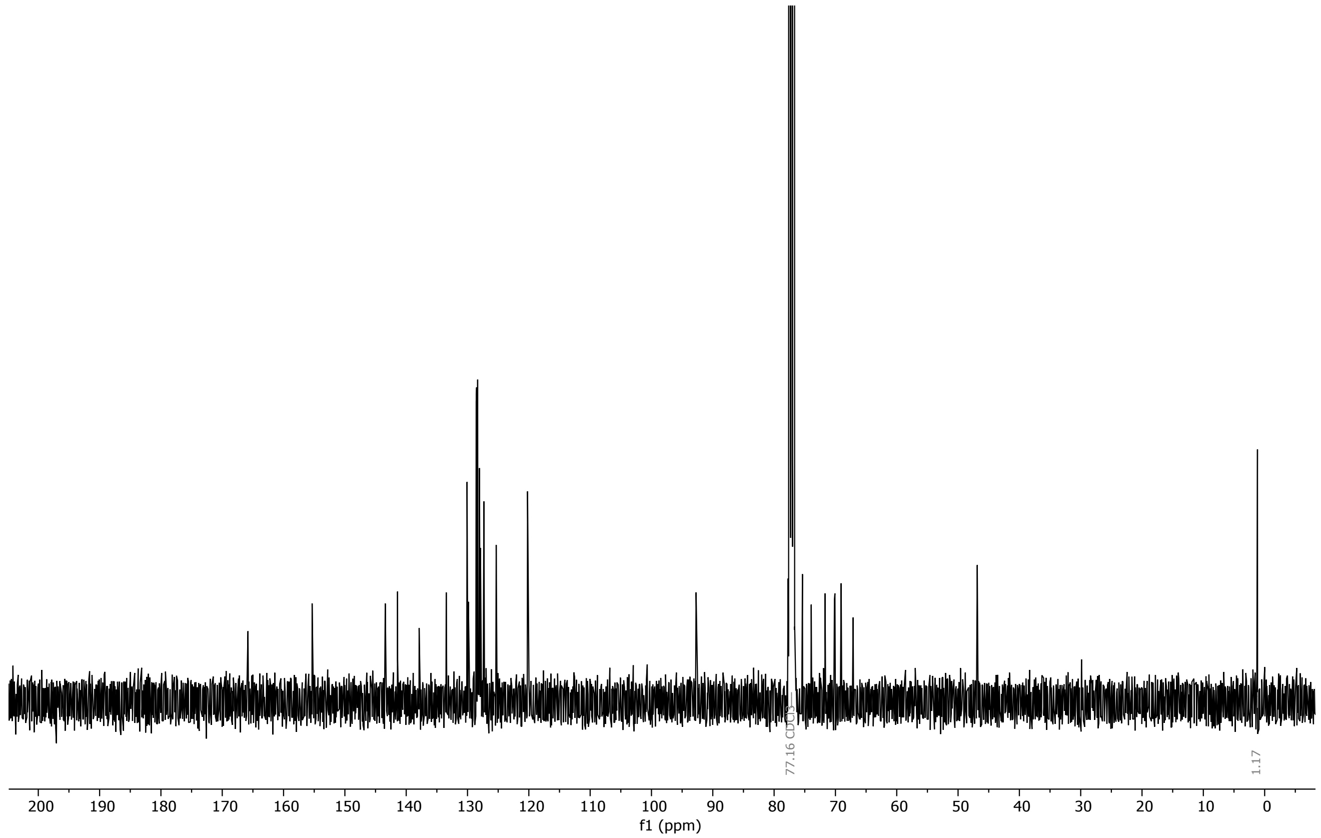

HSQC ($CDCl_3$)

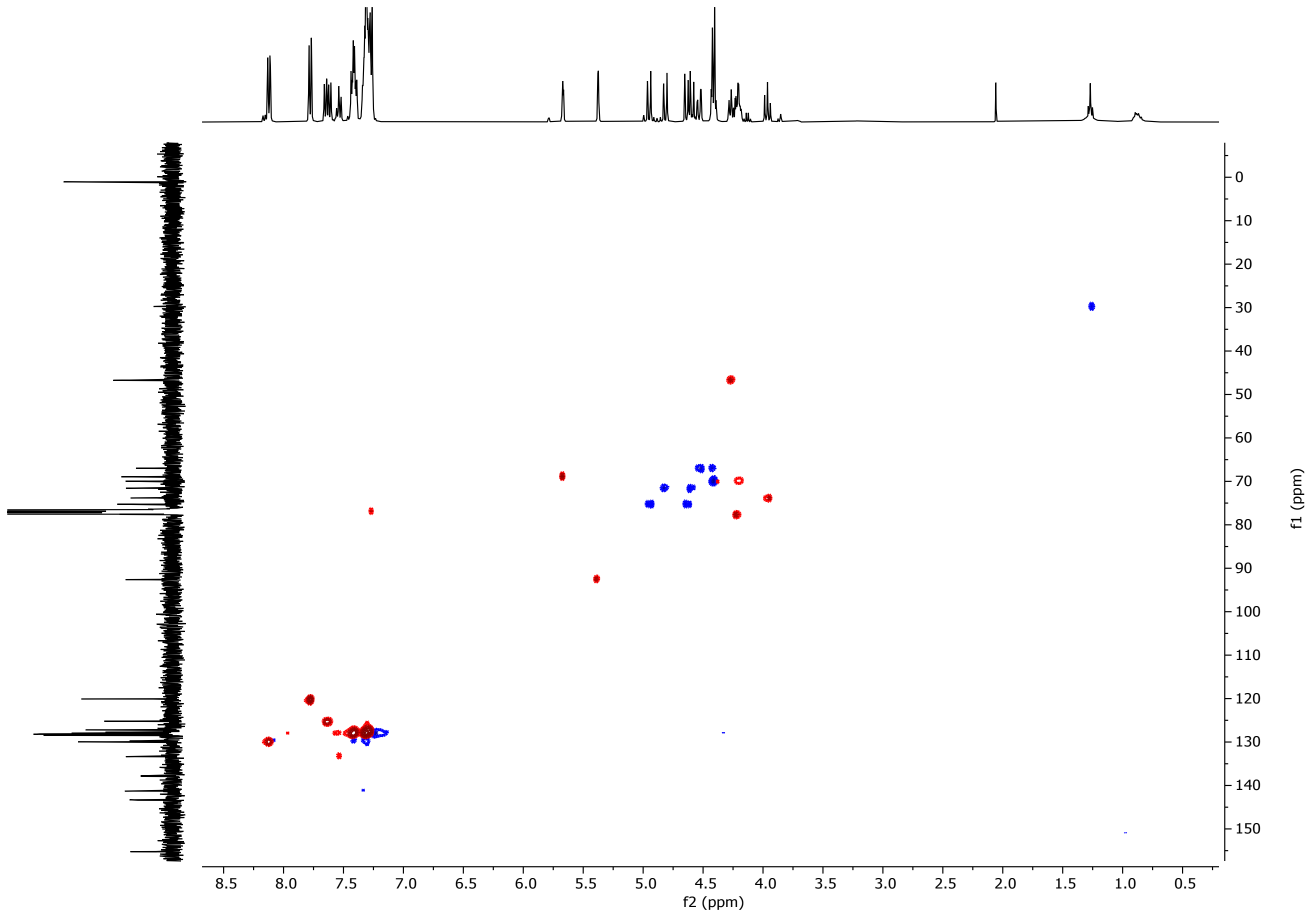

*2-O-benzoyl-3,4-di-O-benzyl-6-O-(9-fluorenylmethoxycarbonyl)-α-D-mannopyranosyl trichloroacetimidate (**5**)*

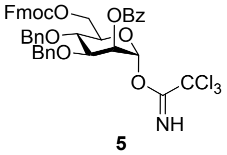

5

To a stirred solution of **5.2** (2.68 g, 3.90 mmol, 1.00 equiv.) in DCM (40 mL), trichloroacetonitrile (3.91 mL, 39.0 mmol, 10.00 equiv.) was added and NaH (60% dispersion in oil) (47.0 mg, 1.20 mmol, 0.30 equiv.) were added. After 4 h, additional amount of NaH (10.0 mg, 0.25 mmol, 0.06 equiv.) was added. Upon completion, the solvent was evaporated (in presence of silica to quench the remaining NaH) and the residue was purified by flash column chromatography using a mixture of hexane/ethyl acetate (3:1) to afford the desired compound as a white foam in 81% yield (2.64 g, 3.18 mmol). ^{1}H NMR (400 MHz, $CDCl_3$): δ = 8.73 (s, 1H, = N*H*), 8.21 – 7.20 (m, 23H, *-Ar)*, 6.41 (d, *J* = 2.1 Hz, 1H, *H*-1), 5.78 (t, *J* = 2.2 Hz, 1H, *H*-2), 4.95 (d, *J* = 10.8 Hz, 1H, -C*H*H-, Bn), 4.85 (d, *J* = 11.3 Hz, 1H, -C*H*H- Bn), 4.66 (d, *J* = 3.4 Hz, 1H, -C*H*H-, Bn), 4.63 (d, *J* = 4.0 Hz, 1H, -C*H*H-, Bn), 4.54 – 4.41 (m, 2H, *H*-6a, *H*-6b), 4.38 (dd, *J* = 7.7, 1.5 Hz, 2H, -CH_2-CH-, Fmoc), 4.26 (t, *J* = 7.5 Hz, 1H, -C*H*- Fmoc), 4.19 (m, 1H, *H*-3), 4.12 (m, 2H, *H*-4, *H*-5) ppm; ^{13}C NMR (101 MHz, $CDCl_3$): δ = 165.4, 160.0, 155.1, 155.0, 143.5, 143.4, 141.3, 137.7, 137.4, 133.6, 130.1, 129.5, 128.8, 128.7, 128.6, 128.6, 128.6, 128.5, 128.1, 128.0, 127.3, 127.3, 125.3, 125.3, 120.2, 120.2, 120.1, 95.1, 95.1, 90.7, 75.6, 73.1, 73.0, 72.5, 72.4, 71.9, 70.2, 67.4, 66.2, 46.8 ppm; IR (neat) ν_{max}: 3333, 2930, 1729, 1676, 1452, 3334, 3033, 1750, 1729, 1677, 1452, 1261, 1164, 1094 cm^{-1}; ESI-HRMS: m/z [M+Na]$^{+}$ calcd. for $C_{44}H_{38}Cl_3NO_9Na$: 852.1504, found 852.1543.

^{1}H NMR ($CDCl_3$)

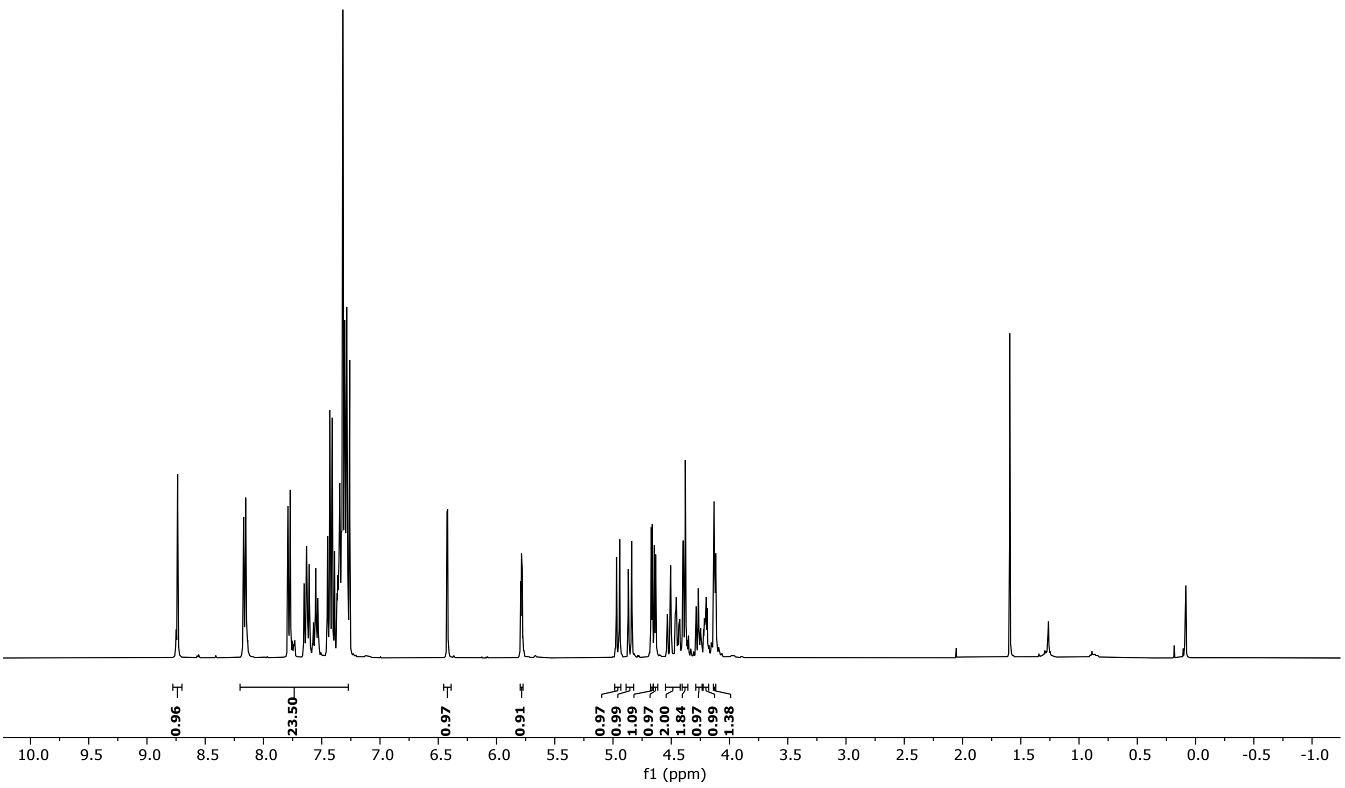

^{13}C NMR ($CDCl_3$)

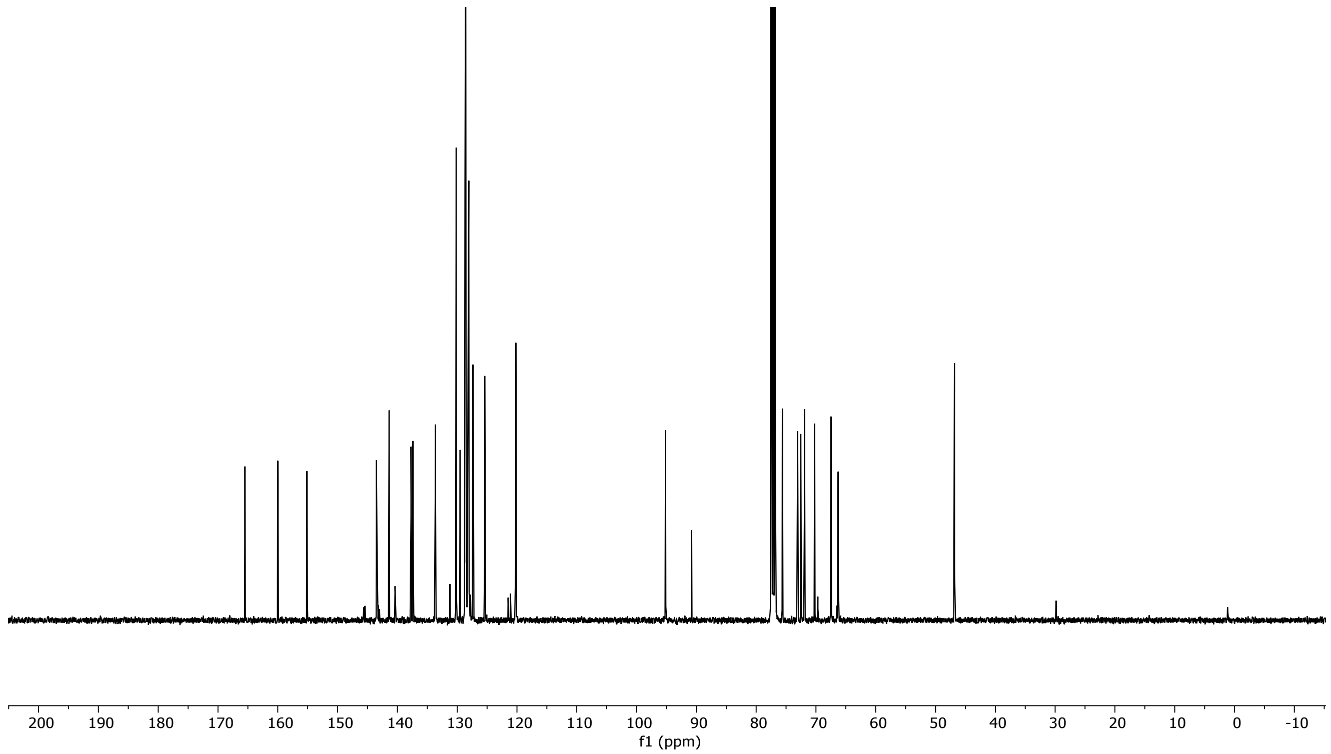

HSQC ($CDCl_3$)

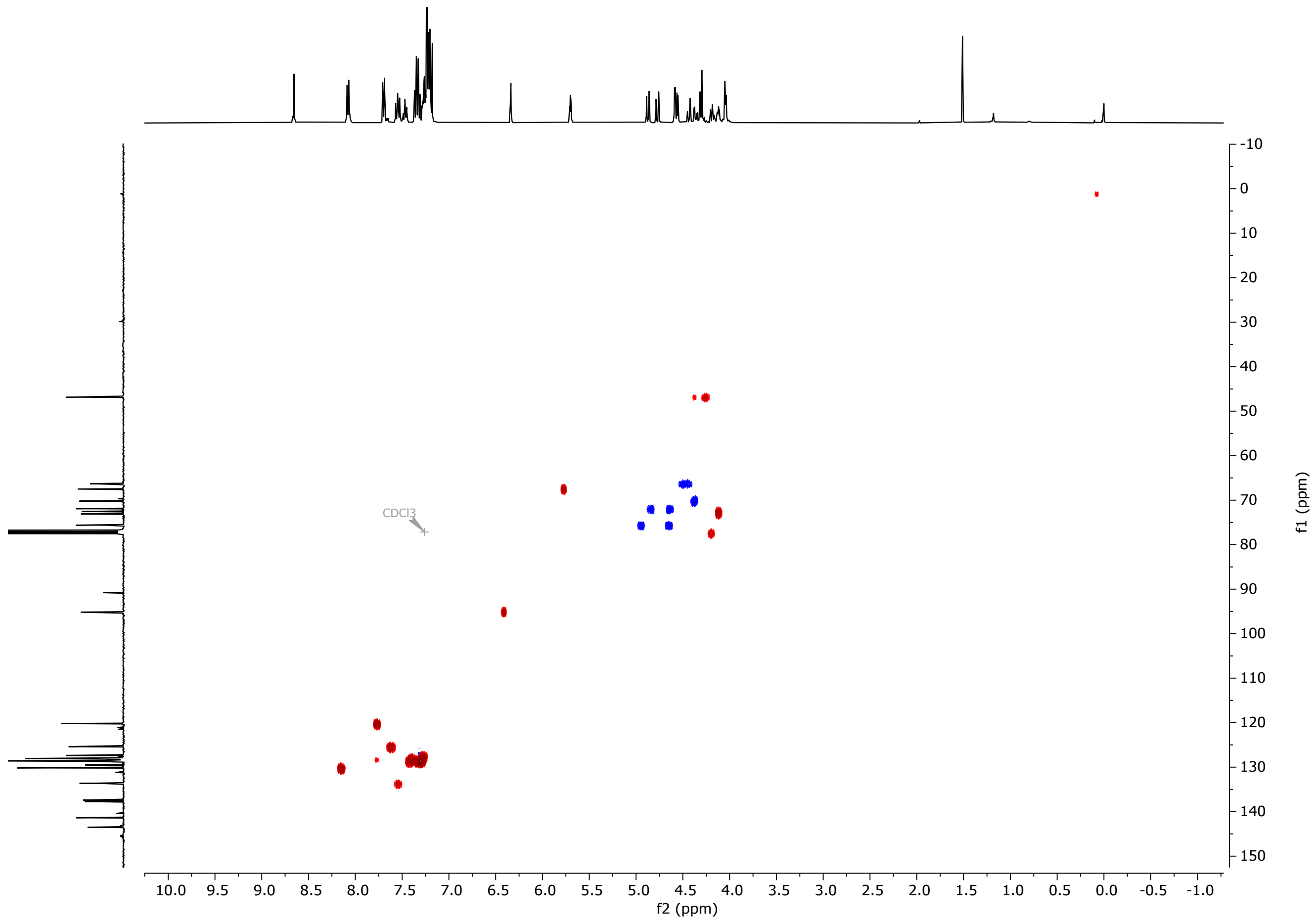

Synthesis of dibutoxyphosphoryloxy 2-*O*-benzoyl-3,4-di-*O*-benzyl-6-*O*-(9-fluorenylmethoxycarbonyl)-α-D-mannopyranosyl phosphate (6)

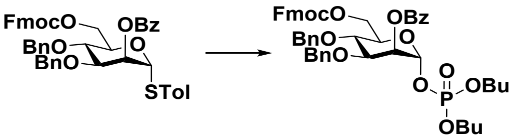

5.1 **6**

A solution of dibutyl phosphate (2.50 mL, 12.6 mmol, 16.80 equiv.) in DCM (5 mL) was dried over molecular sieves. After 1 h the supernatant (0.90 mL) was added to a solution of thioglycoside **5.1** (598 mg, 0.75 mmol, 1.00 equiv.) in DCM (5 mL) and cooled to 0°C. Then, *N*-iodosuccinimide (NIS) (204 mg, 0.91 mmol, 1.20 equiv.) and triflic acid (TfOH) (20 µL, 0.23 mmol, 0.30 equiv.) were added. The reaction was stirred for 2 h, and quenched with an aqueous $Na_2S_2O_3$/$NaHCO_3$ solution (1:1, 100 mL), and extracted with DCM (100 mL). The organic layer was dried over Na_2SO_4 and the crude product was purified by flash column chromatography using a mixture of hexane/ethyl acetate (3:1) as eluent. The product was obtained as a yellow oil in 82% yield (544 mg, 0.62 mmol). ^1H NMR (400 MHz, $CDCl_3$): δ = δ 8.15 – 7.21 (m,, 23H -*Ar*), 5.78 (dd, *J* = 6.5, 2.1 Hz, 1H, *H*-1), 5.71 (t, *J* = 2.6 Hz, 1H, *H*-2), 4.93 (d, *J* = 10.8 Hz, 1H, -C*H*H- Bn), 4.83 (d, *J* = 11.2 Hz, 1H, -C*H*H- Bn), 4.61 (dd, *J* = 11.1, 8.5 Hz, 2H, 2 -C*H*H-, Bn), 4.43 – 4.34 (m, 2H, *H*-6a, *H*-6b), 4.43 – 4.36 (m, 2H, -CH_2-CH- Fmoc), 4.26 (t, *J* = 7.4 Hz, 1H, -C*H*-, Fmoc), 4.20 – 3.99 (m, 6H, *H*-3, *H*-4, 2-OCH_2-CH$_2$-, Bu), 1.84 – 1.58 (m, 4H, 2-CH_2-CH$_3$, Bu), 1.49 – 1.33 (m, 1H, 4H, 2-CH_2-CH$_3$, Bu), 0.93 (q, *J* = 7.3 Hz, 6H, 2-CH$_3$ Bu) ppm; ^{13}C NMR (101 MHz, $CDCl_3$): δ = 165.1, 155.0, 143.3, 143.1, 141.2, 137.6, 137.4, 133.4, 130.0, 129.9, 129.3, 128.5, 128.4, 128.3, 128.2, 128.1, 128.0, 127.9, 127.8, 127.1, 125.1, 120.0, 95.3, 75.3, 73.0, 71.7, 71.4, 70.0, 68.2, 68.0, 66.3, 46.7, 32.2 18.6, 13.5 ppm; IR (neat) ν_{max}: 1750, 1452, 1268, 1099, 1027 cm^{-1}; ESI-HRMS: m/z [M+Na]$^+$ calcd. for $C_{50}H_{55}O_{12}PNa$: 901.3323 found 901.3353.

^{1}H NMR ($CDCl_3$)

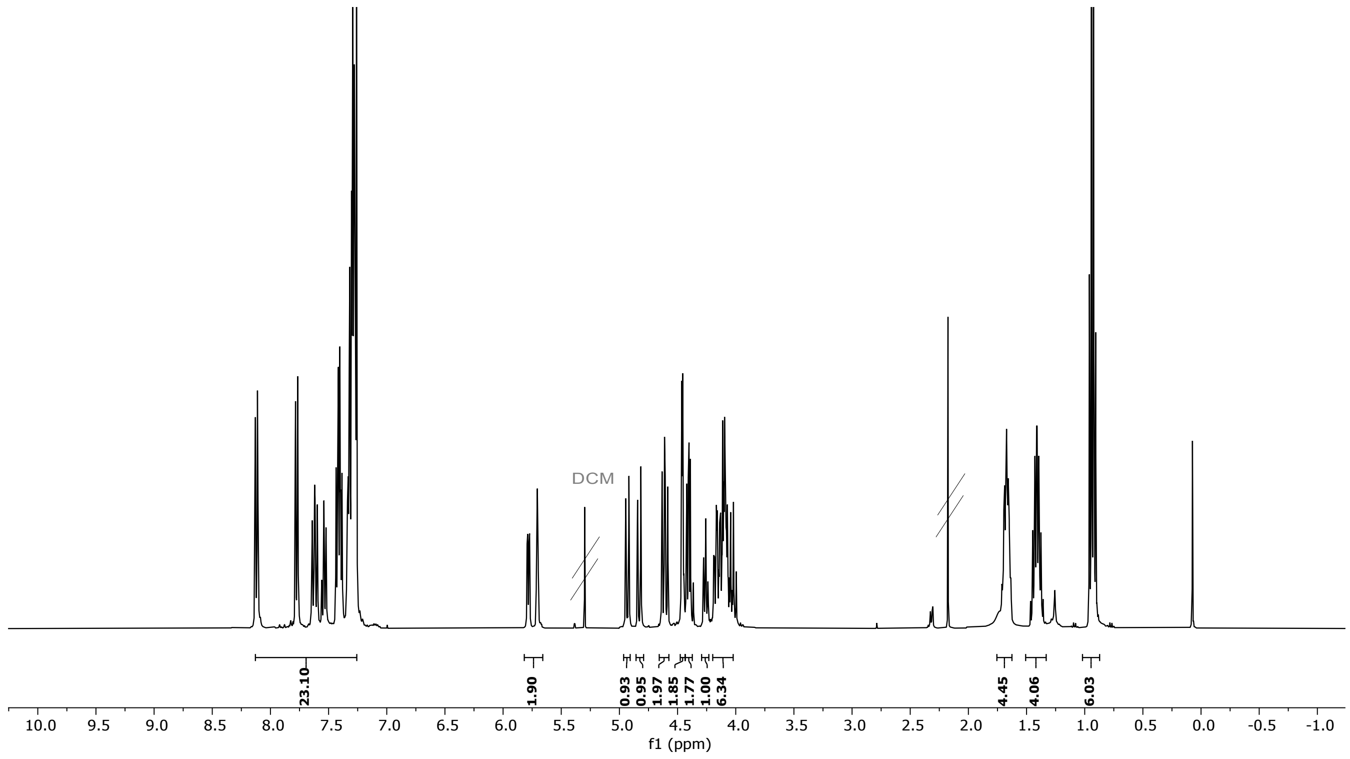

^{13}C NMR ($CDCl_3$)

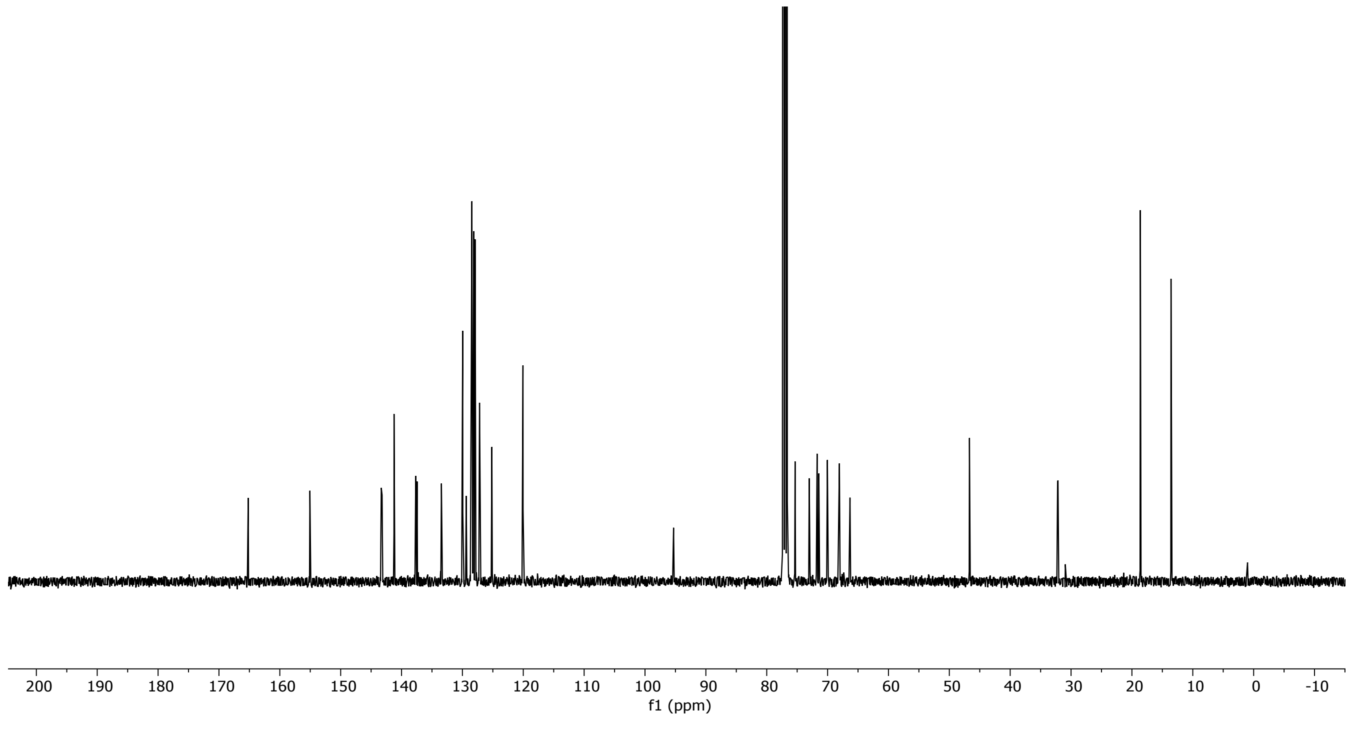

HSQC NMR ($CDCl_3$)

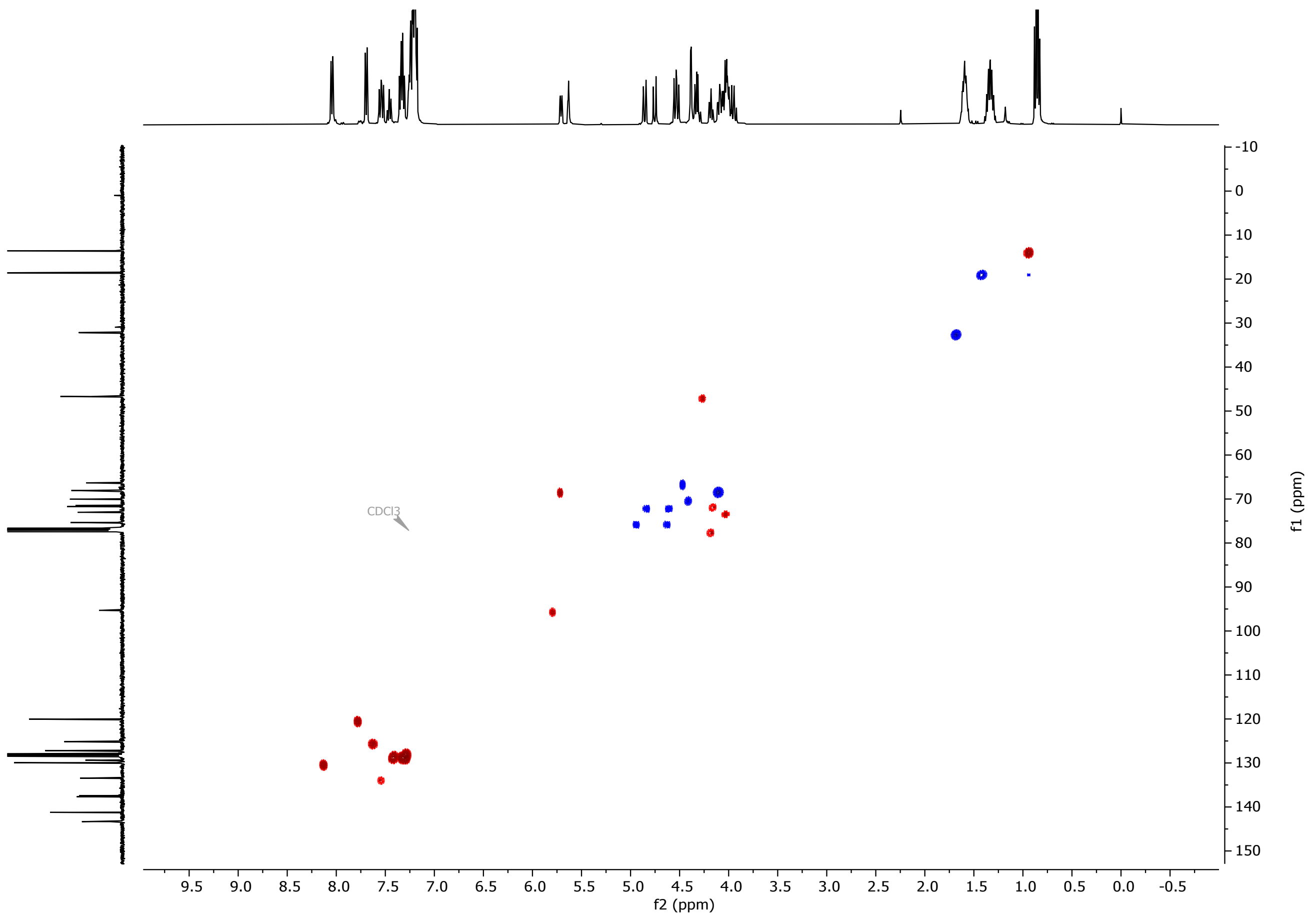

Synthesis of dibutoxyphosphoryloxy 2,3,4-*O*-tri-benzoyl-6-*O*-levulinoyl-α-D-mannopyranosyl phosphate (8)

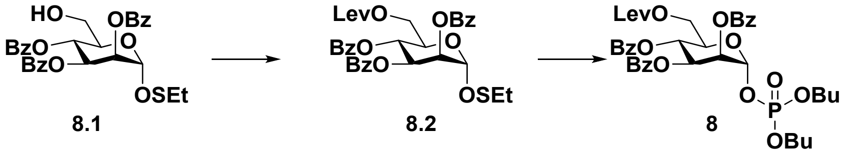

Ethyl 2,3,4-tri-*O*-benzoyl-1-thio-α-D-mannosylpyranoside **8.1** was synthesized as reported in the literature.[6]

*Ethyl 2,3,4-tri-O-benzoyl-6-O-levulinoyl-1-thio-α-D-mannosylpyranoside (**8.2**)*

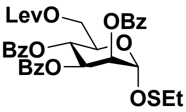

8.2

8.1 (0.44 g, 0.82 mmol, 1.00 equiv.) was dissolved in 22 mL of anhydrous DCM under argon atmosphere and cooled down to 0 °C. *N,N*-diisopropylcarbodiimide (DIC) (0.24 mL, 1.56 mmol, 1.90 equiv.), 4-(Dimethyl-amino)pyridine (DMAP) (0.11 g, 0.90 mmol, 1.10 equiv.) and levulinic acid (0.13 g, 1.15 mmol, 1.40 equiv.), were added and the reaction was protected with aluminum foil from the light. The mixture was allowed to warm slowly overnight to room temperature. The reaction was quenched by adding $NaHCO_3$-solution (50 mL). The aqua phase washed with ethyl acetate (2 × 50 mL) and the combined organic layer dried over $MgSO_4$ and the solvent was removed under reduced pressure. The crude product was purified by flash chromatography using a mixture of hexane/ethyl acetate (2:1) as eluent. The product was obtained as a white foam in 93% yield (0.49 g, 0.77 mmol). ^1H NMR (400 MHz, $CDCl_3$): δ = 8.10 – 7.32 (m, 14H, *-Ar*), 7.25 – 7.22 (m, 1H, *-Ar*), 5.98 (d, *J* = 3.2 Hz, 1H, *H*-2), 5.80 (t, *J* = 10.0 Hz, 1H, *H*-4), 5.62 (dd, *J* = 10.1, 3.4 Hz, 1H, *H*-3), 5.07 (d, *J* = 1.1 Hz, 1H, *H*-1), 4.43 – 4.30 (m, 2H, *H*-6a, *H*-6b), 4.04

(ddd, *J* = 9.4, 5.5, 3.4 Hz, 1H, *H*-5), 2.87 – 2.70 (m, 4H, -C*H₂*-, Lev, -C*H₂*-CH₃, SEt), 2.58 (m, 2H, -C*H₂*-, Lev), 2.17 (s, 3H, -CO-C*H₃*, Lev), 1.34 (t, *J* = 7.4 Hz, 3H, -CH₂-C*H₃*, SEt) ppm; ^{13}C NMR (101 MHz, CDCl₃): δ = 206.5, 172.5, 130.2, 129.9, 129.3, 128.9, 128.7, 128.6, 128.4, 83.0, 76.6, 72.7, 71.4, 67.1, 63.6, 38.0, 29.9, 27.9, 26.0, 15.1 ppm; ESI-LRMS: m/z [M+Na]$^+$ calcd. for $C_{34}H_{34}O_{11}SNa$: 673.1 found 673.0.

∗ Product was used without further characterization

^{1}H × NMR

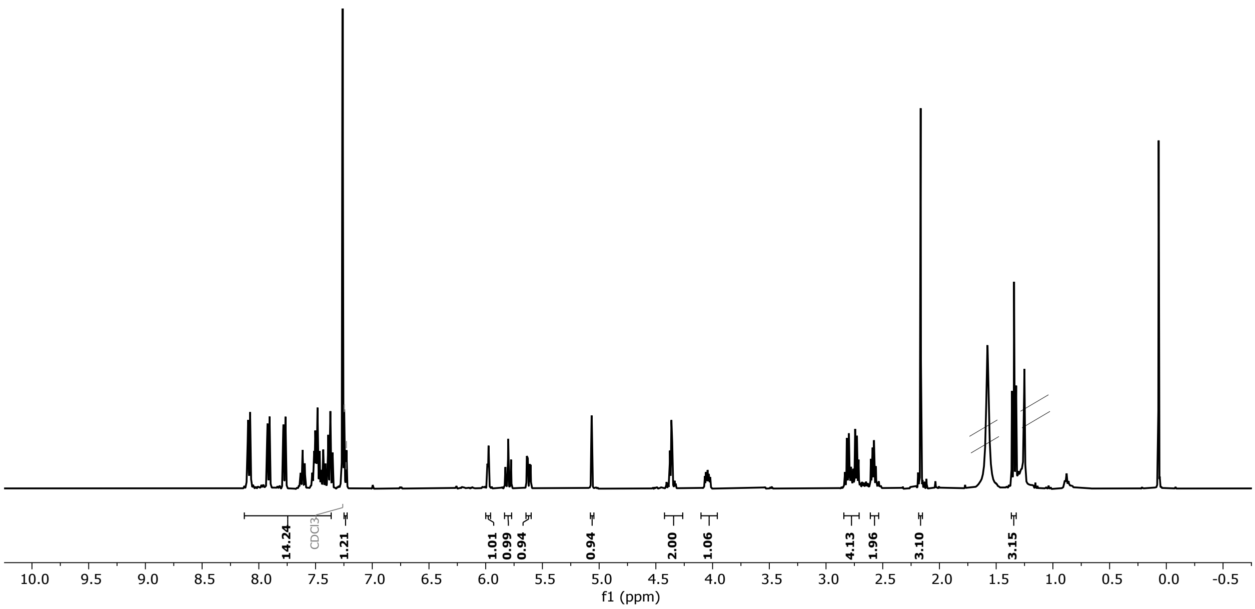

^{13}C × NMR

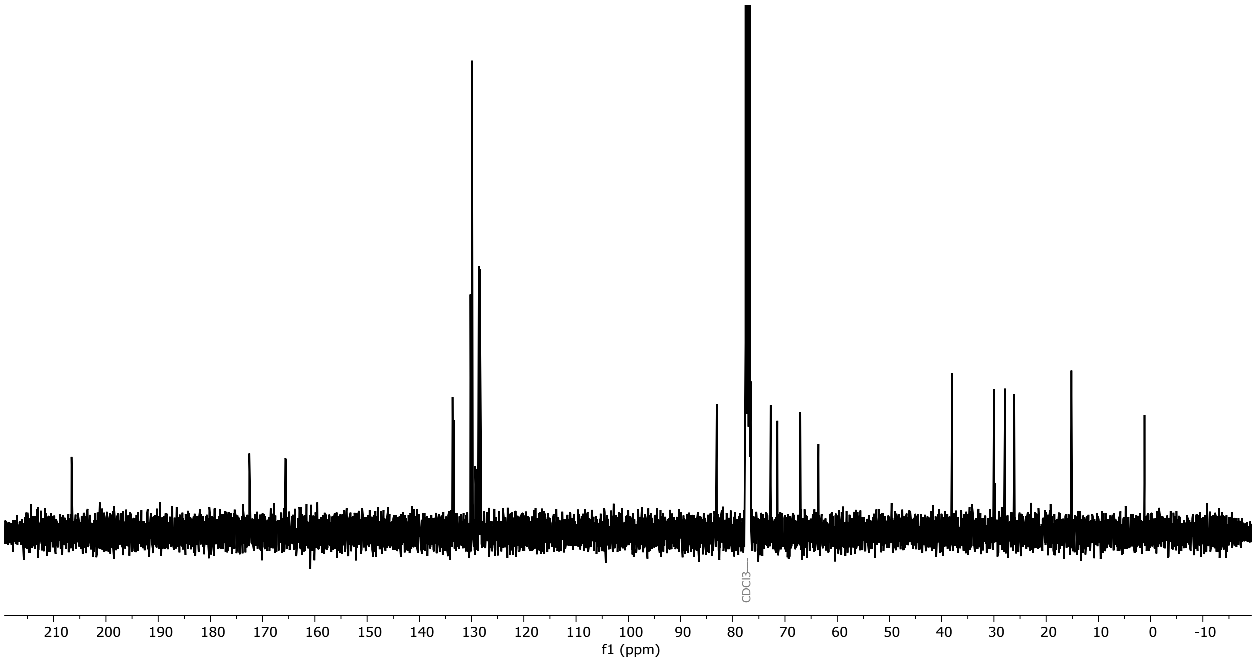

HSQC × NMR

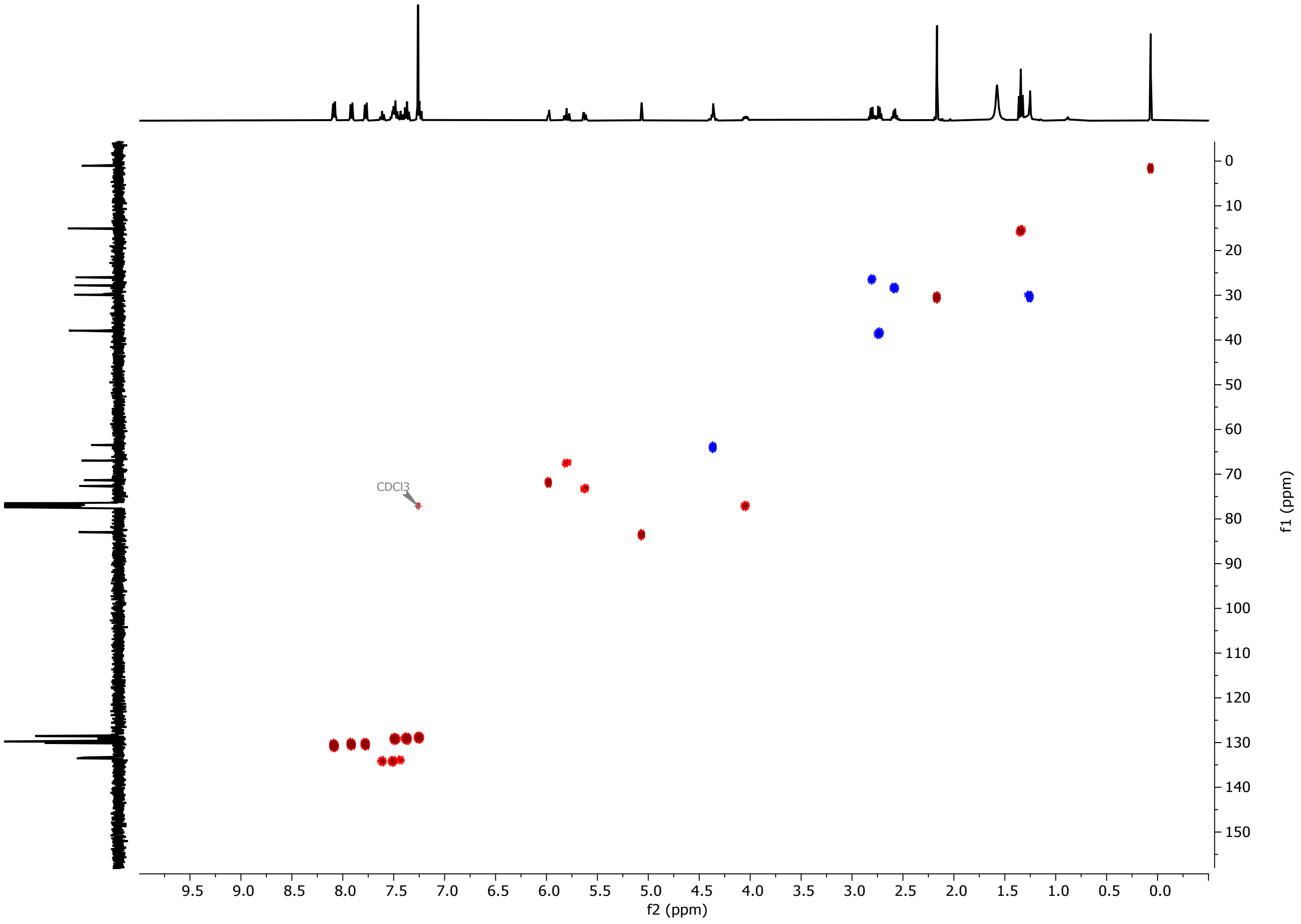

*Dibutoxyphosphoryloxy 2,3,4-tri-O-benzyl-6-O-levulinoyl-α-D-mannopyranosyl phosphate (**8**)*

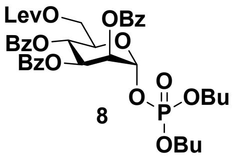

Dibutyl hydrogen phosphate (0.17 mL, 0.87 mmol, 2.00 equiv.) was added to a round-bottom flask containing activated 4Å molecular sieves anhydrous DCM (5.00 mL) and left stirring for 1.5 h. The molecular sieves were allowed to settle and the supernatant (5.00 mL) was added to a solution of the donor **8.2** (275 mg, 0.43 mmol, 1.00 equiv.) in anhydrous DCM (3.00 mL). The mixture was cooled down to 0 °C, and *N*-iodosuccinimide (NIS) (120 mg, 0.42 mmol, 1.23 equiv.) and triflic acid (10.0 µL, 0.13 mmol, 0.30 equiv.) were added. The reaction was stirred for 1h and then quenched with $NaHCO_3$ (1.00 mL). The organic layer was washed with $Na_2S_2O_3$ (5.00 mL) and water (5.00 mL), dried with Na_2SO_4, filtered and the solvent was removed under reduced pressure. The crude product was purified by flash column chromatography using a mixture of hexane/ethyl acetate (2:1.5). The product was obtained as a yellow/orange oil in 79% yield (266 mg, 0.34 mmol). ^{1}H NMR (400 MHz, $CDCl_3$): δ = 8.12 – 7.28 (m, 14H, -*Ar*), 7.25 (m, 1H, -*Ar*), 5.99 (t, *J* = 10.1 Hz, 1H, *H*-4), 5.92 – 5.85 (m, 2H, *H*-1, *H*-2), 5.75 (t, *J* = 2.6 Hz, 1H, *H*-3), 4.52 (dt, *J* = 10.0, 3.4 Hz, 1H, H-5), 4.37 (dd, *J* = 12.3, 4.2 Hz, 1H, *H*-6a), 4.30 (dd, *J* = 12.3, 2.8 Hz, 1H, *H*-6b), 4.20 (m, 4H, 2-OCH_2-, Bu), 2.83 – 2.68 (m, 2H, -CH_2-, Lev), 2.64 (m, 2H, -CH_2-, Lev), 2.16 (s, 3H, -CO-CH_3, Lev), 1.75 (p, *J* = 6.8 Hz, 4H, -CH_2-, Bu), 1.47 (h, *J* = 7.4 Hz, 4H, -CH_2-, Bu), 0.97 (t, *J* = 7.4 Hz, 6H, 2-CH_3, Bu) ppm; ^{13}C NMR (101 MHz, $CDCl_3$) δ 206.3, 172.3, 165.3, 165.0, 133.8, 133.6, 133.3, 129.9, 129.8, 129.7, 128.9, 128.8, 128.7, 128.5, 128.3, 94.8, 70.3, 69.7, 69.6, 69.1, 68.4, 68.4, 68.3, 68.3, 66.0, 62.4, 37.8, 32.3, 32.3, 32.2, 32.2, 29.8, 27.7, 18.6, 13.6 ppm; IR (neat) ν_{max}: 1733, 1453,1262, 1095, 1027cm^{-1}; ; ESI-HRMS: m/z [M+Na]$^+$ calcd. for $C_{40}H_{47}O_{14}PNa$: 805.2595 found 805.2606.

^{1}H × NMR

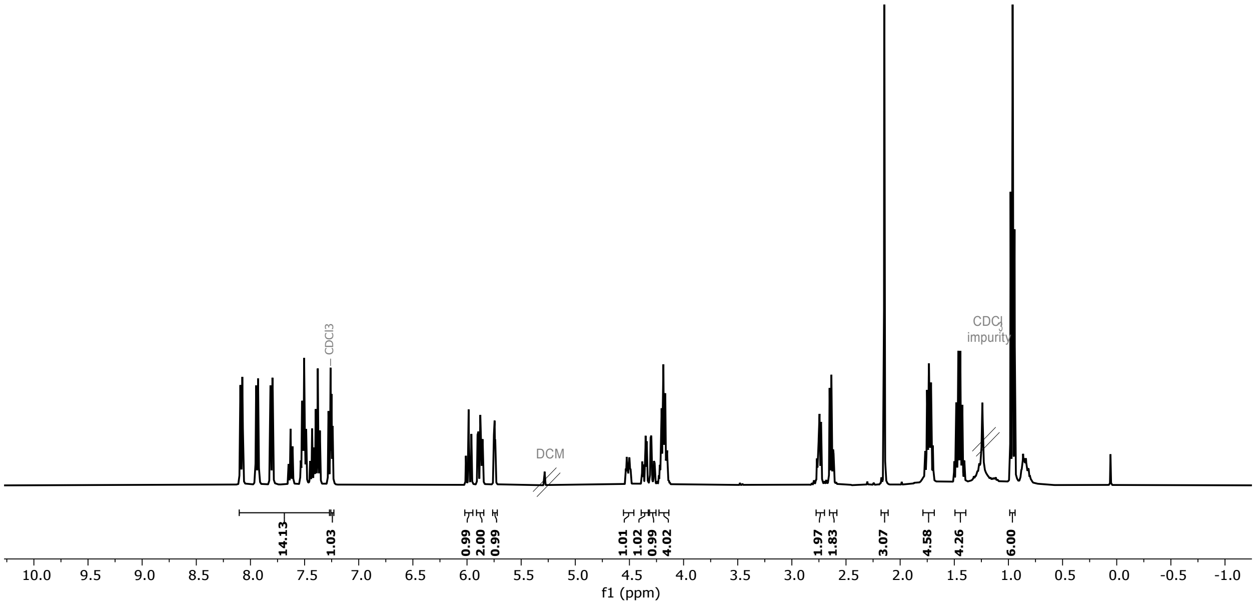

^{13}C × NMR

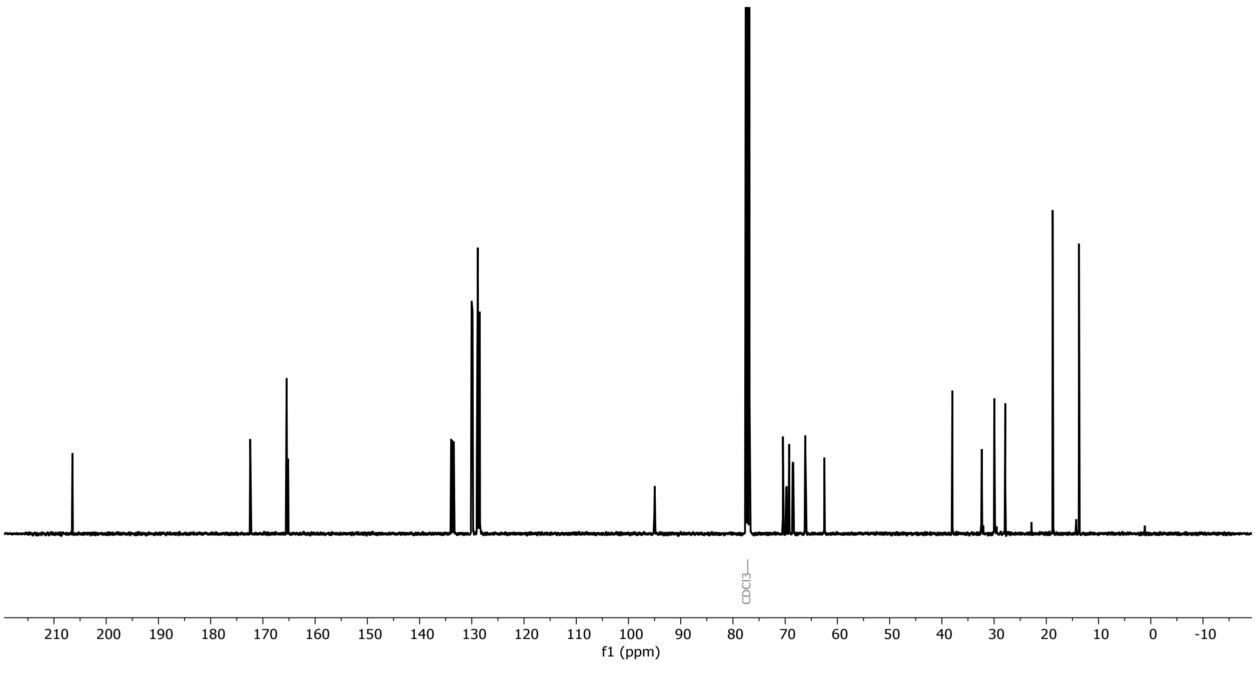

HSQC × NMR

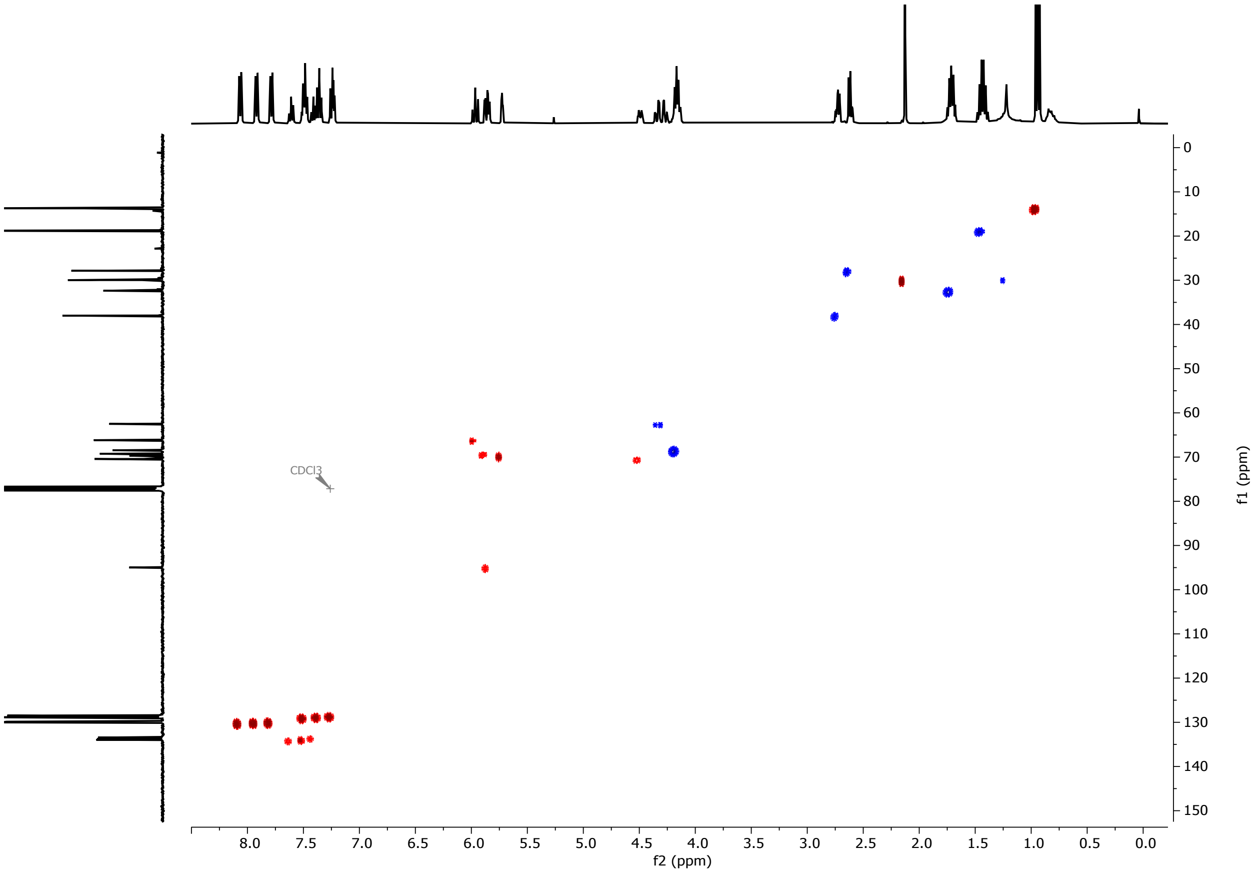

Synthesis of 2,3,4-*O*-tri-benzoyl-6-*O*-(9-fluorenylmethoxycarbonyl)-D-galactopyranosyl trichloroacetimidate (10)

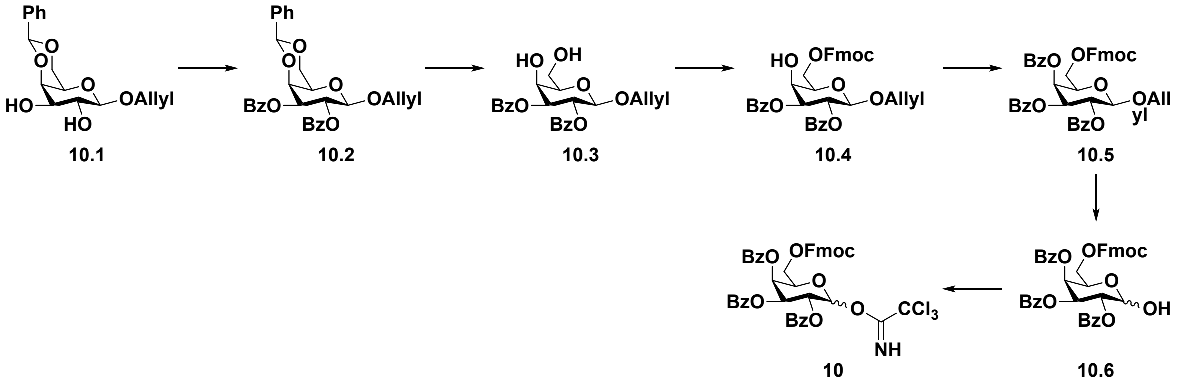

*Allyl 2,3-di-O-benzoyl-4,6-O-benzylidene-β-D-galactopyranoside (**10.2**)*

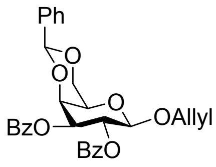

10.2

Allyl 4,6-*O*-benzylidene-β-D-galactopyranoside **10.1** (3.00 g, 9.73 mmol, 1.00 equiv.) was dissolved in pyridine (30 mL) and the solution was cooled to 0°C. BzCl (2.91 mL, 25.3 mmol, 2.60 equiv.) was added dropwise to the solution and left to react overnight at rt. Then, 4-dimethylaminopyridine (DMAP) (590 mg, 4.86 mmol, 0.50 equiv.) was added and the mixture was heated up to 50°C until completion). After completion, the mixture was poured into iced water; the precipitate was filtered off and washed with water. The solid was dissolved in DCM (100 mL) and washed with water (100 mL). Then, the organic layer was washed with hydrochloric acid (1 M, 100 mL), $NaHCO_3$-solution (100 mL), and water (100 mL). The organic layer was

dried over Na_2SO_4 and the solvent was removed under reduced pressure. The crude product was used without any further purification.

*Allyl 2,3-di-O-benzoyl-β-D-galactopyranoside (**10.3**)*

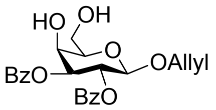

10.3

TFA (4.47 mL, 58.1 mmol, 7.50 equiv.) and water (0.60 mL, 33.3 mmol, 4.30 equiv.) were added to a solution of **10.2** (4.00 g, 7.74 mmol, 1.00 equiv.) in DCM (105 mL). The mixture was stirred for 3 h, diluted with DCM, washed with 10% (w/v) aqueous $NaHCO_3$-solution until neutral pH; and 10% (w/v) aqueous NaCl-solution. The organic layer was dried over Na_2SO_4, filtered, and concentrated. The residue was purified by flash column chromatography using a mixture of hexane/ethyl acetate (1:2) as eluent. The product was obtained as a white solid in 62% yield over two steps (2.04 g, 4.76 mmol). ^1H NMR (400 MHz, $CDCl_3$): δ = 7.98 (ddd, *J* = 8.5, 3.3, 1.4 Hz, 4H, *-Ar)*, 7.62 – 7.32 (m, 6H, *-Ar*), 5.86 – 5.73 (m, 2H, *H*-2, -CH_2-C*H*=CH_2 Allyl), 5.30 (dd, *J* = 10.3, 3.1 Hz, 1H, *H*-3), 5.25 (dd, *J* = 17.3, 1.7 Hz, 1H, -CH_2-CH=C*H*H Allyl), 5.13 (dd, *J* = 10.5, 1.5 Hz, 1H, -CH_2-CH=C*H*H Allyl), 4.77 (d, *J* = 8.0 Hz, 1H, *H*-1), 4.38 (m, 2H, *H*-4, -C*H*H-CH=CH_2 Allyl), 4.18 (m, 1H, -C*H*H-CH=CH_2 Allyl), 4.14 – 4.02 (m, 1H, *H*-6a), 3.95 (dd, *J* = 11.8, 4.4 Hz, 1H, *H*-6b), 3.78 (m, 1H, *H*-5); ^{13}C NMR (101 MHz, $CDCl_3$): δ = 166.0, 165.5, 133.7, 133.6, 133.2, 130.0, 129.8, 129.6, 129.1, 128.6, 128.4, 117.8, 100.5, 74.4, 74.2, 70.2, 69.6, 68.6, 62.8 ppm; IR (neat) ν_{max}: 3478, 1723, 1603, 1452, 1316, 1279, 1179, 1111, 1071, 1029 cm^{-1}; ESI-HRMS: m/z $[M+Na]^+$ calcd. for $C_{23}H_{24}O_8Na$: 451.1363 found 451.1360.

^1H NMR ($CDCl_3$)

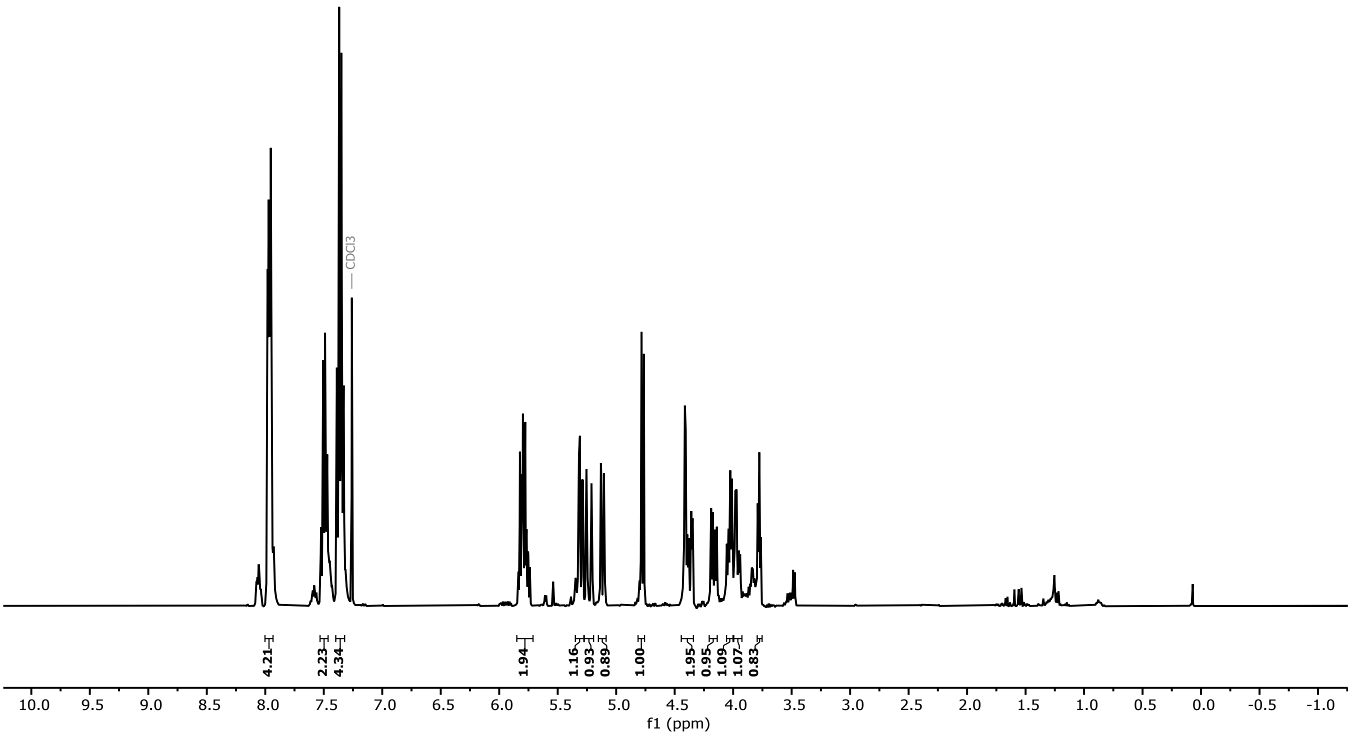

^{13}C NMR ($CDCl_3$)

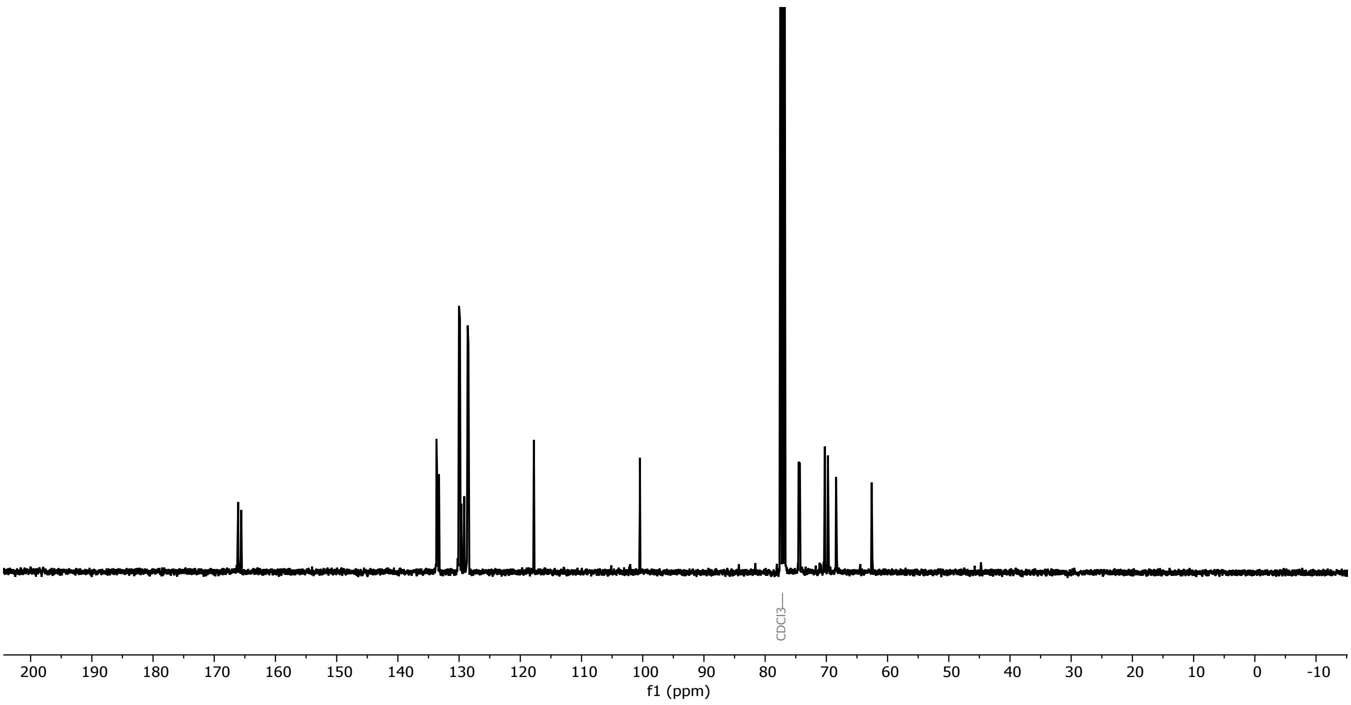

HSQC NMR ($CDCl_3$)

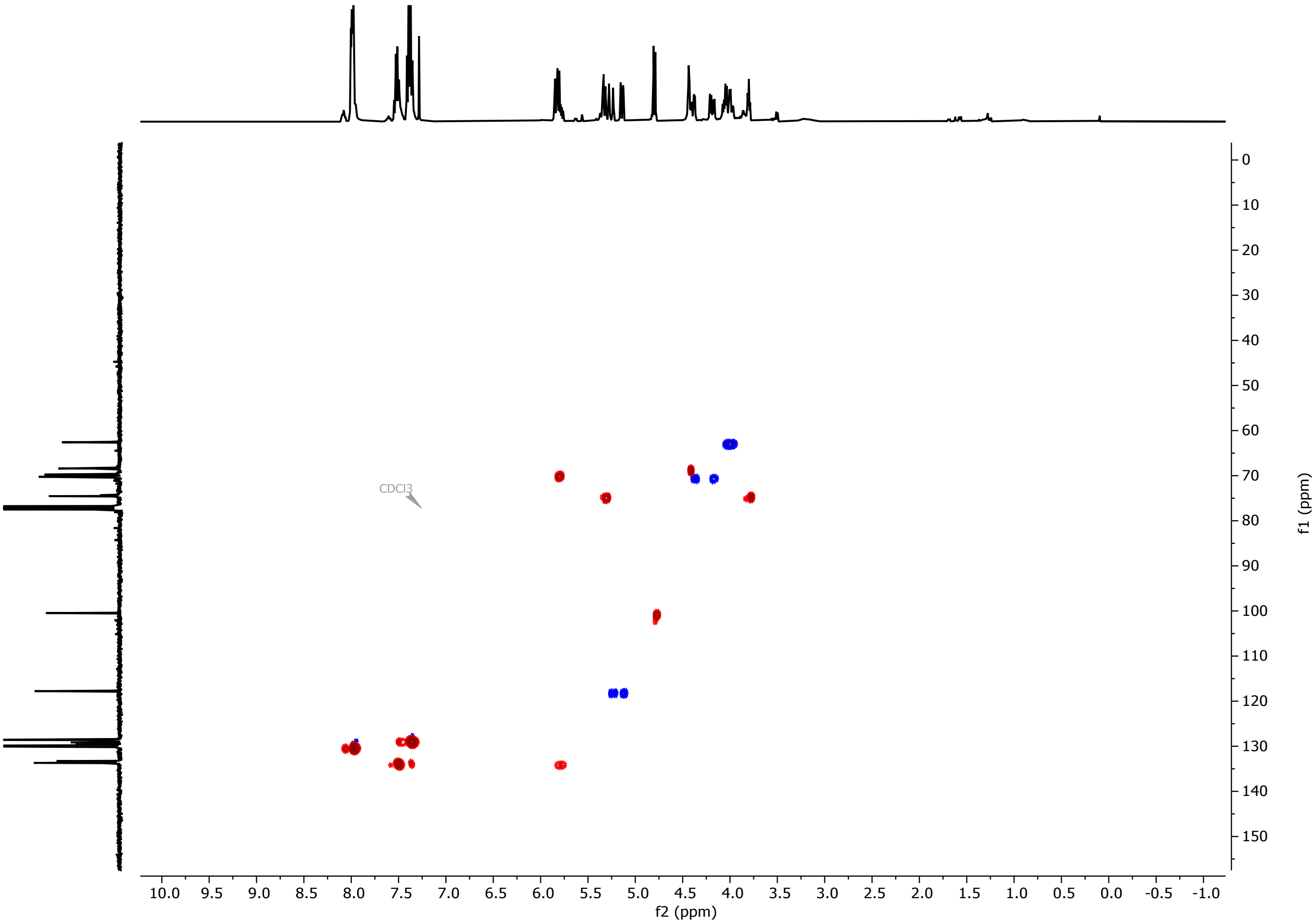

*Allyl 2,3-di-O-benzoyl-6-O-(9-fluorenylmethoxycarbonyl)-β-D-galactopyranoside (**10.4**)*

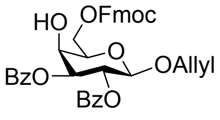

10.4

To a stirred suspension of **10.3** (1.30 g, 2.94 mmol, 1.00 equiv.) in anhydr. DCM (51 mL), pyridine (1.18 mL, 14.7 mmol, 5.00 equiv.) was added and the solution was stirred for 15 min at rt. Then, the mixture was cooled to 0°C and FmocCl (910 mg, 3.52 mmol, 1.20 equiv.) was added. After complete conversion (2 h), the solvent was removed and the residue was purified by flash column chromatography using a mixture of hexane/ethyl acetate (4:1 and 1% toluene) to yield the titled compound as a yellowish solid in 50% yield (962 mg, 1.48 mmol). ^{1}H NMR (400 MHz, $CDCl_3$): δ = 7.99 (m, 4H, -*Ar*), 7.78 (ddt, *J* = 7.6, 2.1, 0.9 Hz, 2H, -*Ar*), 7.62 (ddt, *J* = 7.6, 1.8, 0.9 Hz, 2H, -*Ar*), 7.56 – 7.44 (m, 2H, -*Ar*), 7.43 – 7.30 (m, 8H, -*Ar*), 5.86 – 5.72 (m, 2H, -CH_2-C*H*=CH_2 Allyl, *H*-2), 5.33 (dd, *J* = 10.3, 3.2 Hz, 1H, *H*-3), 5.24 (dd, *J* = 17.3, 1.7 Hz, 1H, -CH_2-CH=C*H*H Allyl), 5.13 (dd, *J* = 10.4, 1.4 Hz, 1H, -CH_2-CH=CH*H* Allyl,), 4.75 (d, *J* = 7.9 Hz, 1H, *H*-1), 4.53 – 4.42 (m, 4H, *H*-6a, *H*-6b, -C*H₂*-CH- Fmoc), 4.37 (ddt, *J* = 13.2, 4.9, 1.6 Hz, 1H, -CH*H*-CH=CH_2 Allyl,), 4.33 – 4.23 (m, 2H, *H*-4, *H*-5), 4.17 (ddt, *J* = 13.2, 6.3, 1.4 Hz, 1H, -C*H*H-CH=CH_2), 3.96 (t, *J* = 6.3 Hz, 1H, -C*H*- Fmoc), 2.32 (s, 1H, -O*H*) ppm; ^{13}C NMR (101 MHz, $CDCl_3$): δ = 165.8, 165.3, 155.0, 143.2, 143.2, 141.3, 133.6, 133.4, 133.2, 129.9, 129.7, 129.4, 128.9, 128.5, 128.3, 127.9, 127.2, 125.1, 125.1, 120.1, 117.8, 100.0, 74.0, 72.0, 70.1, 69.9, 69.4, 67.2, 65.7, 46.7 ppm; IR (neat) ν_{max}:2354, 1728, 1451, 1263, 1110 cm^{-1}; ESI-HRMS: m/z [M+Na]$^+$ calcd. for $C_{38}H_{34}O_{10}Na$: 673.2044 found 673.2068 .

^{1}H NMR ($CDCl_3$)

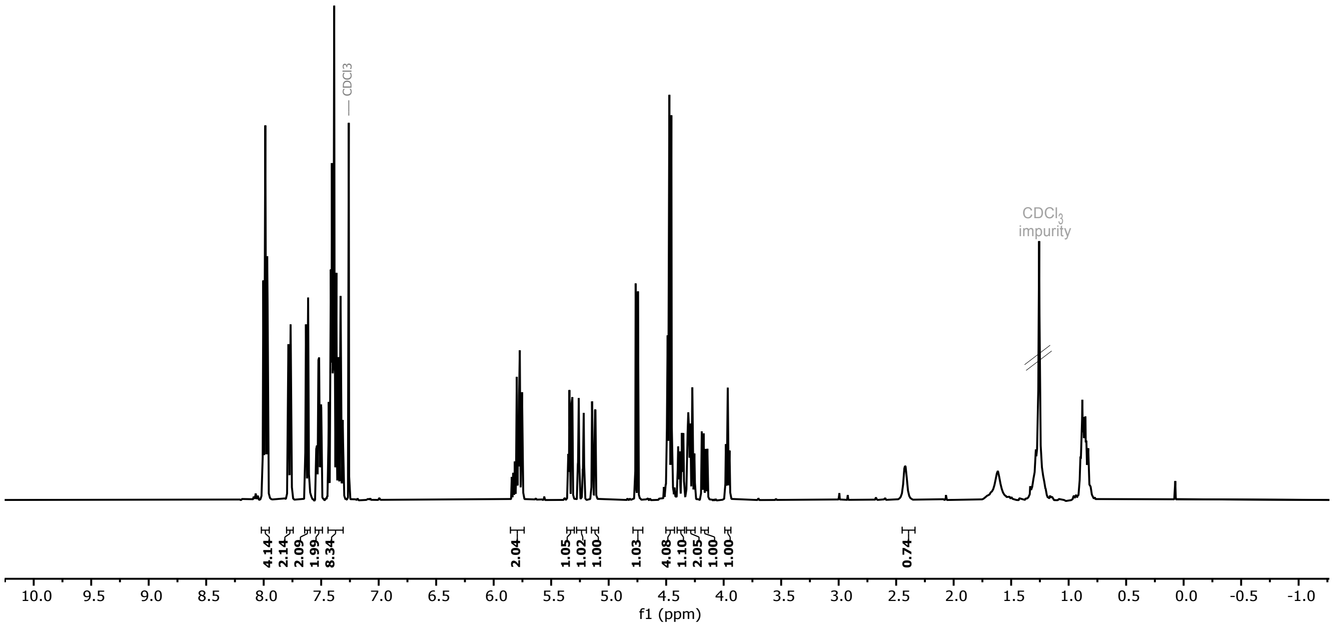

^{13}C NMR ($CDCl_3$)

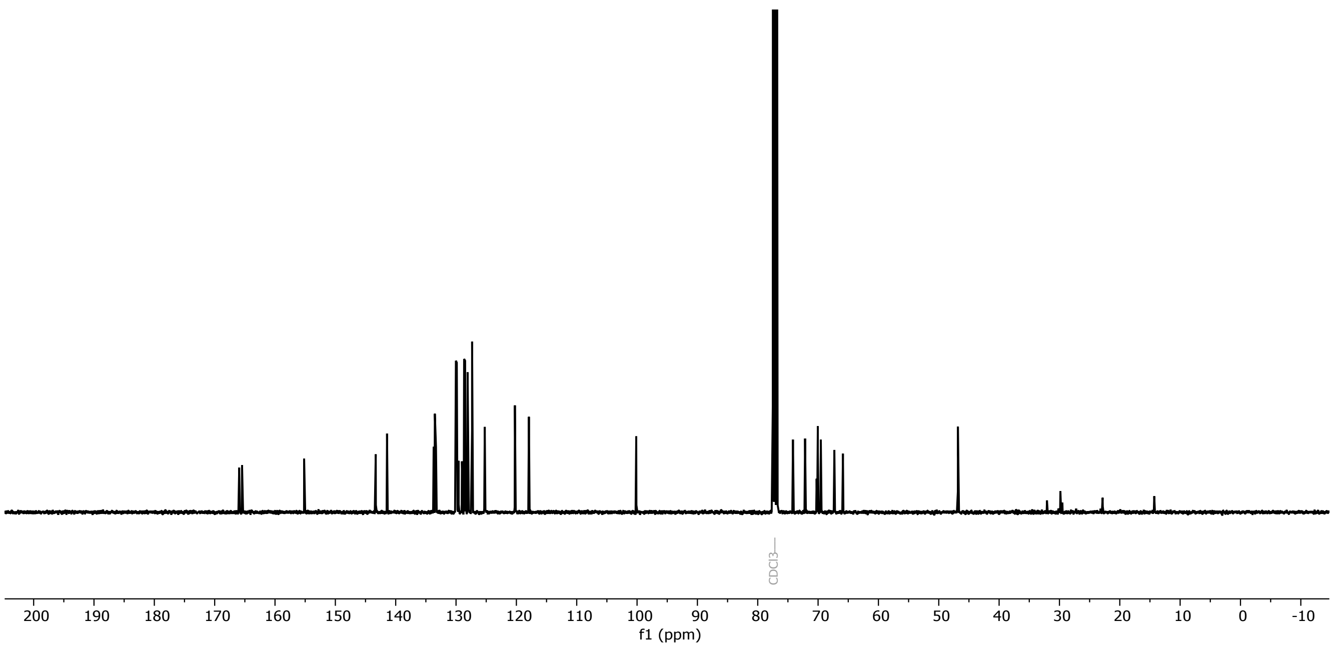

HSQC NMR ($CDCl_3$)

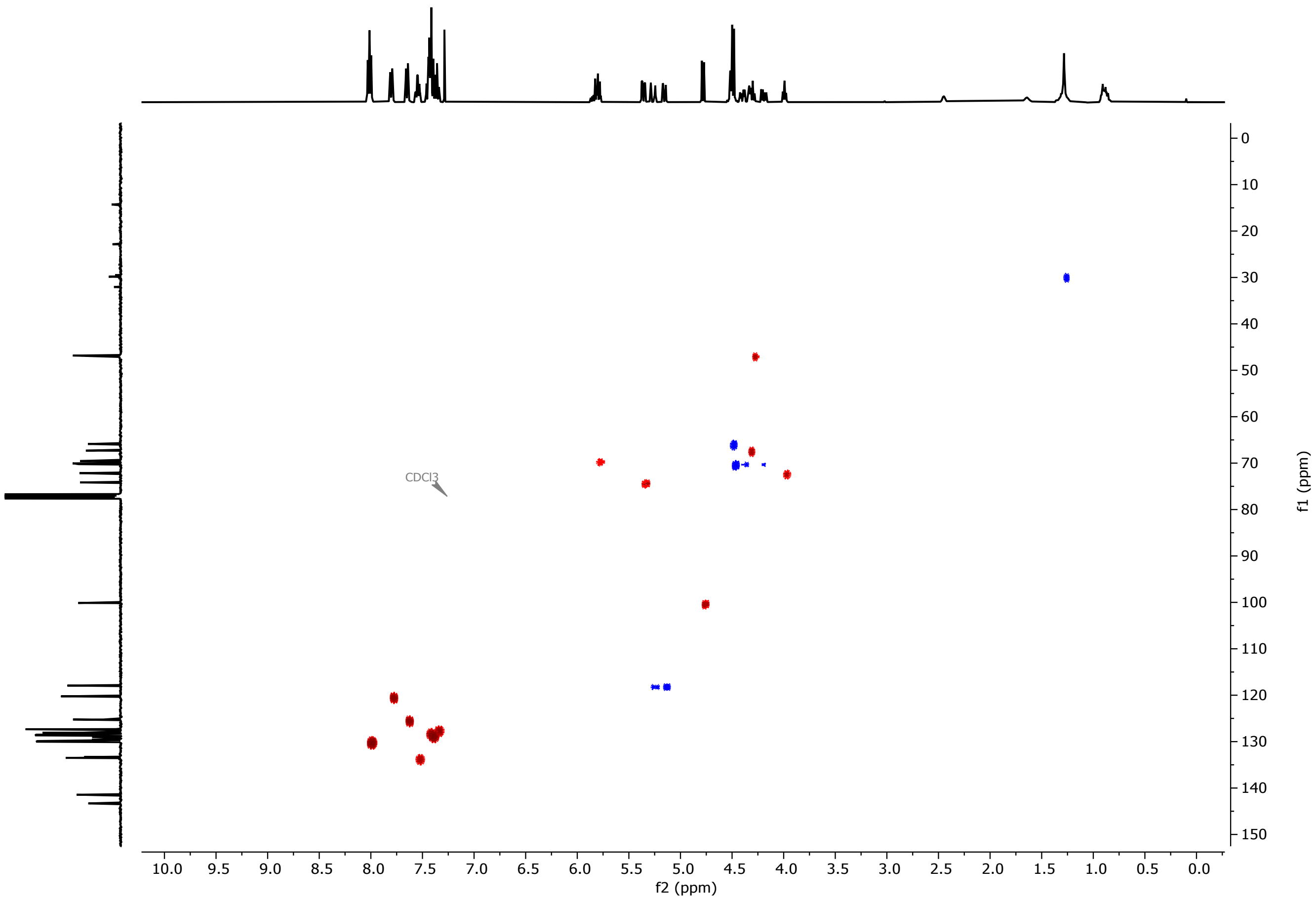

*Allyl 2,3,4-tri-O-benzoyl-6-O-(9-fluorenylmethoxycarbonyl)-β-D-galactopyranoside (**10.5**)*

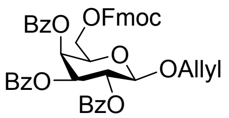

10.5

10.4 (960 mg, 1.47 mmol, 1.00 equiv.) was dissolved in pyridine (10 mL) and the solution was cooled to 0°C. BzCl (0.68 mL, 5.90 mmol, 4.00 equiv.) was added dropwise to the solution and the mixture was stirred for 2 h at rt. Then, the mixture was quenched with iced water. The precipitate was filtered off and washed with water (50 mL). The precipitate was dissolved in DCM and washed with water (50 mL). Then, the organic layer was washed with hydrochloric acid (1 M, 50 mL), $NaHCO_3$-solution (50 mL), and water (50 mL). The organic layer was dried over Na_2SO_4 and the solvent was removed under reduced pressure. The crude product was purified by flash column chromatography using a mixture of hexane/ethyl acetate (3:1) as eluent. The product was obtained as a white foam in 94% yield (1.05 g, 1.39 mmol). ^{1}H NMR (400 MHz, $CDCl_3$): δ = 8.12 – 7.27 (m, 23H, -*Ar*), 5.92 (dd, *J* = 3.5, 1.2 Hz, 1H, *H*-4), 5.86 – 5.75 (m, 2H, -CH_2-C*H*=CH_2 Allyl, *H*-2), 5.57 (dd, *J* = 10.4, 3.4 Hz, 1H, *H*-3), 5.27 (dd, *J* = 17.2, 1.6 Hz, 1H, -CH_2-CH=C*H*H Allyl), 5.16 (dd, *J* = 10.5, 1.4 Hz, 1H, -CH_2-CH=C*H*H Allyl), 4.85 (d, *J* = 8.0 Hz, 1H, *H*-1), 4.47 – 4.38 (m, 4H, *H*-6a, -C*H*$_2$-CH- Fmoc, -C*H*H-CH=CH_2 Allyl), 4.36 (m, 1H, *H*-6b), 4.28 – 4.18 (m, 3H, -C*H*H-CH=CH_2 Allyl, *H*-5, -C*H*- Fmoc) ppm; ^{13}C NMR (101 MHz, $CDCl_3$): δ = 165.7, 165.6, 165.3, 154.8, 143.3, 143.3, 141.4, 133.7, 133.4, 133.4, 133.4, 130.2, 129.9, 129.8, 129.4, 129.0, 128.8, 128.7, 128.6, 128.5, 128.4, 128.0, 127.3, 127.3, 125.3, 125.2, 120.2, 118.1, 100.3, 71.8, 71.4, 70.4, 70.3, 69.7, 68.2, 65.7, 46.7 ppm; IR (neat) ν_{max}: 3068, 2982, 2928, 1726, 1603, 1452, 1251, 1177, 1069, 1027 cm^{-1}; ESI-HRMS: m/z [M+Na]$^+$ calcd. for $C_{45}H_{38}O_{11}Na$: 777.2306 found 777.2333.

^{1}H NMR ($CDCl_3$)

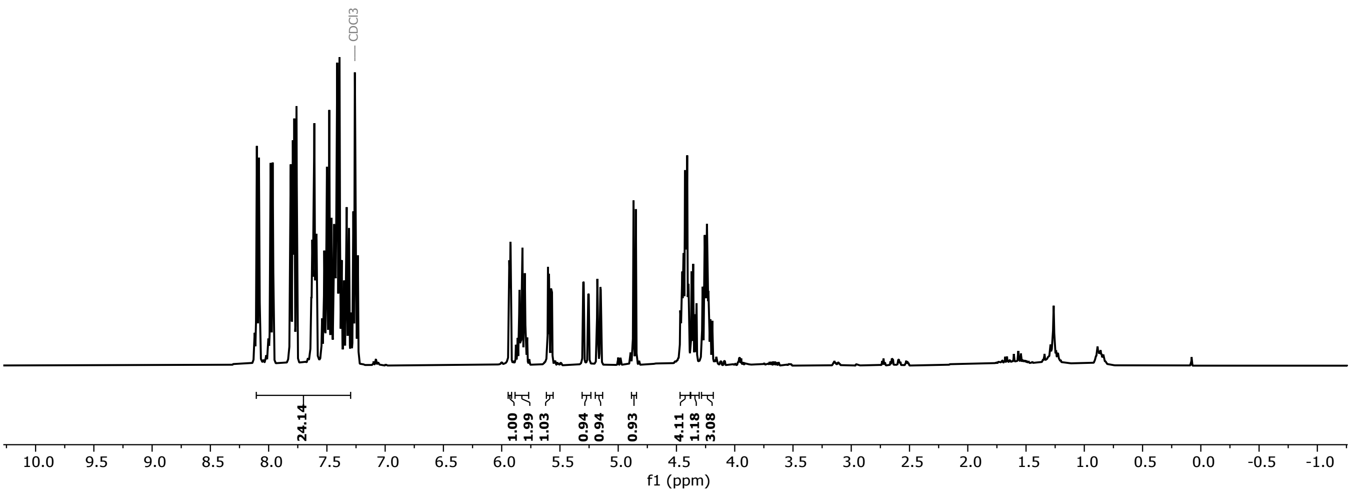

^{13}C NMR ($CDCl_3$)

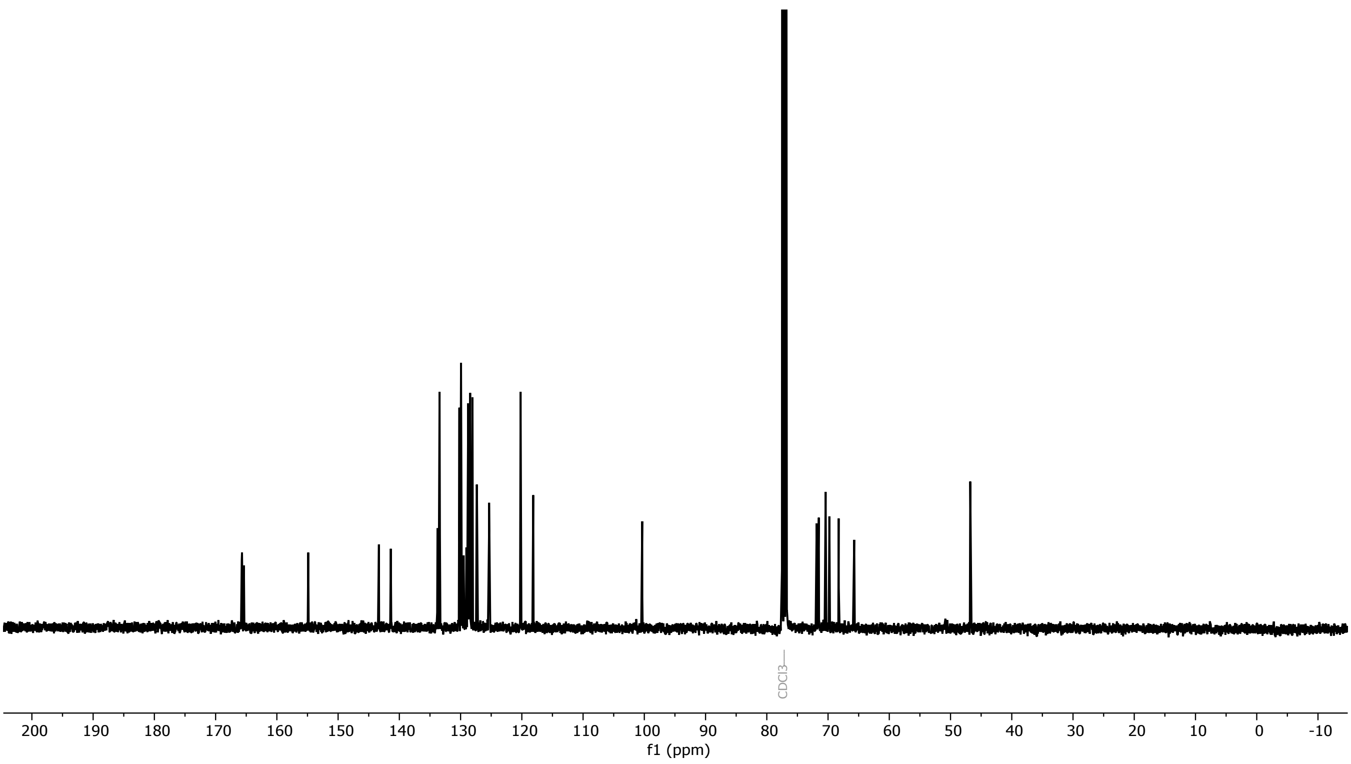

HSQC NMR ($CDCl_3$)

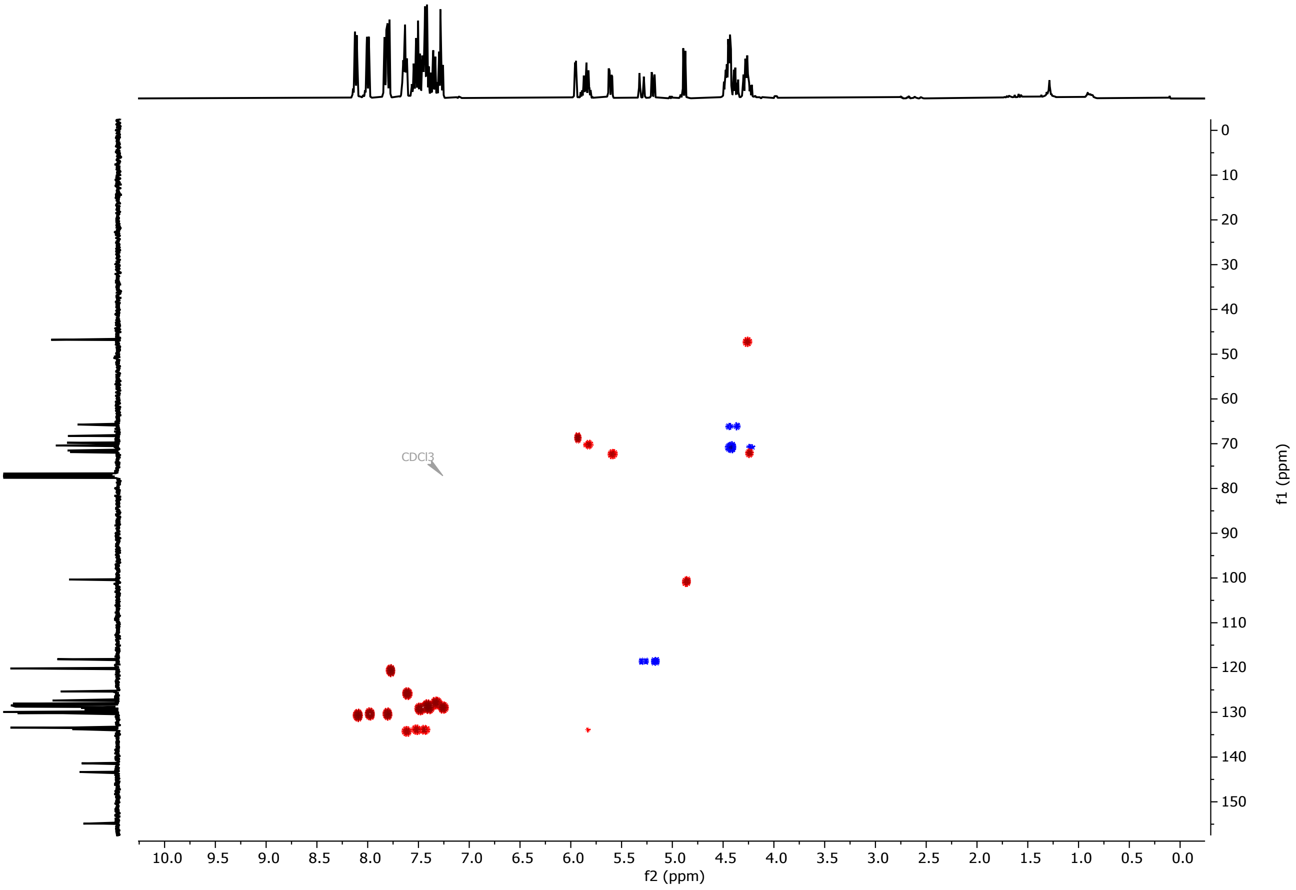

*2,3,4-tri-O-benzoyl-6-O-(9-fluorenylmethoxycarbonyl)-β-D-galactopyranoside (**10.6**)*

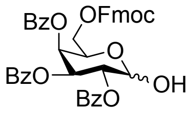

10.6

A mixture of **10.5** (980 mg, 1.30 mmol, 1.00 equiv.) and $PdCl_2$ (115 mg, 0.65 mmol, 0.50 equiv.) in MeOH/DCM (1:1 = 7 mL) was stirred for 5 h at rt. The reaction mixture was filtered through Celite® and concentrated under reduced pressure. The residue was purified by flash column chromatography using a mixture of hexane/ethyl acetate (2:1) as eluent. The product was obtained as a white solid in 68% yield (635 mg, 0.89 mmol). The crude product was used without any further purification. For α-anomer; ^{1}H NMR (400 MHz, $CDCl_3$): δ = 8.11 – 7.28 (m, 23H, -*Ar*), 6.03 (dd, *J* = 10.6, 3.4 Hz, 1H, *H*-3), 5.99 (dd, *J* = 3.5, 1.3 Hz, 1H; *H*-4), 5.84 (t, *J* = 3.6 Hz, 1H; *H*-1), 5.70 (dd, *J* = 10.7, 3.7, Hz, 1H; *H*-2), 4.76 (t, 1H; *J* = 6.0 Hz, -C*H*- Fmoc), 4.46 – 4.30 (m, 4H, *H*-6a, *H*-6b, -CH_2-Fmoc), 4.27 – 4.21 (m, 1H, *H*-5), 2.97 (dd, *J* = 3.7, 1.3 Hz, 1H, -O*H*) ppm; ^{13}C NMR (101 MHz, $CDCl_3$): δ = 165.6, 154.9, 143.3, 141.4, 133.7, 133.6, 133.3, 130.1, 129.9, 129.8, 129.1, 128.8, 128.6, 128.4, 128.0, 127.3, 125.3, 120.2, 91.1, 70.2, 69.4, 68.0, 67.1, 66.2, 60.5, 46.7 ppm. ESI-LRMS: m/z $[M+Na]^+$ calcd. for $C_{42}H_{34}O_{11}Na$: 737.2 found 737.3.

The analytical data agree with the literature[11]

*2,3,4-O-tri-benzoyl-6-O-(9-fluorenylmethoxycarbonyl)-D-galactopyranosyl trichloroacetimidate (**10**)*

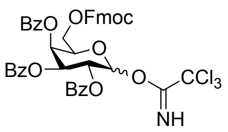

10

To a stirred solution of **10.6** (400 mg, 0.56 mmol, 1.00 equiv.) in trichloroacetonitrile (1.12 mL, 11.2 mmol, 20.00 equiv.) anhydr. DCM (5 mL), NaH (60 % dispersion in oil, 6.70 mg, 0.28 mmol, 0.50 equiv.) was added at rt under argon atmosphere and the reaction was left to react overnight. Upon completion, solvent (in presence of silica to quench the remaining NaH) was evaporated and the residue was purified by flash column chromatography using a mixture of hexane/ethyl acetate (3:1) to afford the desired compound as a white solid in 45% yield (208 mg, 0.24 mmol). ^{1}H NMR (400 MHz, $CDCl_3$): δ = 8.63 (s, 1H, =N*H*), 8.16 – 7.33 (m, 23H, -*Ar*), 6.91 (d, *J* = 3.6 Hz, 1H, *H*-1), 6.13 – 6.01 (m, 2H, *H*-4, *H*-3), 5.95 (dd, *J* = 10.6, 3.6 Hz, 1H, *H*-2), 4.81 (t, *J* = 6.2 Hz, 1H, *H*-5), 4.45 – 4.30 (m, 4H, -CH_2-CH- Fmoc, *H*-6a, *H*-6b), 4.22 (t, *J* = 7.6 Hz, 1H, -C*H*- Fmoc) ppm; ^{13}C NMR (101 MHz, $CDCl_3$): δ = 165.5, 165.5, 165.4, 160.5, 154.6, 143.2, 141.2, 141.2, 133.7, 133.5, 133.3, 129.9, 129.8, 129.7, 128.8, 128.7, 128.6, 128.4, 128.3, 127.8, 127.2, 127.1, 125.2, 120.0, 93.6, 70.2, 69.5, 68.3, 68.2, 67.7, 65.2, 46.5 ppm; IR (neat) ν_{max}: 3341, 3068, 2959, 2927, 1729, 1677, 1603, 1452, 1259, 1094, 1069, 1026 cm^{-1}; ESI-HRMS: m/z $[M+Na]^+$ calcd. for $C_{44}H_{34}Cl_3NO_{11}Na$: 880.1090 found 880.1104.

^{1}H NMR ($CDCl_3$)

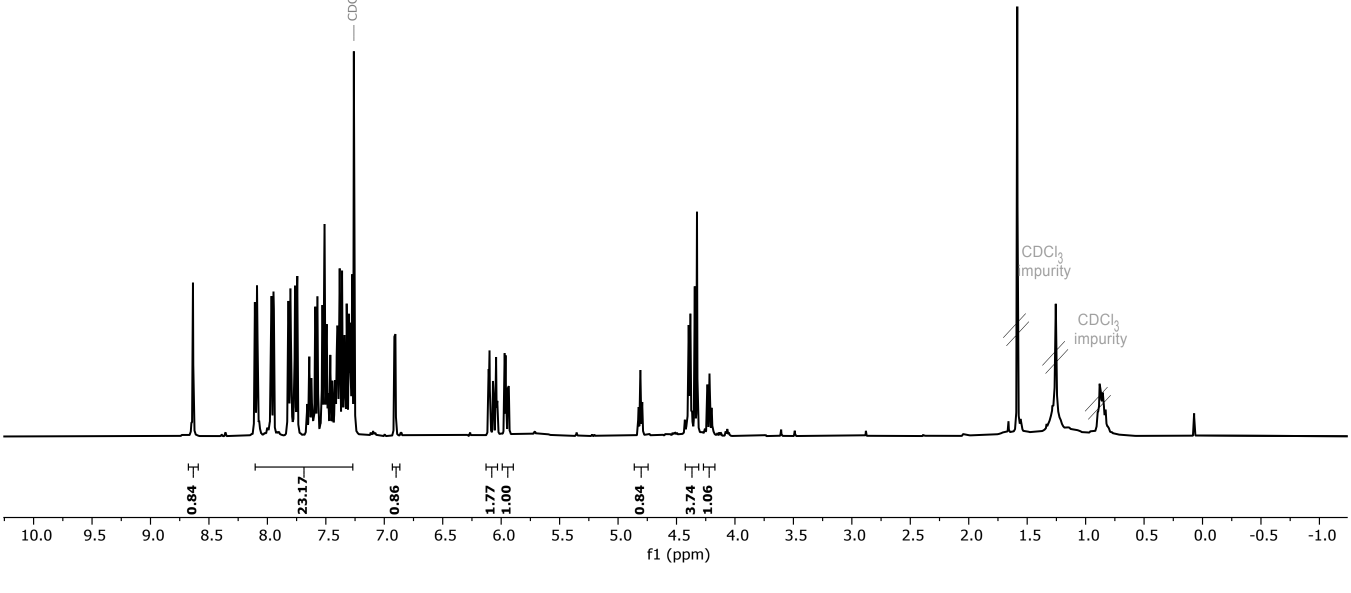

^{13}C NMR ($CDCl_3$)

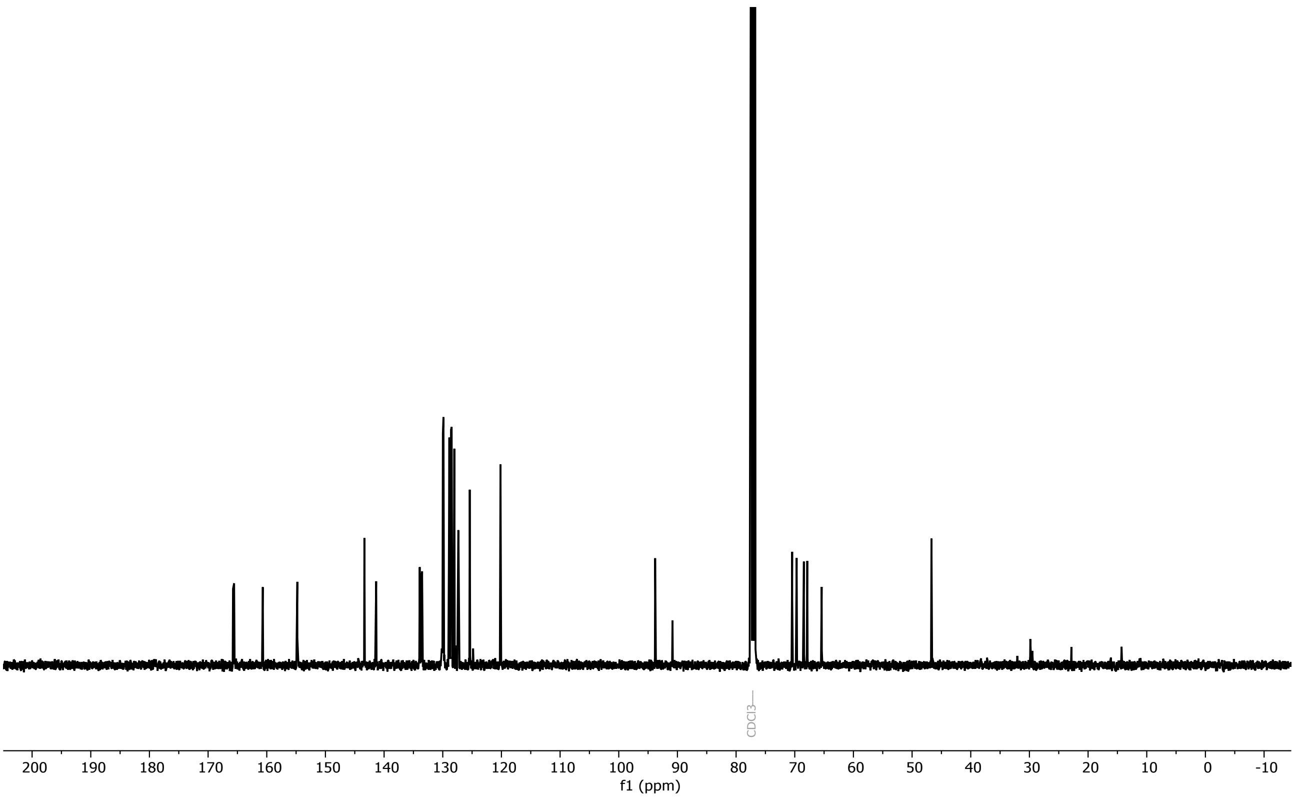

HSQC NMR ($CDCl_3$)

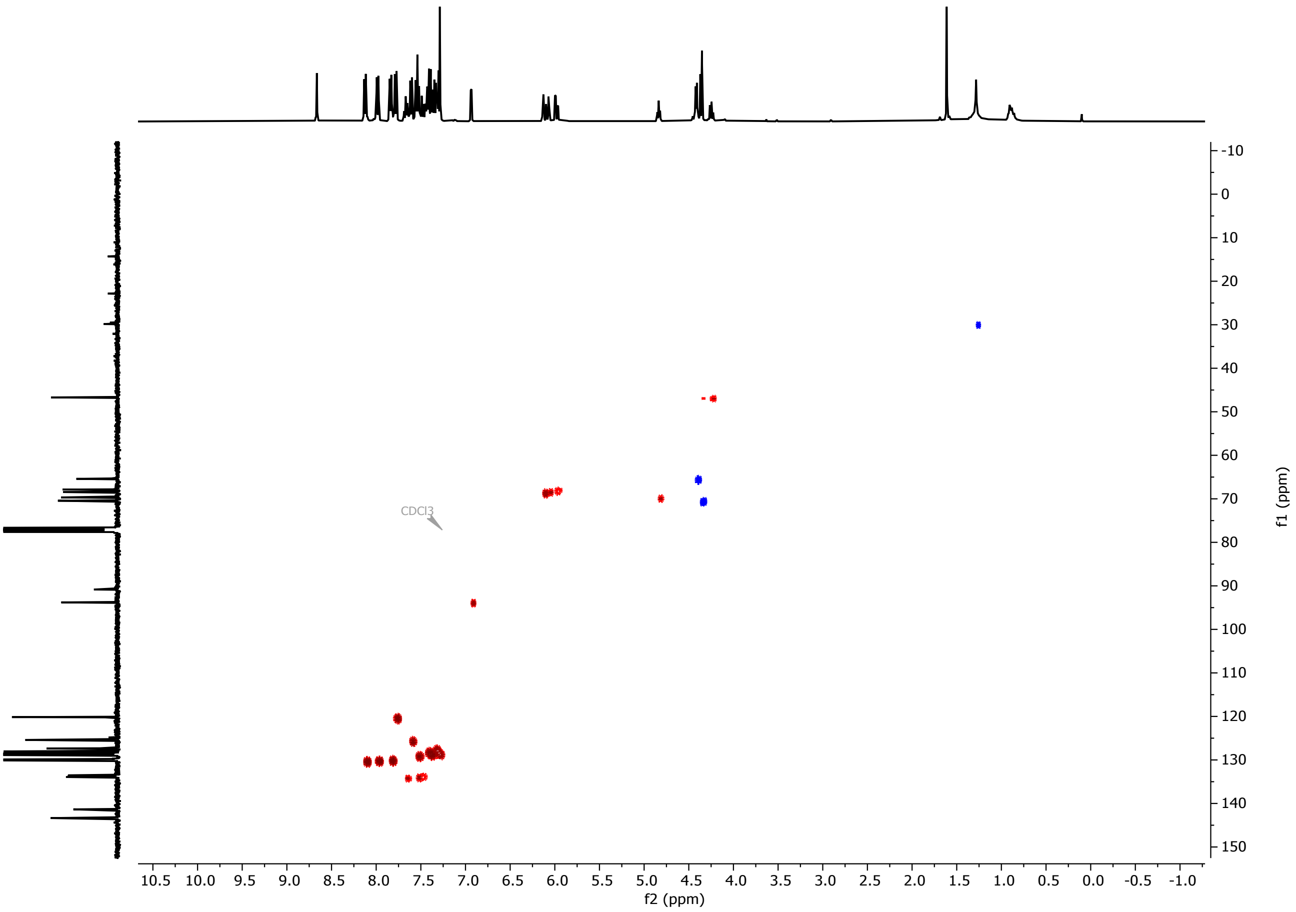

Synthesis of dibutoxyphosphoryloxy 2,3,4-tri-*O*-benzoyl-6-*O*-(9-fluorenylmethoxycarbonyl)-β-D-glucopyranosyl phosphate (27)

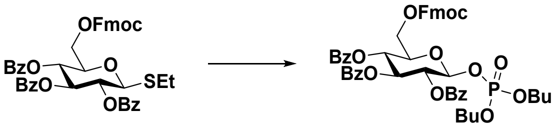

27.1 **27**

2,3,4-tri-*O*-benzoyl-6-*O*-(9-fluorenylmethoxycarbonyl)-1-thio-β-D-glucopyranoside **27.1** was synthesized as reported in the literature[12].

Dibutyl hydrogen phosphate (0.16 mL, 0.79 mmol, 3.00 equiv.) was added to a round-bottom flask containing activated 4Å molecular sieves anhydrous DCM (3.00 mL) and left stirring for 1.5h. The molecular sieves were allowed to settle and the supernatant (3.00 mL) was added to a solution of donor 40.1 (200 mg, 0.26 mmol, 1.00 equiv.) in anhydrous DCM (3.00 mL). The mixture was cooled down to 0°C and NIS (72.9 mg, 0.32 mmol, 1.23 equiv.) and triflic acid (6.0 µL, 0.79 mmol, 0.30 equiv.) were added. The reaction was stirred for 1h and then quenched with $NaHCO_3$-solution (1.00 mL). The organic layer was washed with $Na_2S_2O_3$ (5.00 mL) and water (5.00 mL), dried with Na_2SO_4, filtered and the solvent was removed under reduced pressure. The crude product was purified by flash column chromatography using a mixture of hexane/ethyl acetate (2:1.5). The product was obtained as a white solid in 79% yield (210 mg, 0.23 mmol, 88%). ^{1}H NMR (400 MHz, $CDCl_3$): δ = 8.02 – 7.91 (m, 4H, -*Ar*), 7.87 – 7.81 (m, 2H, -*Ar*), 7.77 (d, J = 7.0 Hz, 2H, -*Ar*), 7.61 (m, 2H, -*Ar*), 7.57 – 7.48 (m, 2H, -*Ar*), 7.47 – 7.27 (m, 11H, -*Ar*), 5.94 – 5.81 (m, 1H, *H*-2), 5.70 – 5.59 (m, 3H, *H*-1, *H*-3, *H*-4), 4.51 – 4.28 (m, 4H, 2-OC*H*$_2$-, Bu), 4.27 – 4.16 (m, 2H, *H*-5, -C*H*- Fmoc), 4.11 – 3.99 (m, 2H, -C*H*$_2$- Fmoc), 3.85 – 3.67 (m, 2H, *H*-6a, *H*-6b), 1.68 – 1.57 (m, 2H, -C*H*$_2$-, Bu), 1.39 – 1.17 (m, 4H, -C*H*$_2$-, Bu), 1.13 – 1.00 (m, 2H, -C*H*$_2$-, Bu), 0.88 (t, J = 7.4 Hz, 3H, -C*H*$_3$, Bu), 0.70 (t, J = 7.4 Hz, 3H, -C*H*$_3$, Bu) ppm; ^{13}C NMR (101 MHz, $CDCl_3$) δ 206.3, 172.3, 165.3, 165.0, 133.8, 133.6, 133.3, 129.9, 129.8, 129.7, 128.9, 128.8, 128.7, 128.5, 128.3, 94.8, 70.3, 69.7, 69.6, 69.1, 68.4, 68.4, 68.3, 68.3, 66.0, 62.4, 37.8, 32.3, 32.3, 32.2, 32.2, 29.8, 27.7, 18.6, 13.6 ppm; IR (neat) ν_{max}: 1736, 1452, 1262, 1258, 1092, 1026 cm^{-1}; ESI-HRMS: m/z [M+Na]$^+$ calcd. for $C_{50}H_{51}O_{14}PNa$: 929.2909 found 929.2792.

^{1}H × NMR

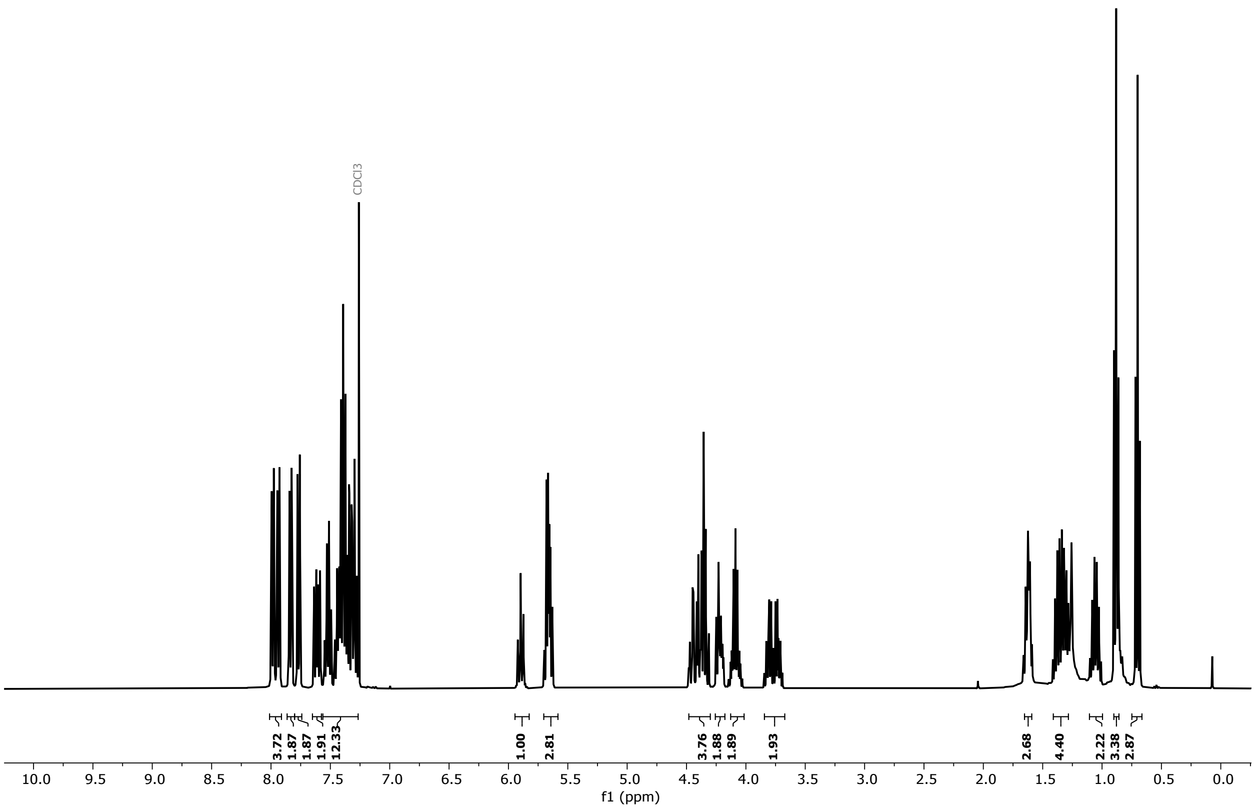

^{13}C × NMR

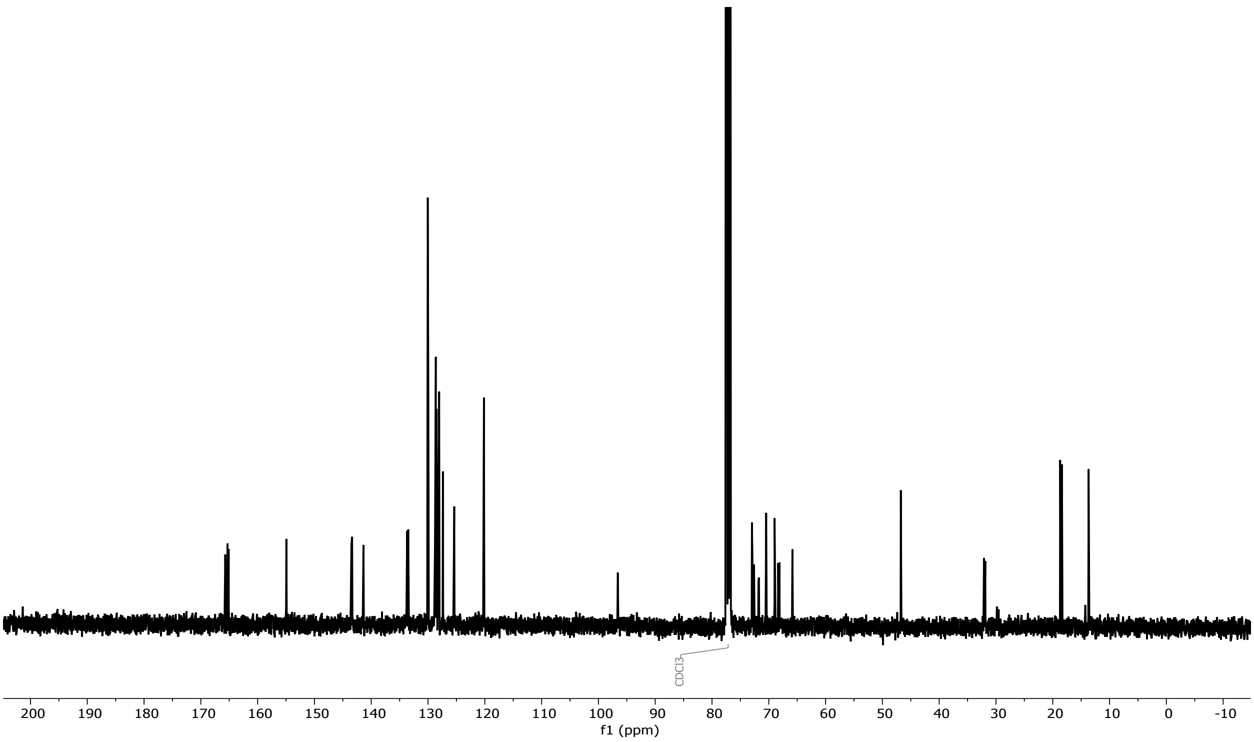

HSQC × NMR

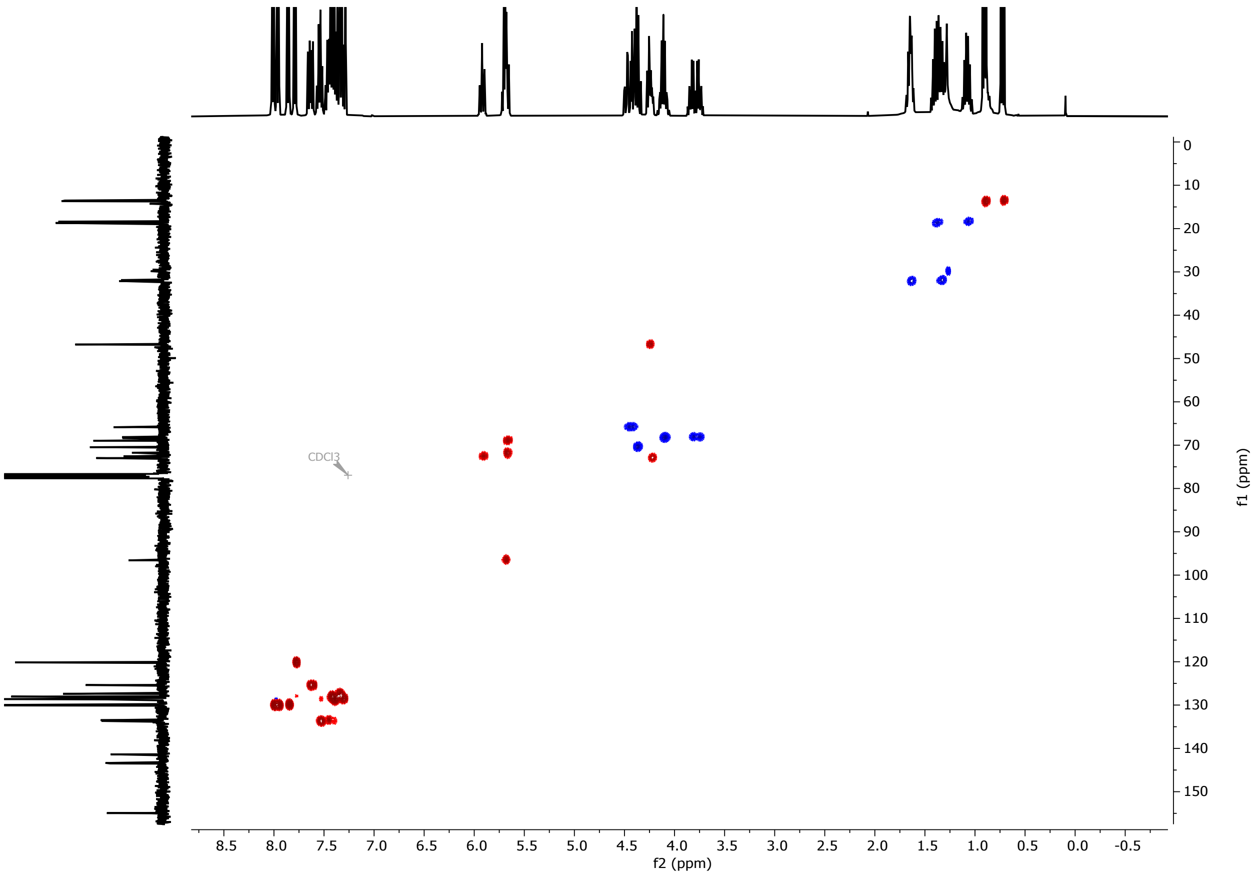

C. Synthesis of base-labile linker (28)

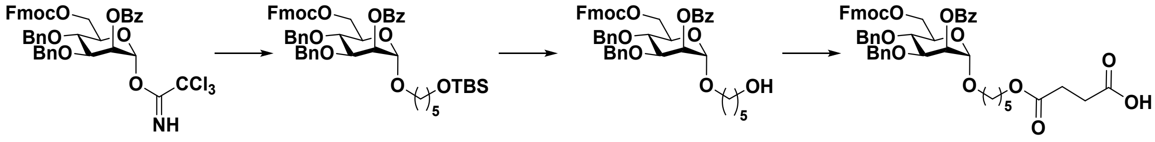

*Synthesis of 4-tert-butyldimethylylsilyloxypent-1-yl-2-O-benzoyl-3,4-di-O-benzyl-6-O-(9-fluorenylmethoxycarbonyl)-α-D-mannopyranoside (**28.1**)*

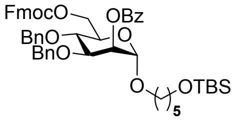

28.1

2-*O*-Benzoyl-3,4-di-*O*-benzyl-6-*O*-(9-fluorenylmethoxycarbonyl)-α-D-mannopyranosyl trichloroacetimidate (**5**) (2.33 g, 2.80 mmol, 1.16 equiv.) was dissolved in anhydr. DCM (20 mL) under argon atmosphere in the presence of 4 Å molecular sieves (powder) and 5-(tert-butyldimethylsilyloxy)-1-pentanol (0.83 mL, 3.38 mmol, 1.40 equiv.) was added. The mixture was cooled down to –78°C and stirred for 15 min. TMSOTf (0.04 mL, 0.24 mmol, 0.10 equiv.) was added and stirring was continued for further 10 min at –78°C. After that the mixture was allowed to warm over 1 h to 0°C. The molecular sieves were filtered off and the reaction was quenched by adding $NaHCO_3$-solution and the organic layer was washed with NaCl-solution (80 mL). The organic layer was dried over $MgSO_4$ and the solvent was removed under reduced pressure. The crude product was purified by flash column chromatography using a mixture of hexane/ethyl acetate (3:1) as eluent. The product was obtained as a white foam in 56% yield (1200 mg, 1.35 mmol). ^{1}H NMR (400 MHz, $CDCl_3$): δ = 8.10 – 7.17 (m, 23H, -*Ar*), 5.59 (dd, *J* = 4.2, 2.1 Hz, 1H, *H*-2), 4.91 (d, *J* = 1.9 Hz, 1H, *H*-1), 4.89 (d, *J* = 10.8 Hz, 1H, -C*H*H- Bn), 4.78 (d, *J* = 11.2 Hz, 1H, -C*H*H- Bn), 4.56 (m, 2H, -C*H*H- Bn, -C*H*H- Bn), 4.45 (d, *J* = 2.8 Hz, 2H, *H*-6α, *H*-6β), 4.36 (d, *J* = 7.6 Hz, 2H, -CH_2-CH- Fmoc), 4.23 (t, *J* = 7.6 Hz, 1H, -C*H*- Fmoc), 4.11 (dd, *J* = 8.6, 3.1 Hz, 1H, *H*-3), 3.97 – 3.86 (m, 2H, *H*-4, *H*-5), 3.68 (dt, *J* = 9.4, 6.6 Hz, 1H, -C*H*H-OSi), 3.57 (t, *J* = 6.4 Hz, 2H, -O-CH_2-), 3.42 (dt, *J* = 9.5, 6.5 Hz, 1H, -C*H*H-OSi), 1.62 – 1.46 (m, 4H; -CH_2-CH$_2$-CH_2-), 1.35 (ddt, *J* = 14.9, 8.3, 4.8 Hz, 2H; -CH_2-), 0.85 (s, 9H; -C$(CH_3)_3$, TBS), 0.01 (s, 6H, -Si$(CH_3)_2$, TBS) ppm; ^{13}C NMR (101 MHz, $CDCl_3$): δ = 165.7, 155.2, 143.4, 143.3, 141.2, 137.9, 137.8, 133.3, 129.9, 129.8, 128.5, 128.4, 128.3, 128.3, 128.1, 128.1, 127.9, 127.9, 127.7, 127.2, 125.2, 125.2, 120.1, 97.7, 78.4, 75.3, 73.8, 73.8, 71.5, 71.5, 70.0, 69.7, 68.8, 68.2, 66.8, 63.0, 46.7, 32.6, 29.2, 22.5, 18.4 ppm; IR (neat) ν_{max}: 2931, 2355, 1730, 1452, 1259, 1098 cm^{-1}; ESI-HRMS: m/z $[M+Na]^+$ calcd. for $C_{53}H_{62}O_{10}SiNa$: 909.4004 found 909.4043.

^{1}H NMR ($CDCl_3$)

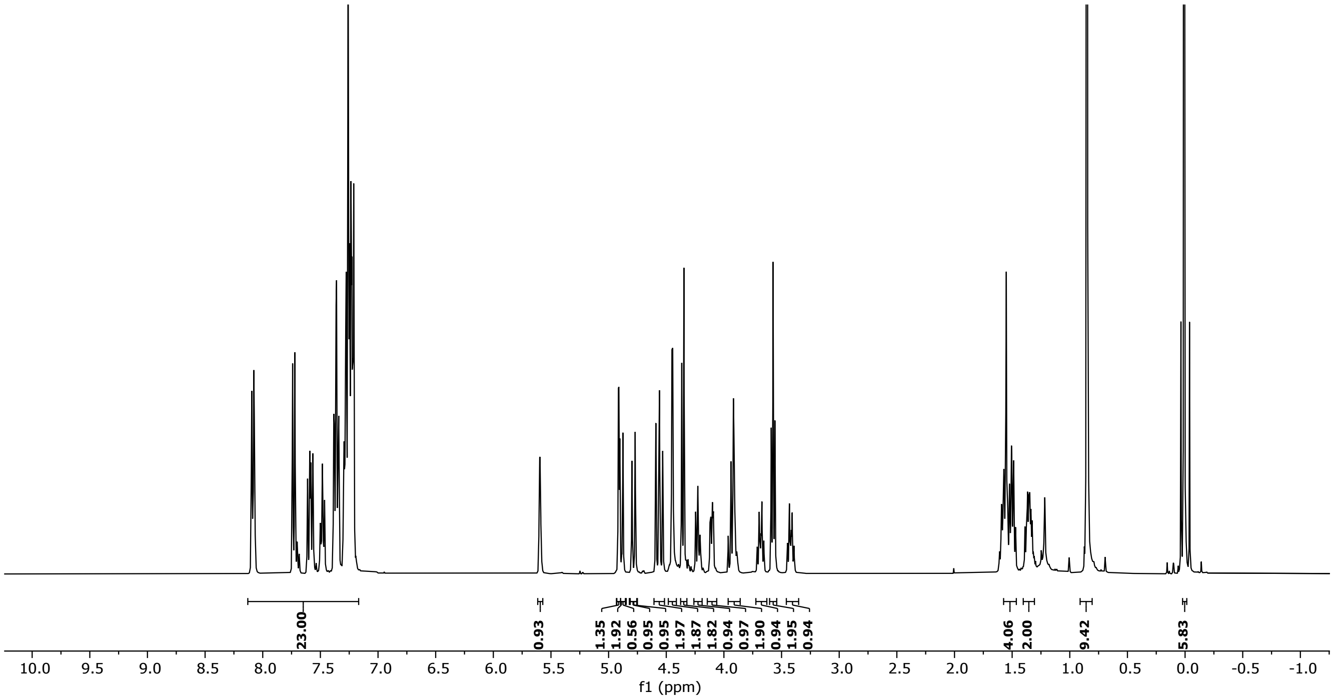

^{13}C NMR ($CDCl_3$)

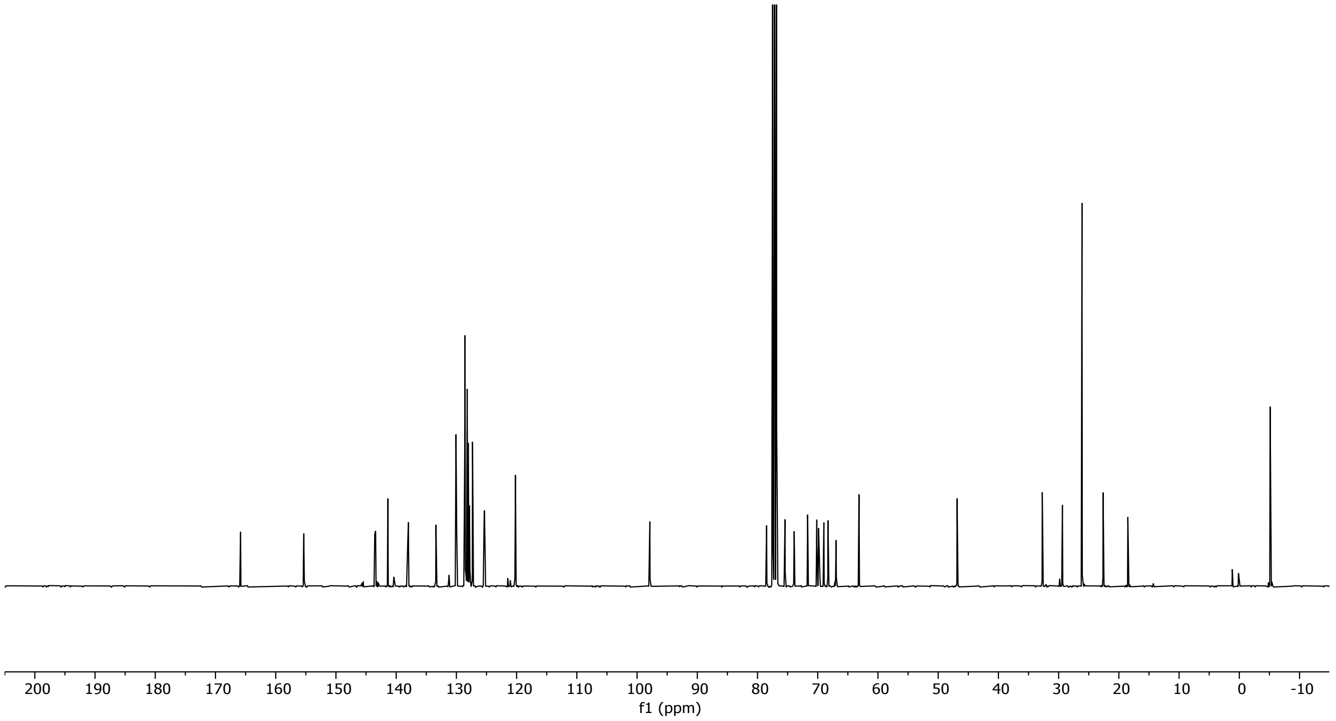

HSQC NMR ($CDCl_3$)

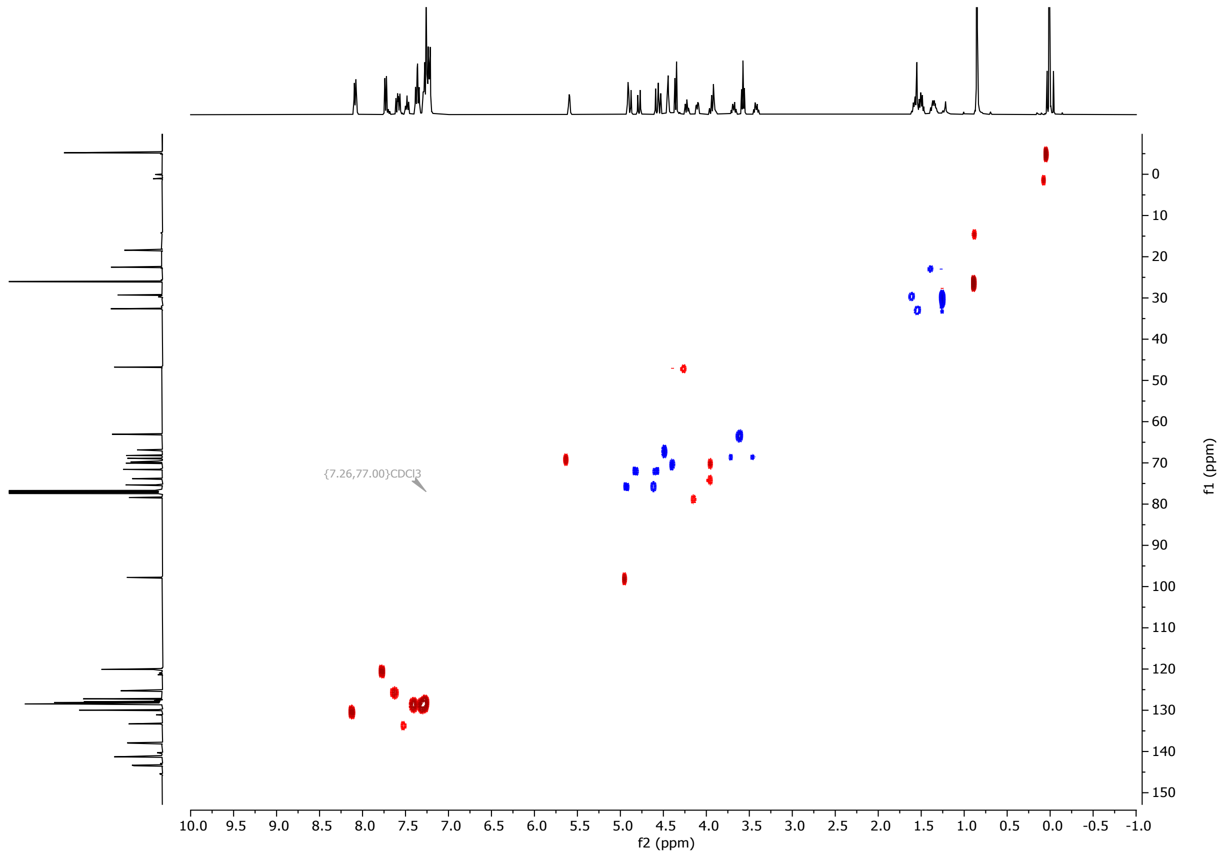

*Synthesis of 5-hydroxypent-1-yl-2-O-benzoyl-3,4-di-O-benzyl-6-O-(9-fluorenylmethoxycarbonyl)-α-D-mannopyranoside (**28.2**)*

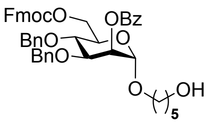

28.2

To a stirred solution of **28.1** (1.16 g, 1.31 mmol, 1.00 equiv.) in anhydr. MeOH (20 mL) was added acetyl chloride (AcCl) (0.20 mL, 0.28 mmol, 0.22 equiv.), and the reaction mixture was stirred for 1 h at rt. Then, DCM was added (50 mL), and the reaction was neutralized with $NaHCO_3$-solution (50 mL). The aqueous phase was extracted with DCM (50 mL) and washed with water (70 mL). The combined organic layer was dried over Na_2SO_4 and the solvent was removed under reduced pressure. The crude product was purified by flash column chromatography using a mixture of hexane/ethyl acetate (3:1) as eluent. The product was obtained as a white foam in 98% of yield (989 mg, 1.28 mmol). ^1H NMR (400 MHz, $CDCl_3$): δ = 8.14 – 7.25 (m, 23H, *-Ar)*, 5.62 (dd, *J* = 3.2, 1.9 Hz, 1H, *H*-2), 4.97 – 4.91 (m, 2H, *H*-1, -C*H*H- Bn), 4.82 (d, *J* = 11.2 Hz, 1H, -C*H*H- Bn), 4.60 (t, *J* = 10.8 Hz, 2H, -C*H*H- Bn, -C*H*H- Bn), 4.52 – 4.45 (m, 2H, *H*-6α, *H*-6β), 4.40 (d, *J* = 7.5 Hz, 2H, -CH_2-CH- Fmoc), 4.27 (t, *J* = 7.5 Hz, 1H, -C*H*- Fmoc), 4.14 (m, 1H, *H*-3), 3.99 – 3.92 (m, 2H, *H*-4, *H*-5), 3.73 (dt, *J* = 9.7, 6.5 Hz, 1H, -C*H*H-OH), 3.64 (t, *J* = 6.5 Hz, 2H, -O-CH_2-), 3.47 (dt, *J* = 9.7, 6.3 Hz, 1H, -C*H*H-OH), 1.66 – 1.52 (m, 4H, -CH_2-CH_2-CH_2-), 1.43 (qd, *J* = 7.7, 5.5 Hz, 2H, -CH_2-), ppm; ^{13}C NMR (101 MHz, $CDCl_3$): δ = 165.7, 155.2, 143.4, 143.3, 141.2, 137.9, 137.8, 133.3, 129.9, 129.8, 128.5, 128.4, 128.3, 128.3, 128.1, 128.1, 127.9, 127.9, 127.7, 127.2, 125.2, 125.2, 120.1, 97.7, 78.4, 75.3, 73.8, 73.8, 71.5, 71.5, 70.0, 69.7, 68.8, 68.2, 66.8, 63.0, 46.7, 32.6, 29.2, 22.5, 18.4 ppm; IR (neat) ν_{max}: 2982, 1723, 1452, 1263, 1073 cm^{-1}; ESI-HRMS: m/z $[M+Na]^+$ calcd. for $C_{47}H_{48}O_{10}Na$: 795.3140 found 795.3163.

^1H NMR ($CDCl_3$)

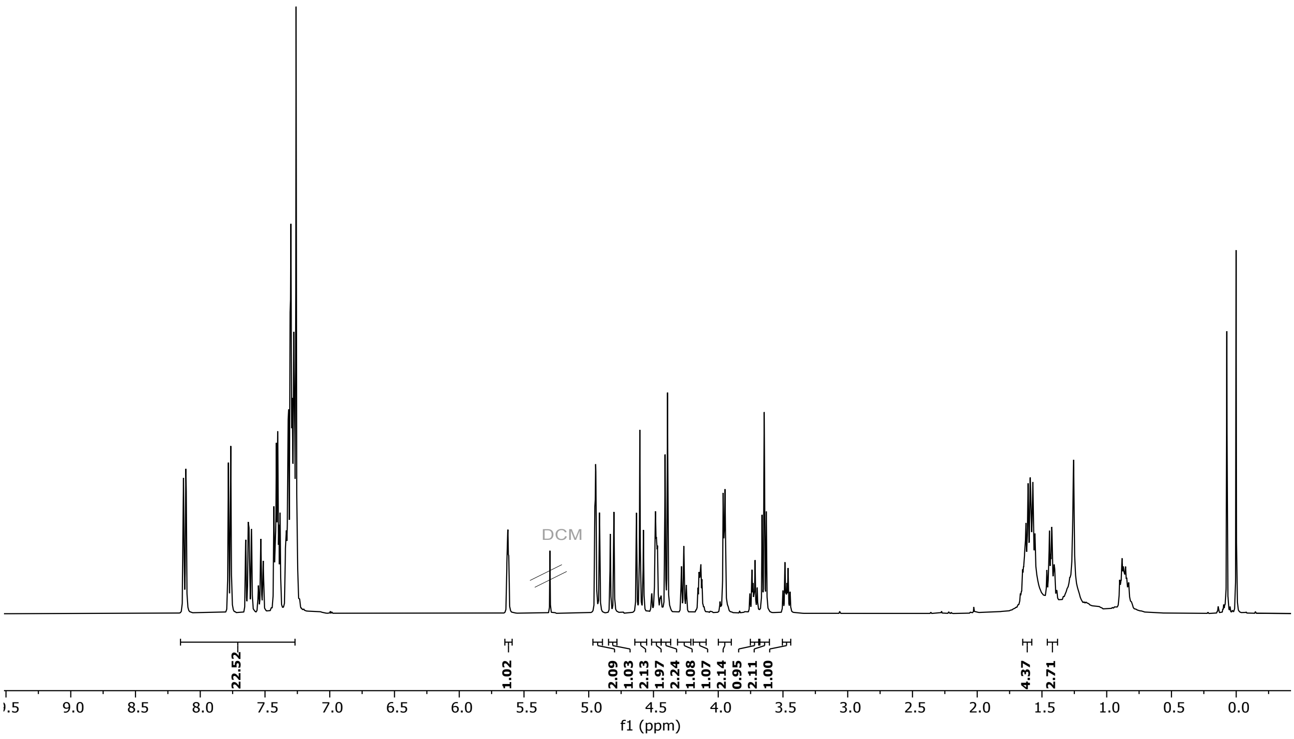

^{13}C NMR ($CDCl_3$)

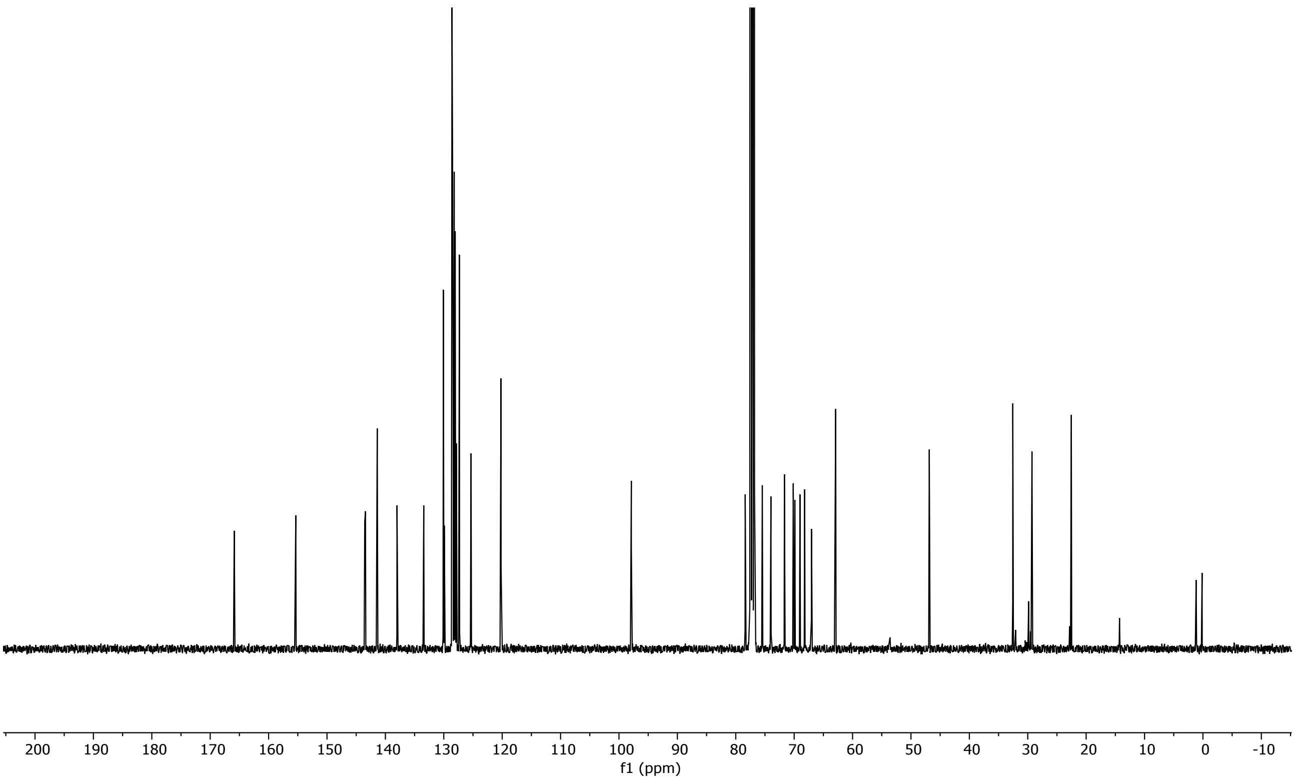

HSQC NMR ($CDCl_3$)

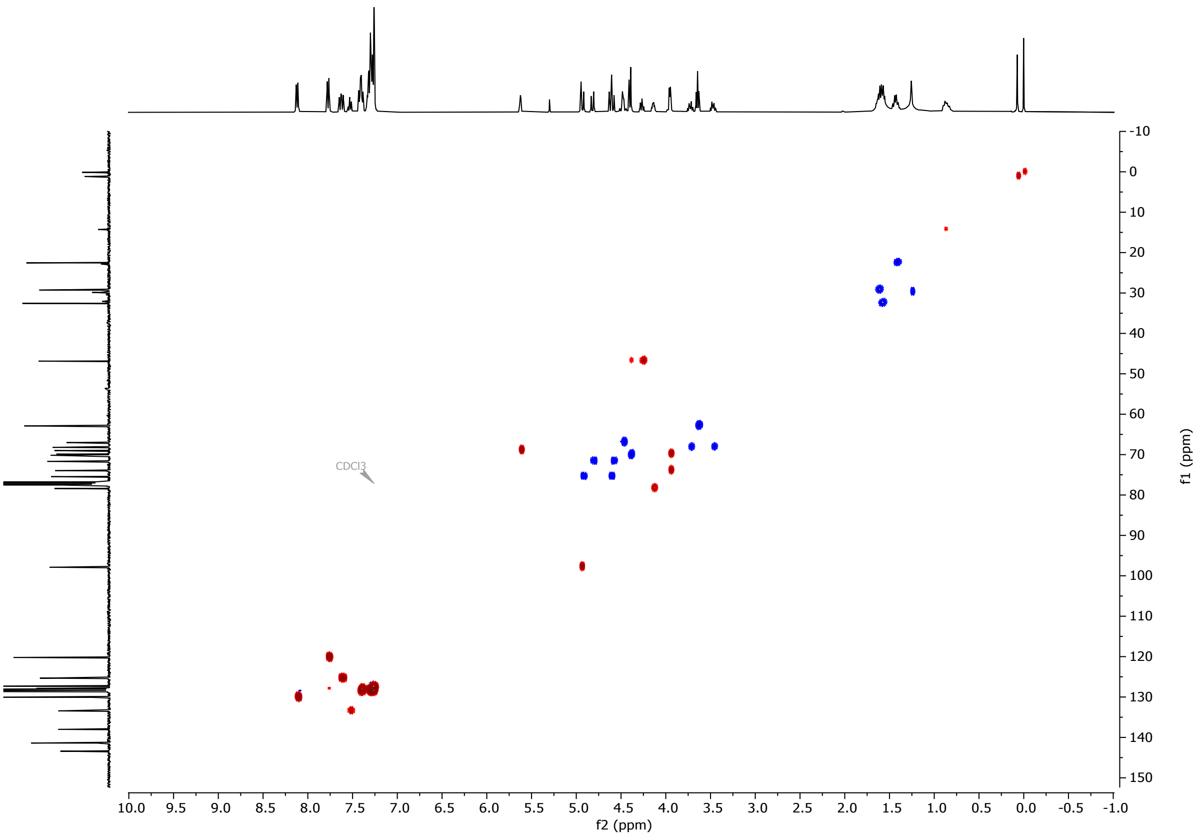

*Synthesis of 4-succinoyloxypent-1-yl-2-O-benzoyl-3,4-di-O-benzyl-6-O-(9-fluorenylmethoxycarbonyl)-α-D-mannopyranoside (**28**)*

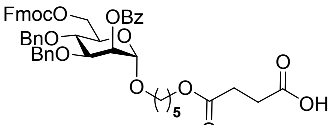

28

To a stirred solution of **28.2** (989 mg, 1.28 mmol, 1.00 equiv.) in pyridine (4 mL) were added succinic anhydride (384 mg, 3.84 mmol, 3.00 equiv.) and stirred at 65°C overnight under argon atmosphere. After conversion, solvents were removed under reduced pressure, and the residue was co-evaporated with toluene (3 × 10 mL). The crude product was purified by flash column chromatography using a mixture of hexane/ethyl acetate (1:1, with 0.5% of acetic acid) as eluent. The product was obtained as a white foam in 79% yield (885 mg, 1.02 mmol, 79%). ^{1}H NMR (400 MHz, $CDCl_3$): δ = 8.14 – 7.20 (m, 23H, -*Ar*), 5.68 (dd, *J* = 3.2, 1.9 Hz, 1H, *H*-2), 4.96 – 4.90 (m, 3H, *H*-1, -CHH- Bn), 4.82 (d, *J* = 11.1 Hz, 1H, -CHH- Bn), 4.64 (d, *J* = 10.9 Hz, 1H, -CHH- Bn), 4.60 (d, *J* = 11.1 Hz, 1H, -CHH- Bn), 4.47 (m, 2H, *H*-6α, *H*-6β), 4.40 (d, *J* = 1.5 Hz, 2H, -CH_2-CH-Fmoc), 4.27 (d, *J* = 7.5 Hz, 1H, -C*H*- Fmoc), 4.23 – 4.18 (m, 1H, *H*-3), 4.18 – 4.09 (m, 2H, -CH_2-O-CO), 4.03 – 3.96 (m, 2H, *H*-4, *H*-5), 3.72 (ddd, *J* = 9.6, 7.1, 5.3 Hz, 1H, -O-CH_2-CH_2-), 3.47 (dt, *J* = 9.6, 5.7 Hz, 1H, -O-CH_2-CH_2-), 2.66 – 2.55 (m, 4H, -CO-CH_2-CH_2-COOH), 1.64 (tp, *J* = 17.7, 5.9 Hz,4H, -CH_2-CH_2-CH_2-), 1.45 (ddt, *J* = 15.4, 13.5, 7.7 Hz, 2H, -CH_2-CH_2-CH_2-) ppm; ^{13}C NMR (101 MHz, $CDCl_3$): δ = 174.8, 172.4, 166.0, 155.3, 143.5, 143.4, 141.4, 137.9, 137.5, 133.5, 130.1, 129.8, 129.1, 128.7, 128.6, 128.6, 128.4, 128.3, 128.2, 128.2, 128.0, 127.8, 127.3, 125.3, 125.3, 120.2, 97.9, 78.2, 75.5, 73.9, 71.6, 70.1, 69.6, 69.1, 68.2, 66.8, 64.7, 46.8, 29.2, 28.6, 23.1 ppm; IR (neat) ν_{max}: 2982, 1724, 1386, 1255, 1154 cm^{-1}; ESI-HRMS: m/z [M+Na]+ calcd. for $C_{51}H_{52}O_{13}Na$: 895.3300 found 895.3337.

^{1}H NMR ($CDCl_3$)

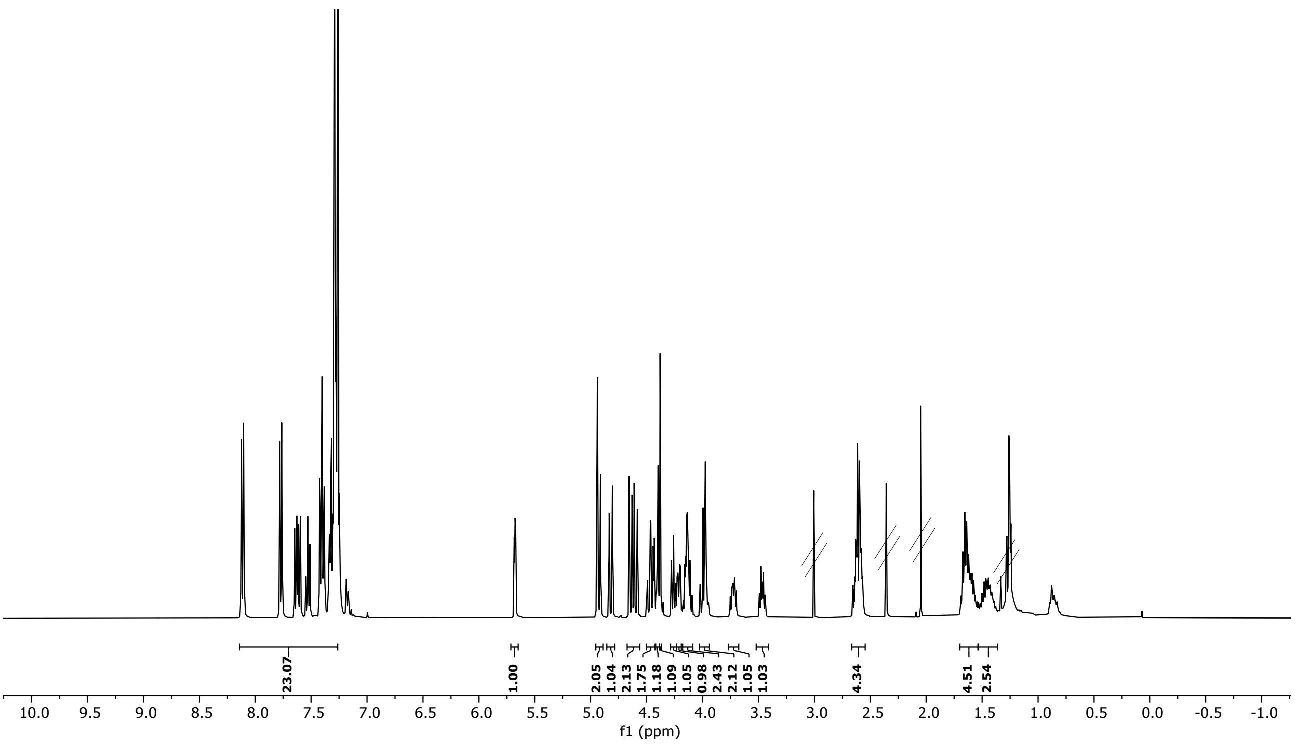

^{13}C NMR ($CDCl_3$)

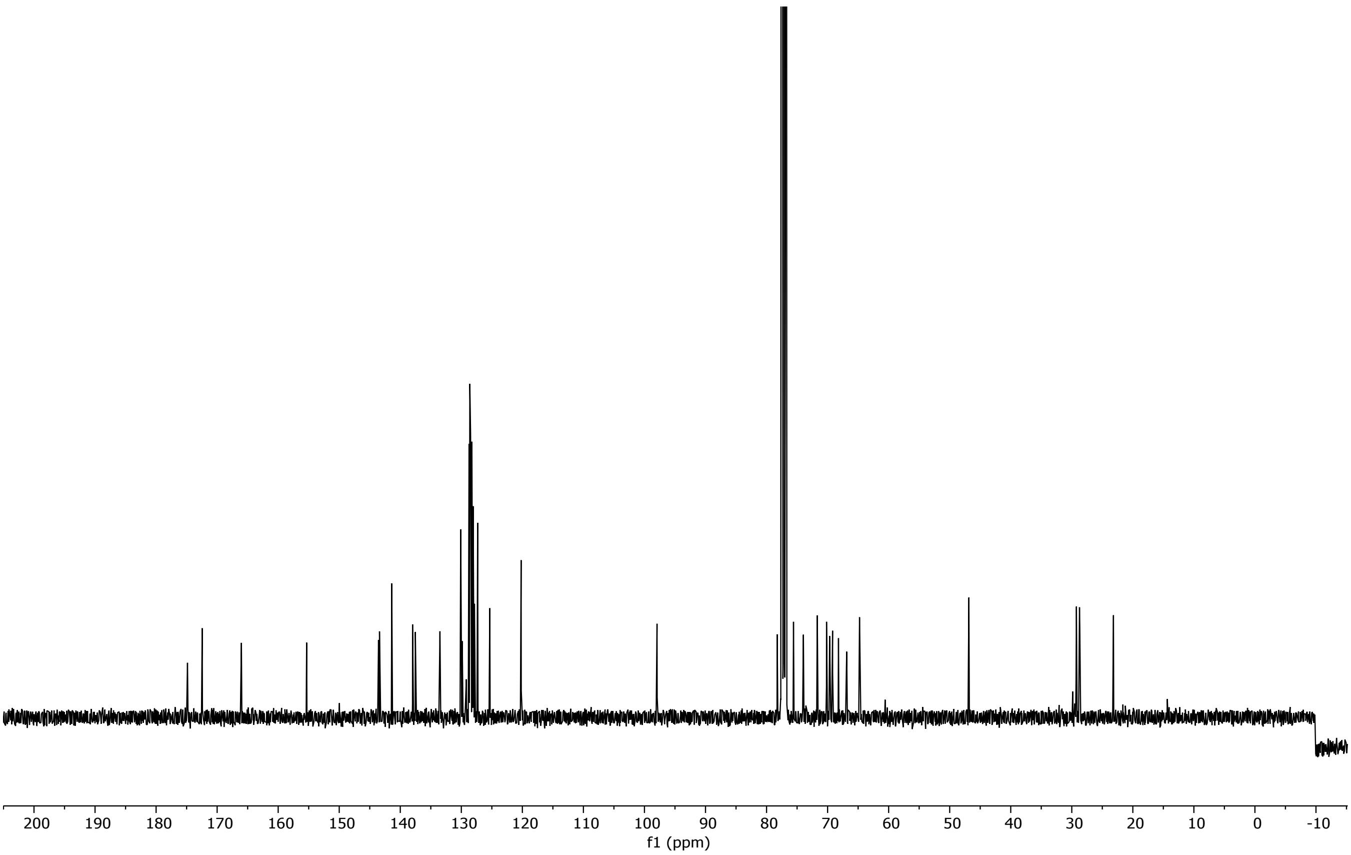

HSQC NMR ($CDCl_3$)

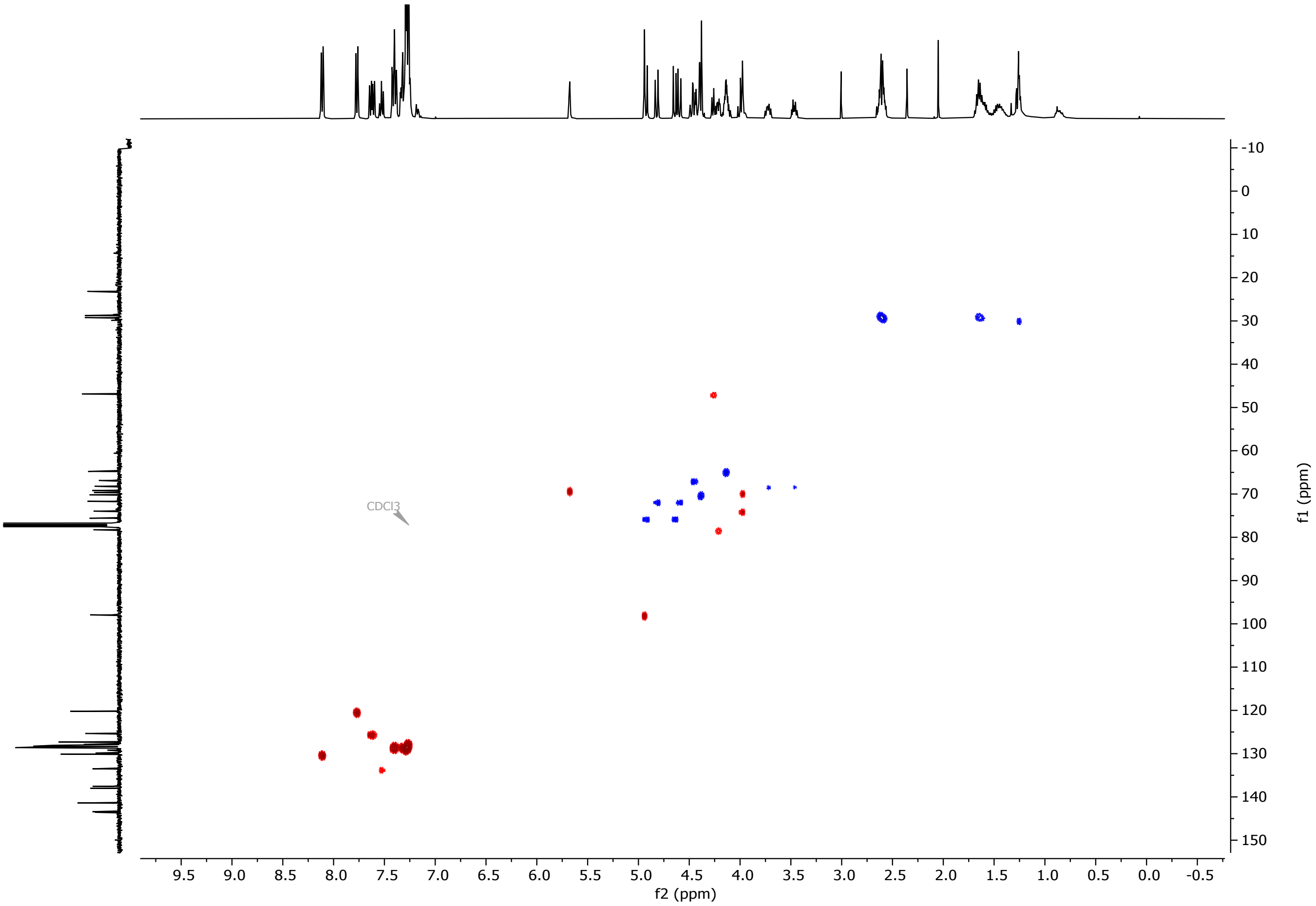

D. Synthesis of photo-labile linker (29)

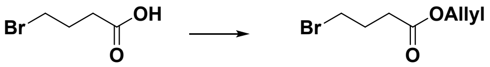

29.1 29.2

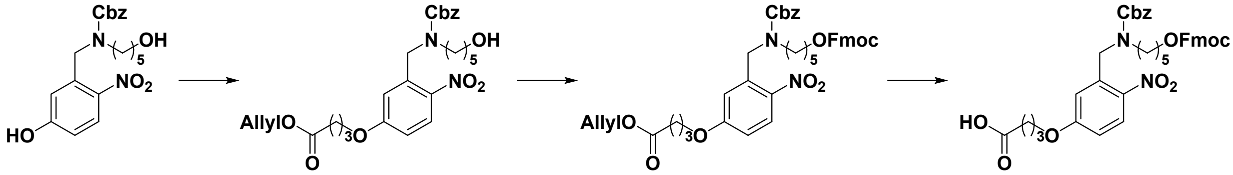

29.3 29.4 29.5 29

*Synthesis of allyl-bromobutanoate (**29.2**)*

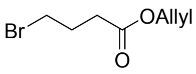

29.2

A solution of allyl alcohol (0.41 mL, 5.98 mmol, 1.00 equiv.), 4-bromobutanoic acid **29.1** (1.00 g, 5.98 mmol, 1.00 equiv.), and DMAP (92.0 mg, 0.75 mmol, 0.25 equiv.) in anhydr. DCM (60 mL) was stirred at 0°C under argon atmosphere for 30 min. Then *N,N'*-diisopropylcarbodiimide (DIC) (1.70 mL, 10.8 mmol, 1.80 equiv) was added dropwise. The reaction mixture was slowly left to warm up to rt overnight. Then, the reaction mixture was filtrated to get rid of the formed solid. The residue was diluted in cold hexane, and then filtered on a pad of silica gel. The pad of silica gel was washed with hexane/ethyl acetate (4.5:1) and the solvents were removed under reduced pressure giving the desired compound as a yellow oil in 79% yield (971 mg, 4.69 mmol). ^1H NMR (400 MHz, $CDCl_3$): δ = 5.91 (m, 1H, -

C*H*=CH$_2$), 5.32 (dd, *J* = 17.2 Hz, 1H, -CH=C*H*$_2$), 5.24 (dd, *J* = 10.4 Hz, 1H, -CH=C*H*$_2$), 4.59 (dt, *J* = 5.8, 1.4 Hz, 2H, -O-C*H*$_2$-), 3.47 (t, *J* = 6.4 Hz, 2H, Br-C*H*$_2$-), 2.53 (t, *J* = 7.2 Hz, 2H, -CO-C*H*$_2$-), 2.13 – 2.21 (m, 2H, -C*H*$_2$-) ppm; ^{13}C NMR (101 MHz, CDCl$_3$): δ = 172.1, 131.9, 118.3, 65.2, 32.6, 32.3, 27.6 ppm.

The analytical data agree with the literature.[13]

*Synthesis of 7-(3-(((5-hydroxypentyl)amino)methyl)-4-nitrophenoxy)hept-1-en-4-one (**29.4**)*

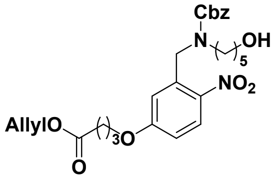

29.4

N-[(5-Hydroxy-2-nitrophenyl)methyl]-N-(5-hydroxypentyl) phenylmethyl ester carbamic acid [14] **29.3** (2.37 g, 4.70 mmol, 1.00 equiv.) and potassium carbonate (K_2CO_3) (1.62 g, 11.7 mmol, 2.50 equiv.) were dissolved in DMF (30 mL) at 60°C. After 1h, allyl 4-bromobutanoate **29.2** (384 mg, 1.86 mmol, 1.00 equiv.) was added dropwise and the solution stirred for 24 h. The reaction mixture was quenched with water and stirred for additional 15 min to reach rt. The resulted mixture was diluted in DCM, and washed with water. The aqua phase was washed with DCM three times. The combined organic phases were washed with NaCl-solution and dried over Na_2SO_4. The residue was purified by flash column chromatography using a mixture of hexane/ethyl acetate (1:1) to afford the desired compound as a yellow oil in 95% yield (2.28 g, 4.44 mmol). ^{1}H NMR (400 MHz, CDCl$_3$): δ = 8.15 (m, 1H, -*Ar*), 7.44 – 7.33 (m, 3H,-*Ar*), 7.20 – 7.11 (m, 1H, -*Ar*), 6.88 – 6.59 (m, 3H, -*Ar*), 5.92 (ddt, *J* = 17.3, 10.4, 5.8 Hz, 1H, -CH$_2$-C*H*=CH$_2$, Allyl), 5.33 (dd, *J* = 17.1, 1.6 Hz, 1H, -CH=C*H*H, Allyl), 5.25 (dd, *J* = 10.4, 1.3 Hz, 1H, -CH=C*H*H, Allyl), 5.15 (d, *J* = 4.2 Hz, 2H, -C*H*$_2$-Ar, Cbz), 4.89 (d, *J* = 4.4 Hz, 2H, Ar-C*H*$_2$-N-), 4.60 (dd, *J* = 5.8, 1.4 Hz, 2H, -C*H*$_2$-CH=CH$_2$), 3.94 (m, 2H, -CH$_2$-C*H*$_2$-O), 3.65 – 3.53 (m, 2H, -C*H*$_2$-OH), 3.31 (m, 2H, -NCbz-C*H*$_2$-), 2.54 (m, 2H, -CO-C*H*$_2$-CH$_2$-), 2.10 (m, 2H, -CO-CH$_2$-C*H*$_2$-), 1.71 – 1.43 (m, 4H, -C*H*$_2$-CH$_2$-C*H*$_2$-), 1.40 – 1.26 (m, 2H, -CH$_2$-C*H*$_2$-CH$_2$-) ppm; ^{13}C NMR (101 MHz, CDCl$_3$): δ = 172.5, 163.2, 156.3, 140.9, 137.7, 136.4, 131.9, 128.5, 128.3, 128.0, 127.9, 118.4, 113.5, 113.0, 112.5, 67.4, 67.1, 65.3, 62.6, 60.3, 48.7, 48.1, 32.2, 30.3, 27.6, 24.1, 22.8 ppm; ESI-LRMS: m/z [M+Na]$^+$ calcd. for $C_{27}H_{34}N_2O_8$ Na: 537.2207 found 537.2.

The compound was used without further characterization.

^{1}H NMR (CDCl$_3$)

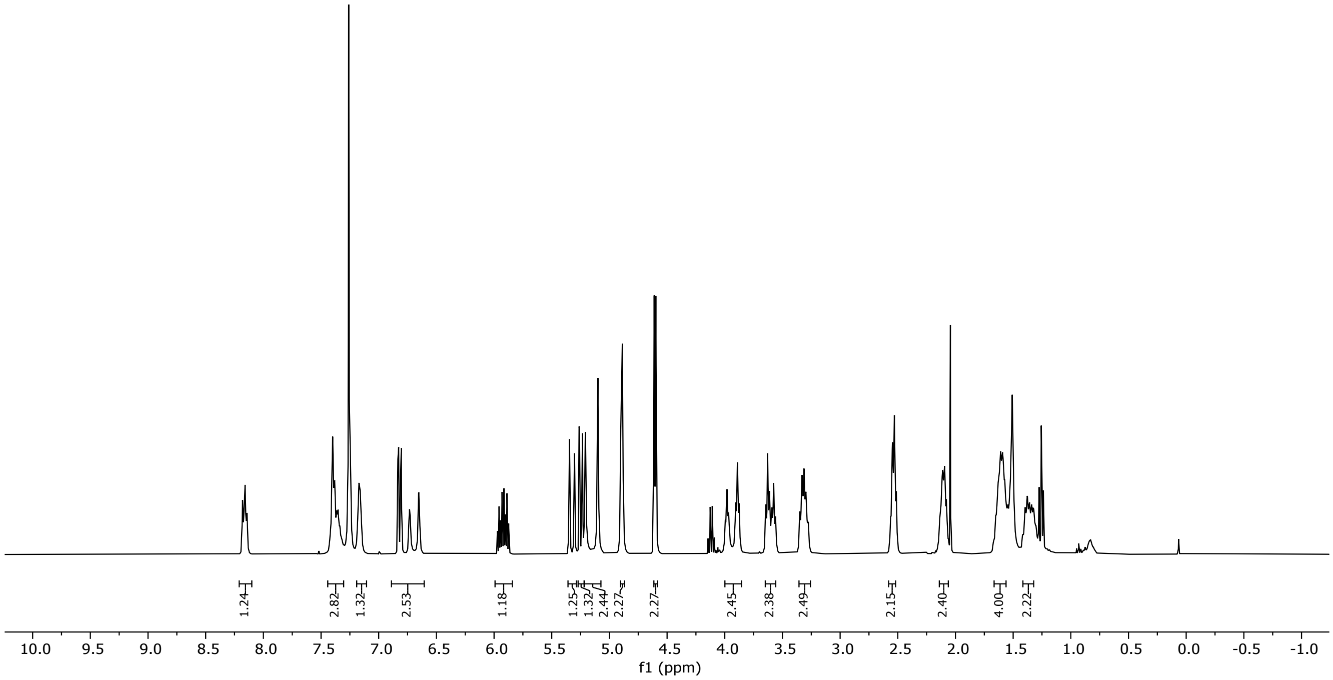

^{13}C NMR ($CDCl_3$)

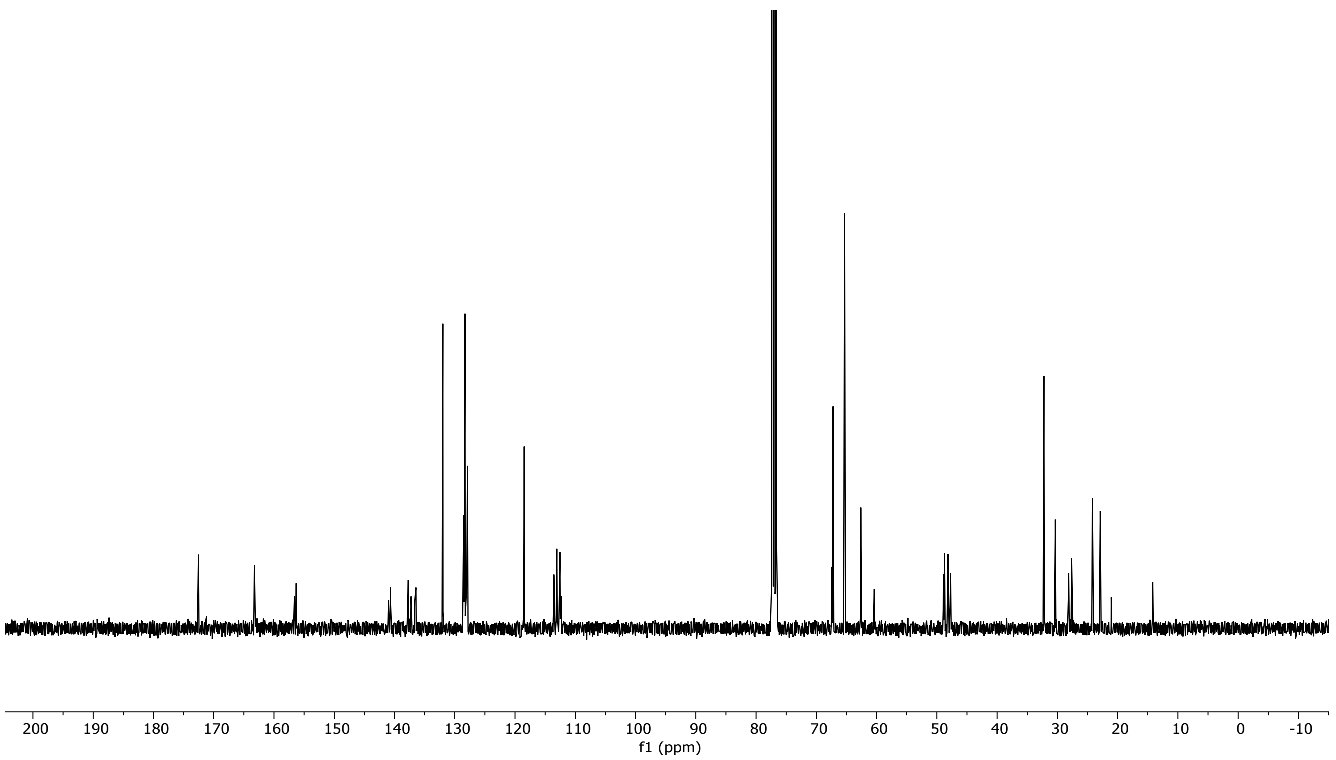

HSQC ($CDCl_3$)

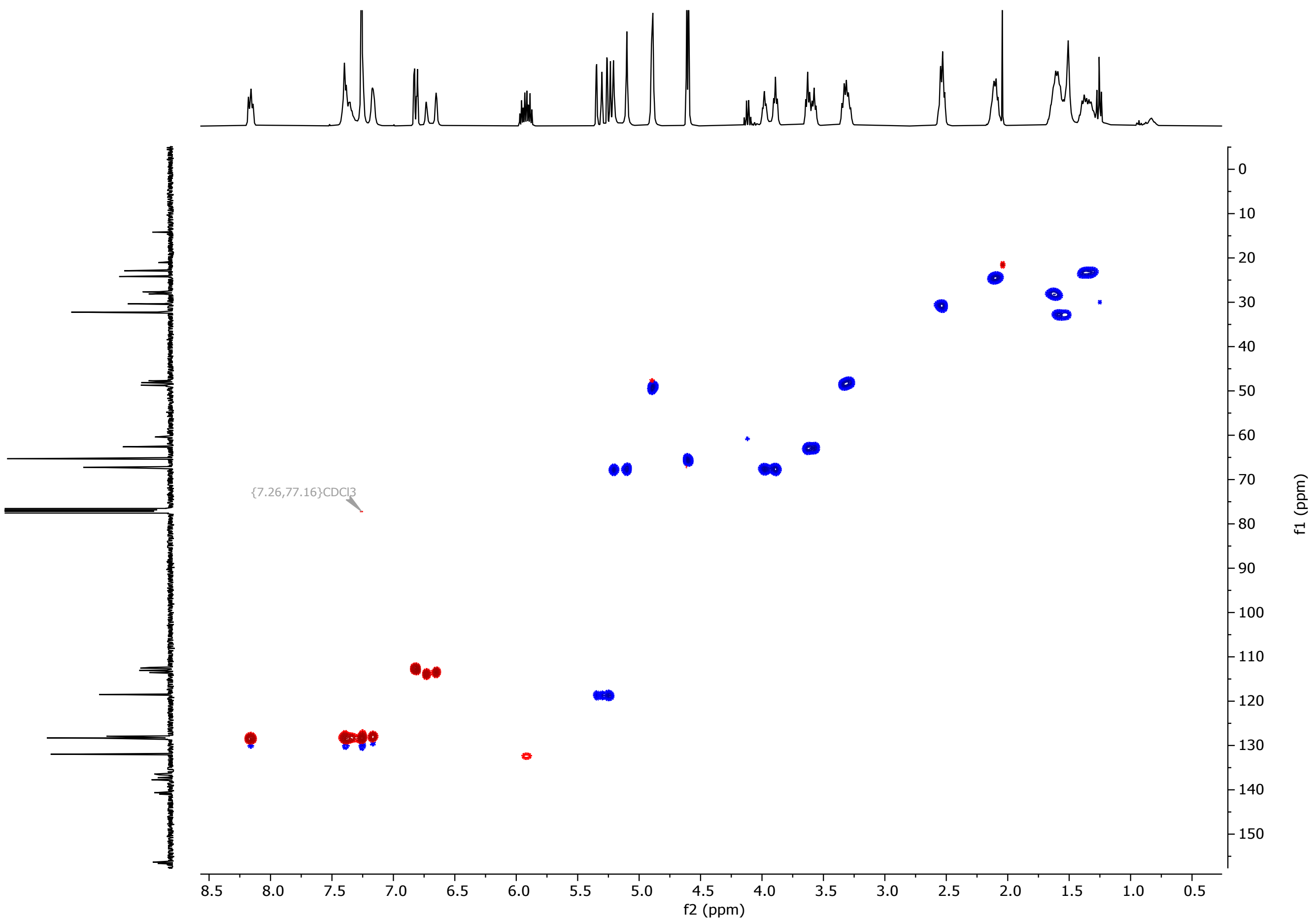

*Synthesis of allyl-4-(3-(((5-((((9-fluorenylmethoxycarbonyl) pentyl) ((benzyloxy) carbonyl) amino) methyl)-4-nitrophenoxy) butanoate (**29.5**)*

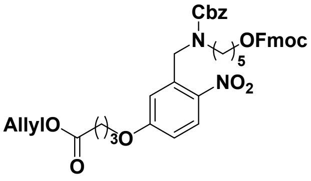

29.5

7-(3-(((5-Hydroxypentyl)amino)methyl)-4-nitrophenoxy)hept-1-en-4-one **29.3** (2.28 g, 4.43 mmol, 1.00 equiv.) was dissolved in anhydr. DCM (50 mL) and pyridine (2.50 mL, 10.0 mmol, 7.00 equiv.) and FmocCl (2.30 mg, 8.86 mmol, 2.00 equiv.) were added consecutively. The solution was stirred overnight at rt, and then the solvent was removed under reduced pressure. Residual pyridine was coevaporated with toluene. The crude product was purified by flash column chromatography using a mixture of hexane/ethyl acetate (2:1) as eluent. The product was obtained as a yellow oil in 99% yield (3.22 g, 4.37 mmol).^{1}H NMR (400 MHz, $CDCl_3$):): δ = 8.17 (m, 1H, -*Ar*), 7.76 (dt, *J* = 7.6, 0.9 Hz, 2H, -*Ar*), 7.66 – 7.58 (m, 2H, -*Ar*), 7.45 – 7.28 (m, 8H, -*Ar*), 7.18 (m, 1H, -*Ar*), 6.82 (dd, *J* = 9.1, 2.7 Hz, 1H, -*Ar*), 6.76 – 6.62 (m, 1H, -*Ar*), 6.00 – 5.83 (m, 1H, -C*H*=CH$_2$, Allyl), 5.37 – 5.24 (m, 2H, , -CH=C*H$_2$*, Allyl), 5.10 (d, *J* = 4.2 Hz, 2H, -CH$_2$-, Cbz), 4.90 (d, *J* = 4.5 Hz, 2H, Ar-C*H$_2$*-N-), 4.60 (dd *J* = 5.8, 1.4 Hz, 2H, -C*H$_2$*-CH=CH$_2$), 4.40 (d, *J* = 7.6 Hz, 2H, -C*H$_2$*-*O*Fmoc), 4.26 (t, *J* = 7.4 Hz, 1H; -C*H*-; Fmoc), 4.20 – 4.07 (m, 2H, -C*H$_2$*-; Fmoc), 3.94 (dt, *J* = 34.5, 6.0 Hz, 1H, -CH$_2$-C*H$_2$*-O-Ar), 3.32 (m, 2H, -NCbz-C*H$_2$*-CH$_2$-), 2.54 (m, 2H, -CO-C*H$_2$*-CH$_2$-), 2.10 (, 2H, -CO- CH$_2$-C*H$_2$*-), 1.77 – 1.64 (m, 4H, -C*H$_2$*-CH$_2$-C*H$_2$*-), 1.46 – 1.29 (m, 2H, -CH$_2$-C*H$_2$*-CH$_2$-); 13C NMR (101 MHz, CDCl3): δ = 172.6, 155.3, 143.5, 141.4, 132.1, 128.5, 127.9, 127.2, 125.3, 120.1, 118.6, 113.7, 113.2, 112.6, 69.8, 68.0, 67.6, 67.4, 65.4, 60.5, 53.5, 48.8, 48.2, 46.8, 30.5, 28.1, 24.3, 23.1 ppm; ESI-LRMS: m/z [M+Na]$^+$ calcd. for $C_{42}H_{34}N_2O_8$ Na: 759.28 found 759.2.

∗The compound was used without further characterization.

^{1}H NMR ($CDCl_3$)

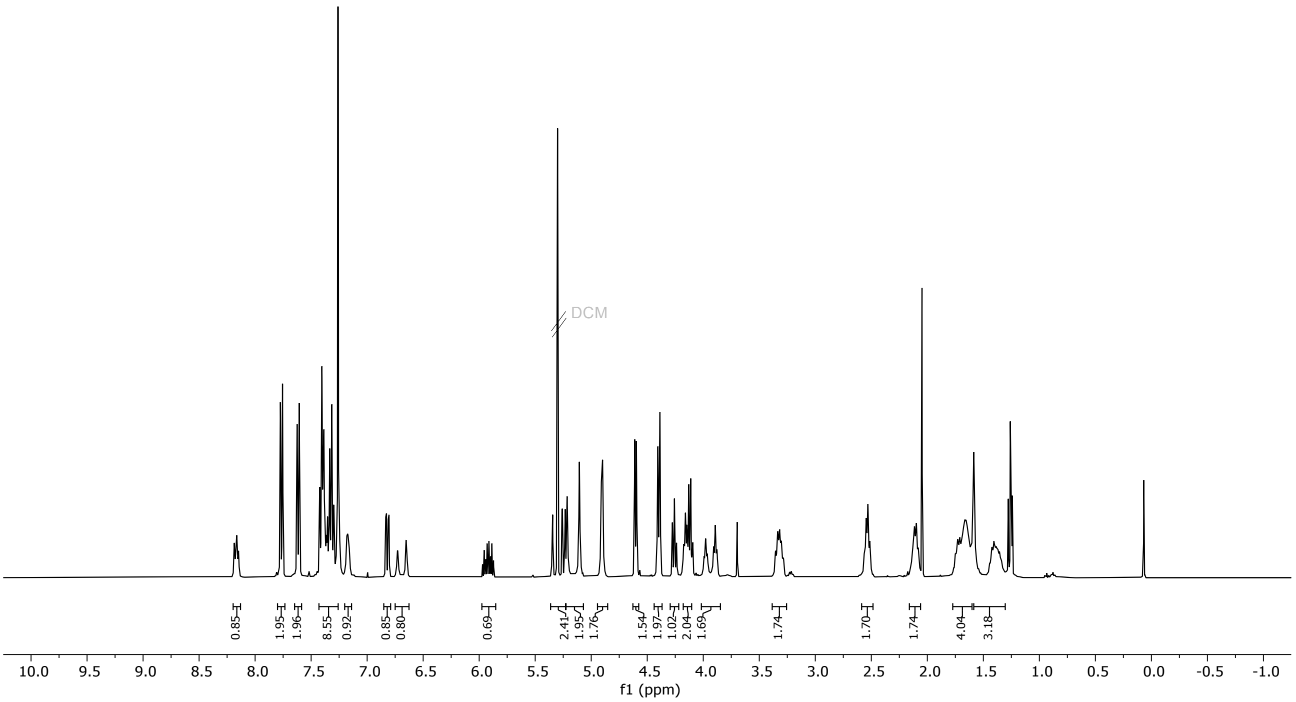

^{13}C NMR ($CDCl_3$)

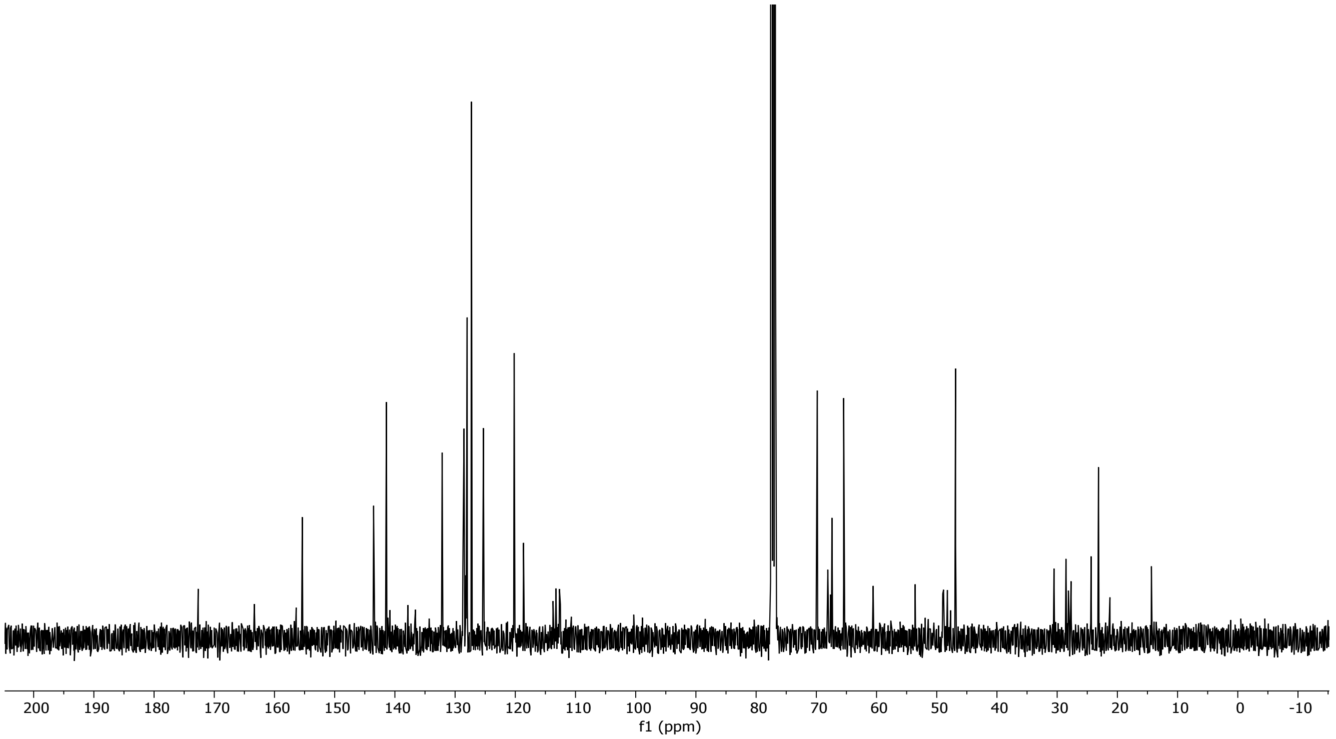

HSQC NMR ($CDCl_3$)

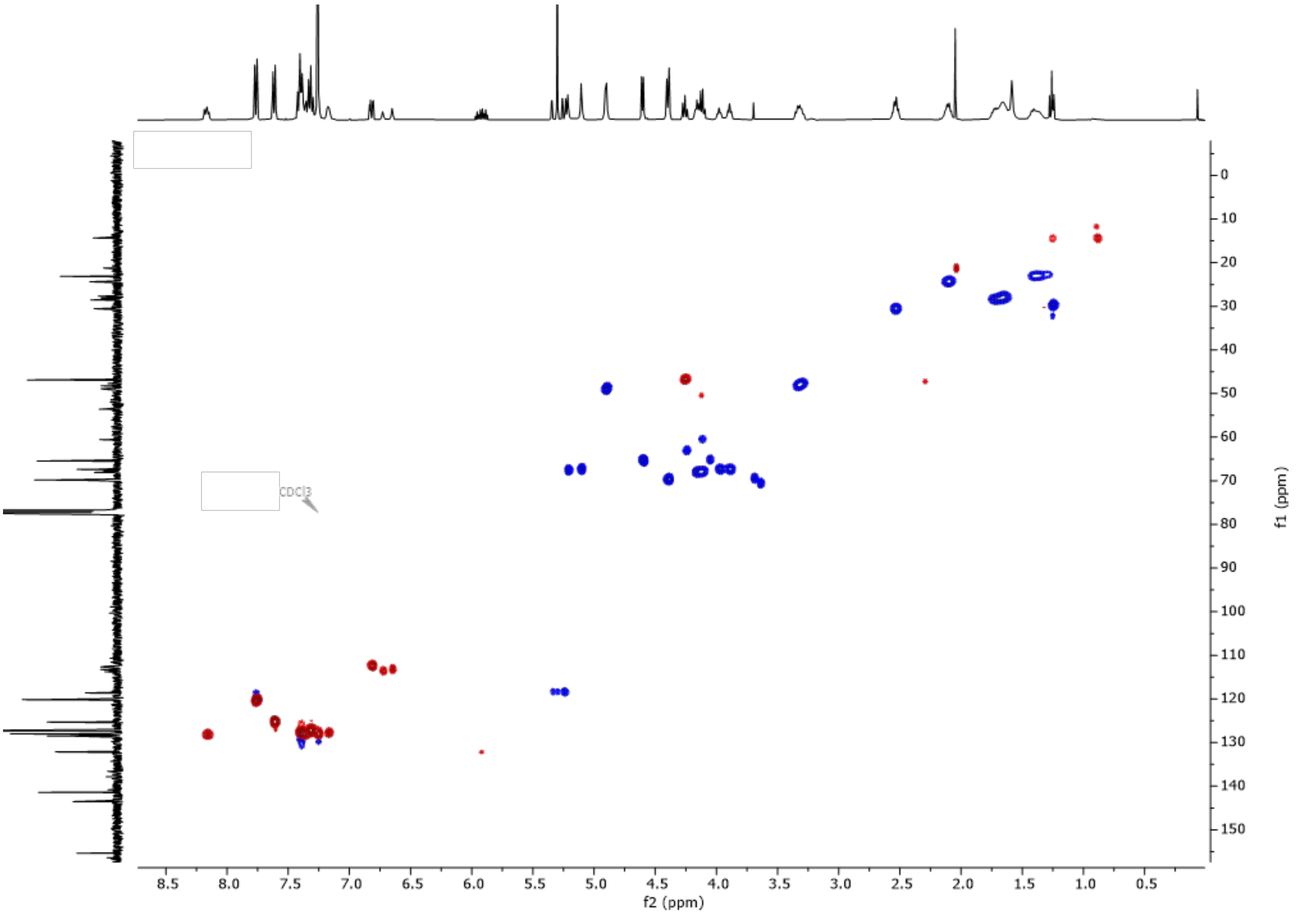

*4-(3-(((5-((((-(9-fluorenylmethoxycarbonyl)-pentyl) ((benzyloxy) carbonyl) amino) methyl)-4-nitrophenoxy) butanoic acid (**29**)*

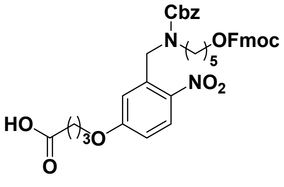

29

Allyl-4-(3-(((5-((((9H-fluoren-9yl) methoxy) carbonyl) oxy) pentyl) ((benzyloxy) carbonyl) amino)methyl)-4-nitrophenoxy)butanoate **29.4** (3.22 g, 4.38 mmol, 1.00 equiv.) was dissolved in anhydr. tetrahydrofuran (THF) (138 mL) under argon atmosphere and $Pd(PPh_3)_4$ (557 mg, 0.48 mmol, 0.11 equiv.) and acetic acid (20.4 mL) were added consecutively. The solution was stirred for 4 h at rt, and then, diluted with DCM (200 mL). Then the mixture was washed with 0.5 M aqueous hydrochloric acid-solution (200 mL), the organic layer was dried over Na_2SO_4 and the solvent was removed under reduced pressure. The crude product was purified by flash column chromatography using a mixture of hexane/ethyl acetate (1:1 with 0.5% of acetic acid) as eluent. The product was obtained as a yellow oil in 83% yield (2.53 g, 3.63 mmol). ^{1}H NMR (400 MHz, $CDCl_3$): δ = 8.17 (m, 1H, *-Ar*), 7.76 (dd, *J* = 7.6, 1.0 Hz, 2H*, -Ar)*, 7.63 – 7.59 (m, 2H*, -Ar)*, 7.43 – 7.28 (m, 8H*, -Ar)*, 7.21 – 7.13 (m, 1H, *-Ar*), 6.84 – 6.63 (m, 2H, *-Ar)*, 5.16 (d, *J* = 44.7 Hz, 2H, -C*H₂*-, Cbz), 4.91 (d, *J* = 2.4 Hz, 2H, Ar-C*H₂*-N-), 4.40 (d, *J* = 7.4 Hz, 2H, , -C*H₂*-OFmoc), 4.25 (t, *J* = 7.4 Hz, 1H, ,-C*H*-, Fmoc), 4.20 – 4.07 (m, 2H, -C*H₂*-OFmoc), 3.94 (dt, *J* = 42.4, 6.0 Hz, 2H, -O-*CH₂*-CH₂-CH₂-COOH), 3.40 – 3.26 (m, 2H, -NCbz-C*H₂*-CH₂-), 2.59 – 2.52 (m, 2H, -CH₂-C*H₂*-COOH), 2.14 – 2.05 (m, 2H, -CH₂-C*H₂*-CH₂-), 1.76 – 1.56 (m, 4H, C*H₂*-CH₂-C*H₂*-), 1.45 – 1.3 (m, 2H, -CH₂-C*H₂*-CH₂-) ppm; ^{13}C NMR (101 MHz, $CDCl_3$): δ = 178.2, 177.1, 163.3, 156.5, 155.4, 143.5, 141.4, 141.1, 140.8, 137.8, 137.3, 136.5, 128.7, 128.5, 128.4, 128.3, 128.2, 128.1, 128.0, 127.2, 125.3, 120.1, 113.7, 112.9, 112.9, 112.6, 69.8, 68.1, 67.7, 67.4, 67.2, 49.1, 48.9, 48.2, 47.7, 46.8, 30.3, 30.2, 28.4, 28.1, 27.6, 24.1, 24.1, 23.1, 20.8 ppm; IR (neat) ν_{max}: 2951, 1743, 1707, 1580, 1514, 1258, 744 cm^{-1}; UV/Vis (DMF): $\lambda_{max}(\varepsilon)$ = 268; 300 nm; ESI-HRMS: m/z $[M+Na]^+$ calcd. for $C_{39}H_{40}N_2O_{10}$ Na: 719.2575 found 719.2585.

^{1}H NMR ($CDCl_3$)

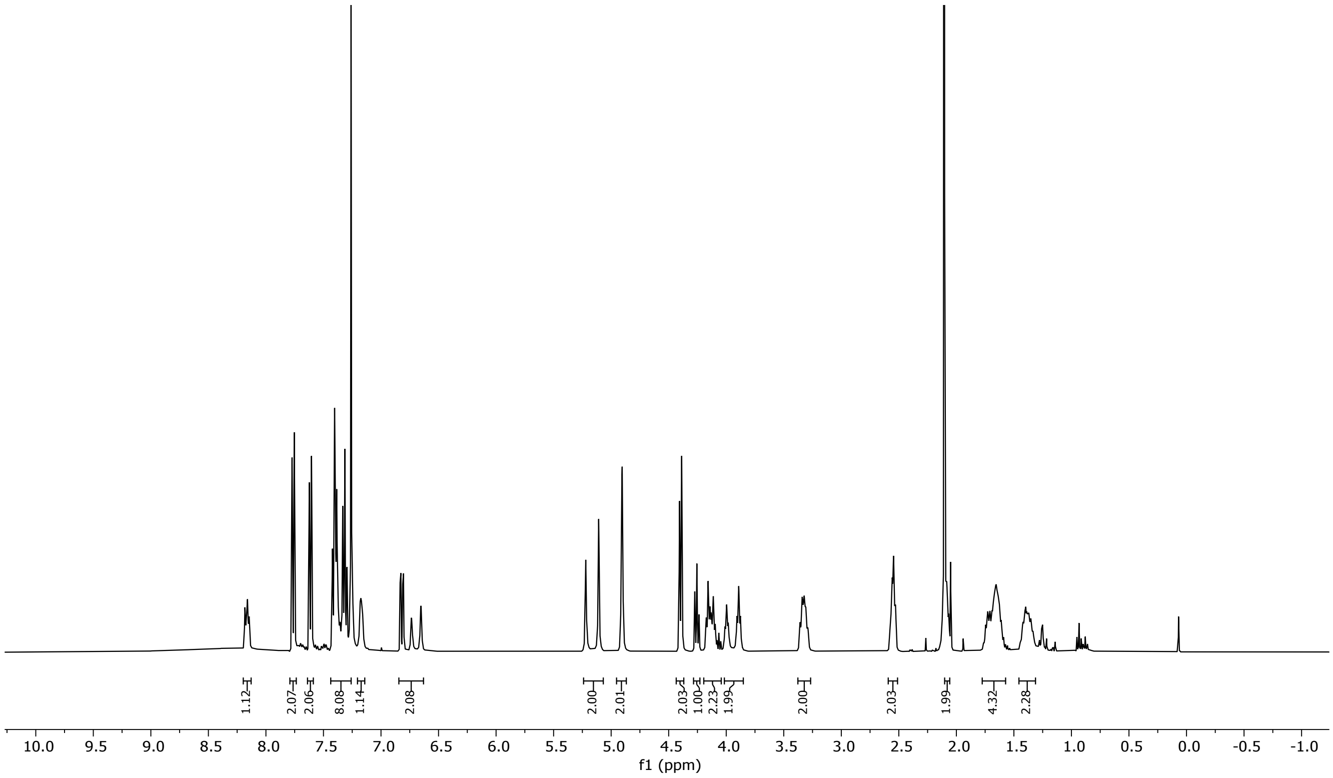

^{13}C NMR ($CDCl_3$)

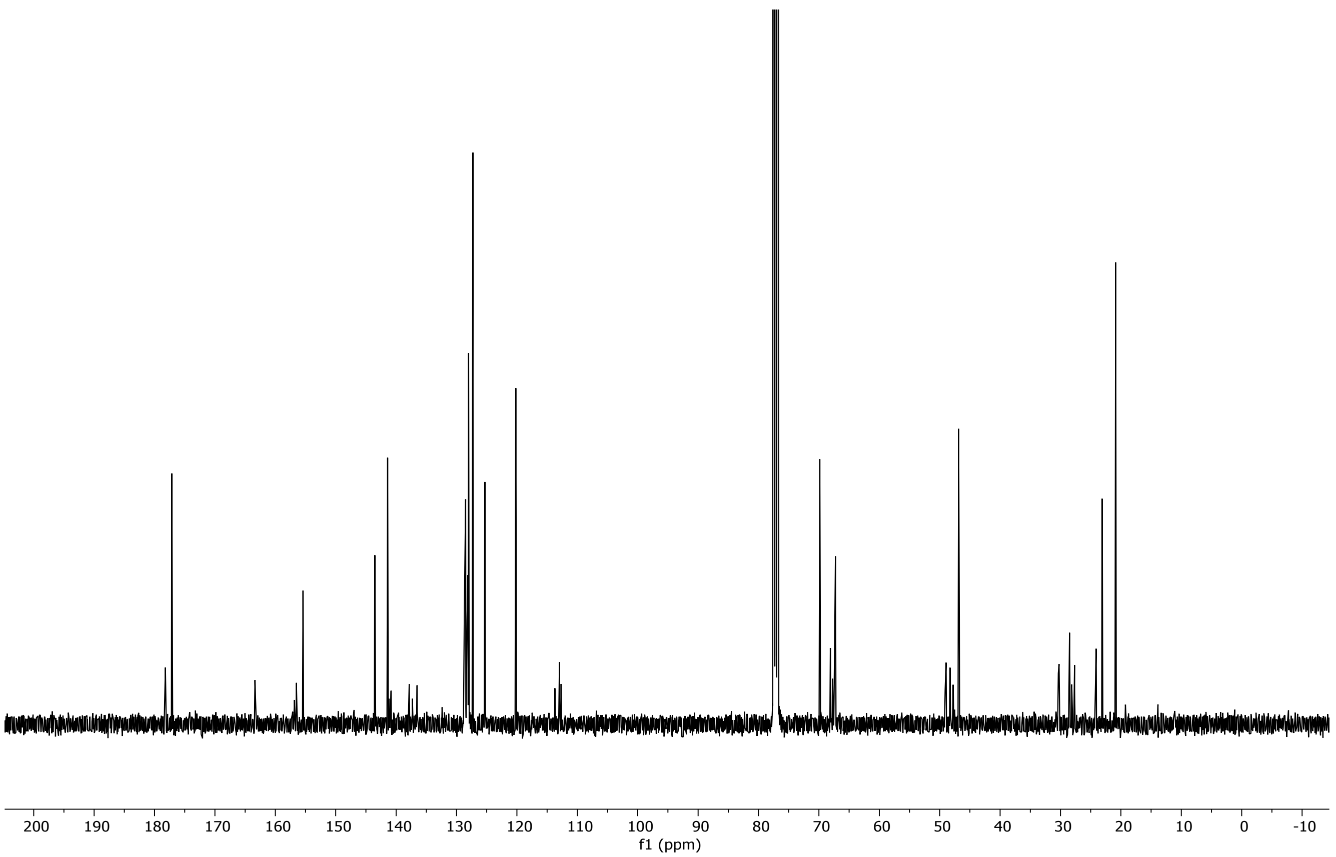

HSQC ($CDCl_3$)

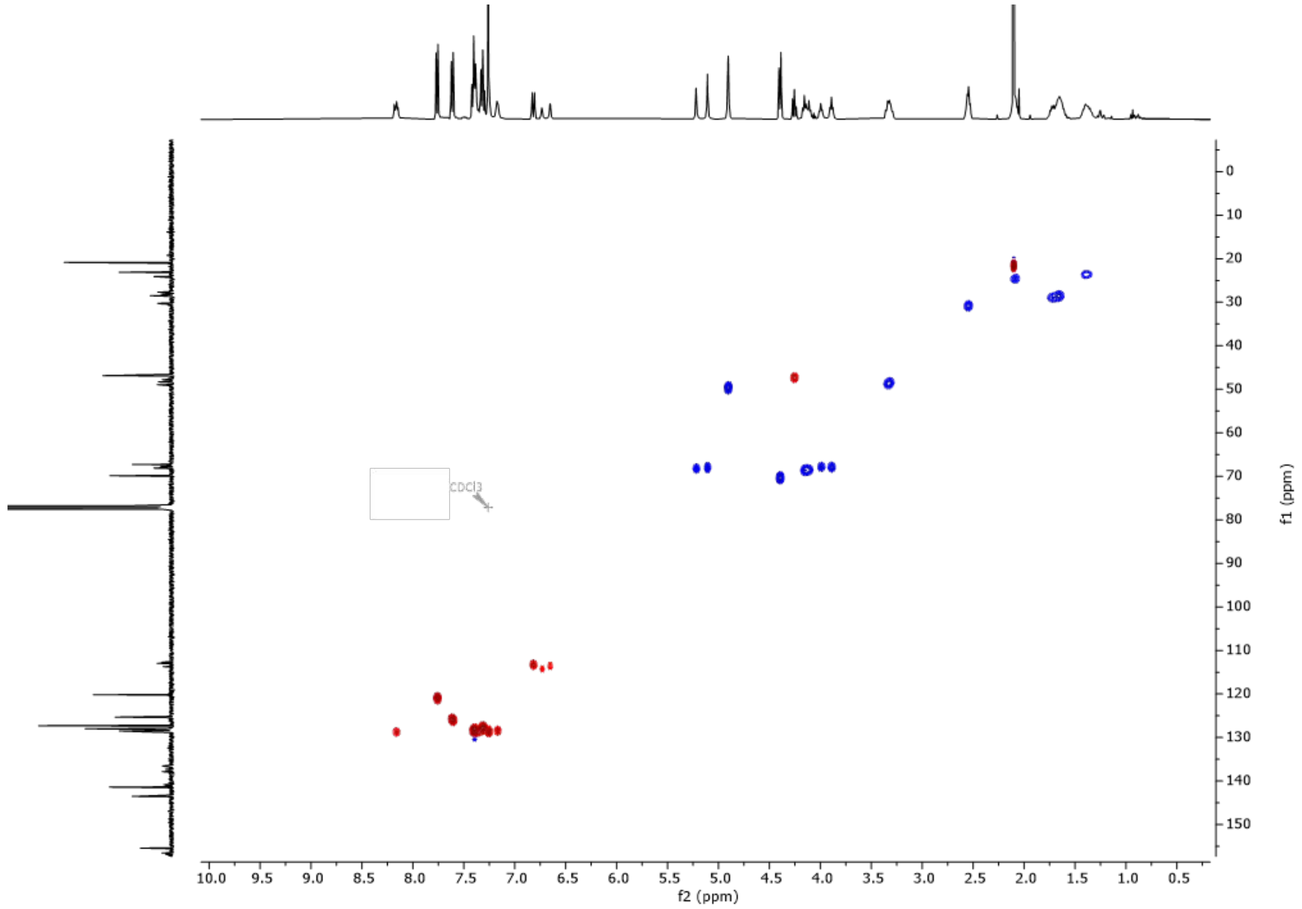

E. Functionalization of cellulose membrane

Pretreatment of cellulose membrane

Commercially available Fmoc-β-alanine (AIMS Scientific Products) functionalized membrane is shown as a simplified representation **30** (Figure S1). The membrane was immersed in DMF for swelling in a petri dish. After 15 min of shaking (300 rpm) at ambient temperature, DMF was removed. The membrane was washed consecutively with DMF (3 × 5 min each), MeOH (1 × 2 min), DCM (1 × 1 min) and dried in a jet of air.

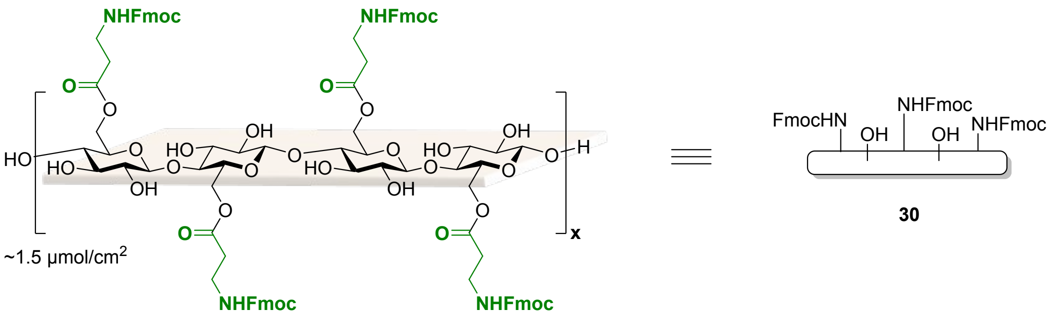

Figure S1: Simplified representation of cellulose membrane used for our experiments

Capping of β-alanine-cellulose membrane

The remaining free hydroxyl groups on the membrane **30** were capped, with the acidic capping mixture (see Preparation of stock solutions) for 30 min. The same process repeated for another 30 min at rt (300 rpm). Then, the membrane washed consecutively with DMF (3 × 10 mL for 5 min each), MeOH (1 × 10 mL for 2 min), DCM (1 × 10 mL for 1 min) and dried in a jet of air.

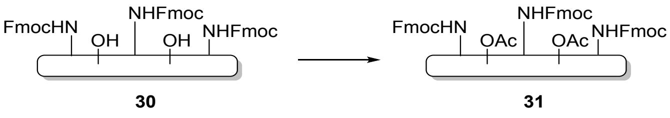

Fmoc-deprotection of β-alanine on cellulose membrane

The Fmoc-protected β-alanine-cellulose membrane **31** was immersed in Fmoc-deprotection solution (2 mL/cm^2 of membrane, see Preparation of solutions) for 20 min on a shaker. The membrane was washed and dried in a jet of air to obtain the free amino groups on the membrane **32**.

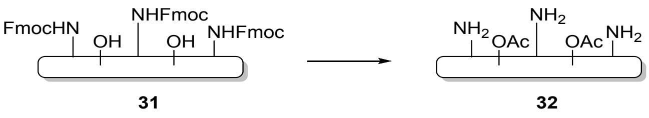

Functionalization with base-labile linker

Base-labile linker attachment

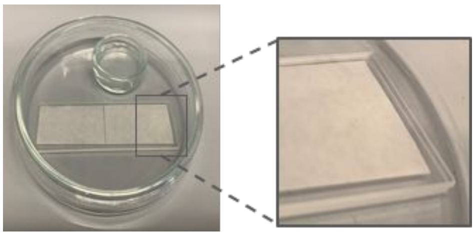

Figure S2: Attachment of linker on cellulose membrane via sandwich approach.

Base-labile linker **28** (2.00 equiv) was dissolved in DMF (peptide grade) (0.05 mL/cm^2) in a vial, DIC (2.00 equiv.) and hydroxybenzotriazole (HOBt) (2.00 equiv.) were added consecutively, and the vial was shaken for 5 min. The resulting solution was pipetted on the free amino membrane **32** between two microscope glass slides in a sandwich approach (Figure S2), and was left overnight in a petridish to react under humid DMF conditions. Then, the membrane washed consecutively with DMF (3 × 10 mL for 5 min each), MeOH (1 × 10 mL for 2 min), DCM (1 × 10 mL for 1 min) and dried in a jet of air.

Capping of unreacted amine groups

The remaining unreacted free NH_2 groups on the membrane were subjected to basic acetylation (see Preparation of solutions) for 30 min. The same process was repeated with a freshly prepared capping solution for another 30 min at rt (300 rpm). Then, the membrane was dried in a jet of air to obtain the Fmoc-protected amine base-labile functionalized membrane **33** with capped unreacted amine groups.

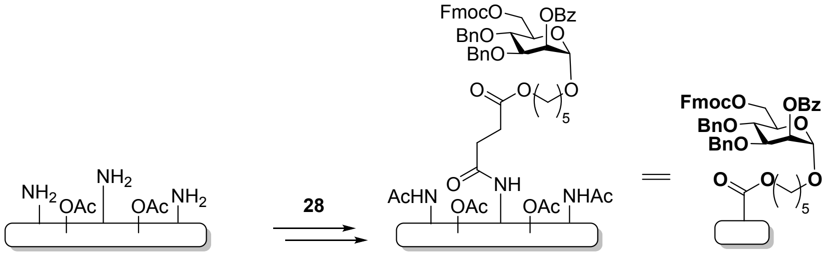

32 **33** **33**
simplified representation

Functionalization with photo-labile linker

Photo-labile linker attachment

Photo-labile linker **29** (2.00 equiv.) was dissolved in DMF (peptide grade) (0.05 mL/cm^2) in a vial, DIC (2.00 equiv.) and HOBt (2.00 equiv.) were added consecutively and the vial was shaken for 5 min. The resulting solution was pipetted on the free amino membrane **32,** between two microscope glass slides, sandwich approach (Figure S2), and was left overnight in a petri dish to react under humid DMF conditions. Then, the membrane was washed consecutively with DMF (3 × 10 mL for 5 min each), MeOH (1 × 10 mL for 2 min), DCM (1 × 10 mL for 1 min) and dried in a jet of air.

Capping of unreacted amine groups

The remaining unreacted free NH_2 groups on the membrane were subjected to basic acetylation (see Preparation of solutions) for 30 min. The same process was repeated with a freshly prepared capping solution for another 30 min at rt (300 rpm). Then, the membrane was dried in a jet of air to obtain the Fmoc-protected amine-base-labile-functionalized membrane **34** with capped unreacted amine groups.

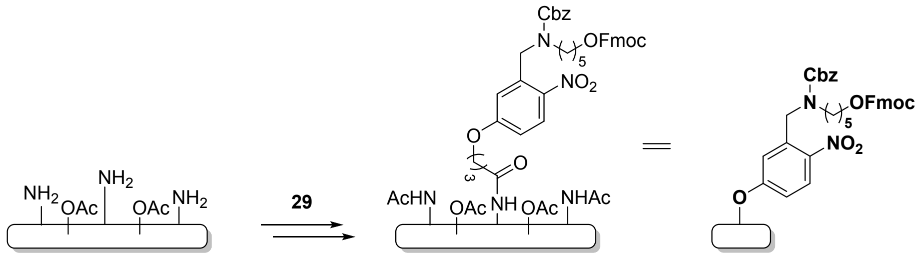

32 **34** **34**

simplified representation

Quantification/loading determination of functionalized membrane

Dry functionalized **33**/**34** membrane (2 cm^2, suppliers loading: 1.55-1.33 µmol/cm^2), was immersed in DMF for swelling in a vial. After 15 min of shaking (300 rpm) at rt, DMF was removed and Fmoc-deprotection solution was added. The membrane was shaken for 20 min at rt (300 rpm). From this vial, containing the membrane, 100 µL of the Fmoc deprotection solution were diluted in 900 µL of the stock deprotection solution reaching 1.00 mL of total volume and the UV absorption of this solution was measured at 290 nm. Calibration of the absorbance of the spectrophotometer was performed at 290 nm using 1.00 mL of the stock Fmoc-deprotection solution. The absorbance was measured three times for each solution and the average value was obtained for the determination of the Fmoc loading of the functionalized membrane using the following equation:

$$Loading\ (\frac{\mu mol}{cm2}) = (E \times V \times DF)/(\ d \times \varepsilon \times A)$$

E: Average of absorbance of the sample solution at 290 nm

V: Volume of deprotection solution used (4.00 mL)

DF: Dilution factor (10)

d: Path length of cuvette (1 cm)

ε: Molar absorption coefficient at 290 nm (5253 M^{-1} cm^{-1})

A: Size of membrane (2 cm^2)

Loading of used membrane: $(0.20 \times 4\ mL \times 10)$ / (1 cm^1 × 5253 10^3 mL 10^{-6} μmol^{-1} cm^{-1} × 2 cm^2) ≈ 0.76 µmol/cm^2

F. VaporSPOT glycosylation

Preparation of stock solutions

- **Building block solution:** Building block (6.00 equiv. /cycle) was dissolved in 50 µL of anhydrous dichloromethane (DCM).
- **Fmoc-deprotection:** A solution of 20% piperidine in dimethylformamide (DMF) (v/v) was prepared.
- **Acidic capping:** A solution of 10% acetic anhydride (Ac_2O) and 2% methanesulfonic acid (MsOH) in 88% anhydr. DCM (v/v) was used.
- **Basic capping:** A solution containing 10% Ac_2O and 20% *N,N*-diisopropylethylamine (DIPEA) in 70% DMF (v/v) was prepared.
- **Acidic wash:** A solution containing 0.5% trimethylsilyl trifluoromethanesulfonate (TMSOTf) in DCM was prepared.
- **Activator solution A:** A solution of 8% TMSOTf in DCM was prepared.
- **Activator solution B:** A solution of 4% TMSOTf in DCM was prepared.
- **Activator solution C:** A solution of 2% TMSOTf in DCM was prepared.

- **Methanolysis solution:** Solution for deprotection of the ester protecting groups & cleavage from the membrane prepared dissolving 0.5 mL of sodium methoxide (NaOMe, 0.5 M) solution in 5 mL of methanol (MeOH).

Homebuilt setup

The glycosylation setup (Figure S3) has five compartments: the vapor generator; the syringe; the glycosylation chamber; the two valves controlling the flow and the amount of the deposited solution, and the computer system for temperature control inside the glycosylation chamber. The temperature is controlled by a thermoelectric cooling element, which is located at the bottom of the glycosylation chamber, connected to a computer for automated software control of the temperature during the synthesis. The thermoelectric cooling element PE-127-14-11 (15.7 V, 8.5 A, 82 W, Laird Connectivity, UK) for temperature control of the chamber was connected to a power supply unit Manson HCS - 3402 (0 – 32 V, 0 – 20 A, Manson Engineering Industrial Ltd, Hongkong). To ensure temperature stability of the system, the back of the thermoelectric element is connected to a CPU heat sink with a fan, whereas the top is covered with a thin copper plate and an insulating foam with a cutaway for the glycosylation chamber. A custom-developed software (LabView) is used to control the temperature of the thermoelectric cooling element, which is monitored by a temperature sensor at the copper plate.

The two valves provide control over the atmosphere inside the setup (air, vacuum, and argon). The right valve provides atmospheric air, as well as high vacuum or argon (connection with the Schlenk line), while the left valve is connected with the vapor generator vessel. The vapor generation vessel is a fritted U-shaped glass tube connected with the Schlenk line for argon flow, which results in vapor generation of the activator solution and transfer into the glycosylation chamber. The activator solution is injected into the vapor generator with a 1 mL syringe. All tubing and valves are PTFE-based.

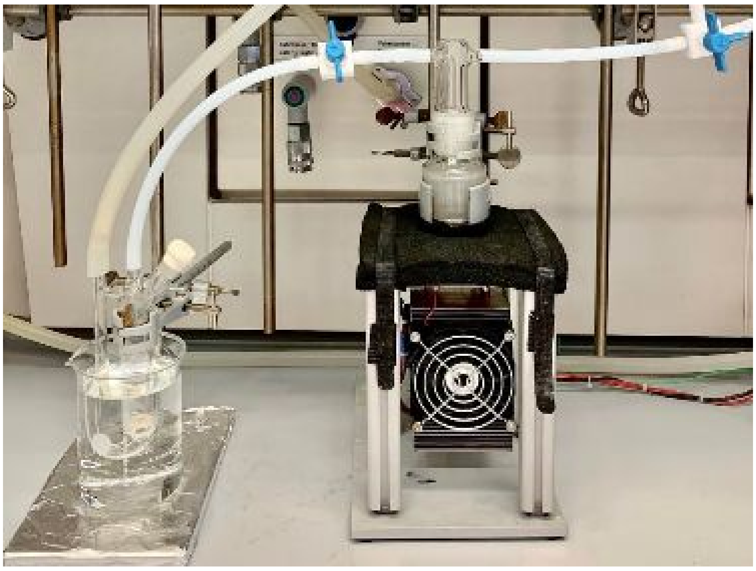

Figure S3: Experimental setup used for VaporSPOT glycosylation.

Temperature profile and regulation system

The thermoelectric cooling element, which is located at the bottom of the glycosylation chamber is connected to a computer. The temperature range per glycosylation cycle is from -15°C to rt (25°C), set temperature (set point, Figure S4, blue). The cooling process, before initiating the glycosylation, lasts 30 min, while the cooling rate is 8°C/min. After the cooling process, the reaction starts by transferring the total amount of the activator solution into the glycosylation chamber using argon flow (2 min) at -15°C. Then, the temperature is increased from -15°C to rt (25°C), and the reaction continues for 30 min under these conditions. The heating and cooling rate are both 8°C/min. After 30 min, the setup was subjected to high vacuum for 5 min at 25°C, to remove the remaining solvent and activator from the glycosylation chamber. Figure S3 depicts the temperature profile

of every step inside the glycosylation chamber, with the green line representing the actual (measured) temperature profile and in blue the set points.

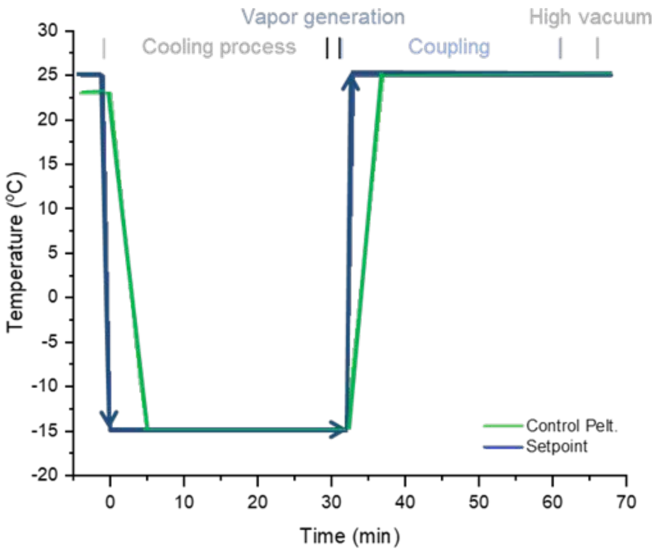

Figure S4: Temperature profile inside the reaction vessel during one coupling cycle (cooling and heating rate: 8°C/min).

Concentration of activator solution

To develop a stable and reproducible method for oligosaccharide synthesis on membranes, the protocol had to be optimized. We initially used an activator solution (dimer synthesis and screening of building blocks **1**-**6, 8**-**13**) with 8% TMSOTf in DCM (Activator solution A). Unfortunately, when we tried to synthesize longer oligosaccharides, the membrane lost its flexibility and decomposed after the second or third glycosylation step (Figure S5). Therefore, we tested different TMSOTf concentrations for trisaccharide synthesis **18**. We observed that reducing the amount of activator solution can have a positive outcome on the reaction yield and the membrane stability (see Section of Oligosaccharide synthesis). Using 4% of TMSOTf (Activator solution B) led to 32% yield of the desired trimer **18**, while 2% of TMSOTf (Activator solution C) gave 25% yield. Thus, for further syntheses, we selected Activator solution B (4% TMSOTf). Subsequent glycosylation reactions such as parallel synthesis with building blocks **1-3**, **7**, **13-14**, screening of lactose imidate **14**, and synthesis of tetramannoside **19** were performed with Activator solution B.

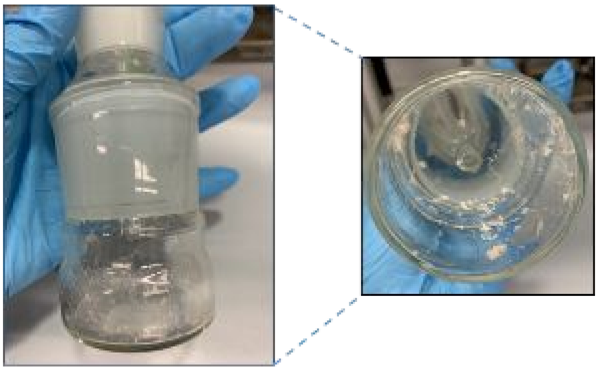

Figure S5: Decomposition of the cellulose membrane after the 3rd glycosylation step, using activator solution A.

Concentration/equivalents of glycosyl donor

We calculated this in respect to the loading of the functionalized membrane. We based our conclusions on the qualitative crude ELSD trace, calculating the ratio of the peak integrals for the dimer vs. monomer (Figure S6).

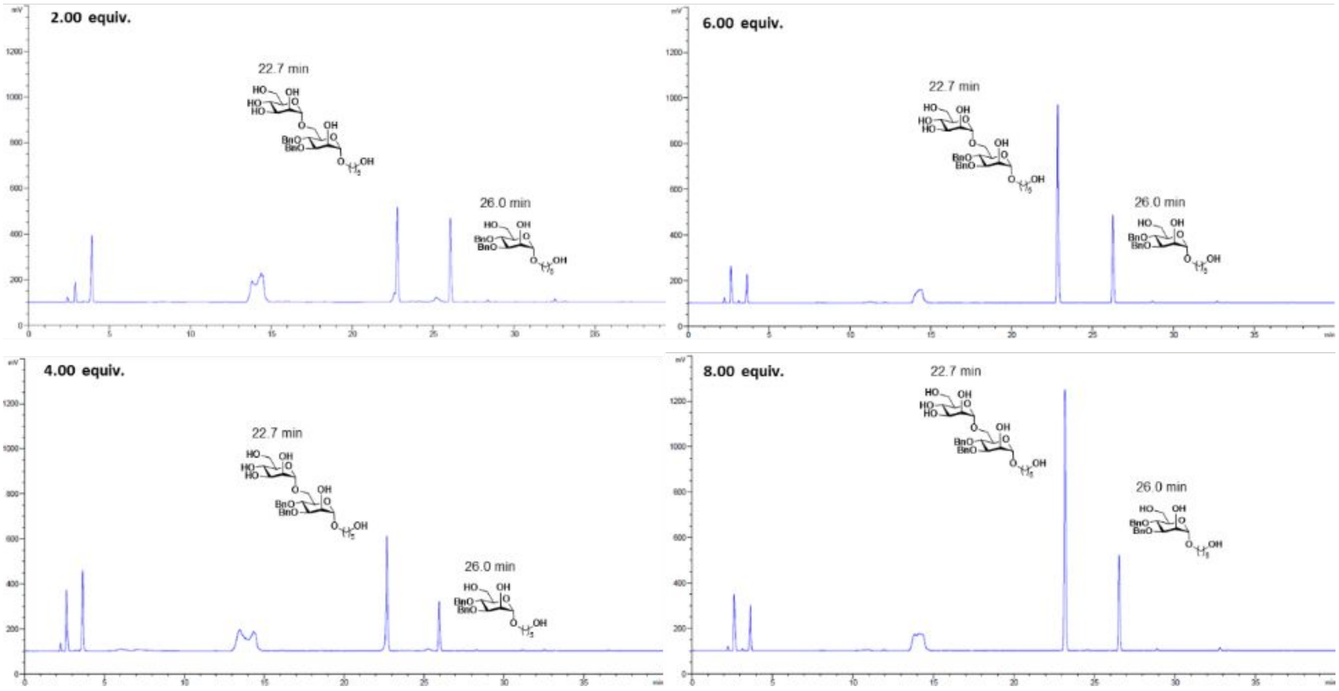

Figure S6: RP-HPLC (ELSD trace, Method B) of crudes, synthesized using different donor equivalents.

Only minor differences were observed (Table S1) in the dimer/monomer ratio between 4.00, 6.00 and 8.00 equivalents of donor. However, the peak intensity of the synthesized dimer increased with increasing donor equivalents. The intensity of the synthesized dimer between 6.00 and 8.00 equiv. was relatively similar and since we wanted to establish a cost efficient methodology, 6.00 equivalents were chosen as the optimum amount for the spotted donor/glycosylation cycle in respect to the membrane functionalization (1.00 equiv.).

Table S1: Qualitative analysis of the ratio between dimer vs. monomer formation

Activator solution: 4 % TMSOTf in DCM<br>Parallel synthesis, A = 1 cm^2, loading: ≈ 0.8 µmol/cm^2	
**BB 1**	**Dimer-Monomer**
2.00 Equiv.	≈ 1.3:1
4.00 Equiv.	≈ 2.2:1
6.00 Equiv.	≈ 2.3:1
8.00 Equiv.	≈ 2.7:1

Module A: Membrane preparation for synthesis (39 min)

All syntheses were performed on 2 cm^2 cellulose membrane pieces. The membrane was initially placed into a vial, and swollen for 15 min in DMF on a shaker at rt prior to the synthesis (300 rpm). Then, the membrane was washed with DMF (3 × 3 min), deprotected using Fmoc-deprotection solution, and washed with DMF (3 × 3 min), MeOH (1 × 3 min), and DCM (1 × 3 min). The membrane was placed in a Schlenk flask and dried overnight under high vacuum (16 h).

Module B: Acidic wash prior to glycosylation (58 min)

The membrane was swollen again for 15 min in anhyd. DCM under inert conditions, and washed with anhydr. DCM (3 mL, 3 × 1 min). The acidic wash solution was added to the flask under inert conditions and stirred for 1 min. The acidic solution was removed, the membrane washed with anhydr. DCM (3 mL, 1 × 3 min), DMF (3 mL, 1 × 3 min), DCM (3 mL, 1 × 3 min) and dried under high vacuum for 30 min.

Module C: Spotting of BB (31 min)

The building block solution (6.00 equiv./glycosylation cycle in 100 µL of anhydr. DCM, with respect to the loading of the used functionalized membrane) was spotted onto the membrane carefully under high argon flow inside the flask, 1 min, and dried for 30 min under high vacuum.

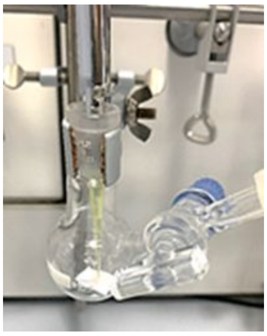

Figure S7: Spotting of a glycosyl donor onto a functionalized membrane, bearing a glycosyl acceptor, inside a Schlenk flask under argon counterflow.

Module D: Vapor glycosylation (106 min)

The membrane, carrying an unreacted glycosyl donor (and the acceptor) was placed inside the glycosylation chamber and the temperature adjusted to -15°C. For the next 30 min, the glycosylation chamber was kept under constant argon flow, and after the set temperature was reached, the reaction started by transferring the activator solution into the glycosylation chamber using argon flow. After complete deposition of solvent and activator inside the chamber (2 min), the two valves of the system were closed. Then, the temperature was increased to rt (8°C/min to 25°C). After 30 min, the setup was subjected to high vacuum for 5 min to remove the remaining solvent and activator from the glycosylation chamber. Finally, the membrane was washed with DCM (1 × 3 min), DMF (1 × 3 min), DCM (1 × 3 min), dried in a jet of air, and stored under high vacuum for 30 min. Modules C and D were repeated one more time to ensure high/quantitative conversion.

Module E: Fmoc-deprotection (35 min)

The membrane was deprotected using Fmoc-deprotection solution for 20 min, and washed with DMF (3 × 3 min), MeOH (1 × 3 min), and DCM (1 × 3 min). Then, the membrane was placed in a Schlenk flask, and dried overnight under high vacuum (16 h). All modules were repeated for the synthesis of longer structures.

Post-Synthesis manipulation

Module F1: Base-labile linker

After completion of the synthesis, the oligosaccharides were deprotected and simultaneously cleaved from the solid support/ membrane using Zemplén deprotection conditions. The membranes were placed separately in 5 mL flasks, containing a stirring bar and 2 mL of the deprotection solution was added. The mixtures were left to react overnight at rt (Figure S8, A). Then, the

solution was neutralized by Amberlite IR-120 H^+. Finally, the solution was filtered off, washed with acetonitrile (ACN) and water via a hydrophobic syringe filter, and concentrated under reduced pressure.

Module F2: Photo-labile linker

After completion of the synthesis, the oligosaccharides bound to the photo-labile linker were subjected to cleavage under a UV-lamp (365 nm, 2 × 8 W). The distance between the lamp and the membranes was approximately 4 cm, and the membranes were irradiated 15 min on both sides (Figure S8, B). Then, each membrane was cut into small pieces and placed inside a vial. The pieces were washed with MeOH (2 mL × 5 min) and DCM (2 mL × 5 min). The solutions were concentrated under reduced pressure.

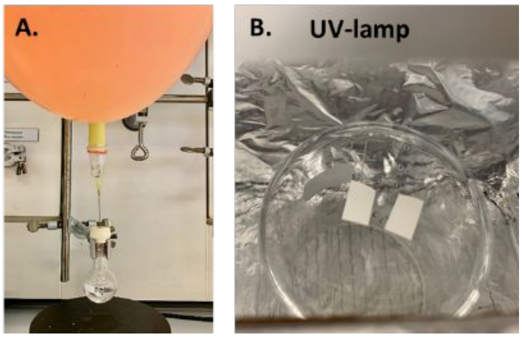

Figure S8: Post synthesis manipulation of synthesized structures: A). Deprotection and cleavage of the synthesized structure using sodium methanolate in solution, and B) Parallel UV-cleavage of synthesized structures on two different membrane pieces under UV-light.

Purification & Characterization

Module G: Reverse-phase purification

To identify and characterize the final oligosaccharides, the crude products were dissolved in water and purified in a preparative reverse phase HPLC (Agilent 1200 Series, Method A), and the pure compounds were analyzed using an analytical HPLC (Agilent 1200 Series, Method B).

- Method A: Synergi Hydro RP18 column, 250 x 10 mm, flow rate of 4 mL/min with 5% ACN in H_2O (0.1% formic acid) as eluents [isocratic (5 min), linear gradient to 5% ACN (35 min), linear gradient to 100% ACN (5 min)].
- Method B: Synergi Hydro RP18 column, 250 x 4.6 mm, flow rate of 1 mL/min with 5% ACN in H_2O (0.1% formic acid) as eluents [isocratic (5 min), linear gradient to 5% ACN (35 min), linear gradient to 100% ACN (5 min)].

Finally, all deprotected products were lyophilized prior to characterization.

G. Oligosaccharide synthesis

5-Hydroxypentyl 3,4-di-*O*-benzy-α-D-mannopyranoside (35)

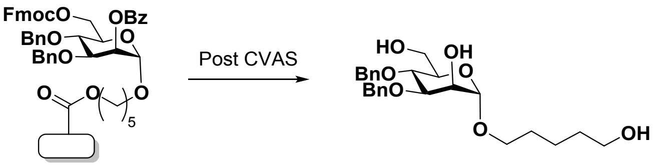

33 35

Action	BB	Modules	Notes	Result (mg, µmol, yield)
CVG		A	**33** swell	
	-	-	-	0.45 mg, 1.00 µmol, 40%
Post CVG		F1		

^{1}H NMR (400 MHz, D_2O):): δ = 77.51 – 7.17 (m, 10H, -*Ar*), 4.81 (d, *J* = 1.9 Hz, 1H, *H*-1), 4.71y (dd, *J* = 11.0, 1.9 Hz, 2H; -CH_2-Ar), 4.70 (d, *J* = 1.3 Hz, 2H; -CH_2-Ar); 4.09 (dd, *J* = 3.1, 1.9 Hz, 1H; *H*-2), 3.81 – 3.75 (m, 2H, *H*-3; *H*-6α), 3.71 (d, *J* = 9.8 Hz, 1H; *H*-4), 3.69 – 3.66 (m, 1H; *H*-6β), 3.66 – 3.60 (m, 1H; -O-C*H*H-CH_2-), 3.57 (ddd, *J* = 9.8, 5.5, 2.1 Hz, 1H; *H*-5), 3.51 (t, *J* = 6.6 Hz, 2H, -CH_2-OH), 3.45 (dd, *J* = 9.9, 6.0 Hz, 1H, -O-C*H*H-CH2-), 3.57 (ddd, *J* = 9.8, 5.5, 2.1 Hz, 4H, -CH_2-CH_2-), 1.28 – 1.11 (m, 2H, -O-CH_2-CH_2-) ppm; ^{13}C NMR (101 MHz, $CDCl_3$): δ = 171.9, 138.2, 138.0, 129.7, 129.6, 129.4, 129.3, 100.5, 79.4, 76.0, 75.0, 72.8, 72.2, 68.6, 67.9, 62.6, 61.6, 31.9, 29.1, 22.7 ppm; ESI-HRMS: m/z $[M+Na]^+$ calcd. for $C_{25}H_{34}O_7Na$: 469.2196 found 469.2203.

RP-HPLC (ELSD trace, Method B, t_R = 26.2 min)

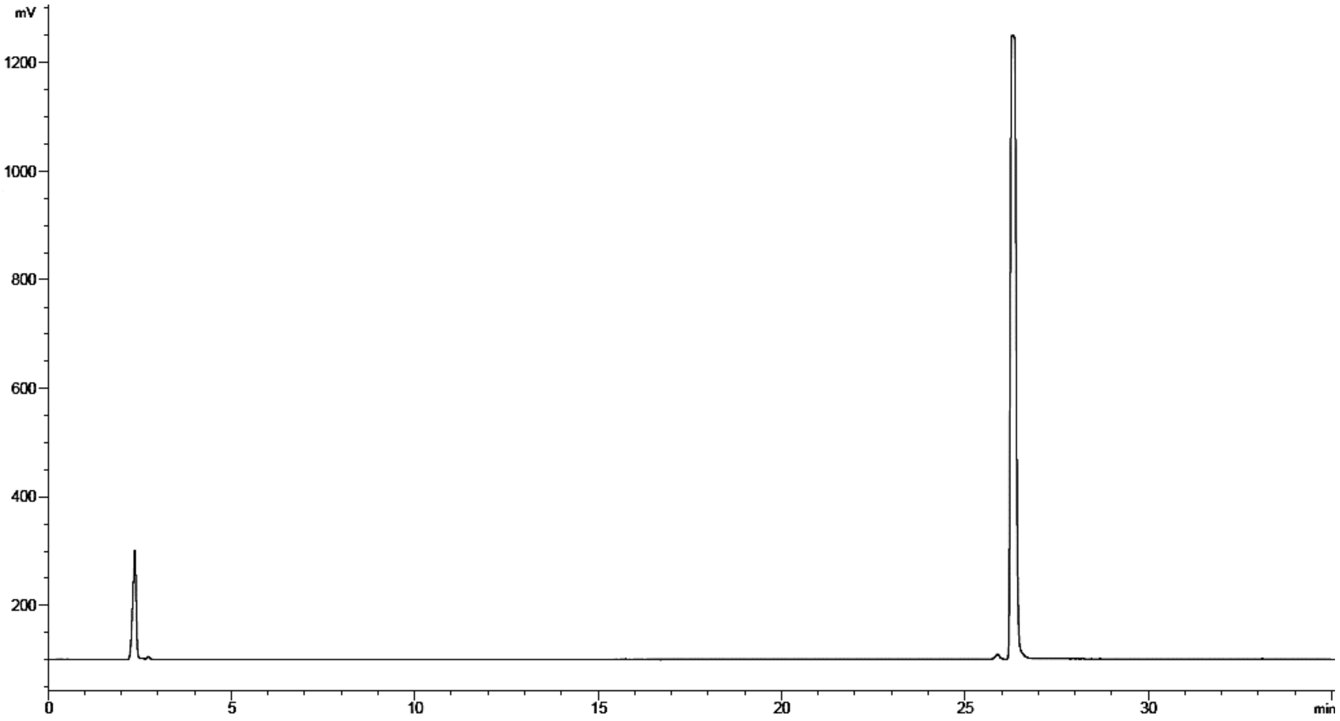

^{1}H NMR (D_2O)

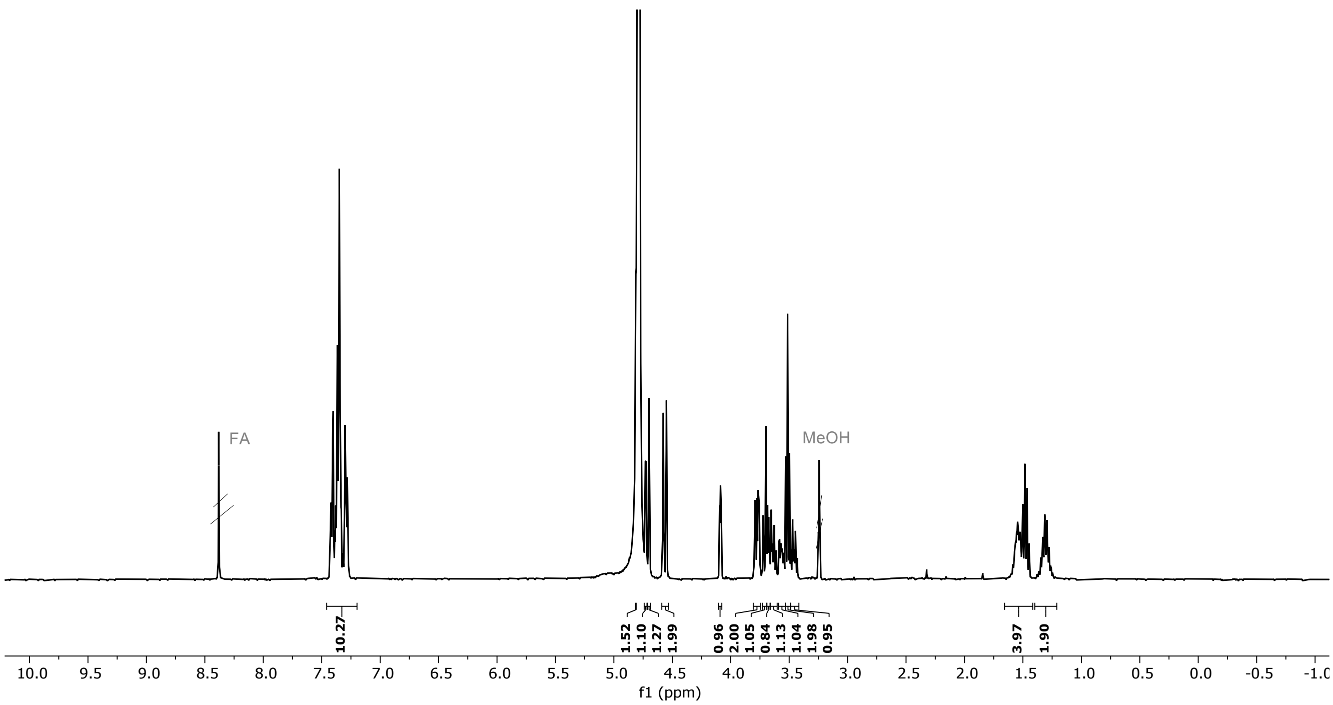

^{13}C NMR (D_2O)

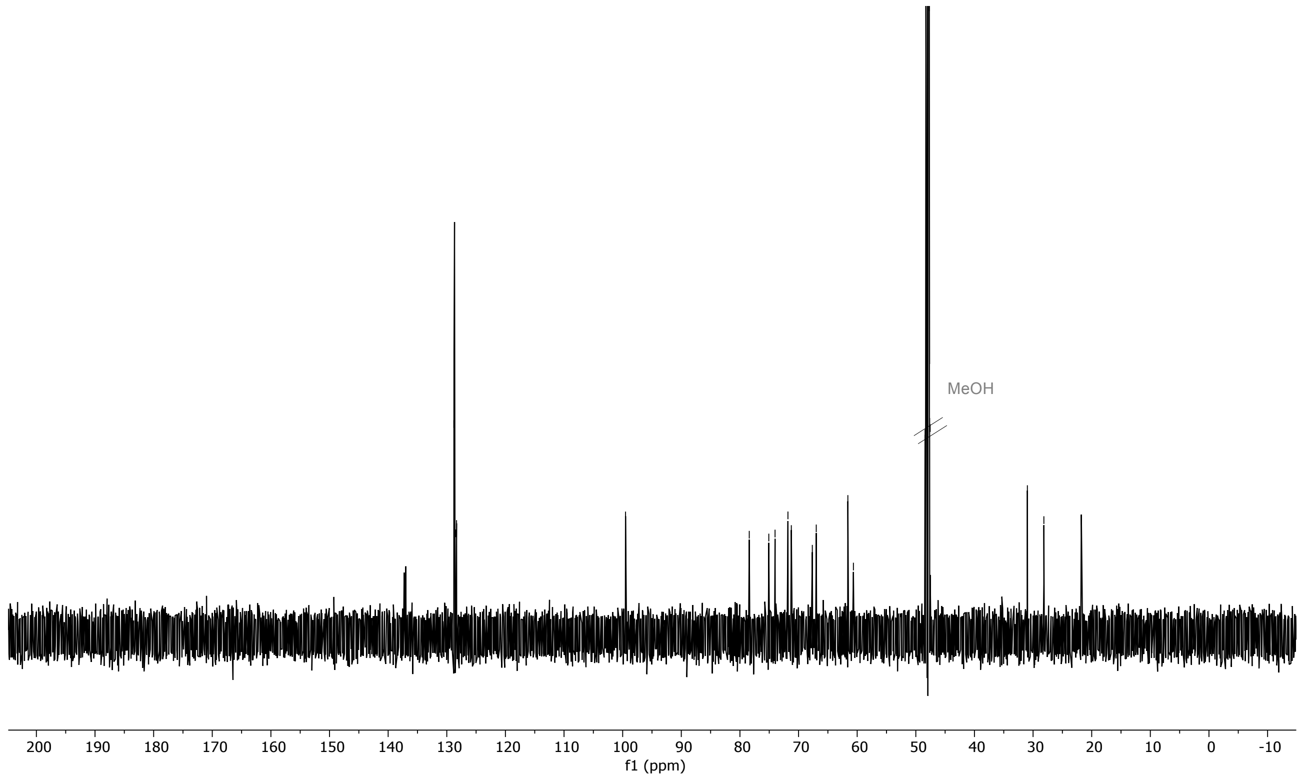

HSQC (D_2O)

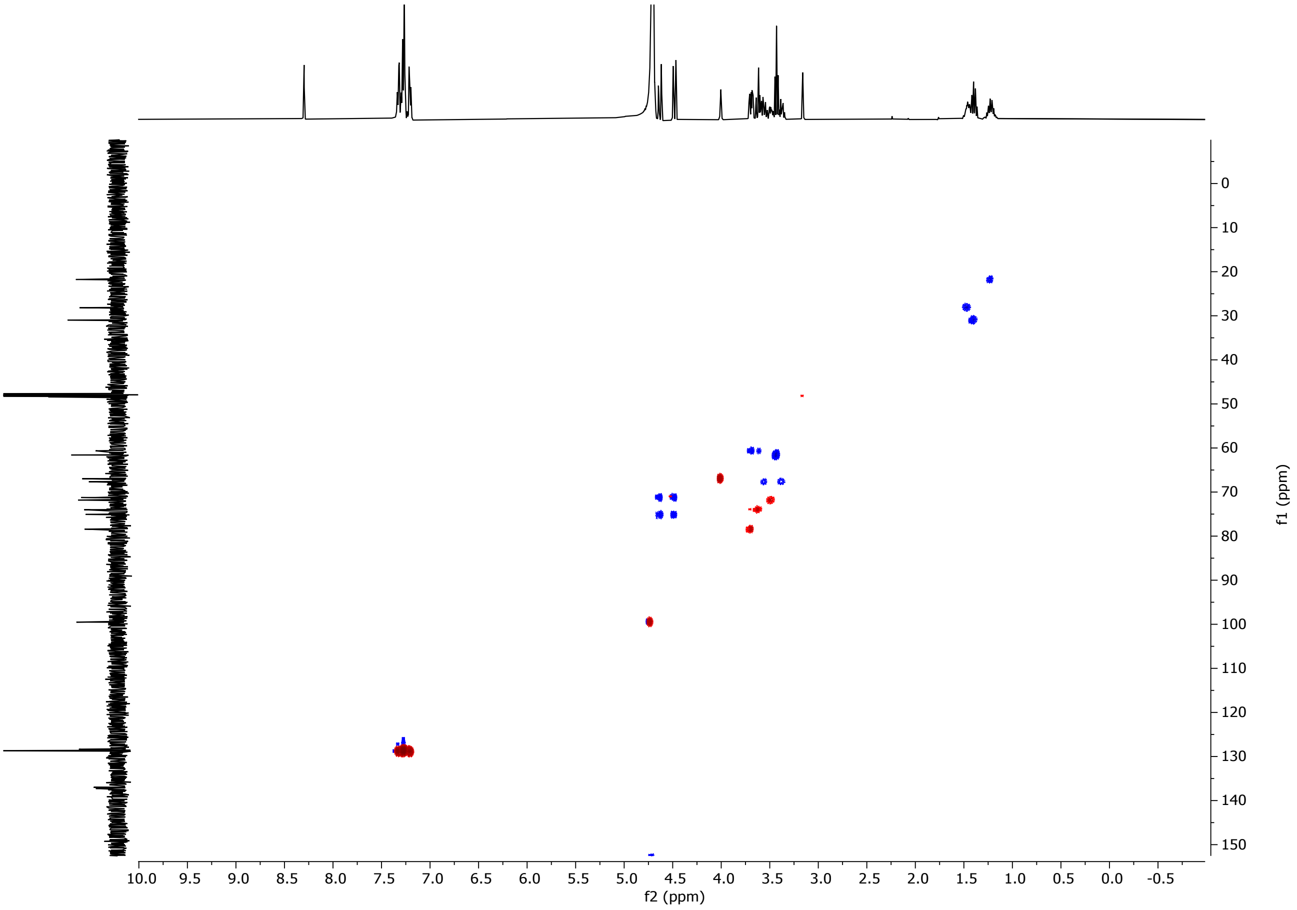

5-Hydroxypentyl α-D-mannopyranosyl-(1→6)-3,4-di-*O*-benzyl-α-D-mannopyranoside (15)

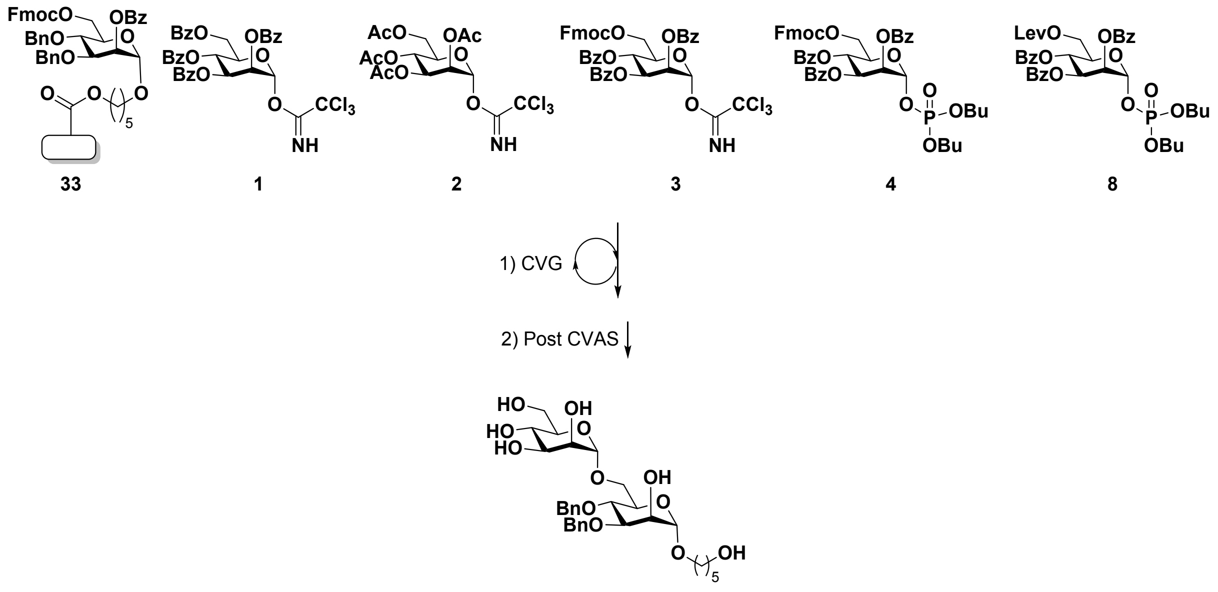

Action	BB	Modules	Notes	Result (mg, µmol, yield)
Method A				
CVG	1	A	**33** swell; Fmoc-deprotection	
		B; C; D	Activator solution A, 1.00 mL	0.35 mg, 0.58 µmol, 26%
Post CVG		F1		
Method B				
CVG	1	A	**33** swell; Fmoc-deprotection (1 cm^2 size of membrane)	0.1 mg, 0.16 µmol, 21%
		B; C; D	Activator solution B, 1.00 mL	
Post CVG		F1		
Method C				
CVG	2	A	**33** swell; Fmoc-deprotection	
		B; C; D	Activator solution A, 1.00 mL	0.29 mg, 0.48 µmol, 21%
Post CVG		F1		
Method D				
CVG	3	A	**33** swell; Fmoc-deprotection	
		B; C; D; C; D[*]	Activator solution A, 1.00 mL	0.41 mg, 0.67 µmol, 30%
Post CVG		F1		
Method E				
CVG	4	A	**33** swell; Fmoc-deprotection	
		B; C; D; C; D	Activator solution A, 1.00 mL	0.21 mg, 0.35 µmol, 15%
Post CVG		F1		
Method F				
CVG	8	A	**33** swell; Fmoc-deprotection (1 cm^2)	
		B; C; D; C; D	Activator solution A, 1.00 mL	0.23 mg, 0.38 µmol, 48%
Post CVG		F1		

[*]Single glycosylation of building block **3** under the same glycosylation conditions (modules A, B, C, D, and Post CVG) delivered only 0.24 mg, 17% of the desired dimer **15**. Thus, repetion of modules C and D was required to increase the coupling yield. According to this result, Modules C and D were repeated for all building blocks bearing electron-donating groups (EDG) as well temporary protecting groups for chain elongation.

^{1}H NMR (700 MHz, D_2O): δ = 7.37 – 7.25 (m, 8H), 7.22 – 7.18 (m, 2H), 4.65 (s, 2H), 4.63 (s, 1H), 4.48 (dd, *J* = 11.2, 5.0 Hz, 2H), 4.03 (t, *J* = 2.4 Hz, 1H), 3.85 – 3.79 (m, 1H), 3.78 – 3.34 (m, 13H), 1.43 (dd, *J* = 21.1, 7.2 Hz, 4H), 1.28 – 1.11 (m, 2H) ppm; ^{13}C NMR (176 MHz, D_2O): δ = 128.7, 128.4, 128.4, 99.5, 78.2, 74.7, 73.7, 72.6, 71.3, 70.1, 69.8, 67.9, 66.6, 66.1, 65.9, 61.1, 60.5, 30.7, 27.6, 22.0 ppm (in respect to the coupled ^{1}H–^{13}C HSQC NMR); ESI-HRMS: m/z $[M+Na]^+$ calcd. for $C_{31}H_{44}O_{12}Na$: 631.2724 found 631.2745.

RP-HPLC (ELSD trace, Method B, t_R = 22.4 min)

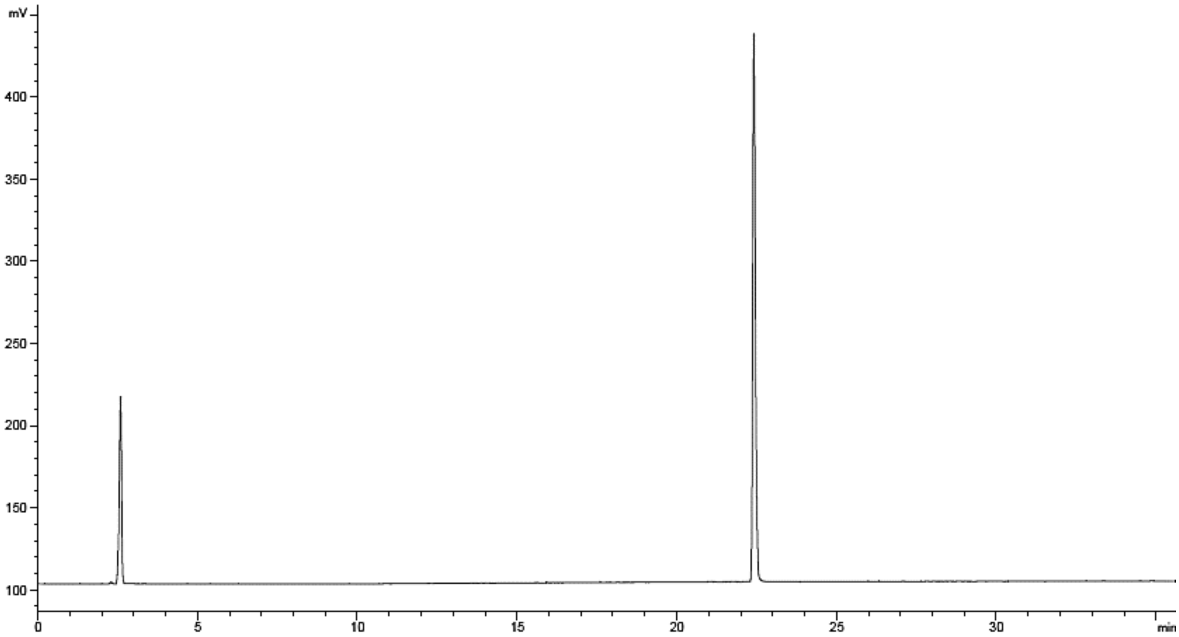

^1H NMR (D_2O)

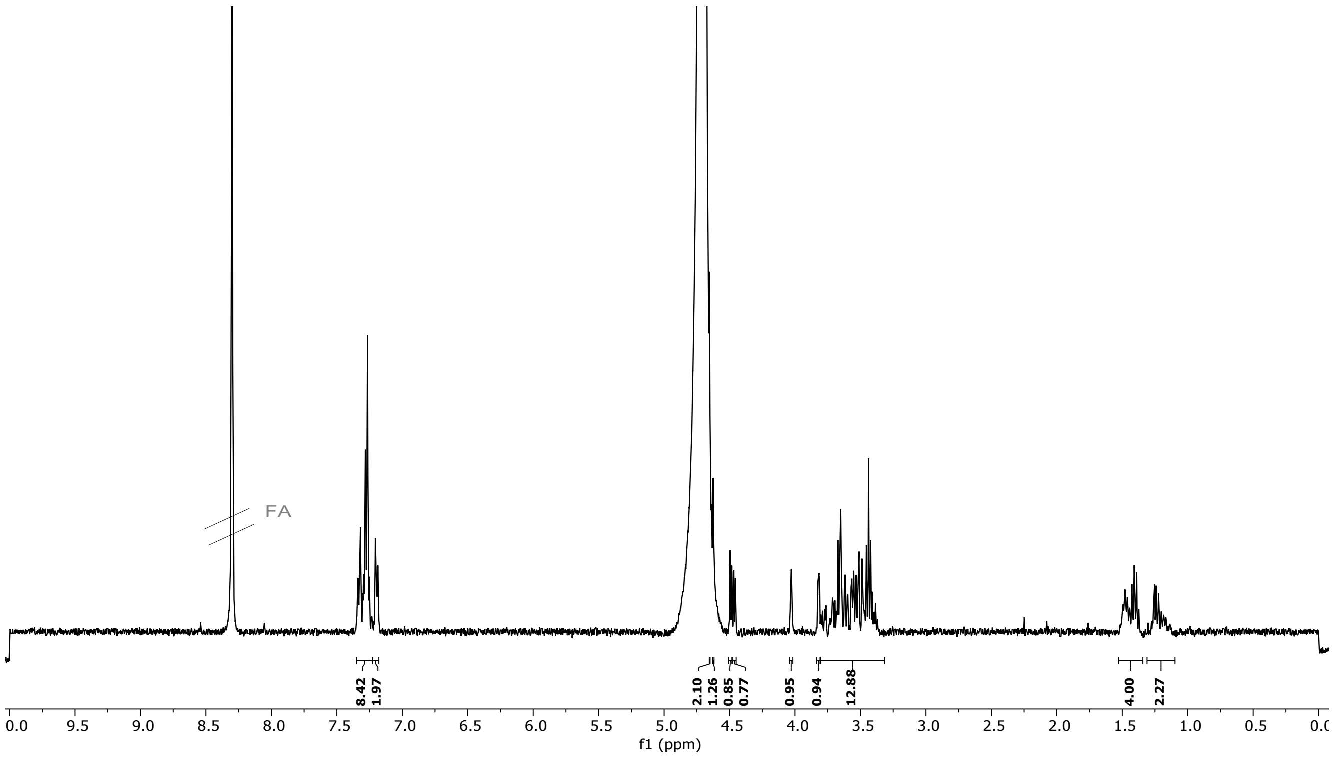

^{13}C NMR (D_2O)

Could not be obtained due to low amount of material. Carbon peaks are reported in respect to coupled ^1H–^{13}C HSQC NMR

HSQC (D_2O)

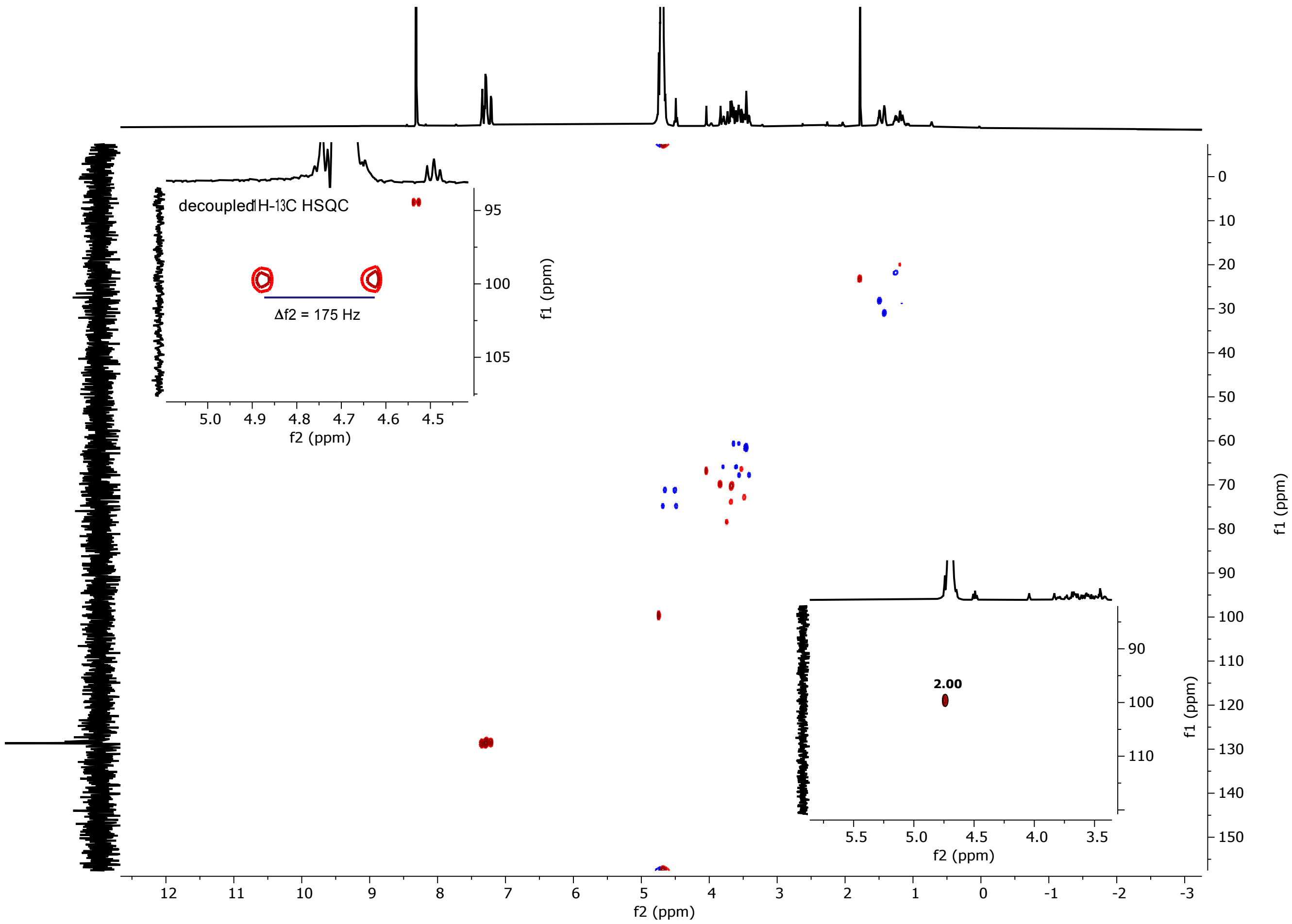

5-Hydroxypentyl β-D-galactopyranosyl-(1→6)-3,4-di-*O*-benzyl-α-D-mannopyranoside (16)

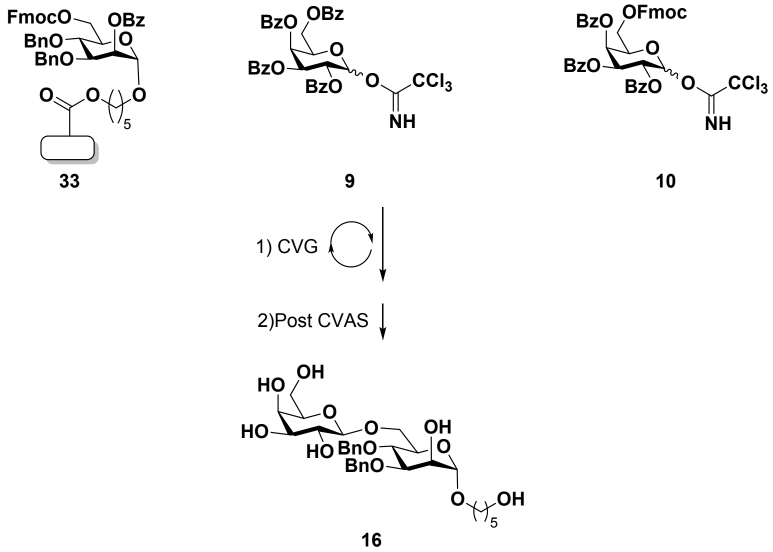

Action	BB	Modules	Notes	Result (mg, µmol, yield)
Method A				
CVG		A	**2** swell; Fmoc-deprotection	
	**9**	B; C; D	Activator solution A, 1.00 mL	0.35 mg, 0.58 µmol, 26%
Post CVG		F1		
Method B				
CVG		A	**2** swell; Fmoc-deprotection	
	**10**	B; C; D; C; D	Activator solution A, 1.00 mL	0.17 mg, 0.28 µmol, 15%
Post CVG		F1		

^{1}H NMR (600 MHz, D_2O): δ = 7.37 – 7.27 (m, 10H), 4.78 (m, 3H), 4.57 – 4.50 (m, 2H), 4.22 (d, *J* = 7.8 Hz, 1H), 4.07 – 3.97 (m, 3H), 3.83 – 3.43 (m, 14H), 2.67 (d, *J* = 11.0 Hz, 1H), 1.54 – 1.42 (m, 4H), 1.35 – 1.24 (m, 2H) ppm; ^{13}C NMR (151 MHz, D_2O): δ = 128.5, 128.4, 127.1, 103.0, 99.4, 78.5, 75.1, 74.9, 73.3, 72.6, 70.8, 70.5, 70.0, 67.7, 67.6, 66.4, 60.8, 30.7, 27.0, 21.7 ppm (in respect to the coupled ^{1}H–^{13}C HSQC NMR); ESI-HRMS: m/z [M+Na]+ calcd. for $C_{31}H_{44}O_{12}Na$: 631.2724 found 631.2754.

RP-HPLC (ELSD trace, Method B, t_R = 22.2 min)

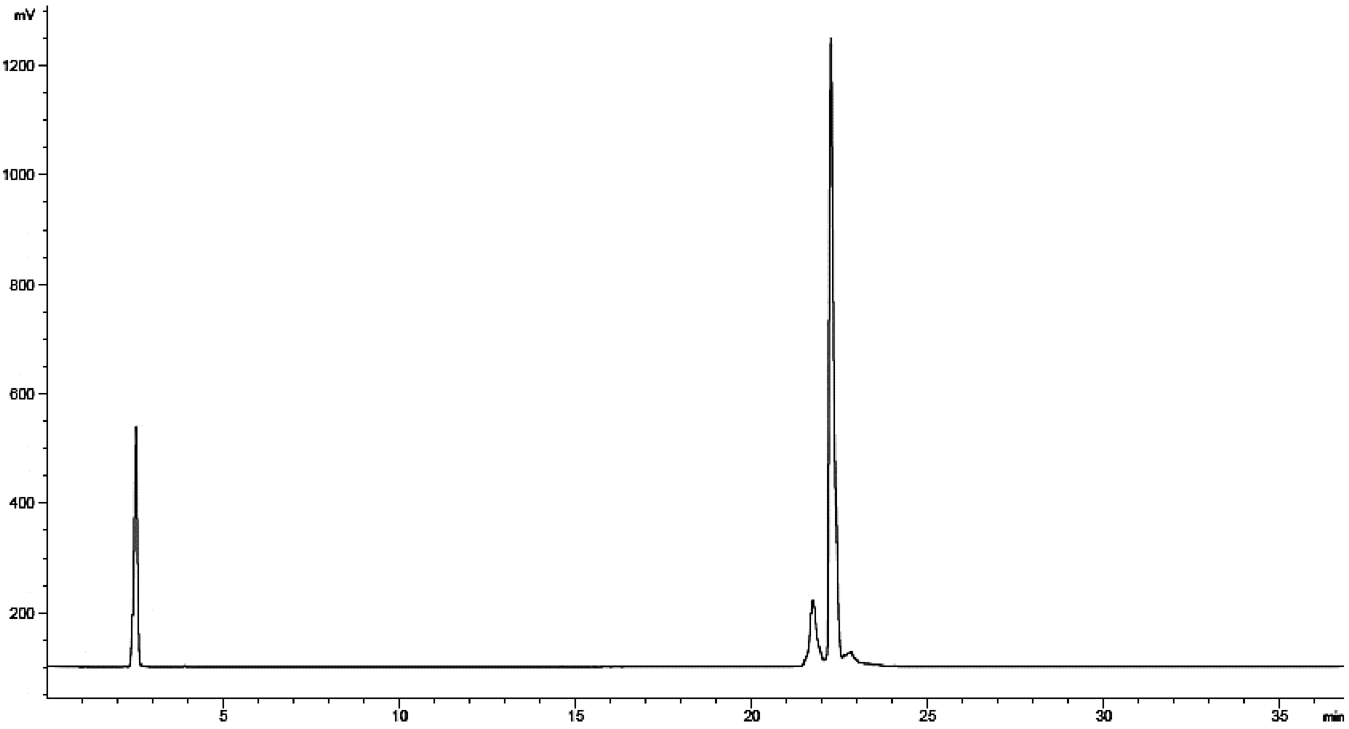

^{1}H NMR (D_2O)

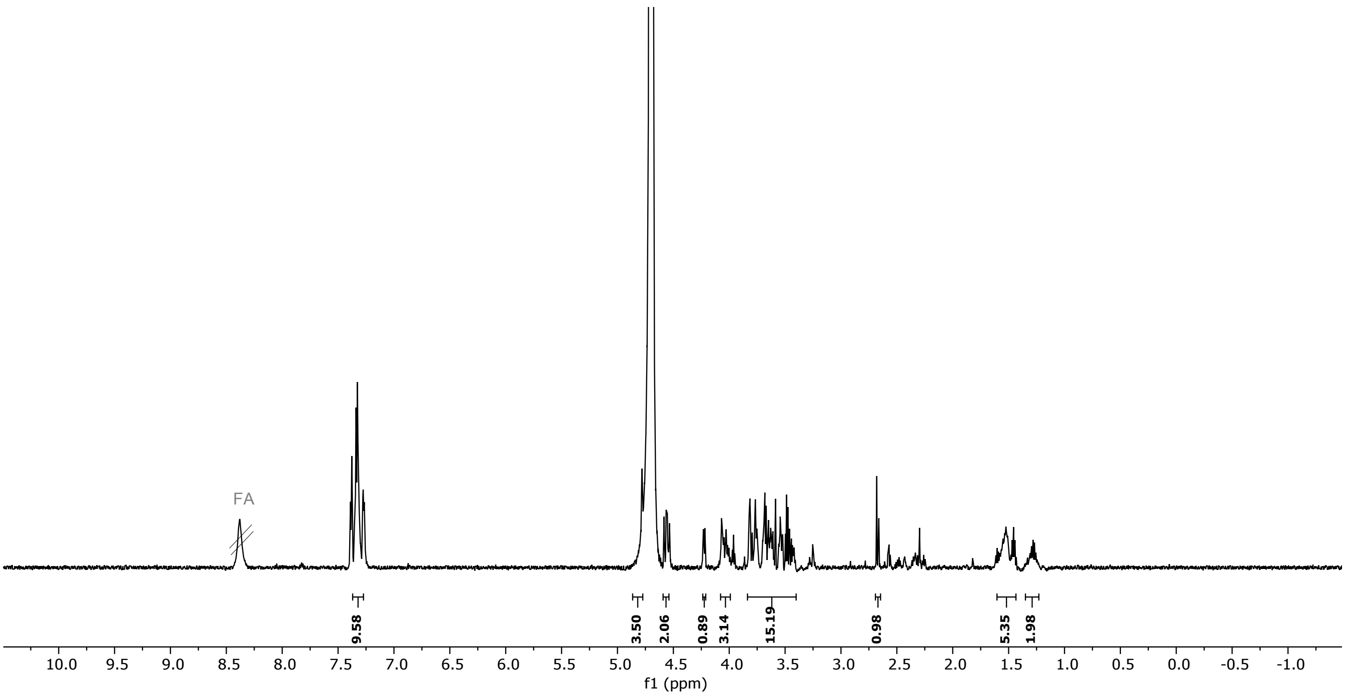

^{13}C NMR (D_2O)

Could not be obtained due to low amount of material. Carbon peaks are reported in respect to coupled ^{1}H–^{13}C HSQC NMR

HSQC (D_2O)

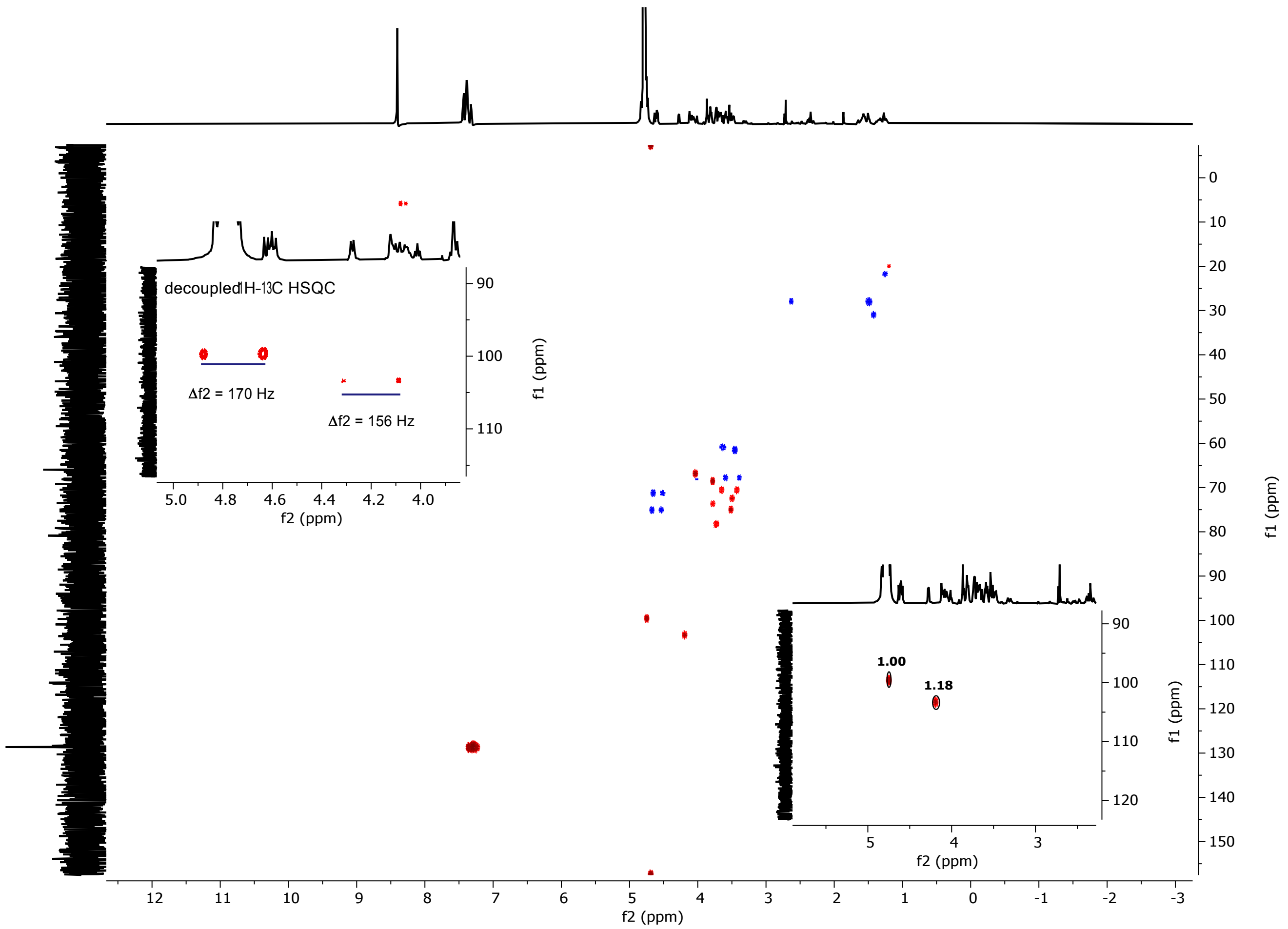

5-Hydroxypentyl β-D-glucopyranosyl-(1→6)-3,4-di-*O*-benzyl-α-D-mannopyranoside (17)

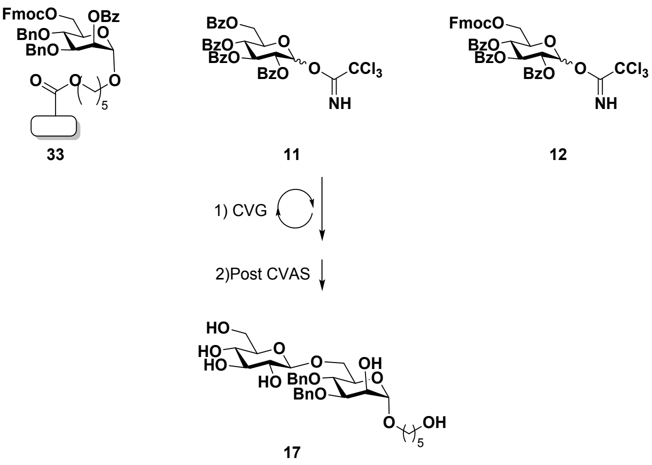

Action	BB	Modules	Notes	Result (mg, mmol, yield)
Method A				
CVG	11	A	**2** swell; Fmoc-deprotection	
		B; C; D	Activator solution A, 1.00 mL	0.29 mg,0.48 μmol, 20%
Post CVG		F1		
Method B				
CVG	12	A	**2** swell; Fmoc-deprotection	
		B; C; D; C; D	Activator solution A, 1.00 mL	0.08 mg,0.13 mmol, 16%
Post CVG		F1		

^{1}H NMR (600 MHz, D_2O): δ = 7.38 – 7.24 (m, 10H), 4.78 (m, 3H), 4.55 (t, J = 11.3 Hz, 2H), 4.27 (d, J = 7.9 Hz, 1H), 4.09 – 3.96 (m, 2H), 3.83 – 3.18 (m, 14H), 1.59 – 1.45 (m, 4H), 1.28 – 1.11 (m, 2H ppm); ^{13}C NMR (176 MHz, D_2O, D_2O): δ = 128.8, 128.6, 128.5, 102.6, 99.6, 78.2, 75.3, 75.3, 75.1, 73.3, 72.6, 70.9, 70.4, 66.7, 69.4, 69.2, 61.5, 30.6, 27.7, 21.7 ppm (in respect to the coupled ^{1}H–^{13}C HSQC NMR); ESI-HRMS: m/z [M+Na]$^+$ calcd. for $C_{31}H_{44}O_{12}Na$: 631.2724 found 631.2756.

RP-HPLC (ELSD trace, Method B, t_R = 22.3 min)

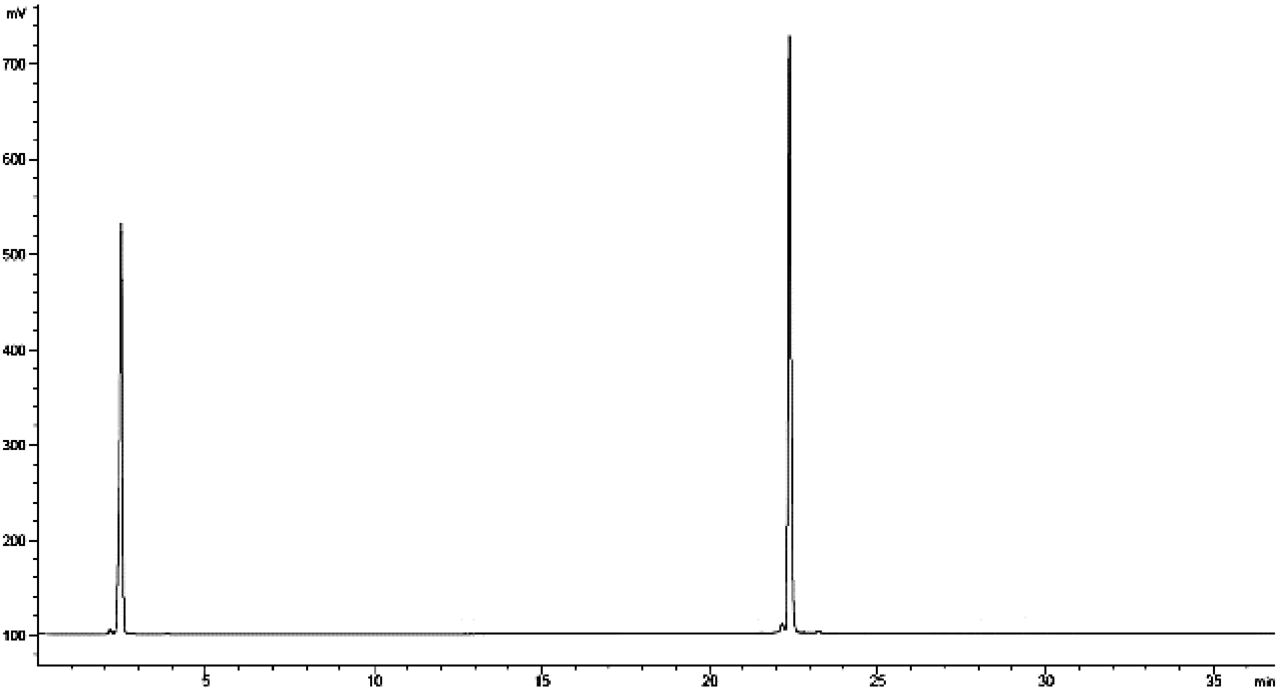

^1H NMR (D_2O)

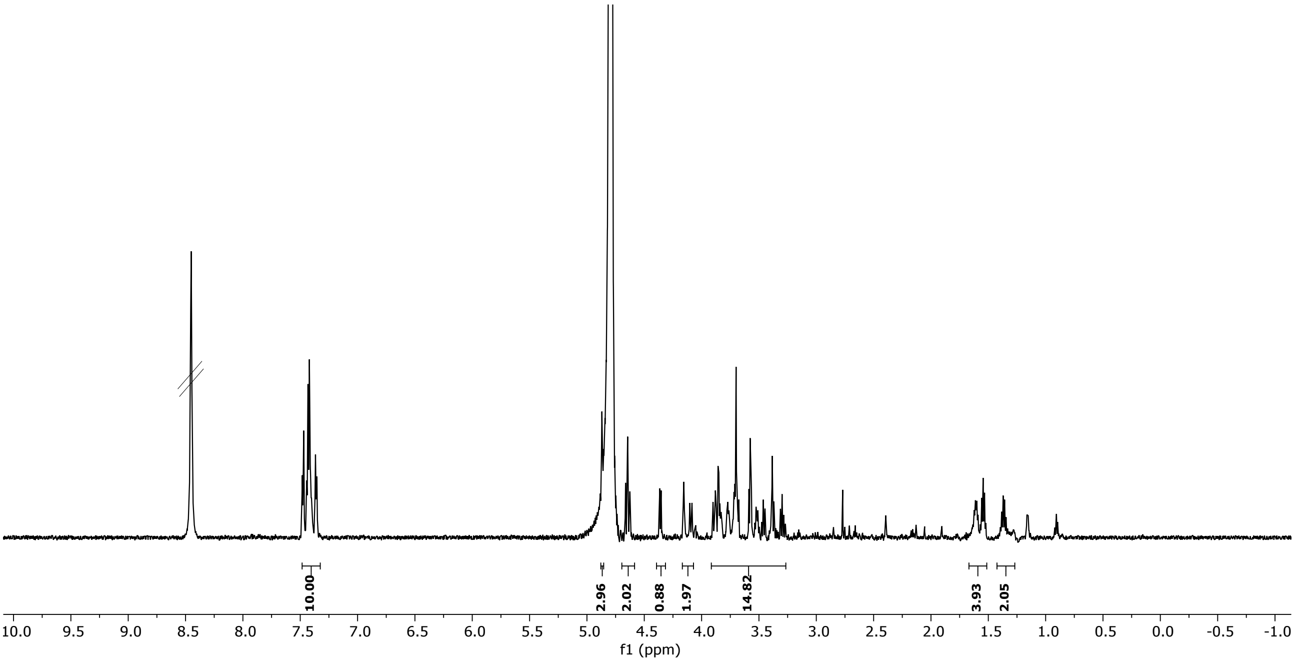

^{13}C NMR (D_2O)

Could not be obtained due to low amount of material. Carbon peaks are reported in respect to coupled ^1H–^{13}C HSQC NMR

HSQC (D_2O)

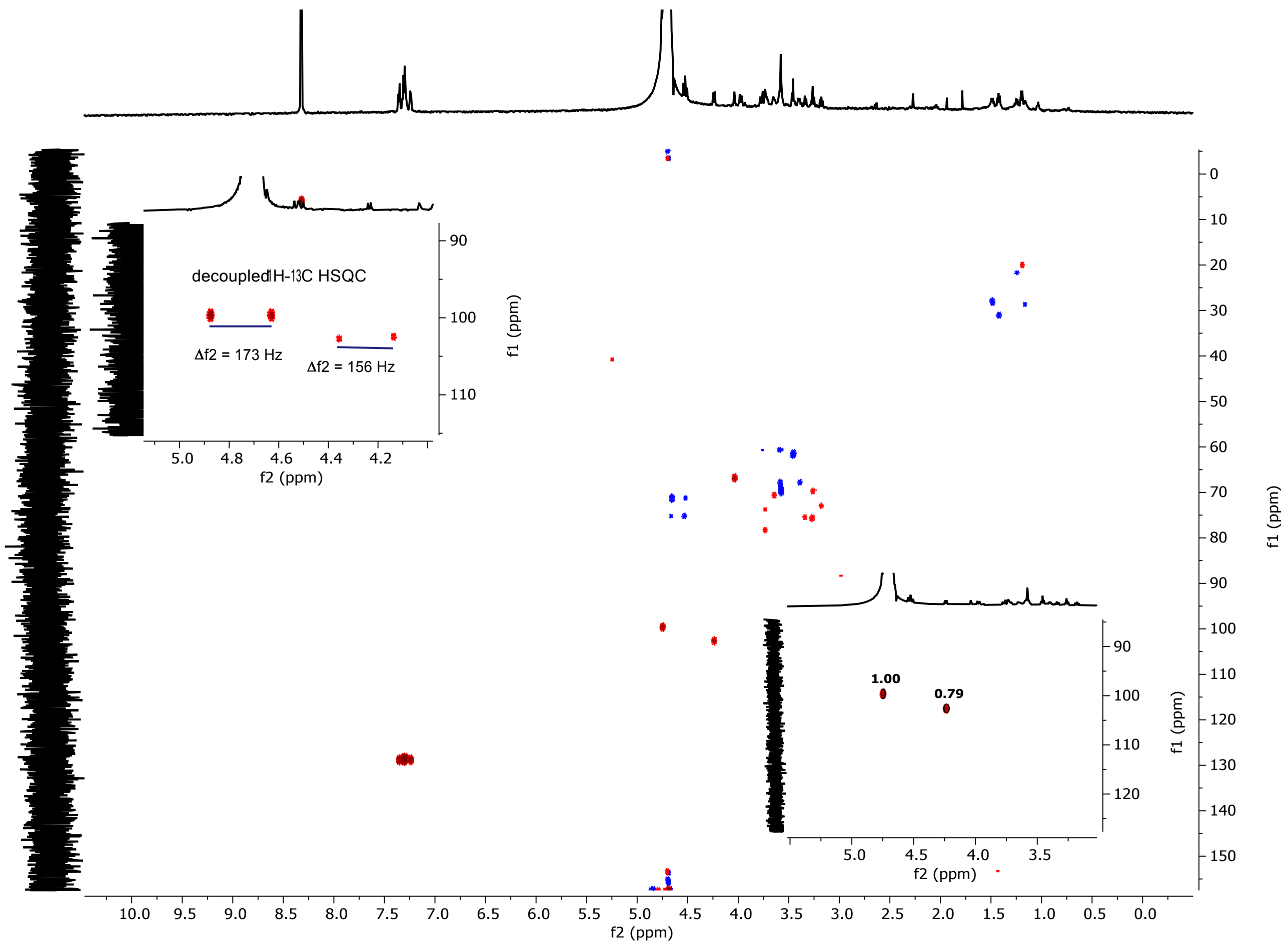

5-Hydroxypentyl α-D-mannopyranosyl (1→6)-α-D-mannopyranosyl-(1→6)-3,4-di-*O*-benzyl-α-D-mannopyranoside (18)

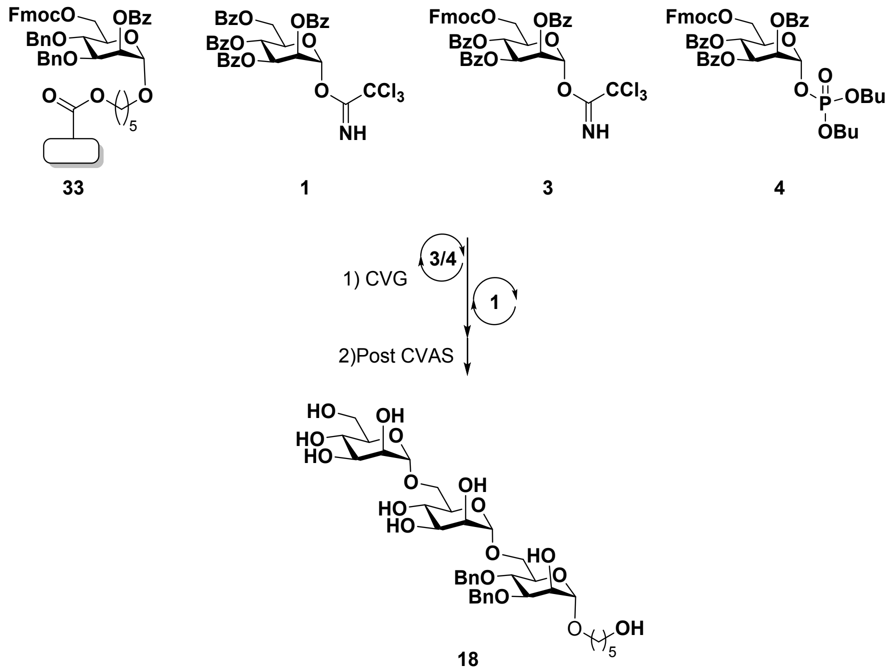

Action	BB	Modules	Notes	Result (mg, μmol, yield)
Method A				
		A	**2** swell; Fmoc-deprotection	
CVG	**3**	B; C; D; C; D, E,	Activator solution A, 1.00 mL	0.49 mg, 0.64 μmol, 30%
	**1**	B; C; D	Activator solution A, 1.00 mL	
Post CVG		F1		
Method B				
		A	**2** swell; Fmoc-deprotection	
CVG	**4**	B; C; D; C; D, E,	Activator solution A, 1.00 mL	0.4 mg, 0.52 μmol, 21%
	**1**	B; C; D	Activator solution A, 1.00 mL	
Post CVG		F1		
Method C				
		A	**2** swell; Fmoc-deprotection	
CVG	**3**	B; C; D; C; D, E,	Activator solution B, 1.00 mL	0.49 mg, 0.64 μmol, 32%
	**1**	B; C; D	Activator solution B, 1.00 mL	
Post CVG		F1		
Method D				
		A	**2** swell; Fmoc-deprotection	
CVG	**3**	B; C; D; C; D, E,	Activator solution C, 1.00 mL	0.34 mg, 0.44 μmol, 25%
	**1**	B; C; D	Activator solution C, 1.00 mL	
Post CVG		F1		

^{1}H NMR (600 MHz, D_2O): δ = 7.49 – 7.32 (m, 10H), 4.85 (d, *J* = 11.2 Hz, 4H), 4.61 (dd, *J* = 23.0, 11.2 Hz, 2H), 4.17 (s, 1H), 3.97 (d, *J* = 2.7 Hz, 1H), 3.91 – 3.53 (m, 21H), 1.65 – 1.57 (m, 2H), 1.55 (q, *J* = 7.2 Hz, 2H), 1.43 – 1.31 (m, 2H), 1.16 (d, *J* = 6.4 Hz, 1H) ppm; ^{13}C NMR (151 MHz, D_2O) δ = 170.9, 137.1, 137.0, 131.1, 128.7, 128.6, 128.6, 128.3, 128.3, 128.2, 99.99, 99.6, 99.3, 78.3, 74.7, 73.7, 72.5, 71.1, 70.9, 70.7, 70.4, 69.9, 69.8, 69.7, 67.7, 66.8, 66.6, 66.3, 65.9, 65.9, 65.3, 61.5, 60.8, 30.9, 28.1, 21.7 ppm; ESI-HRMS: m/z $[M+Na]^+$ calcd. for $C_{37}H_{54}O_{17}Na$: 793.325318 found 793.3313.

RP-HPLC (ELSD trace, Method B, t_R = 20.8 min)

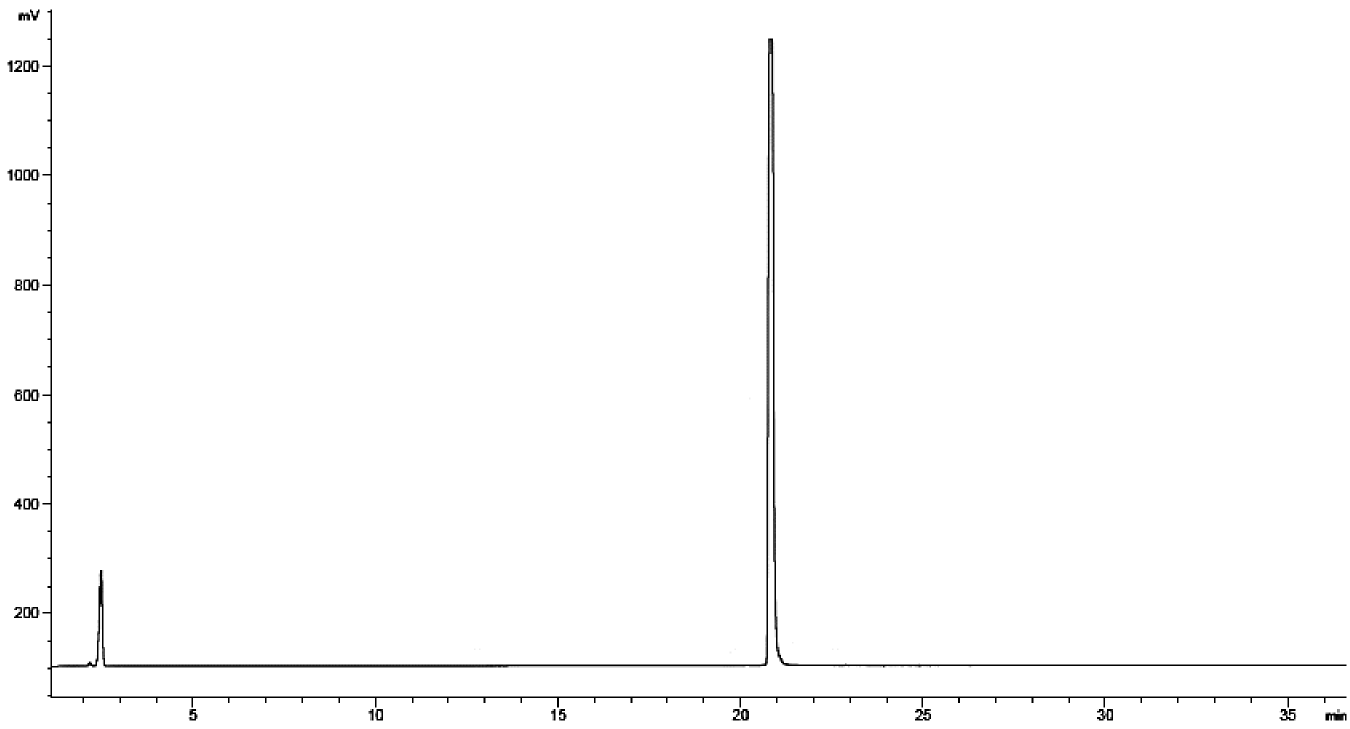

^{1}H NMR (D_2O)

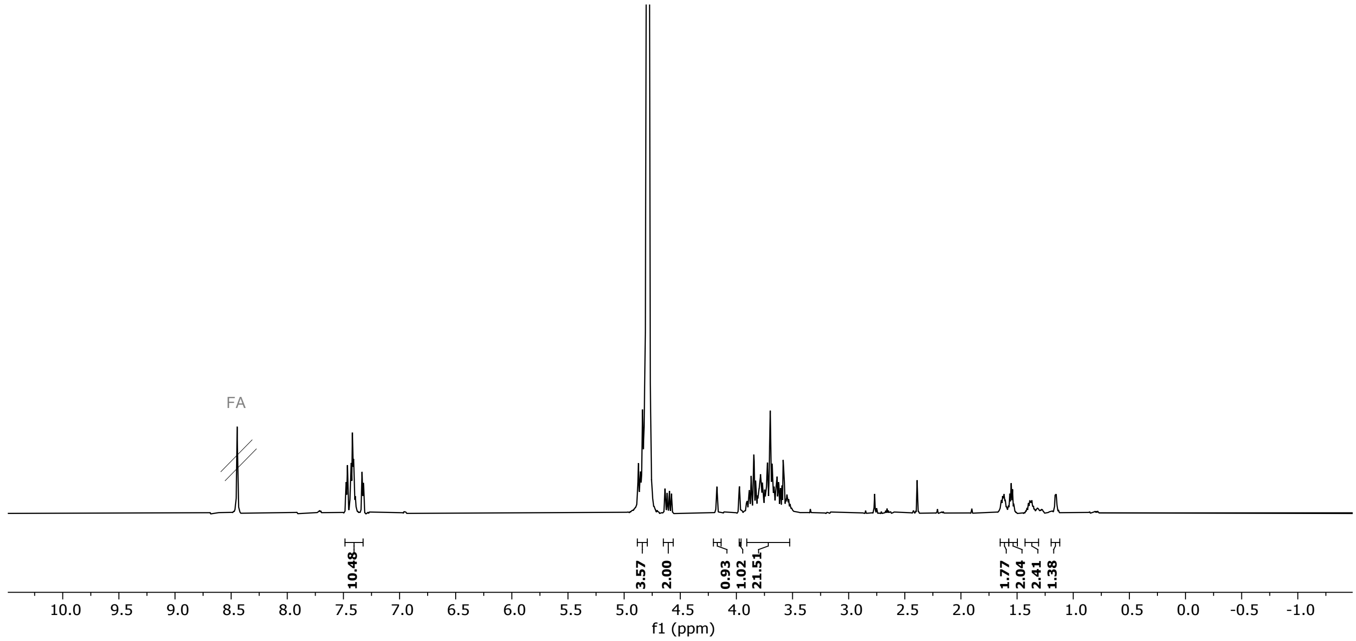

^{13}C NMR (D_2O)

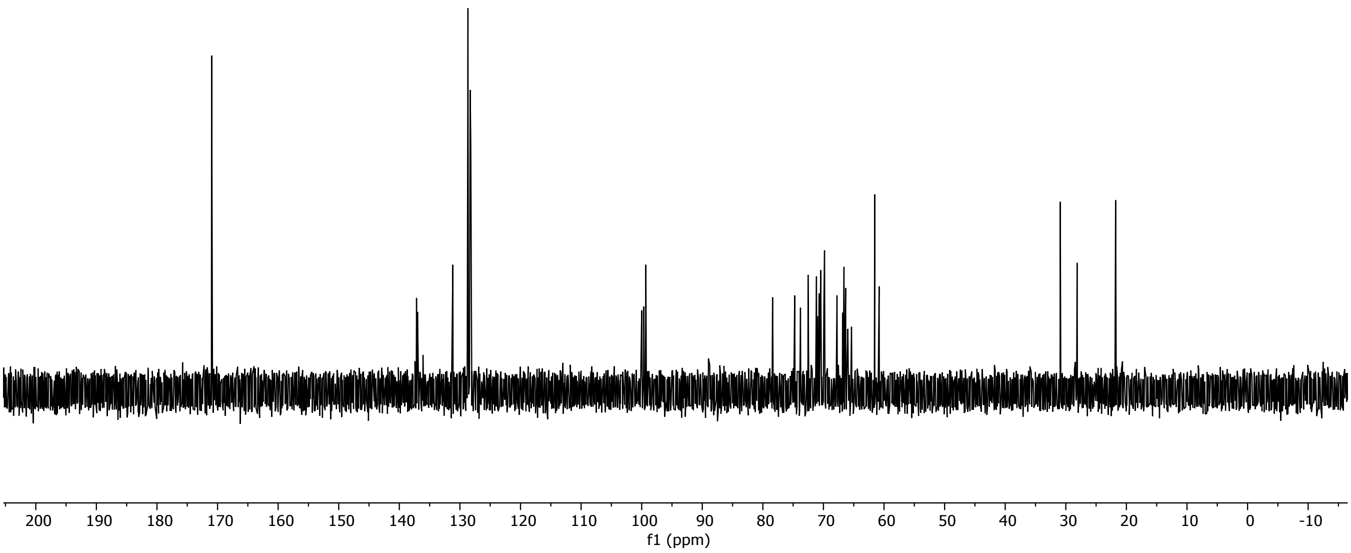

HSQC (D_2O)

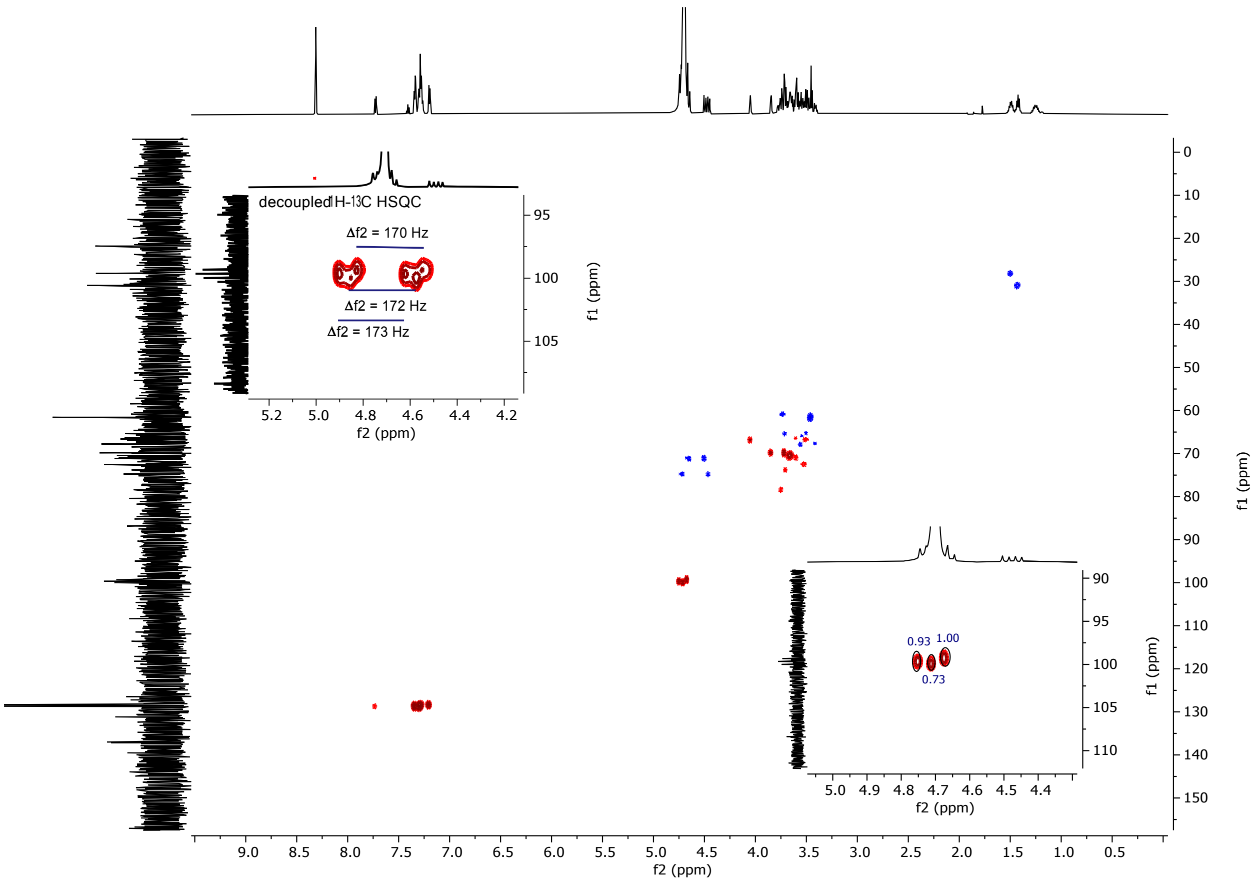

5-Hydroxypentyl α-D-mannopyranosyl (1→6)-α-D-mannopyranosyl-(1→6)-α-D-mannopyranosyl-(1→6)-3,4-di-*O*-benzyl-α-D-mannopyranoside (19)

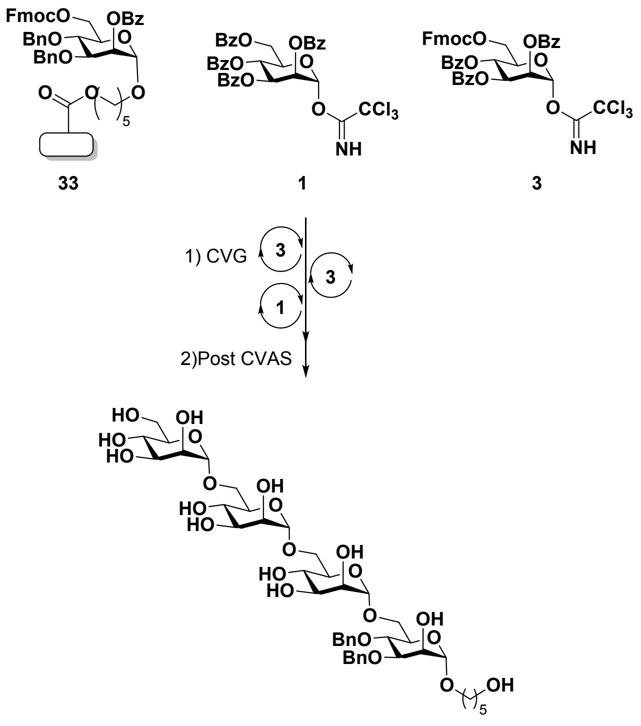

Action	BB	Modules	Notes	Result (mg, μmol, yield)
CVG		**A**	**2** swell; Fmoc-deprotection<br>4 cm² membrane	0.22 mg, 0.24 μmol, 8% overall yield
	**3**	B; C; D; C; D, E,	Activator solution B, 1.00 mL	
	**3**	B; C; D; C; D, E,	Activator solution B, 1.00 mL	
	**1**	B; C; D	Activator solution B, 1.00 mL	
Post CVG		F1		

^{1}H NMR (700 MHz, D2O): δ = 7.45 – 7.25 (m, 10H), 4.87 – 4.82 (m, 4H), 4.74 (m, 2H), 4.57 (dd, *J* = 24.6, 11.2 Hz, 2H), 4.14 (t, *J* = 2.1 Hz, 1H), 3.92 (d, *J* = 14.0 Hz, 2H), 3.88 – 3.46 (m, 26H), 1.62 – 1.47 (m, 4H), 1.40 – 1.14 (m, 3H) ppm; ^{13}C NMR (176, D_2O): δ = 128.7, 128.3, 128.1, 99.8, 99.2, 99.2, 78.2, 74.2, 73.6, 72.3, 70.6, 70.1, 69.9, 69.7, 67.8, 66.7, 66.6, 65.4, 65.1, 61.2, 60.7, 30.6, 28.1, 21.80 ppm (in respect to the coupled ^{1}H–^{13}C HSQC NMR); ESI-HRMS: m/z [M+Na]$^+$ calcd. for $C_{43}H_{64}O_{22}Na$: 955.3781 found 955.3873 .

RP-HPLC (ELSD trace, Method B, t_R = 20.2 min)

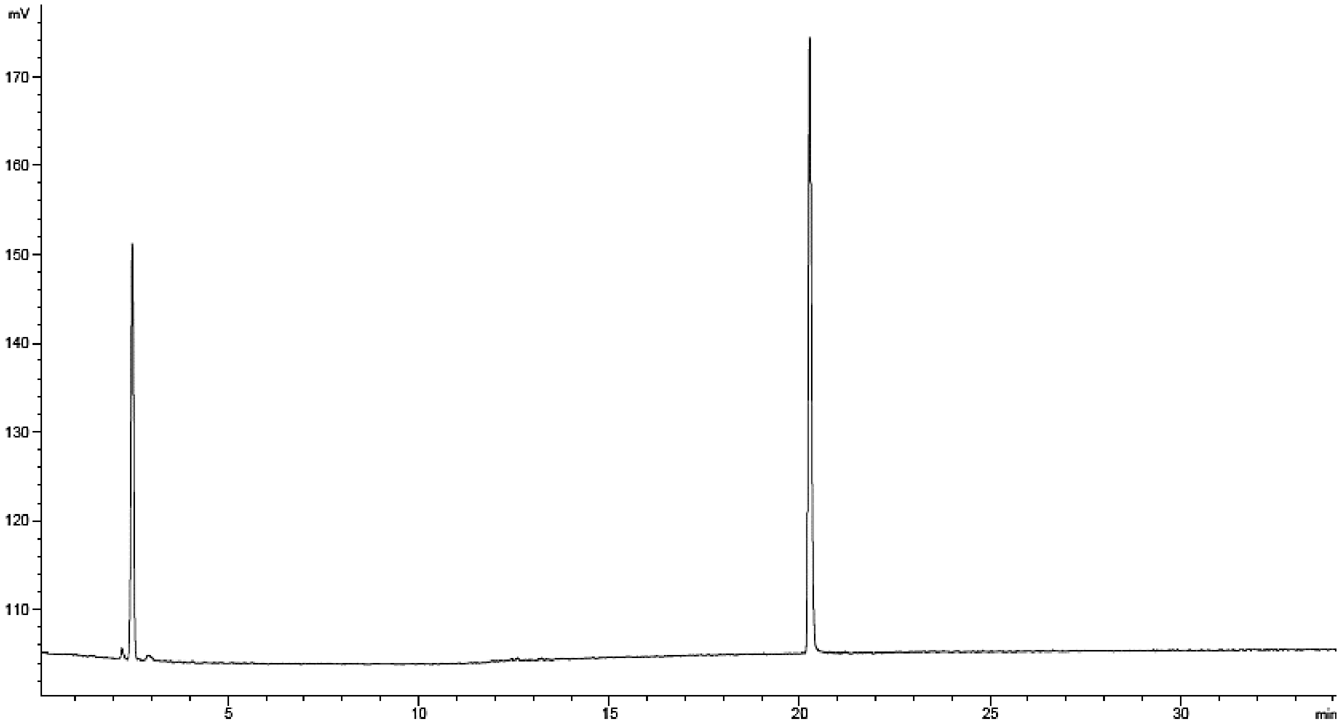

^1H NMR (D_2O)

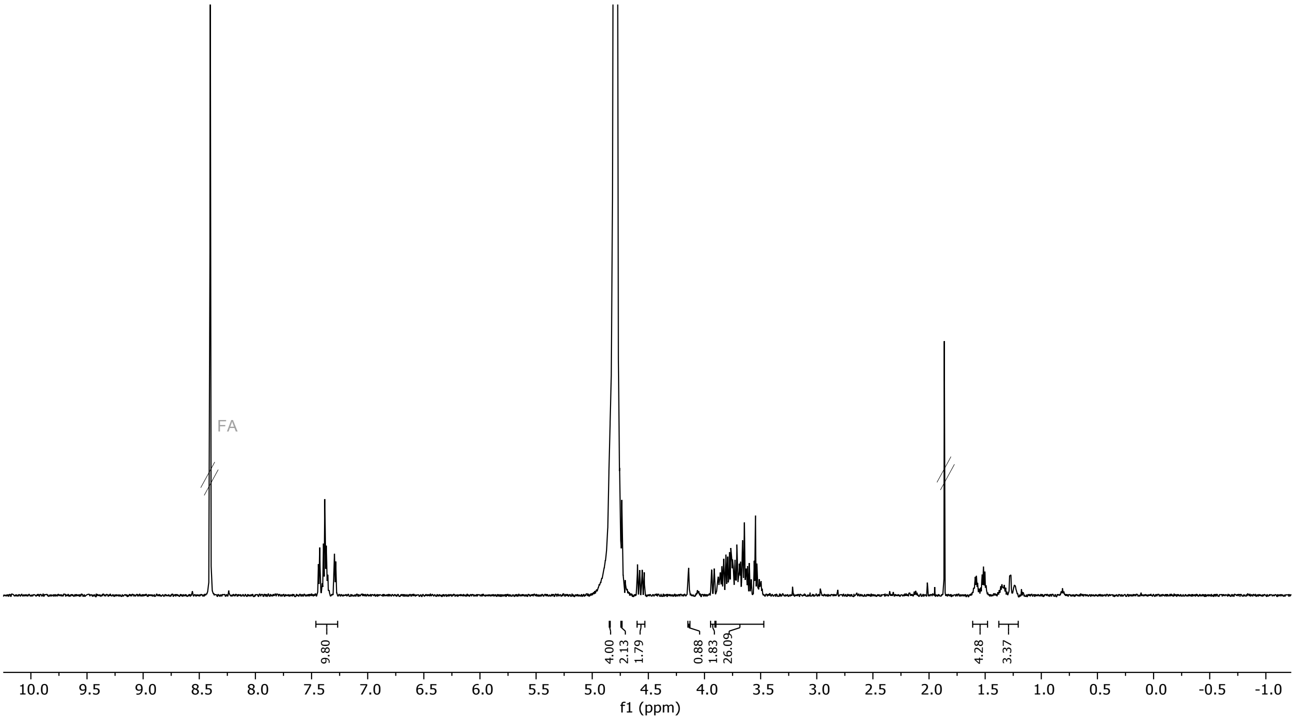

^{13}C NMR (D_2O)

Could not be obtained due to low amount of material. Carbon peaks are reported in respect to coupled ^1H–^{13}C HSQC NMR

HSQC (D_2O)

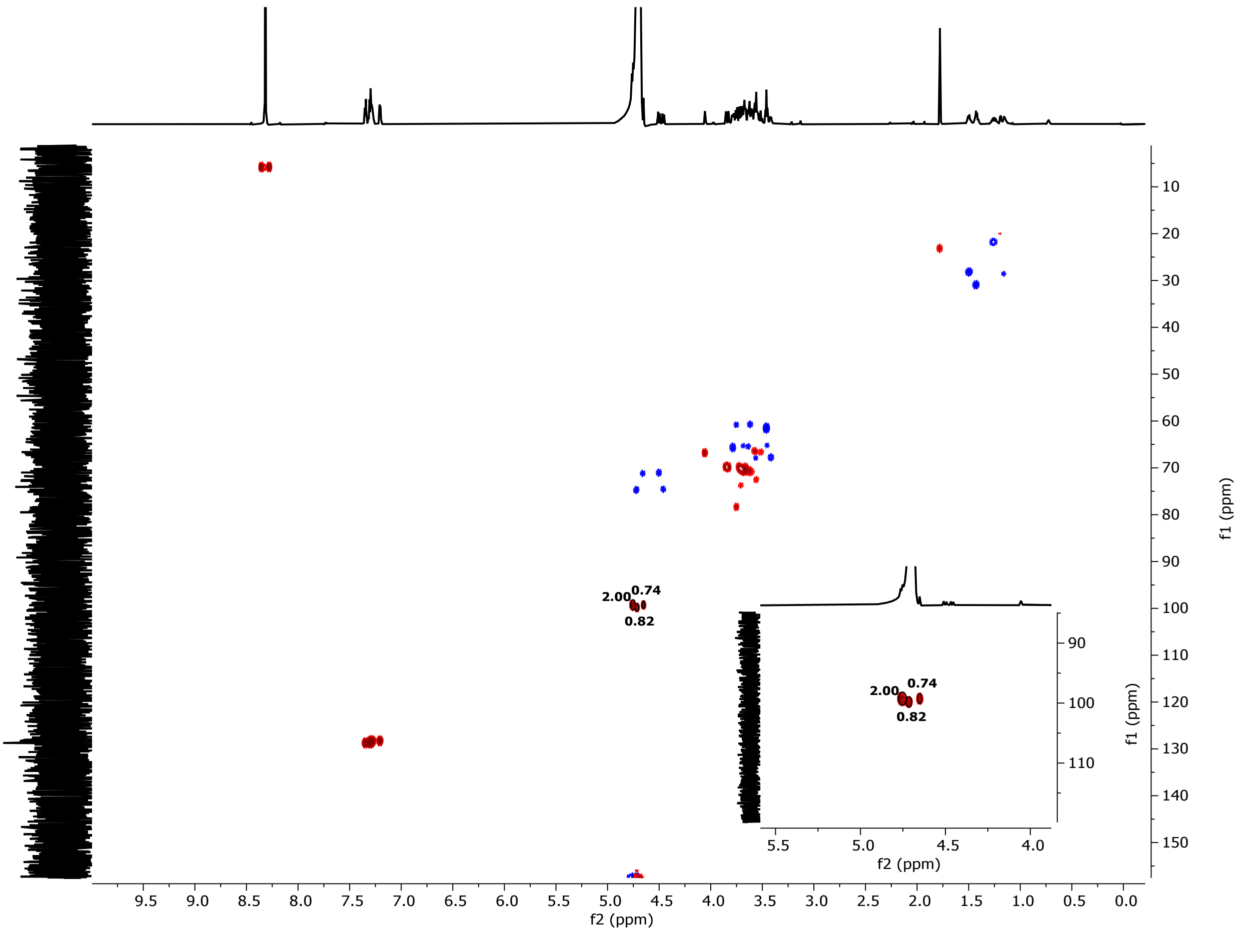

5-Hydroxypentyl β-d-galactopyranosyl (1→4)- β-d-glucopyranosyl-(1→6)-3,4-di-O-benzyl-α-d-mannopyranoside (20)

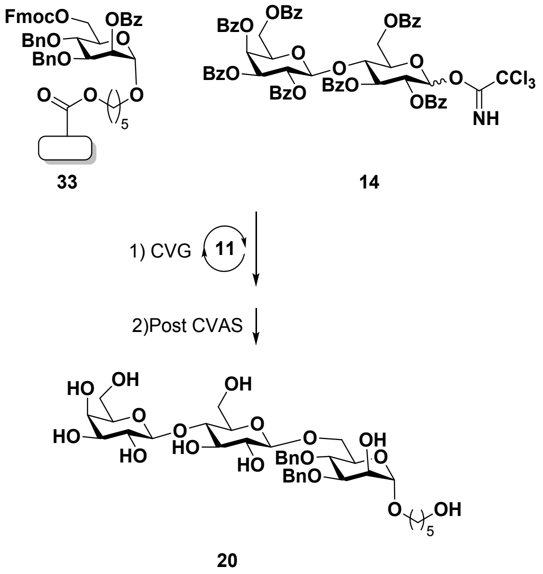

Action	BB	Modules	Notes	Result (mg, µmol, yield)
Method A				
CVG		A	**2** swell; Fmoc-deprotection	
	**14**	B; C; D; C; D	Activator solution B, 1.00 mL	0.37 mg, 0.48 µmol, 34%
Post CVG		F1		

^{1}H NMR (600 MHz, D2O): δ = 7.42 (tt, *J* = 33.7, 7.0 Hz, 10H), 4.86 (s, 1H), 4.76 – 4.70 (m, 2H), 4.66 – 4.61 (m, 2H), 4.43 (d, *J* = 7.9 Hz, 1H), 4.39 (d, *J* = 7.9 Hz, 1H), 4.15 (d, *J* = 4.9 Hz, 1H), 4.08 (d, *J* = 11.2 Hz, 1H), 3.97 – 3.90 (m, 2H), 3.88 – 3.79 (m, 4H), 3.79 – 3.73 (m, 3H), 3.73 – 3.68 (m, 2H), 3.67 – 3.60 (m, 3H), 3.57 (t, *J* = 6.6 Hz, 2H), 3.52 (dt, *J* = 15.0, 7.5 Hz, 3H), 3.34 (t, *J* = 8.3 Hz, 1H), 1.61 (m, 2H), 1.53 (m, 2H), 1.36 (m, 2H) ppm; ^{13}C NMR (176 MHz, D_2O) δ = 170.9, 137.1, 136.9, 128.7, 128.7, 128.6, 128.4, 128.3, 102.8, 102.4, 99.6, 78.2, 75.2, 75.1, 74.6, 74.1, 73.8, 72.6, 72.42, 71.27, 70.8, 70.5, 68.4, 68.1, 67.8, 61.5, 60.9, 59.9, 30.9, 28.0, 21.7 ppm; ESI-HRMS: m/z $[M+Na]^+$ calcd. for $C_{37}H_{54}O_{17}Na$: 793.3253 found 793.3307.

RP-HPLC (ELSD trace, Method B, t_R = 22.3 min)

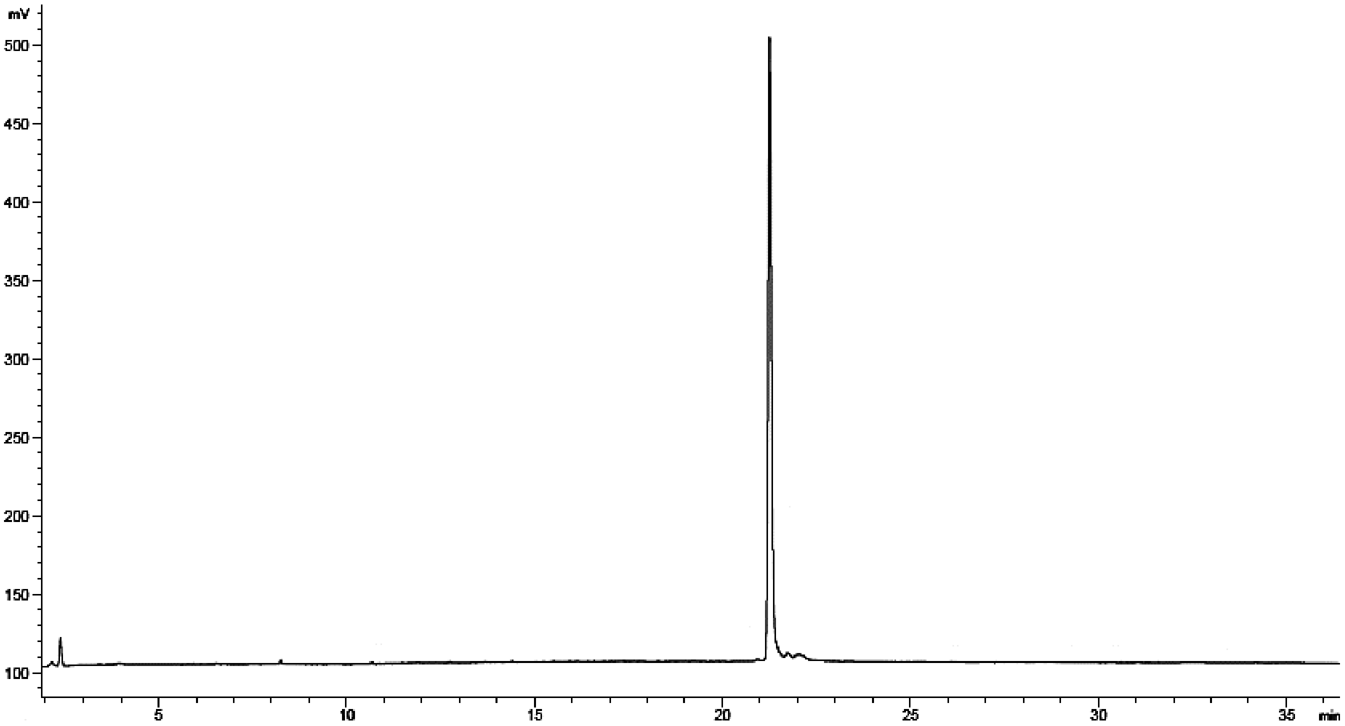

^{1}H NMR (D_2O)

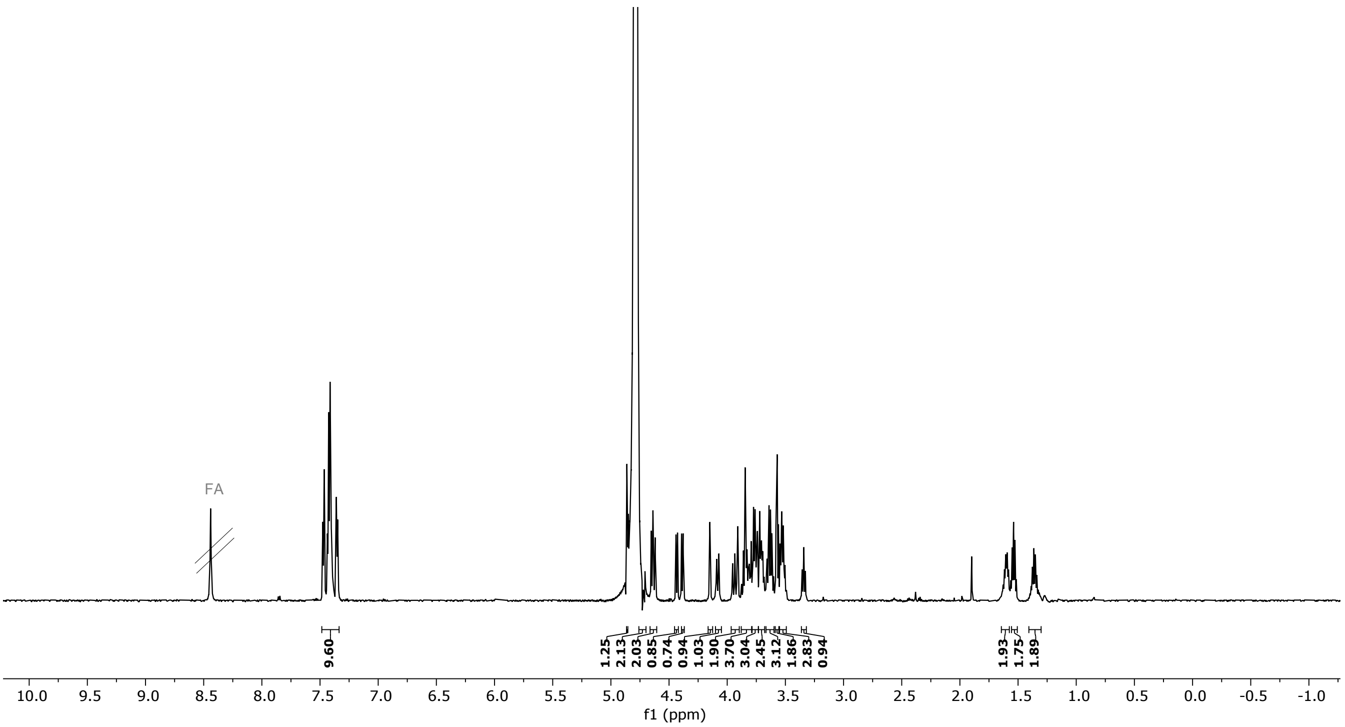

^{13}C NMR (D_2O)

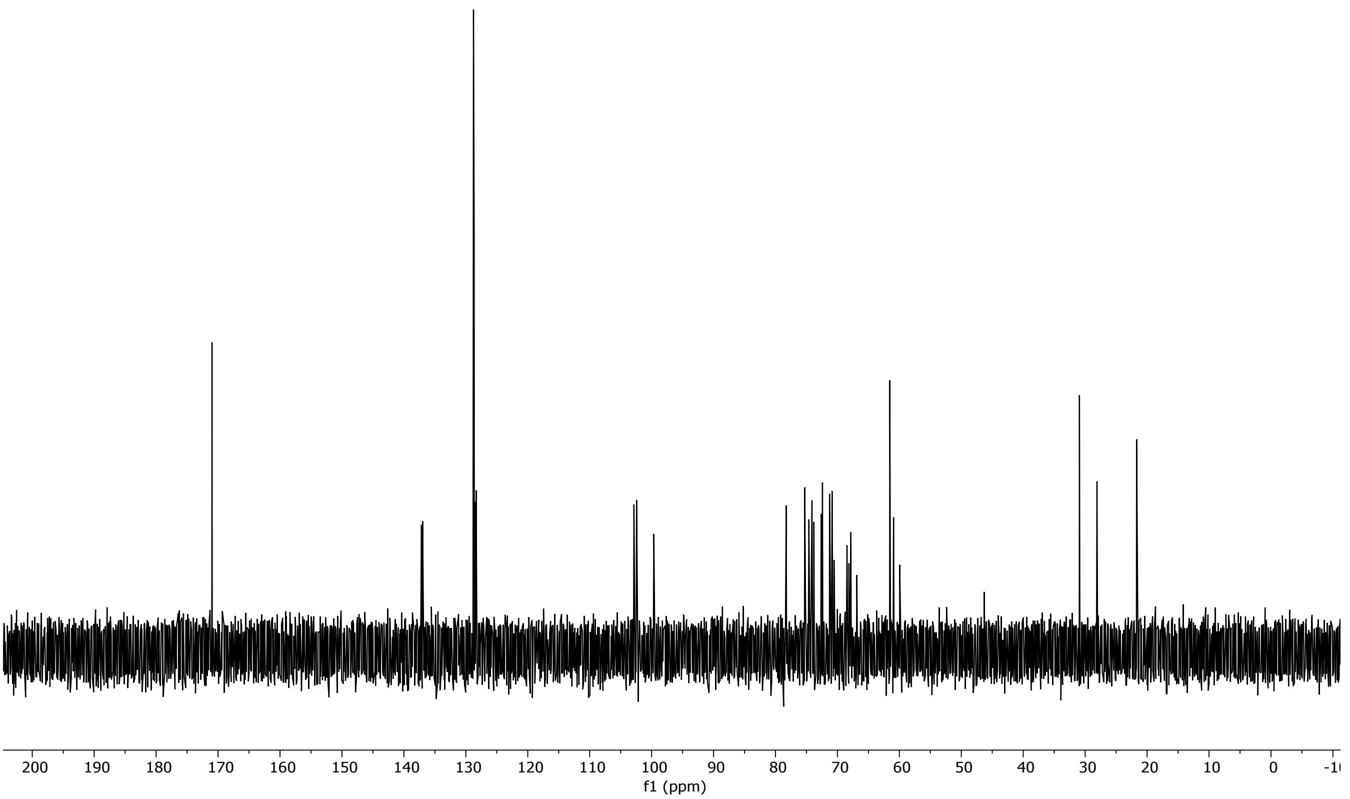

HSQC (D_2O)

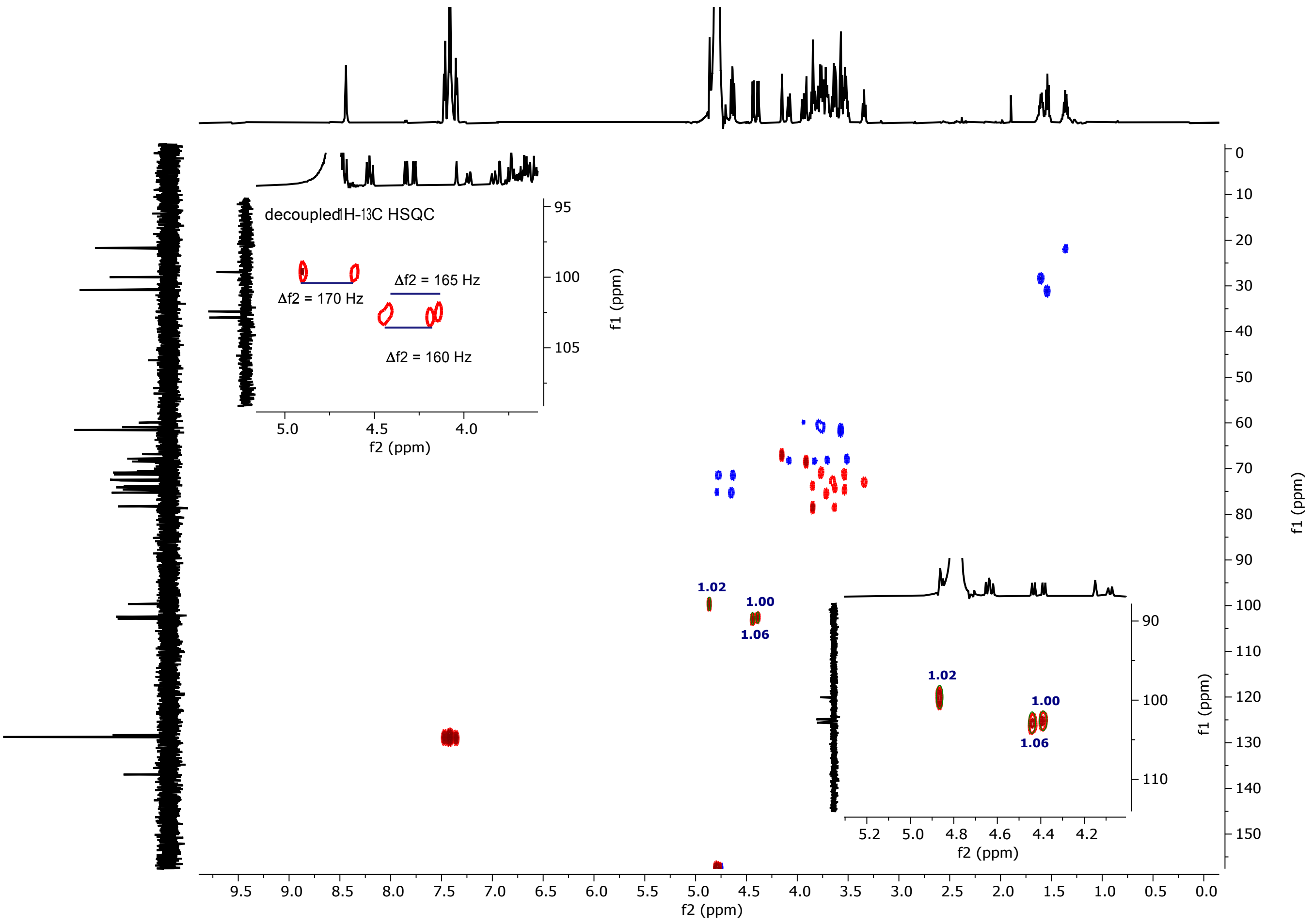
decoupled 1H-13C HSQC
Δf2 = 170 Hz
Δf2 = 165 Hz
Δf2 = 160 Hz
1.02
1.00
1.06
f2 (ppm)
f1 (ppm)

5-Hydroxypentyl 3,4-di-*O*-benzyl-α-D-mannopyranosyl-(1→6)-3,4-di-*O*-benzyl-α-D-mannopyranoside (36)

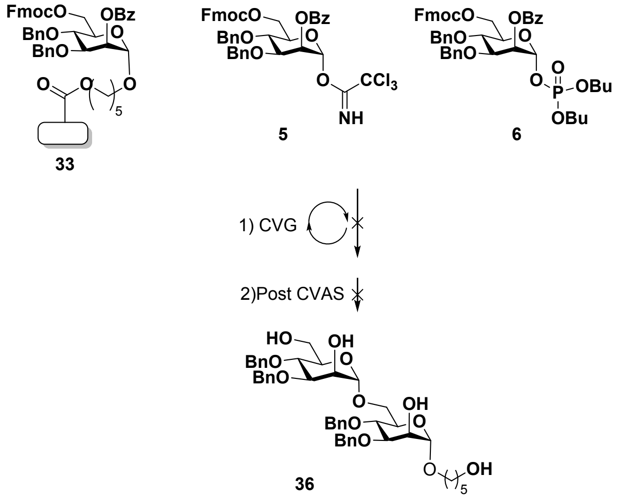

Action	BB	Modules	Notes	Result (mg, mmol, yield)
Method A				
CVG	5	A	**2** swell; Fmoc-deprotection	--
		B; C; D; C; D	Activator solution A, 1.00 mL	
Post CVG		F1		
Method B				
CVG	6	A	**2** swell; Fmoc-deprotection	--
		B; C; D; C; D	Activator solution A, 1.00 mL	
Post CVG		F1		

The desired product **36** was not obtained. The glycosylation reaction for BBs **5** and **6** were performed using 8% TMSOTf activator solution and 6.00 equiv of BB/glycosylation.

H. Parallel synthesis

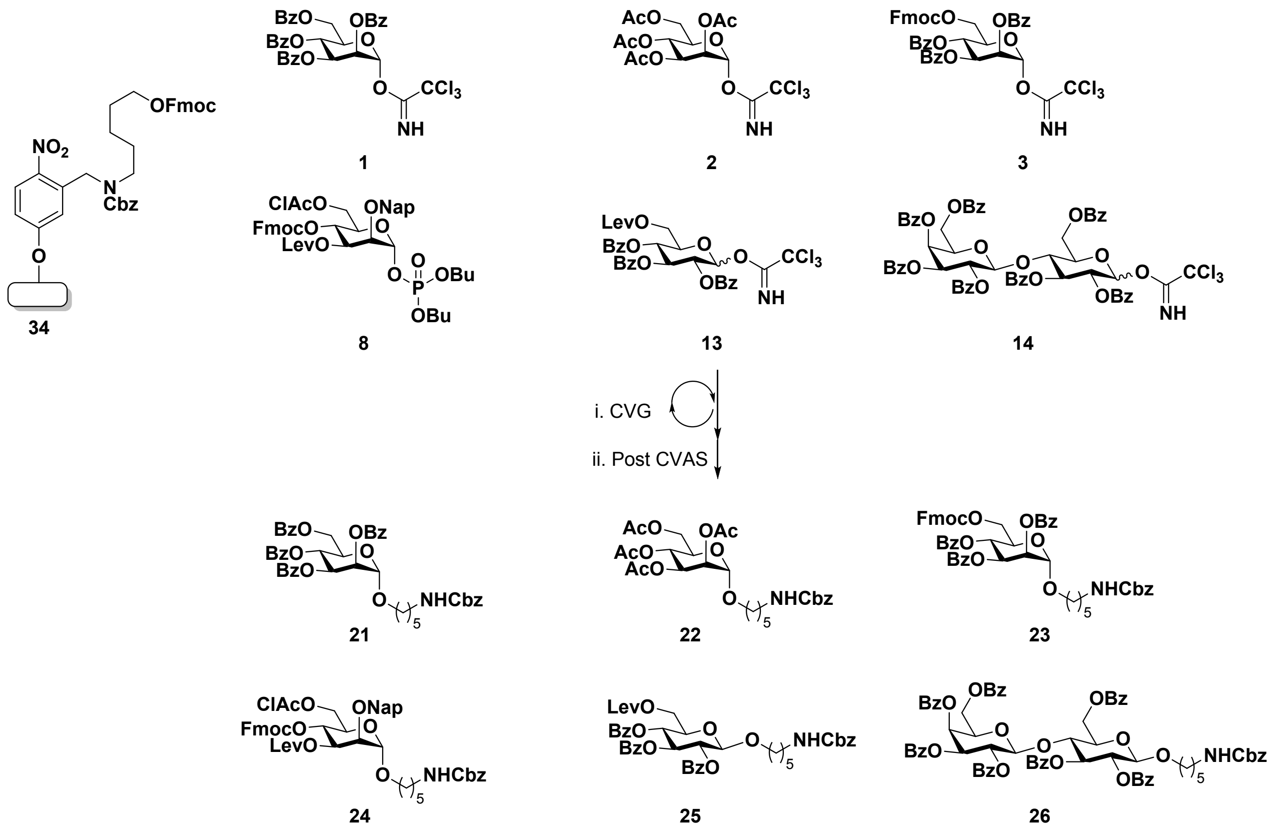

To perform this high-throughput experiment we used six different building blocks (**1-3**, **8**, **13**, **14**) having different molecular weight, and we synthesized a traceless photo-labile linker **29**. For this specific experiment, six different membrane pieces **34** (1 cm^2 each) with the exact same functionalization bearing the photo-labile linker, were placed after in six different Schlenk flasks. The chosen building blocks spotted in the corresponding membranes in separate flasks while for glycosylation all membranes were placed inside the glycosylation chamber having equal distances (≈ 1-1.5 cm) (Figure S9). Between the first and the second glycosylation, the membranes were placed in different positions with different neighboring membranes to detect any possible diffusion between the spotted glycosyl donors. After UV-cleavage and MALDI-detection of the synthesized residues, we observed no diffusion during the two glycosylation steps and successful synthesis of the desired structures. Further purification and characterization was not performed. MALDI analysis showed that in the case of the peracetylated mannose trichloroacetimidate **2** cleavage of one Ac- group and as a result, monomer and dimer formation, but we cannot identify which one. Bellow we attain an indicative structure to justify the observed mass.

Action	BB	Modules	Notes	Result
CVG		A, B	**35** swell, 6 pieces in 50 mL Schlenk flask	
	Each membrane transferred in a different flask for spotting			
	**1**	C; D; C; D	Activator solution B, 0.60 mL	**21**
	**2**	C; D; C; D	Activator solution B, 0.60 mL	**22**
	**3**	C; D; C; D	Activator solution B, 0.60 mL	**23**
	**8**	C; D; C; D	Activator solution B, 0.60 mL	**24**
	**13**	C; D; C; D	Activator solution B, 0.60 mL	**25**
	**14**	C; D; C; D	Activator solution B, 0.60 mL	**26**
Post CVG		F2	in parallel	

Experimental setup/explanation

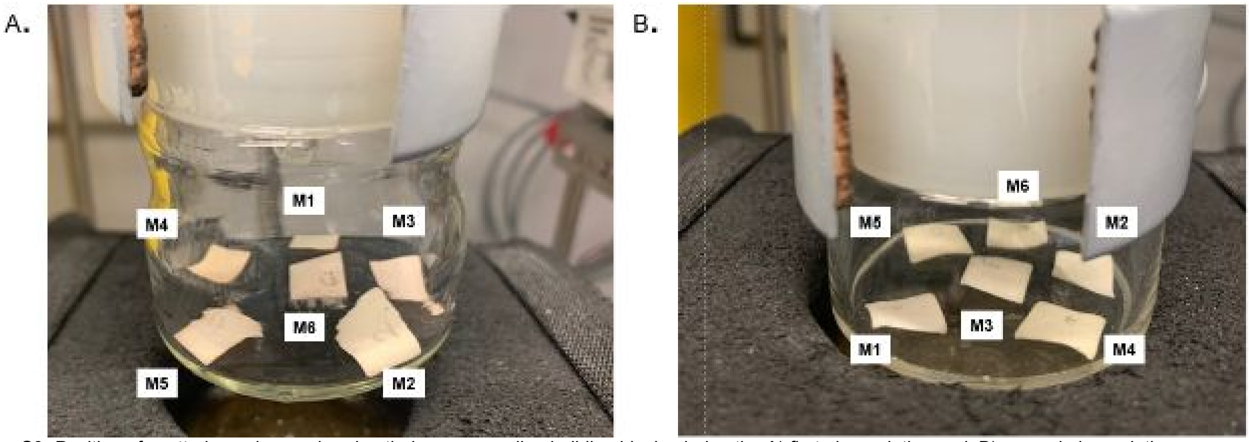

Figure S9: Position of spotted membranes bearing their corresponding building blocks during the A) first glycosylation and, B) second glycosylation.

MALDI-TOF of **21**

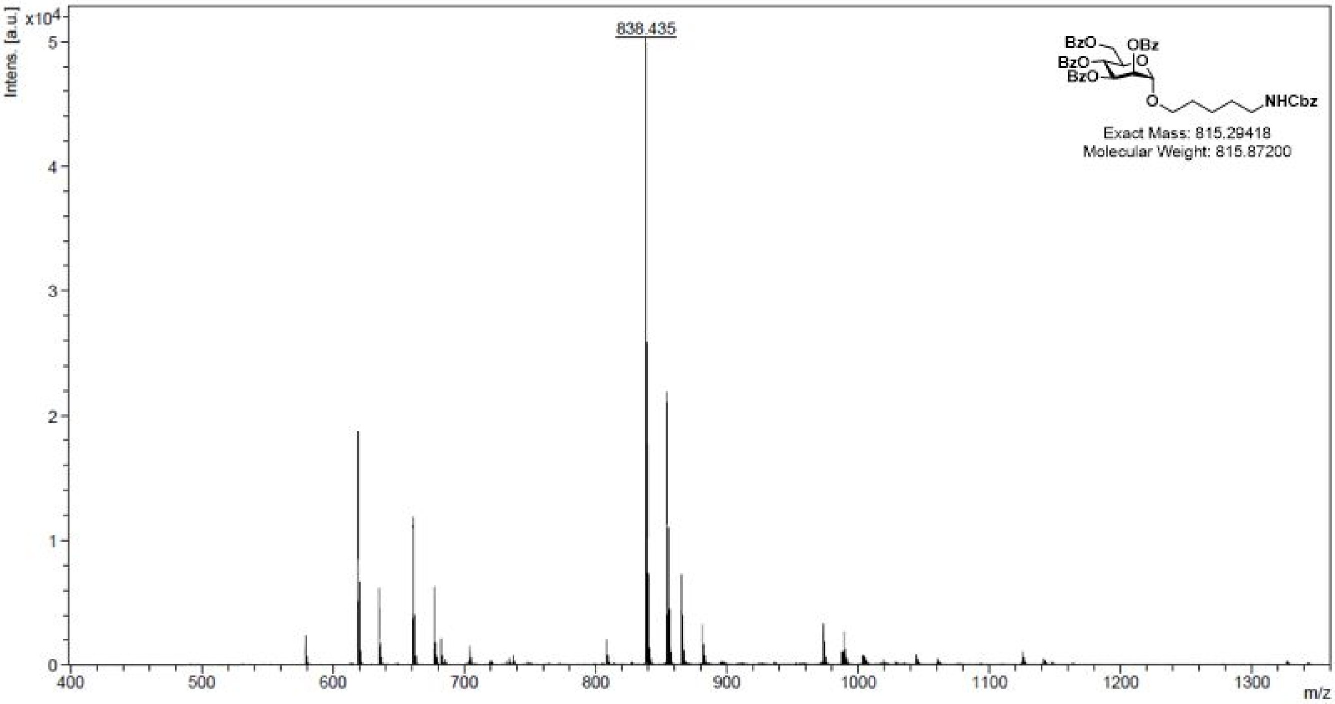

MALDI-TOF of **22**

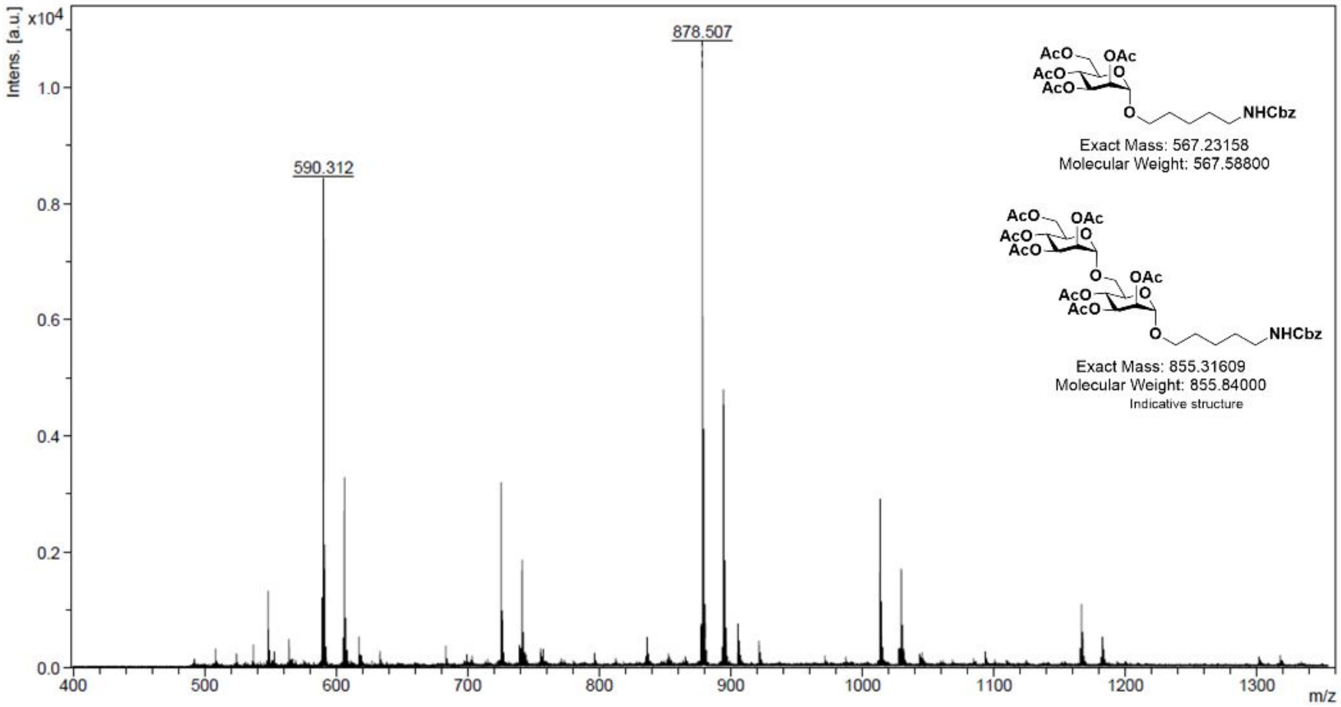

MALDI-TOF of **23**

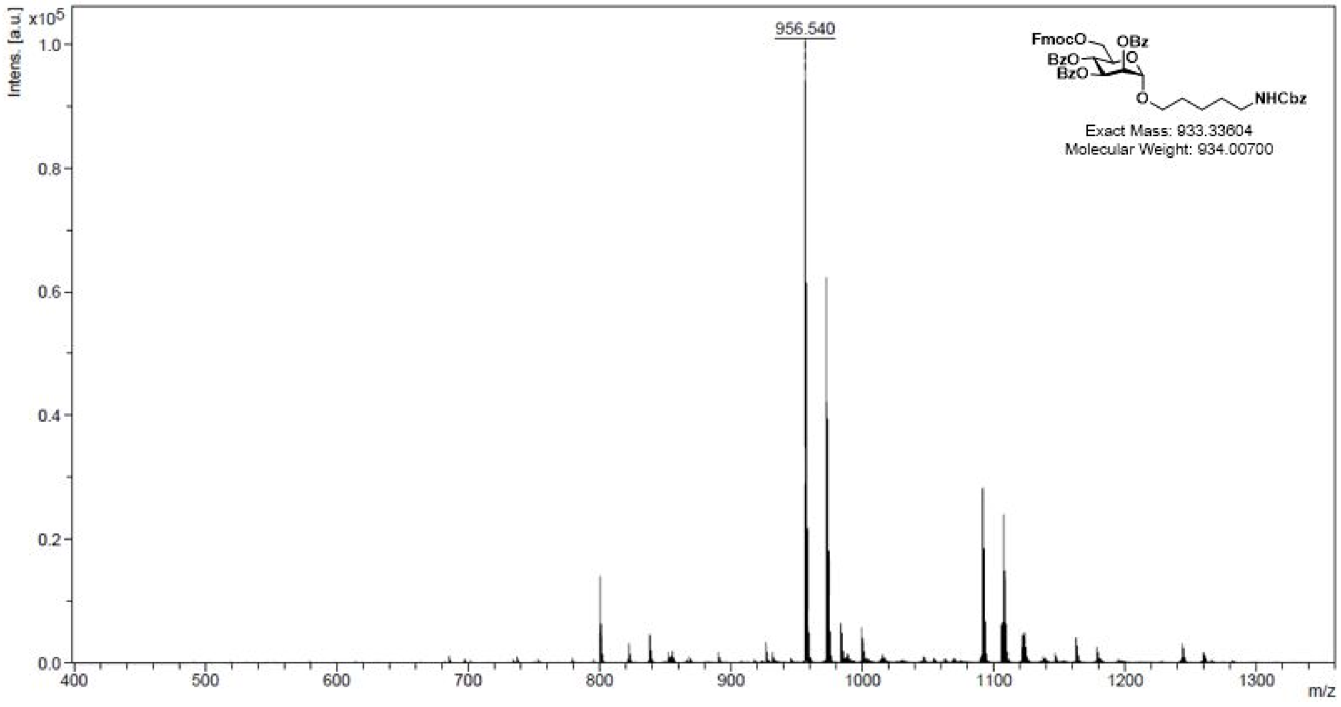

MALDI-TOF of **24**

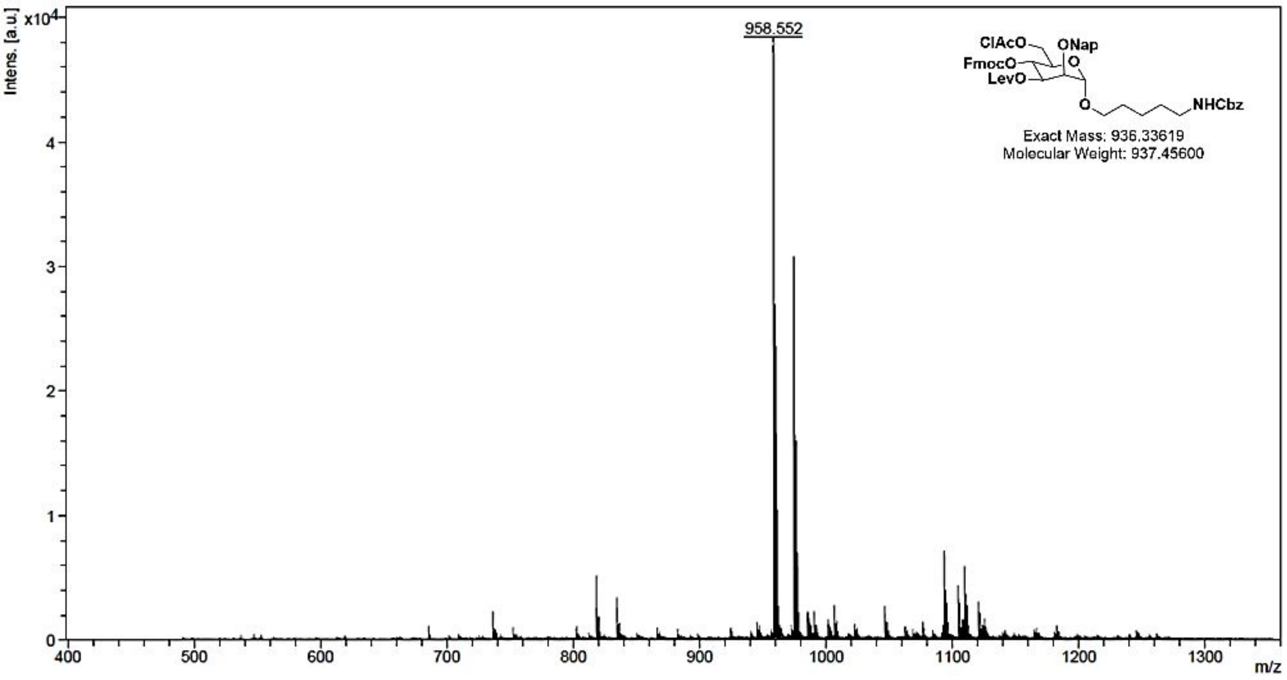

MALDI-TOF of **25**

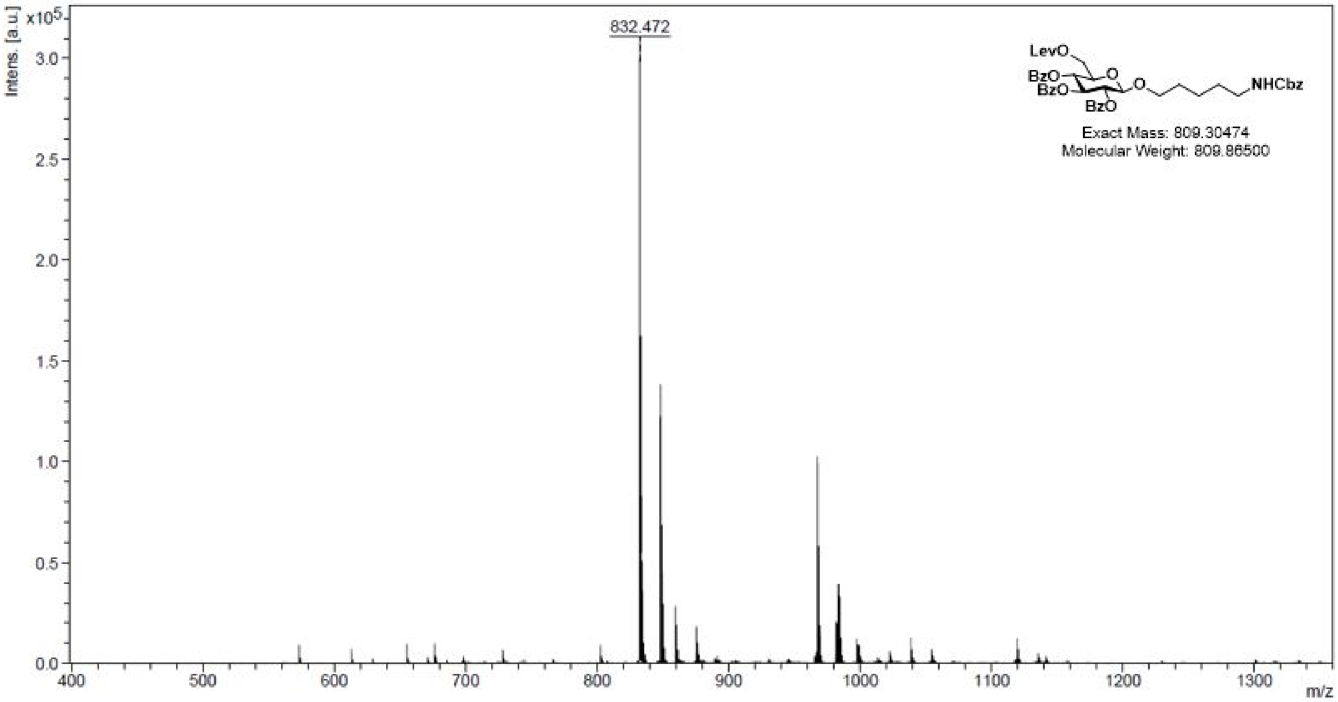

MALDI-TOF of **26**

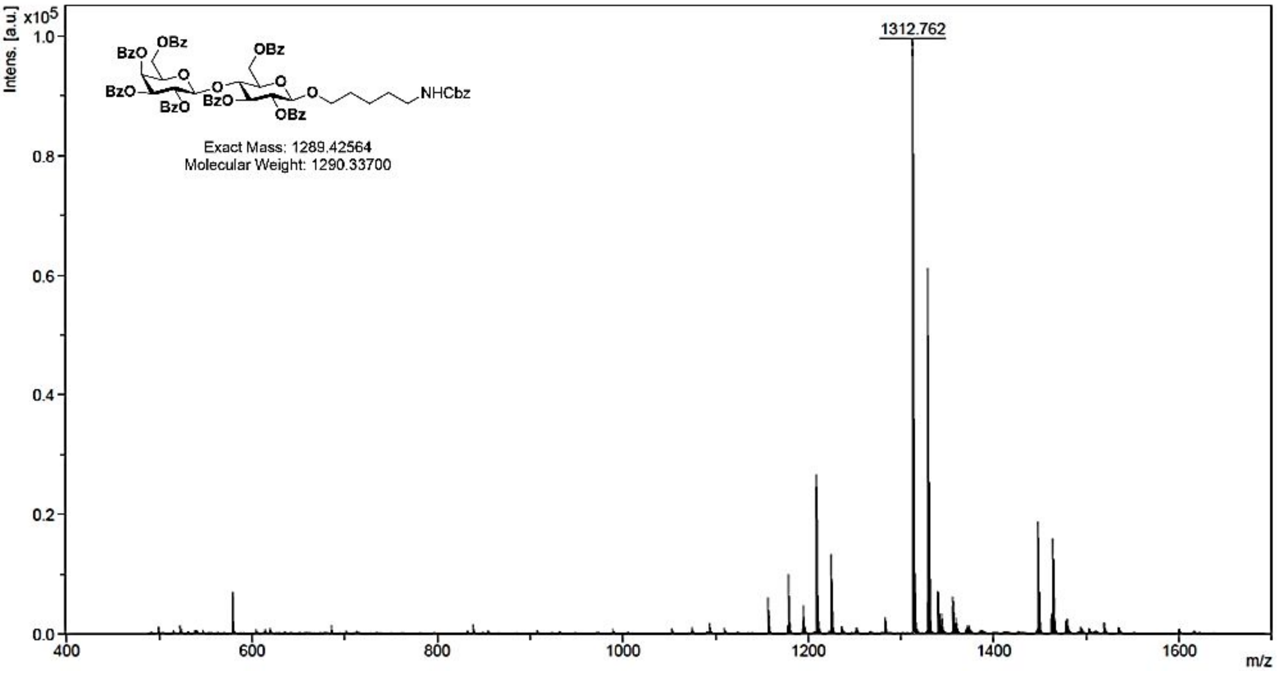

I. VaporSPOT synthesis on glass slide

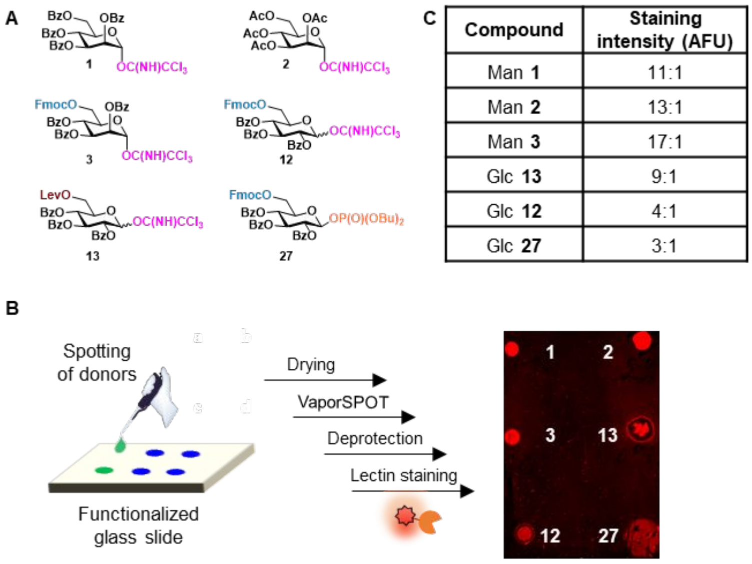

Compound	Staining intensity (AFU)
Man **1**	11:1
Man **2**	13:1
Man **3**	17:1
Glc **13**	9:1
Glc **12**	4:1
Glc **27**	3:1

Figure S10: *In-situ* glycosylation on functionalized glass slides using VaporSPOT: A) Building blocks **1-3**, **12**, **13** and **27** spotted onto the glass slide. B) Building blocks were spotted on defined positions on the glass slide, dried, followed by VaporSPOT glycosylation, deprotection, and ConA staining to visualize the formed structures after fluorescence scan. C) Signal to noise ratio of staining.

Lastly, to display the flexibility and usefulness of this approach, we have employed the vaporSPOT approach on a glass slides for *in-situ* synthesis of an array (Figure S10). Cleavage of the ester and carbonate groups was performed using NaOMe in MeOH , while the synthesized dimers remained attached to the solid support. Subsequently, the compounds were stained using Concanavalin A (ConA) and the result was screened after fluorescence scan.

For vaporSPOT synthesis on a glass solid support, a commercially available 3D amino glass slide (PolyAn GmbH, Germany) was used. The slide was additionally functionalized with a 2-{[2-*O*-benzoyl-3,4-di-*O*-benzyl-6-*O*-(9-fluorenylmethoxycarbonyl)-β-D-galactopyranosyl]oxy}-acetic acid building block, forming an amide bond (data not shown, will be published elsewhere). After Fmoc deprotection, the free hydroxyl group on the C-6 position was used as the glycosyl acceptor. Then, mannopyranoside building blocks **1-3**, and glucopyranosides **12**, **13** and **27** (Figure S10, B) were dissolved in DCM (2 mg/20 µL of DCM) and spotted with an Eppendorf pipette (1 µL) to defined areas on the glass under inert conditions. Afterwards, the slide was transferred into the glycosylation chamber of our custom-built setup and cooled down to –5°C. Activation of the glycosyl donors, bearing different leaving groups, was achieved by deposition of the activator solution under vapor. The temperature was slowly increased to rt and the remaining condensate was removed from the glycosylation chamber under high vacuum. Then, the slide was transferred into a Petri dish and washed with dichloromethane and

dimethylformamide. Removal of the protecting groups (Bz, Ac, Lev, Fmoc) was performed using a solution of NaOMe in MeOH overnight. Detection of the deprotected oligosaccharides was achieved via direct staining with fluorescently labeled ConA (100 µg/mL) and scanned in a fluorescence scanner (632 nm excitation, Figure S10, B), showing a good fluorescence signal to noise (Figure S10, C).

References

(1) Bien, F.; Ziegler, T. Chemoenzymatic Synthesis of Glycosylated Enantiomerically Pure 4-Pentene 1,2- and 1,3-Diol Derivatives. *Tetrahedron: Asymmetry* **1998**, *9* (5), 781–790. https://doi.org/10.1016/S0957-4166(98)00052-4.

(2) Patel, M. K.; Vijayakrishnan, B.; Koeppe, J. R.; Chalker, J. M.; Doores, K. J.; Davis, B. G. Analysis of the Dispersity in Carbohydrate Loading of Synthetic Glycoproteins Using MALDI-TOF Mass Spectrometry. *Chem. Commun.* **2010**, *46* (48), 9119. https://doi.org/10.1039/c0cc03420g.

(3) Rio, S.; Beau, J.-M.; Jacquinet, J.-C. Synthesis of Glycopeptides from the Carbohydrate-Protein Linkage Region of Proteoglycans. *Carbohydr. Res.* **1991**, *219* (C), 71–90. https://doi.org/10.1016/0008-6215(91)89043-F.

(4) Zhang, T.; Li, X.; Song, H.; Yao, S. Ionic Liquid-Assisted Catalysis for Glycosidation of Two Triterpenoid Sapogenins †. *New J. Chem* **2019**, *43*, 16881. https://doi.org/10.1039/c9nj04271g.

(5) Ganesh, N. V.; Fujikawa, K.; Tan, Y. H.; Stine, K. J.; Demchenko, A. V. HPLC-Assisted Automated Oligosaccharide Synthesis. *Org. Lett.* **2012**, *14* (12), 3036–3039. https://doi.org/10.1021/ol301105y.

(6) Calin, O.; Eller, S.; Seeberger, P. H. Automated Polysaccharide Synthesis: Assembly of a 30mer Mannoside. *Angew. Chemie Int. Ed.* **2013**, *52* (22), 5862–5865. https://doi.org/10.1002/anie.201210176.

(7) Danglad-Flores, J.; Leichnitz, S.; Sletten, E. T.; Abragam Joseph, A.; Bienert, K.; Le Mai Hoang, K.; Seeberger, P. H. Microwave-Assisted Automated Glycan Assembly. *J. Am. Chem. Soc.* **2021**, *143* (23), 8893–8901. https://doi.org/10.1021/jacs.1c03851.

(8) Gomez, J. D. C.; Hagenbach, A.; Gerling-Driessen, U. I. M.; Koksch, B.; Beindorff, N.; Brenner, W.; Abram, U. Thiourea Derivatives as Chelating Agents for Bioconjugation of Rhenium and Technetium. *Dalt. Trans.* **2017**, *46* (42), 14602–14611. https://doi.org/10.1039/C7DT01834G.

(9) Sandbhor, M. S.; Soya, N.; Albohy, A.; Zheng, R. B.; Cartmell, J.; Bundle, D. R.; Klassen, J. S.; Cairo, C. W. Substrate Recognition of the Membrane-Associated Sialidase NEU3 Requires a Hydrophobic Aglycone. *Biochemistry* **2011**, *50* (32), 6753–6762. https://doi.org/10.1021/bi200449j.

(10) Liu, B.; Zhang, F.; Zhang, Y.; Liu, G. A New Approach for the Synthesis of O-Glycopeptides through a Combination of Solid-Phase Glycosylation and Fluorous Tagging Chemistry (SHGPFT). *Org. Biomol. Chem.* **2014**, *12* (12), 1892–1896. https://doi.org/10.1039/C3OB42430H.

(11) Wei, G.; Zhang, L.; Cai, C.; Cheng, S.; Du, Y. Selective Cleavage of Sugar Anomeric O-Acyl Groups Using FeCl3·6H2O. *Tetrahedron Lett.* **2008**, *49* (38), 5488–5491. https://doi.org/10.1016/j.tetlet.2008.07.035.

(12) Majumdar, D.; Zhu, T.; Boons, G. J. Synthesis of Oligosaccharides on Soluble High-Molecular-Weight Branched Polymers in Combination with Purification by Nanofiltration. *Org. Lett.* **2003**, *5* (20), 3591–3594. https://doi.org/10.1021/ol0352355.

(13) Gellerman, G.; Elgavi, A.; Salitra, Y.; Kramer, M. Facile Synthesis of Orthogonally Protected Amino Acid Building Blocks for Combinatorial N-Backbone Cyclic Peptide Chemistry. *J. Pept. Res.* **2001**, *57* (4), 277–291. https://doi.org/10.1046/j.1397-002x.2000.0780.x.

(14) Le Mai Hoang, K.; Pardo-Vargas, A.; Zhu, Y.; Yu, Y.; Loria, M.; Delbianco, M.; Seeberger, P. H. Traceless Photolabile Linker Expedites the Chemical Synthesis of Complex Oligosaccharides by Automated Glycan Assembly. *J. Am. Chem. Soc.* **2019**, *141* (22), 9079–9086. https://doi.org/10.1021/jacs.9b03769.
